# Novel Phenolic Constituents of *Pulmonaria officinalis* L. LC-MS/MS Comparison of Spring and Autumn Metabolite Profiles

**DOI:** 10.3390/molecules23092277

**Published:** 2018-09-06

**Authors:** Justyna Krzyżanowska-Kowalczyk, Łukasz Pecio, Jarosław Mołdoch, Agnieszka Ludwiczuk, Mariusz Kowalczyk

**Affiliations:** 1 Department of Biochemistry and Crop Quality, Institute of Soil Science and Plant Cultivation—State Research Institute, Czartoryskich 8, 24-100 Puławy, Poland; lpecio@iung.pulawy.pl (Ł.P.); jmoldoch@iung.pulawy.pl (J.M.); mkowalczyk@iung.pulawy.pl (M.K.); 2 Department of Pharmacognosy with Medicinal Plant Unit, Medical University of Lublin, Chodzki Str.1, 20-093 Lublin, Poland; aludwiczuk@pharmacognosy.org

**Keywords:** *Pulmonaria officinalis*, *Pulmonariae Herba*, lungwort, danshensu/caffeic acid/rosmarinic acid derivatives, HR-QTOF/MS, NMR, CD, seasonal variability, metabolite profiling, multivariate analyses

## Abstract

Lungwort (*Pulmonaria officinalis* L., Boraginaceae) is considered to possess therapeutic properties and it has been traditionally used as a remedy against various lung disorders in many countries. Nevertheless, very few data concerning its phytochemical composition are available. This research aims to provide a detailed description of specialized metabolites from the aerial parts of lungwort. Nine previously undescribed and 36 known phenolic compounds were detected in the 50% methanolic extract. Following multistep preparative procedures, structures of newly discovered compounds were determined using one- and two-dimensional techniques of NMR spectroscopy. Among the identified compounds were caffeic acid esters with aliphatic hydroxycarboxylic acids, conjugates of dicaffeic acid with rosmarinic acid, and previously unknown isomers of isosalvianolic acid A and yunnaneic acid E, as well as other lignans. Concentrations of all identified phenolic derivatives in the investigated herbal material were estimated using a method based on liquid chromatography with high-resolution mass spectrometry detection. Seasonal changes in the concentration of metabolites were also investigated using targeted and untargeted metabolomics techniques.

## 1. Introduction

*Pulmonaria officinalis* L (lungwort), belonging to the Boraginaceae family, is a herbaceous perennial plant, widely spread in Europe and western Asia. It has a long tradition of use in folk medicine of many countries as a remedy against various respiratory diseases including asthma, chronic bronchitis, tuberculosis, laryngitis, and coughs. It also has expectorant, antitussive, and diaphoretic properties [1,2,3,4,5,6]. Other ethnomedicinal sources indicate that infusions or decoctions of *P. officinalis* are administrated as astringent, anticoagulant, anti-microbial, and anti-inflammatory herbs, as well as a remedy for urinary disorders, cystitis, moreover, it shows diuretic and anti-lithiasis activities [2,7,8]. Applied externally, it can be very beneficial in the treatment of burns, wounds, cuts, and eczema [1,2]. *P. officinalis* extract was tested as a component of bioactive hydrogels, which can be used in the treatment of wounds with heavy and medium exudates [9]. Aerial parts of *P. officinalis,* commercially available as *Pulmonariae Herba*, in combination with *Tussilago farfara* (coltsfoot), are often used to treat chronic cough, including whooping cough [1]. *Pulmonariae Herba* is also an ingredient of various herbal mixtures or dietary supplements. Astringent, emollient, and skin conditioning properties allow for the use of *P. officinalis* extract in cosmetology [10,11]. Furthermore, *P. officinalis* and *P. obscura* are also known as wild food plants [12,13] and honey plants [14,15]. Various *Pulmonaria* species are also used as ornamental plants.

Very few pharmacological studies confirming the effects of the traditional use of *P. officinalis* and *P. obscura* are available. Also, only fragmentary research has been done regarding the chemical constituents of *Pulmonaria* species. Consequently, the phytochemical profile of *P. officinalis* remains mostly unknown, particularly regarding phenolic compounds, for which only a very few publications are available. Brantner and Karting, based on thin layer chromatography (TLC) identification, reported on the presence of quercetin and kaempferol glycosides [16]. A fingerprint of methanol extract of *P. officinalis* obtained using micro-two-dimensional TLC, indicated the presence of chlorogenic acid, myricetin, acacetin, glycosides of apigenin, quercetin (rutin and hyperoside), hesperetin (hesperidin), and naringenin (naringin) [1]. Furthermore, based on HPLC analysis, Neagu et al. reported that rosmarinic acid was the main constituent of both aqueous and ethanolic extracts obtained from *P. officinalis*, moreover small amounts of rutin, hyperoside, chlorogenic, and caffeic acids were also detected [17]. Our research revealed that *P. officinalis* extract contains yunnaneic acid B—a unique molecule that has been isolated so far only from *Salvia yunnanensis*, and also confirmed the presence of large amounts of rosmarinic acid [18].

Nevertheless, all of these reports provide an incomplete view of the phytochemicals that are present in lungwort. Thus, the primary goal of this research was systematic detection, isolation, and NMR-based identification of metabolites from the extract of *Pulmonaria officinalis* for dereplication purposes. Secondly, the obtained reference substances were used to investigate the distribution of identified metabolites in *P. officinalis*. Thirdly, we investigated changes in the phytochemical composition of *P. officinalis* at two phenological stages. This experiment was carried out using both targeted and untargeted metabolomics approaches, to discover components and potential biomarkers associated with each stage.

## 2. Results and Discussion

### 2.1. Identification of Metabolites in P. offcinalis Extract

**Figure 1:**
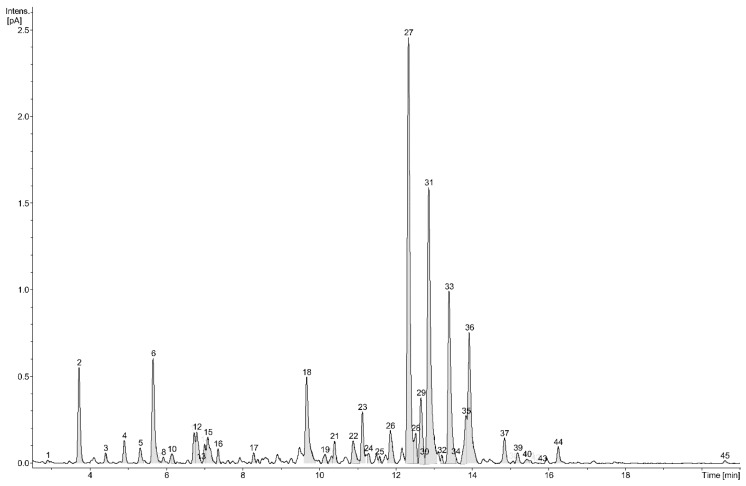
UHPLC profile of the *P. officinalis* 50% MeOH fraction (numbers indicate isolated compounds).

Preliminary chromatographic analyses of the extract from aerial parts of *P. officinalis* L. indicated the presence of several peaks tentatively identified as phenolic derivatives (Figure 1 and Table 1, peak numbers assigned by retention time). A multistep preparation procedure led to the isolation of 45 compounds which were further analyzed using high-resolution mass spectrometry, as well as one-dimensional ^1^H and ^13^C-NMR spectroscopy. Based on these results, we identified nine new and 36 already described in the literature metabolites.

Among the known compounds were mainly conjugates of danshensu ((2*R*)-(3,4-dihydroxyphenyl)-2-hydroxypropanoic acid) (**1**), with caffeic acid (**8**), such as shimobashiric (**26**), rosmarinic (**27**), monardic (**29**), lithospermic (**31**,**34**), salvianolic (**33**), and yunnaneic acids (**22**,**36**). Also present were several conjugates of phenolic acids with quinic acid, such as three isomers of chlorogenic acid (**6**,**9**,**13**) and two isomers of coumaroylquinic acid (**16**,**17**). Additionally, also detected were esters of caffeic acid with threonic and glyceric acid (**4**,**11**,**12**), lignans such as globoidnans A and B (**37**,**18**), a megastigmane glucoside (**7**), a few flavonol glycosides (kaempferol (**20**,**24**,**25**), or quercetin (**19**,**21**) derivatives), which were also present in malonylated forms (**23**,**28**), as well as a nitrile glucoside menisdaurin (**2**), and tryptophan derivative lycoperodine-1 (**5**).

### 2.2. Structural Characterization of the New Compounds

**Figure 2:**
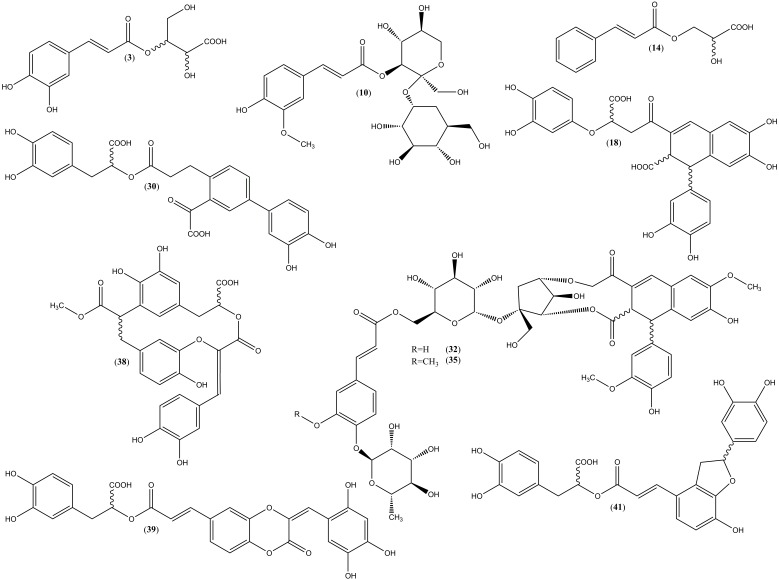
Structures of novel compounds isolated from the aerial parts of *P. officinalis*. (**3**—3-*O*-(*E*)-caffeoyl-threonic acid; **10**—3′−*O*-(*E*)-feruloyl-α-sorbopyranosyl-(2′→1)-α-glucopyranoside; **14**—3-*O*-(*E*)-caffeoyl-glyceric acid; **18**—globoidnan B; **30**—yunnaneic acid E-1; **32**—pulmonarioside A; **35**—pulmonarioside B; **38**—pulmitric acid A; **39**—pulmitric acid B; **41**—isosalvianolic acid A-1).

Complete structures of the nine newly discovered metabolites (**3**,**10**,**14**,**30**,**32**,**35**,**38**,**39**,**41**, shown in Figure 2) were elucidated by one-dimensional as well as two dimensional ^1^H and ^13^C-NMR spectroscopy.

The ^13^C-NMR of compound **3** showed 13 signals that were classified as one CH_2_, 7 CH, and 5 quaternary carbon atoms (Table 2). The aromatic region of the ^1^H and COSY (correlation spectroscopy) spectra of **3** exhibited the presence of set of protons characteristic for the α,β-unsaturated aromatic acid derivative, while the aliphatic alcohols region indicated the presence of another set of protons. The first set corresponded to a tri-substituted aromatic group at δ_H_ 7.04 (d, *J* = 2.1 Hz, H-2′), 6.94 (dd, *J* = 8.2, 2.1 Hz, H-6′), and 6.77 (d, *J* = 8.2 Hz, H-5′), in accordance with the AMX spin system, and coupled doublets, H-α and H-β, at δ_H_ 6.26 and 7.59, corresponding to *E*-(*J*_α,β_ = 15.9 Hz) olefinic moiety. The set of aliphatic protons corresponded to a tri-hydroxylated group at δ_H_ 5.34 (ddd, *J* = 6.8, 6.3, 2.5 Hz, H-3), 4.47 (d, *J* = 2.5 Hz, H-2), and H-4 at δ_H_ 3.81 (dd, *J* = 11.2, 6.9 Hz)/3.75 (dd, *J* = 11.2, 6.3 Hz), in accordance with the ABMX spin system. The assignments of all carbons of the aromatic and aliphatic moiety were accomplished by interpretation of the HSQC (heteronuclear single quantum correlation), H2BC (heteronuclear 2-bond correlation), and HMBC (heteronuclear multiple bond coherence) spectra, and indicated that compound **3** contained a *trans*-caffeic acid derivative connected through an ester bond with tri-hydroxylated sugar acid, namely threonic acid. It was supported by the ^3^J correlation between H-3 and C-9′ (δ_C_ 168.3), and a weak ^4^*J* correlation between H-α and C-3 (δ_C_ 75.8), observed in the HMBC spectrum, and suggested that **3** was 3-*O*-(*E*)-caffeoyl-threonic acid. Similar compounds: (−)-2-*O*-(*E*)-caffeoyl-l-threonic acid (**4**) and (−)-4-*O*-(*E*)-caffeoyl-l-threonic acid (**12**) were also found in *Crataegus* extract [19], leaves of *Dactylis glomerata* [20], leaves of *Cornus controversa* [21], and aerial parts of *Chelidonium majus* [22]. Therefore, compound **3** was elucidated as 3-*O*-(*E*)-caffeoyl-threonic acid.

The ^13^C-NMR of compound **14** showed 12 signals that were classified as one CH_2_, six CH, and five quaternary carbon atoms (Table 2). The aromatic region of the ^1^H and COSY spectra of **14**, similarly to **3**, exhibited the presence of set of protons, characteristic for the α,β-unsaturated aromatic acid derivative, while the aliphatic alcohols region showed the presence of a second set of protons. One set corresponded to a tri-substituted aromatic group at δ_H_ 7.04 (d, *J* = 2.1 Hz, H-2′), 6.94 (dd, *J* = 8.2, 2.1 Hz, H-6′), and 6.78 (d, *J* = 8.2 Hz, H-5′), in accordance with the AMX spin system, and coupled doublets, H-α and H-β, at δ_H_ 6.27 and 7.58, corresponding to E-(*J*_α,β_ = 15.9 Hz) olefinic moiety, typical of a *trans*-caffeoyl residue. The set of aliphatic protons corresponded to a di-hydroxylated group at δ_H_ 4.44 (br d, *J* = 6.0 Hz, H-2), and H-3 at δ_H_ 4.46 (m)/4.38 (m), which were correlated in the HSQC spectrum with their carbon atoms at δ_C_ 70.3 and 66.9 ppm, respectively. Protons H-2/H-3 correlated in the HMBC spectrum, with carbons C-1 (δ_C_ 174.8) and C-9′ (δ_C_ 168.9), thus revealing that the aliphatic portion of **14** was glyceric acid attached to caffeoyl moiety through an ester bond, forming a 3-*O*-caffeoyl-glyceric acid. The other structural isomer of caffeoyl-glyceric acid, found in the investigated plant, was (−)-2-*O*-(*E*)-caffeoyl-d-glyceric acid [22]. Thus **14** was identified as 3-*O*-(*E*)-caffeoyl- glyceric acid.

**Figure 3:**
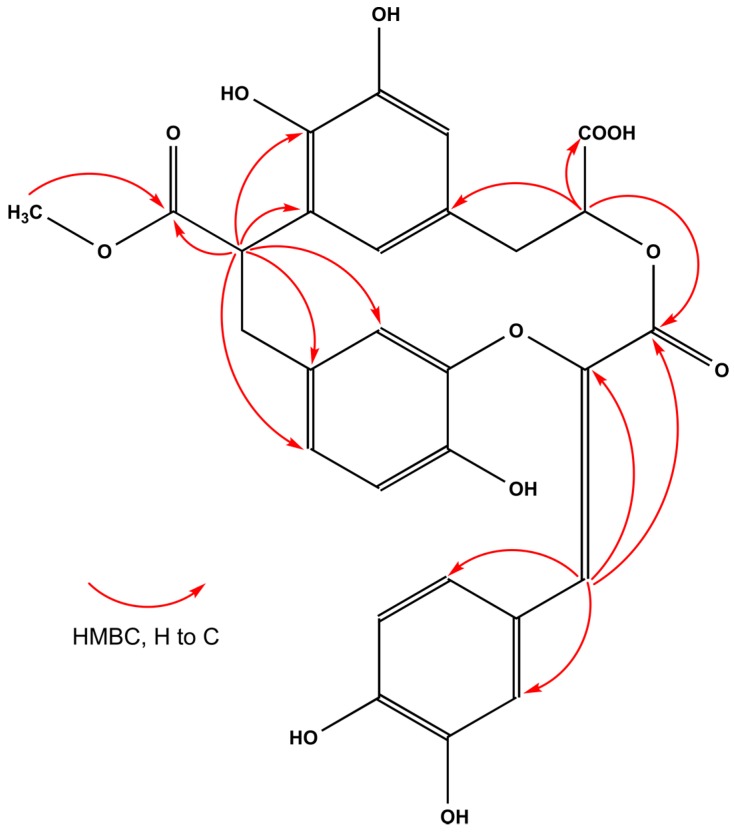
Selected HMBC correlations of compound **38**.

The ^13^C-NMR (DEPTQ-135) (distorsionless enhancement by polarization transfer including the detection of quaternary nuclei) spectrum of **38** contained 28 signals, sorted by HSQC and HMBC spectra, as one CH_3_, two CH_2_, 11 CH, and 14 quaternary carbon atoms (Table 3). The high-field shifted resonances at δ_C_ 174.5, 172.7, and 164.2 suggested the presence of three carboxyl groups (C-9, C-9′, and C-9′′, respectively). The aromatic region of the ^1^H and COSY spectra of **38** revealed the presence of three aromatic rings in the structure. Two of them were 1,3,4-trisubstituted benzene rings—first at δ_H_ 6.98 (br s, H-2), 6.84 (dd, *J* = 8.1, 2.1 Hz, H-6), 6.67 (d, *J* = 8.1 Hz, H-5) and second at δ_H_ 7.40 (d, *J* = 2.1 Hz, H-2″), 7.16 (dd, *J* = 8.3, 2.1 Hz, H-6″), 6.79 (d, *J* = 8.3 Hz, H-5″)—both in accordance with the AMX spin system. The third represented a 1,3,4,5-tetrasubstituted benzene ring and showed *meta*-coupled resonances at δ_H_ 6.52 (d, *J* = 1.9 Hz, H-2′) and 6.72 (br s, H-6′). The signals of the two downfield shifted aliphatic protons at δ_H_ 5.23 (dd, *J* = 11.4, 1.6 Hz, H-8′) and 4.83 (t, *J* = 8.2 Hz, H-8), after selective irradiation in 1D-TOCSY (total correlation spectroscopy) experiments, showed correlation with the CH_2_ groups at δ_H_ 3.15 (dd, *J* = 14.2, 1.8 Hz, H-7′)/2.98 (dd, *J* = 14.2, 11.4 Hz, H-7′), and 2.99 (dd, *J* = 15.4, 8.5 Hz, H-7)/2.96 (dd, *J* = 15.4, 7.6 Hz, H-7), forming AMX and ABX spin systems, respectively. Proton H-8′ correlated in the HSQC spectrum with a carbon at δ_C_ 75.3, while proton H-8 correlated with a carbon at δ_C_ 39.3, suggesting that the first one was an oxygenated methine. The ^1^H-NMR spectrum also revealed two sharp singlets, at δ 7.13 (H-7″) and 3.54 (9-OMe), correlating in the HSQC spectrum with carbons at δ 127.0 and 52.1 (respectively), suggesting the presence of a tri-substituted (*Z*)-double bond [23] and a methyl ester function. This evidence suggested that **38** was a dicaffeic acid-(3,4-dihydroxyphenyl lactic acid) conjugate. The long-range correlations visible in the HMBC spectrum between H-7″and C-8″(δ_C_ 140.2)/C-9″and H-8′ and C-9′′/C-9′ suggested that the core structure of **38** was rosmarinic acid (**27**). Furthermore, the long-range correlations observed in the HMBC spectrum between H-8 and carbons C-1, C-5′ (δ_C_ 132.8), and C-9 unambiguously proved the presence of a dihydrocaffeic acid methyl ester attached to C-5′ and C-8″of the rosmarinic acid, forming a 14-carbon close-ring structure (Figure 3). It was further supported by the NOE effect observed in the NOESY (nuclear Overhauser effect spectroscopy) spectrum between protons H-2 and H-6′, and substantial broadening of their signals, as it suggested the presence of steric hindrance to free rotation, and their close vicinity. Compound **38** was named pulmitric acid A.

The ^13^C-NMR (DEPTQ-135) spectrum of **39** contained 27 signals, sorted by HSQC and HMBC spectra, as one CH_2_, 12 CH, and 14 quaternary carbon atoms (Table 3). The NMR spectra suggested the presence of three phenylpropanoid moieties, similarly to **38**, with rosmarinic acid being the core structure. One ABX spin system at δ_H_ 7.37 (dd, *J* = 8.3, 2.1 Hz, H-6), 7.35 (d, *J* = 2.1, H-2), and 6.99 (d, *J* = 8.3 Hz, H-5), and one AMX proton set at δ_H_ 6.73 (d, *J* = 2.1 Hz, H-2′), 6.68 (d, *J* = 8.1 Hz, H-5′), and 6.60 (dd, *J* = 8.1, 2.1 Hz, H-6′); together with two (*E*)-olefinic protons at δ_H_ 7.59 (d, *J* = 15.9 Hz, H-7), and 6.36 (d, *J* = 15.9 Hz, H-8) appeared in the aromatic part of the COSY spectrum. The ^13^C-NMR spectrum also showed two aliphatic carbons at δ_C_ 74.7 (C-8′), 37.9 (C-7′) and three carboxyl groups at δ_C_ 173.4 (C-9′), 168.1 (C-9) and up-field shifted one at 160.2 (C-9″). Additionally, the ^1^H-NMR spectrum contained three sharp singlets at δ_H_ 7.03 (H-7″), 6.81 (H-6″) and 6.79 (H-3″). Altogether, compound **39** seemed to possess a structure similar to that of lycopic acid, but with 3,4,5-trihydroxycinnamic instead of caffeic acid [24]. The proton H-7″showed a correlation in the NOESY spectrum with H-6′′, but quite surprisingly also with H-2, confirming the (*E*)-stereochemistry of the double bond and the site of conjugation with a rosmarinic acid moiety. It was further supported by the substantial downfield shift of C-2 (Δδ + 6.3) and C-4 (Δδ + 2.7), and the upfield shift of C-3 (Δδ − 2.7), when compared to that of rosmarinic acid (see Supplementary Materials Figure S48). Compound **39** was named pulmitric acid B.

Compounds **40** and **41** were isolated as separated chromatographic peaks using preparative HPLC. However, they presented a set of identical ^1^H and ^13^C-NMR resonances, in agreement with the structure of isosalvianolic acid A, found in *Mentha* species [25,26]. Compound **40** was recognized as isosalvianolic acid A (7R,8′′R), from the value of its optical rotation (${\left[\alpha \right]}_{D}^{23}$ = +39.7°), similar to that of rosmarinic acid. To investigate that in more detail CD (circular dichroism) spectra of **40** were recorded. As the C-8″chiral center and its environment are identical to that of rosmarinic acid, one would expect a similar CD spectrum if the configuration around C-8″is the same as in **27**. In fact, the CD spectra were only quantitatively different (see Supplementary Materials Figure S48 and S96). The values of the chemical shifts of compounds **40** and **41** were identical with the literature data [27]. On the other hand, optical rotation (${\left[\alpha \right]}_{D}^{23}$ = +18.5°) and the negative Cotton effect observed in the CD spectrum at 240–270 nm, suggested that the absolute stereochemistry of C-7 in **41** was (S). Therefore, compound **41** was named isosalvianolic acid A-1.

**Figure 4:**
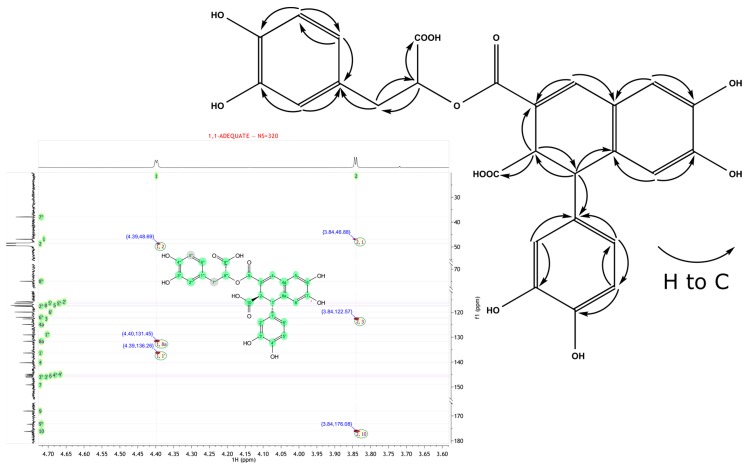
1,1-ADEQUATE spectrum and key correlations of compound **18**.

The ^13^C-NMR of compound **18** showed 27 signals that were classified as one CH_2_, 12 CH, and 14 quaternary carbon atoms (Table 4). The UV and MS spectral properties, as well as the ^1^H and ^13^C-NMR chemical shifts, were almost superimposable with that of salvianolic acid *R* (note the different enumeration of carbons) in [28]. However, the chemical structure of **18** was in agreement with globoidnan B, as presented in [29]. The structures differed in the location of a 3,4-dihydroxyphenyllactic moiety, connected with the core moiety of epiphyllic acid through an ester bond, either with C-9 or C-10 [30]. Our findings suggest that NMR chemical shifts are in agreement with globoidnan B, and are based on the careful study of both HMBC correlations and the analysis of 1,1-ADEQUATE (adequate double-quantum transfer experiment) spectrum and unambiguously identify the site of esterification (Figure 4). Additionally, we presented the optical rotation and CD spectra of this compound.

**Figure 5:**
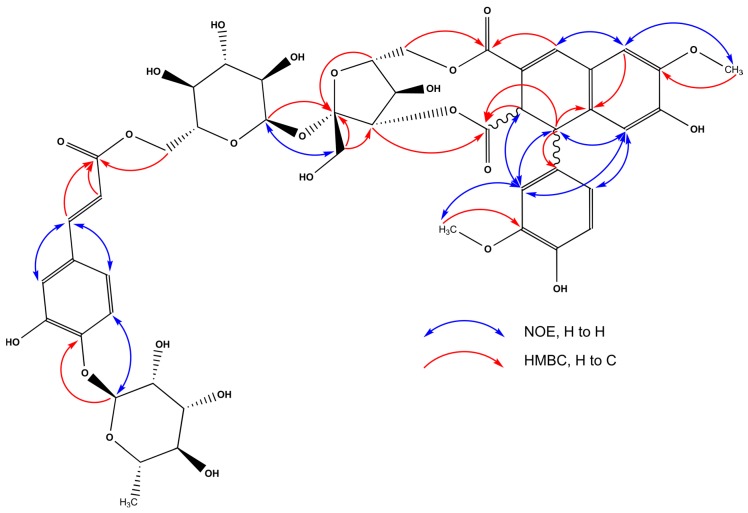
Selected HMBC and NOE correlations of compound **32.**

The ^13^C-NMR of compound **32** showed 47 signals that were classified as three CH_3_, three CH_2_, 26 CH, and 15 quaternary carbon atoms (Table 4). The aromatic region of the ^1^H and COSY spectra of **32** exhibited the presence of two sets of aromatic protons, in accordance with AMX spin systems, at δ_H_ 7.18 (d, *J* = 8.4 Hz, H-5′′″), 7.07 (d, *J* = 2.1 Hz, H-2′′″), 7.01 (dd, *J* = 8.4, 2.1 Hz, H-6′′″), and at δ_H_ 6.90 (d, *J* = 8.1 Hz, H-5′), 6.87 (d, *J* = 2.0 Hz, H-2′), 6.81 (dd, *J* = 8.1, 2.0 Hz, H-6′). The first one was part of the caffeoyl moiety, with (*E*)-oriented double bond with protons resonating at δ_H_ 7.47 (d, *J* = 15.9 Hz, H-7′′″), 6.21 (d, *J* = 15.9 Hz, H-8′′″), and correlated in the HMBC spectrum with the carbonyl group at δ_C_ (168.9, C-9′′″), which suggested the possible site of esterification. The other set was evidenced as part of an 1,2-dihydronaphtalene moiety—similarly to **18**, additionally methylated in positions C-6 (δ_C_ 147.7) and C-3′ (δ_C_ 149.0), which was further supported by the HMBC correlations and the NOE effects visible in the TROESY (transverse rotating-frame Overhauser enhancement spectroscopy) spectra between 6-OMe (δ_H_ 3.79, s) and H-5 (δ_H_ 6.73, s), and between 3′-OMe (δ_H_ 3.84, s), and H-2′ (Figure 5). This part of the molecule can also be recognized as the 8,8′-diferulic acid, in its aryltetralin form, often present in the plant cell-walls [31]. However, the relative configuration of H-1/H-2 must be different when compared to globoidnan B (*gauche)*. This assumption was based on the large (*J* = 15.3 Hz) coupling constant between H-1 (δ 4.34, dd, *J* = 15.3, 1.0 Hz, 1H) and H-2 (δ 4.21, dd, *J* = 15.3, 2.5 Hz, 1H), and allylic coupling (^4^*J*) between H-2 and H-4 (δ 7.39, d, *J* = 2.5 Hz, 1H), suggesting that orientation of C-2–H-2 is probably *quasi*-axial [32]. Additionally, the NOE effect observed in the TROESY spectrum between protons H-8 (δ 6.11, s)/H-2′ and H-8/H-6′ indicated that the orientation of C-1–H-1 is *quasi*-axial too and therefore the relative configuration of H-1/H-2 is *anti* [32]*.* Moreover, two anomeric proton signals at δ_H_ 5.50 (d, *J* = 1.8 Hz, H-1′′′″) and 5.35 (d, *J* = 3.6 Hz, H-1′″) were observed, indicating the presence of two sugar units. Based on the values of coupling constants, and the analysis of 1D TOCSY and 1D TROESY, COSY, HSQC, H2BC, HSQC-TOCSY, F2-coupled HSQC [33], and HMBC data, the two sugar units were elucidated as α-rhamnopyranoside δ_H/C_ 5.50 (H-1′′′″)/100.7 (C-1′′′″), and α-glucopyranoside δ_H/C_ 5.35 (H-1′″)/94.4 (C-1′″) (Table 4). The α orientation of anomeric protons was supported by their ^1^*J*_CH_ coupling constants, with values of ~172 and ~169 Hz [34], respectively, measured in the F2-coupled HSQC experiment. Additionally, the ^3^*J* correlations observed in the HMBC spectrum between the anomeric proton of the Glc (H-1′″) and C-2″of a β-fructofuranose unit (δ_C_ 110.1) indicated the presence of interglycosidic linkage between these sugar units (1→2), forming a sucrose unit. This was further supported by the NOE effect detected in the TROESY spectrum between H-1′″and CH_2_-1″(δ_H_ 3.90, d, *J*= 12.4 Hz/3.72, d, *J* = 12.4 Hz). Careful examination of correlations visible in the HMBC spectrum allowed to assign connectivities between the glycosidic and aromatic parts of **32** unambiguously. Therefore, the correlation between H-1′′′″and C-4′′″(δ_C_ 147.9) of caffeoyl moiety proved a Rha unit to be the last part of the glycosidic chain. It was further supported by the NOE effect in the TROESY spectrum between H-1′′′″and H-5′′′′. Next, geminal protons of CH_2_-6′″group of Glc (δ_H_ 4.48, dd, *J* = 12.0, 2.3 Hz/4.16, dd, *J* = 12.0, 6.5 Hz) gave a ^3^*J* correlation in the HMBC spectrum with C-9′′″of Caf moiety. Finally, the geminal protons of Fru of CH_2_-6″group (δ_H_ 4.71, dd, *J* = 12.4, 2.4 Hz/4.08, d, *J* = 12.4 Hz) gave a ^3^*J* correlation in the HMBC spectrum with C-9 (δ_C_ 168.1), while the proton H-3″(δ_H_ 4.63) showed correlation to C-10 (δ_C_ 176.4) of the core moiety (8,8′-diferulic acid). Hence, the structure of **32** was elucidated, and we propose its trivial name to be pulmonarioside A.

The ^1^H and ^13^C-NMR spectra of compound **35** showed an almost identical set of atoms, with only one additional CH_3_ group located at C-3′′″(δ_C_ 152.0), when compared to **32** (Table 4). In a chain of glycosidation, type of sugar units was precisely the same (with 8,8′-diferulic acid as the core of the molecule), the only difference was the presence of the (*E*)-ferulic acid instead of caffeoyl moiety. This was supported by the long-range correlation that was visible in the HMBC spectrum of **35** between 3′′″-OMe (δ_H_ 3.92, s) and C-3′′′′, and the NOE effect was visible in the TROESY spectrum between proton mentioned above and H-2′′″(δ_H_ 7.26, d, *J* = 2.0 Hz). Hence, the structure of **35** was elucidated, and we proposed its trivial name to be pulmonarioside B.

The ^13^C-NMR of compound **10** showed 22 signals that were classified as one CH_3_, three CH_2_, 13 CH, and five quaternary carbon atoms (Table 5). The aromatic region of the ^1^H and COSY spectra of **10** exhibited the presence of aromatic protons, in accordance with the AMX spin system, at δ_H_ 7.23 (d, *J* = 2.0 Hz, H-2″), 7.14 (dd, *J* = 8.3, 2.0 Hz, H-6″), 6.81 (d, *J* = 8.3 Hz, H-5″), and α/β-unsaturated side chain with (*E*)-stereochemistry (^3^*J*_HH_ coupling constant of 15.9 Hz). Additionally, the NOE effect observed in the TROESY spectrum between H-2″and 3″-OMe (δ_H_ 3.91, s) and the ^3^*J* correlation that was visible in the HMBC spectrum between 3″-OMe and C-3″(δ_C_ 149.4) confirmed the presence of the (*E*)-ferulic acid as part of **10**. It was connected through the ester bond with the disaccharide unit, identified as digobiose [35]. This was supported by the HMBC correlation between H-3′ (δ_H_ 5.46, d = 7.9 Hz) and C-9″(168.3). The interglycosidic linkage between sugar moieties was established as 1→2, from the ^3^*J* correlation that was visible in the HMBC spectrum (H-1 at δ_H_ 5.44 to C-2′ at δ_C_ 104.8). The α-orientation of the glucopyranose moiety was based on the small vicinal coupling constant of H-1 (^3^*J*_HH_ = 3.7 Hz), with H-2 (δ_H_ 3.43, dd, *J* = 9.8, 3.7) and ^1^*J*_CH_ coupling constant between an anomeric pair of resonances H/C (~169 Hz). The α orientation of the sorbopyranose unit was based on the NOE effect between H-3′ and both H-5′/CH_2_-1′ at δ_H_ 3.93 (ddd, *J* = 7.9, 5.6, 3.5 Hz)/(3.66, d, *J* = 12.2 Hz and 3.59, d, *J* = 12.2 Hz), respectively, and comparison of chemical shifts and coupling constants with the literature data. Therefore, the structure of **10** was established as 3′-*O*-(*E*)-feruloyl-α-sorbopyranosyl-(2′→1)-α-glucopyranoside.

Compound **30** was isolated as the minor constituent of *P. officinalis,* and its ^13^C-NMR showed 26 signals that were classified as three CH_2_, 10 CH, and 13 quaternary carbon atoms (Table 5). The spectral features of this compound suggested that it possessed both 3,4-dihydroxyphenyllactic acid and 4-biphenylpropionic acid moieties conjugated through an ester linkage. It was evidenced by the presence of three sets of aromatic protons in the ^1^H and COSY NMR spectra. The one was in accordance with the ABX spin system at δ_H_ 6.73 (d, *J* = 2.1 Hz, H-2″), 6.70 (d, *J* = 8.1 Hz, H-5″), 6.59 (dd, *J* = 8.1, 2.1 Hz, H-6″), consistent with a 1,3,4-trisubstituted aromatic ring. The other two sets were in accordance with the AMX spin systems at δ_H_ 7.87 (d, *J* = 2.0 Hz, H-2), 7.67 (dd, *J* = 8.0, 2.0, H-6), 7.19 (d, *J* = 8.0 Hz, H-5), and at δ_H_ 7.04 (d, *J* = 2.2 Hz, H-2′), 6.94 (dd, *J* = 8.2, 2.2 Hz, H-6′), 6.85 (d, *J* = 8.2 Hz, H-5′), consistent with a 3,3′,4′-trisubstituted 4-biphenylpropionic acid unit. It was further supported by the long-range correlations visible in the HMBC spectrum between H-8″(δ_H_ 5.10, dd, *J* = 8.9, 4.0 Hz) and C-9 (δ_C_ 173.8) presenting the ester linkage, and H-6 to C-1′ (δ_C_ 132.4) with H-2′/H-6′ to C-1 (δ_C_ 141.2). It was consistent with the NOE correlations that were visible in the TROESY spectrum between pairs H-2/H-6 and H-2′/H-6′, confirming the presence of the biphenyl moiety. One ambiguity arose during the elucidation of the structure of **30**—the ^13^C-NMR spectrum showed the presence of resonances at δ_C_ 167.8 and 192.0. The latter was attributable to the oxo- group in the C-10 position from the long-range correlation visible in the HMBC spectrum between H-2 and C-10. However, there was no visible correlation to the resonance at 167.8 ppm, and no aldehyde-type proton that was visible in the ^1^H-NMR spectrum, but the chemical formula established through HR-MS required the presence of 26 carbons. Therefore, we suggest that the α-oxoacid moiety is present in the structure of **30**, located in the carbons C-10 and C-11. This compound showed several similarities to the already known compound—yunnaneic acid E [36], and we proposed its trivial name to be yunnaneic acid E-1.

### 2.3. The Main Phytochemical Constituents of P. officinalis

For quantitative analysis of *P. officinalis* extracts, we employed a method based on liquid chromatography with high resolution mass spectrometric detection. Metabolites isolated for structure determination and confirmation were used as reference standards, and digoxin was used as the internal standard. Results of these measurements are shown in Table 6.

Rosmarinic acid (**27**)—a depside composed of (2*R*)-3-(3,4-dihydroxyphenyl)-lactic and caffeic acid residues, was confirmed as the chief constituent of examined extracts. Although the observed content of rosmarinic acid was relatively high, ranging from 7 to 12 mg/g of dry weight (DW), we previously observed much higher levels (approaching 60 mg/g DW) in similar samples [18]. Reasons for such a broad diversity in the concentration of this compound are unclear. However, comparable levels were reported from other species [37,38]. Rosmarinic acid exerts a variety of well-documented pharmacological properties, such as antioxidant, anti-inflammatory, antibacterial, anti-angiogenic, anti-mutagenic, antidepressant, and neuroprotective, as well as antiallergenic [39,40,41,42,43]. Due to a high content of rosmarinic acid, such properties can also be attributed to whole extracts or tinctures of lungwort. Investigated samples also contained small amounts of methyl rosmarinate (**43**) [44].

Shimobashiric acid C (**26**) was another phenolic acid derivative that was abundantly present in the *P. officinalis* extract (1.2–1.8 mg/g DW)*.* It is a dimer of rosmarinic acid containing cyclobutane scaffold (truxillic acid) formed presumably by a [2 + 2] photocycloaddition of two olefinic moieties [45]. Shimobashiric acid C was isolated from *Keiskea japonica* [46], and recently from *Plectranthus amboinicus* [45]. However, its activity has been poorly studied. It can act as a hyaluronidase inhibitor [46] and also possesses anti-inflammatory properties. Chen et al. confirmed that this molecule inhibited the binding of the AP-1 transcription factor to its consensus DNA sequence, and showed TNF-α inhibitory activity as well [45].

Another significant phenolic derivative observed in extracts of *Pulmonaria* was lithospermic acid A (**31**, approximately 0.6 mg/g DW). Identified by the presence of dihydrobenzofuran moiety with (7*S*,8*S*) configuration [47], lithospermic acid A is one of the major constituents of Chinese medicinal plant *Salvia miltiorrhiza* Bge., although it was observed in several other plants [44,48,49,50,51,52,53,54]. It has substantial therapeutic potential indicated during in vitro tests [52,55,56,57,58,59]. Extracts from *Pulmonaria* species also contained slightly higher levels (0.8–0.97 mg/g DW) of monardic acid A (**29**), an isomer of lithospermic acid A possessing (7*R*,8*R*) configuration [60,61]. Lithospermic acid B (**34**), a minor component of the extract possessing various biological activities [57,62,63], was also detected but not quantified due to an insufficient amount of suitable reference standard. *Pulmonaria* also contains yunnaneic acid B (**36**, 0.2–1.8 mg/g DW) [44,50,64], and yunnaneic acid E (**22**, 0.1–0.2 mg/g DW) in smaller quantities, [36,44]. Although yunnaneic acid B was isolated and characterized in the mid-90s, there is no available data on its activity and occurrence in plants. Our recent study revealed its ability to reduce oxidative damage to blood plasma proteins and lipids, and to enhance the non-enzymatic antioxidant capacity of blood plasma *in vitro* [18].

Salvianolic acid H (**33**, 3′-*O*-(8″-Z-caffeoyl) rosmarinic acid) and its methyl ester (**44**) were also detected at low levels (0.03–0.26 and 0.006–0.03 mg/g DW, respectively). However, their presence can be, at least partially, attributed to the transformation of another compound, lycopic acid C (**45**), during the extraction and isolation processes [24]. Treatment of lycopic acid C with water for three days was reported to produce 3-*O*-(caffeoyl) rosmarinic acid [65], and similar treatment with methanol produced its methyl ester. Nevertheless, the presence of salvianolic acid H was confirmed in some species from genus Salvia, such as *Salvia cavaleriei* [51], and *Salvia miltiorrhiza* [48,50].

In addition to rosmarinic acid derivatives, *Pulmonaria* also appears to have an ability to synthesize chlorogenic acids, as evidenced by the presence of chlorogenic (**6**, 0.2–0.3 mg/g DW), crypto-chlorogenic (**9**, 0.005–0.03 mg/g DW), and neo-chlorogenic (**13**) acid (0.02–0.03 mg/g DW) in the investigated extracts. All these compounds showed the same ion [M − H]^−^ at *m*/*z* 353. Compound **6** was identified as 3-*O*-caffeoylquinic acid, whereas compound **9** was identified as 4-*O*-caffeoylquinic acid, by comparisons of MS/MS fragmentation patterns and retention times with that of authentic standards. The last isomer was only tentatively identified as a 5-*O*-caffeoylquinic acid (**13**) by its fragmentation pattern alone [66,67].

3*-O-p-*coumaroylquinic acid (**15**), preliminarily identified using HR-QTOF-MS/MS (high resolution quadrupole-time of flight tandem mass spectrometry) and subsequently isolated and confirmed using NMR techniques, was detected at levels (0.1–0.36 mg/g DW) that were comparable to that of its sister molecule, chlorogenic acid. Isomeric 4*-O-p-*coumaroylquinic (**16**) and 5*-O-p-*coumaroylquinic (**17**) acids, tentatively identified by the MS/MS fragmentation patterns [66], also occur in *Pulmonaria*, although the former is a relatively minor component, detected only in samples collected in autumn.

Free danshensu (**1**) [44] and caffeic acid (**8**) [44] were also present in low quantities (0.02–0.06 and 0.02–0.1 mg/g DW, respectively). Both of these compounds showed diverse and significant bioactivities in previous studies [68].

Lignans were abundantly represented by globoidnan B (**18**, 3.8–6.8 mg/g DW), and a few other components detected at trace levels. That latter group, included also newly discovered pulmonariosides A and B, as well as globoidnan A (0.02 mg/g DW). Nothing is known about biological activities of globoidnan B. However, globoidnan A was previously isolated from *Eucalyptus globoidea* [69], *Origanum minutiflorum* [70], and *Thymus praecox* [71] and extensively investigated. It is known to inhibit the action of HIV integrase, an enzyme which is responsible for the introduction of HIV viral DNA into a host′s cellular DNA [69]. Moreover, it revealed anti-proliferative activity against HeLa (human cervix carcinoma) or C6 (rat brain tumor) cell lines [71]. 

Lungwort also contains a relatively limited number of flavonol glycosides. Tentative identifications of these compounds by HR-MS (high m-resolution ass spectrometry) were confirmed by full characterization using NMR techniques. Malonylated glucosides of quercetin (**23**, 0.9–1.6 mg/g DW) and kaempferol (**28**, 0.7–1.6 mg/g DW) were the most abundant among the detected flavonol glycosides. Rutin (**19**, (quercetin-3-*O*-rutinoside)) was also present in moderate quantities (0.05–0.36 mg/g DW). The other flavonol-derived glycosides (**21**,**24**,**25**) were detected at slightly lower levels, although some significant seasonal changes in their concentrations were observed. Although rutin, isoquercitin (quercetin-3-*O*-*β*-glucoside), and astragalin were described before in the aerial parts of *P. officinalis* [16], and the presence of their malonyl derivatives was previously not discovered. However, in contrast to Bratner′s research, we did not detect quercitrin (quercetin-3-*O*-rhamnoside). Also, we cannot confirm the presence of myricetin, acacetin, glycosides of apigenin, quercetin (hyperoside), hesperetin (hesperidin), or naringenin (naringin) found in *P. officinalis* extract by Hawrył and Waksmundzka-Hajnos [1]. 

The presence of menisdaurin (**2**), a nitrile glucoside, was confirmed by extensive spectroscopic HR-MS and NMR analyses and comparison with literature data [72]. The occurrence of this compound in *Pulmonaria officinalis* was so far unknown. Menisdaurin was detected in a moderate amount, approximately 0.1 mg/g DW, exclusively in the samples collected during spring. Very few members of Boraginaceae family are known to contain nitrile glucosides. Compounds structurally related to menisdaurin glucosides containing cyclohexenylcyanomethylene aglycones, such as lithospermoside (griffonin) and ehretiosides, were reported from various species of *Lithospermum* and *Ehretia*. Menisdaurin itself was also reported from genus *Tiquilia*. However, these plants are only distantly related to *Pulmonaria*. Although generally considered to be non-cyanogenic, menisdaurin gave positive results for HCN release in the tests carried out by Siegler et al. [72]. Menisdaurin elicits moderate anti-hepatitis B virus activity by inhibiting HBV DNA replication [73,74]. Its anti-tumor [75], as well as anti-inflammatory [75] activity were also reported.

Similarly to menisdaurin, tryptophan-derived tetrahydro-β-carboline alkaloid, lycoperodine-1 (1,2,3,4-tetrahydro-β-carboline-3-carboxylic acid, [76] was also present only in the spring samples, albeit at a much lower concentration (approximately 0.01 mg/g DW). Compounds similar to lycoperodine-1 are known to exhibit a variety of biological activities [77]. However, at the observed concentration, lycoperodine-1 is unlikely to contribute to the therapeutic properties of *P. officinalis*.

The lungwort extract also contained a small amount of megastigmane glucoside, actinidioionoside [78,79] (**7**, 0.02–0.4 mg/g DW), previously isolated from several plants, including distantly related to *P. officinalis* borage (*Borago officinalis* L.) [80].

### 2.4. Seasonal Fluctuation in Phytochemical Composition of P. officinalis

Numerous reports describe seasonal changes in the contents of flavonoid and phenolic acid derivatives in various plant species [81,82,83,84,85]. Although a variety of different accumulation patterns were observed, it is often stated in the literature that seasonal changes in phenolic acids contents follow a trend that is frequently the opposite to the direction of changes in flavonoid glycosides concentration. To investigate the seasonal variability of flavonoids and phenolic acids in *P. officinalis*, we first applied the untargeted metabolomics approach. 

**Figure 6:**
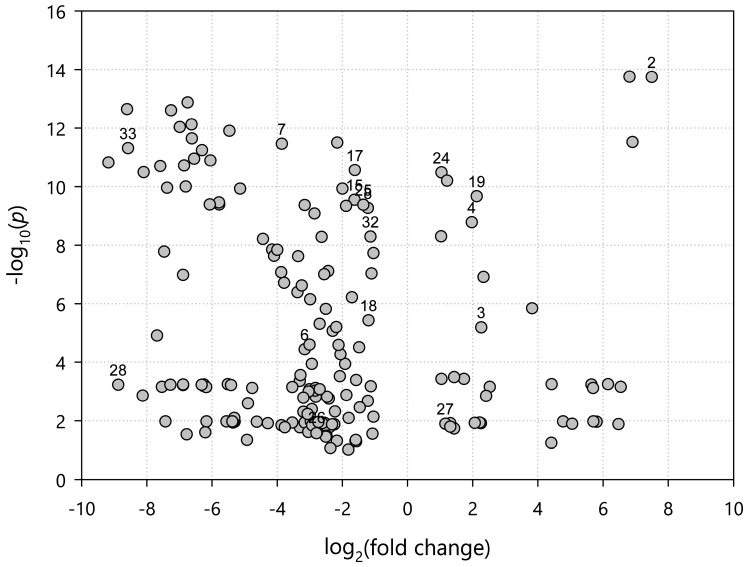
Combined results of the univariate *t-*test, and fold change analyses of *P. officinalis* samples. Numbers indicate compounds isolated and characterized in this study (Figure 1 and Table 1).

Univariate volcano plot analysis, combining *t*-tests and fold change examination, determined several features as linked with spring and autumn stages of *Pulmonaria* life cycle. As shown in Figure 6, the features on the right side of the volcano plot were the most significant for the spring samples, whereas the elements on the left side are connected with the autumn samples. Among the features characteristic for spring samples, a few compounds isolated and characterized in this study were identified. Chief among them is menisdaurin (**2**), which nearly completely disappeared in the autumn samples. Similarly, spring samples could be described by high levels of rutin (**19**), nicotiflorin (**24**) as well as 3-*O*-caffeoyl-threonic acid (**3**). Presence of the latter compound can have some ecological significance, as this type of hydroxycinnamic acid derivative is known as oviposition stimulant for *Papilio* sp. butterflies [86]. On the other hand, high levels of several other compounds, but mainly salvianolic acid H (**33**), actinidioionoside (**7**), three isomers of coumaroylquinic acid (**15**,**16**,**17**), as well as pulmonarioside B (**35**), were linked to the autumn samples. 

**Figure 7:**
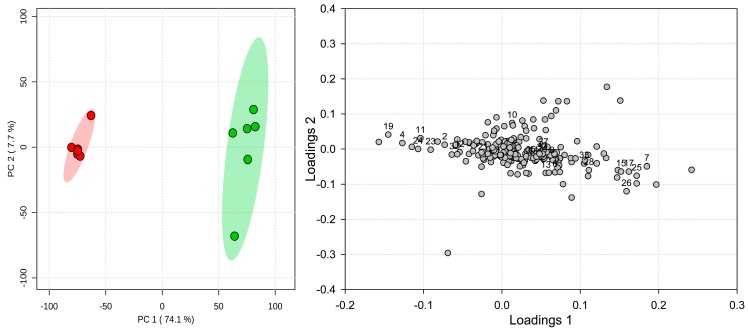
Scores plot (**left**) and loadings (**right**) from principal components analysis of *Pulmonaria officinalis* spring (red circles) and autumn (green circles) samples using untargeted metabolomics approach. Shading indicates 95% confidence intervals.

Unsupervised principal component analysis (PCA) indicated a clear separation of samples according to their phenological stage along the first component, encompassing 74.2% of the dataset variability (Figure 7). Investigation of PCA loadings (Figure 7) revealed that the variables responsible for grouping spring samples were, in large part, virtually the same as these indicated by univariate analyses: rutin (**19**), menisdaurin (**2**), *O*-caffeoyl-l-threonic acids (**3**,**4**), and nicotiflorin (**24**). Additionally, *O*-caffeoyl glyceric acid (**11**) and malonyl glucoside of quercetin (**23**) were also indicated as being characteristic for spring samples. Similarly, autumn samples were again characterized by high amounts of salvianolic acid H, actinidioionoside, and coumaroylquinic acids, but also globoidnan B (**18**) astragalin (**25**), shimobashiric acid C (**26**), and malonyl glucoside of kaempferol (**28**).

**Figure 8:**
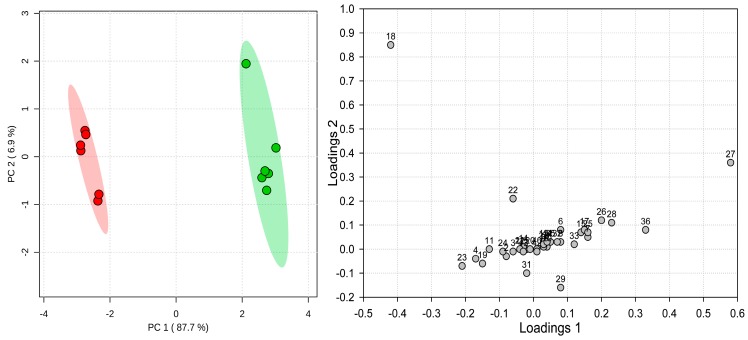
Scores plot (**left**) and loadings (**right**) from the principal components analysis of *Pulmonaria officinalis* spring (red circles) and autumn (green circles) samples, using a targeted metabolomics approach. Shading indicates 95% confidence intervals.

Multivariate analyses carried out on the dataset derived from targeted measurements of selected 39 metabolites produced very similar results (Figure 8). Again, clear separation of the two groups of samples can be observed along the first principal component (87.7% variability). Likewise, loadings still indicated nearly the same set of metabolites as responsible for grouping spring and autumn samples (Figure 8). However, one notable exception is rosmarinic acid (**27**), which in the untargeted analysis, was characteristic for the spring samples. In the targeted study, it conversely appeared to be marker for autumn samples. The reason for this discrepancy underlays a problem with metabolomics based on peak intensities. Both spring and autumn samples were so abundant in rosmarinic acid that its signal during MS analyses was overloaded. Thus, in both cases, registered intensities of the *m*/*z* 359.07 ion had similarly high levels, and even slight variabilities of the signal produces differences that may seem relevant. However, as clearly seen in the volcano plot (Figure 6), where the point representing rosmarinic acid is located in the lower central part, these changes were lesser in the level and have higher *p*-value. A signal overload can be easily corrected using appropriate dilutions; however, high dilutions generate a risk of loss from the analysis of some other less abundant but also essential components of the sample. The trade-off dilution that we applied—10-fold, was unfortunately not sufficient enough to include the rosmarinic acid in the multivariate PCA model in the untargeted analysis. On the other hand, the targeted analysis was based on a series of three dilutions (2, 10, and 50 times), to allow for proper quantitation of the analyzed compounds within the range of the calibration curves. 

**Figure 9:**
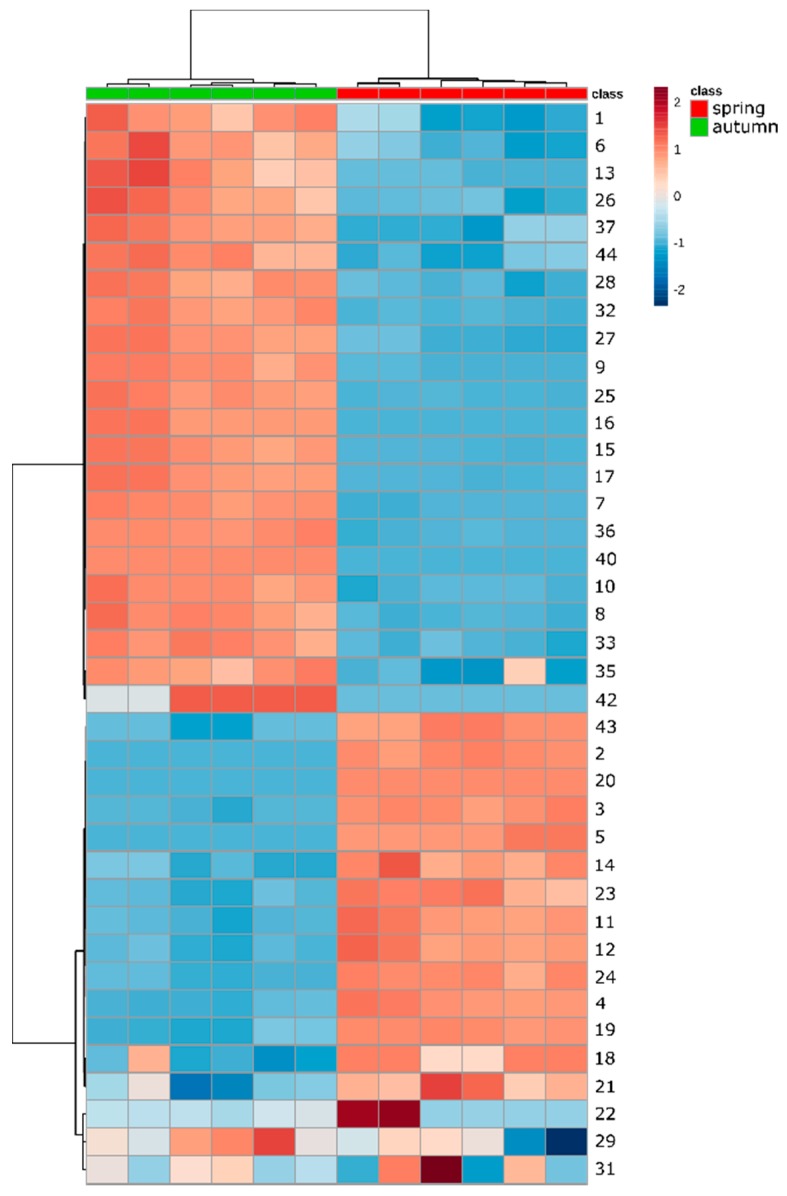
Result of the aggregative hierarchical clustering (Euclidian distance measure, Ward′s clustering algorithm) of *Pulmonaria officinalis* samples and metabolites, shown as a heatmap. Numbers correspond to compounds isolated in this study as shown in Figure 1 and Table 1, heatmap colors represent the relative concentrations in the samples from high (red) to low (blue).

With the results of the targeted analysis, it was clear that phenolic acids and flavonoids accumulation patterns during the life cycle of *Pulmonaria* were quite multifaceted (Table 6, heat map in Figure 9). Flavonol glycosides in other species are known to be at their highest levels during spring, then their concentrations decline, reaching a minimum at the end of the phenological cycle. Such a pattern was observed for unidentified flavonoids in *Rosmarinus officinalis*, although rutin displayed the opposite trend, accumulating in the highest amount at the vegetative stage of the life cycle [81]. Similarly, analyses of flavonoids in *Hypericum perforatum* also showed the accumulation of hyperoside and apigenin glucoside in the pre-flowering and flowering stages, followed by a decline during mature fruiting, and a slight increase in the late vegetative stage. Again, rutin accumulated only during the vegetative stage [82]. The opposite trend for rutin was, however, observed in the case of *Melittis melissophyllum*, where it accumulated in leaves in May, and sharply declined in the same tissues in September [85]. This pattern is mostly the same as observed in the current study for rutin, nicotiflorin, and to a slightly lesser extent, also for malonyl glucoside, as well as for glucosides of quercetin. On the other hand, both malonyl glucoside and a glucoside of kaempferol seemed to accumulate during the vegetative stage. Considering that *P. officinalis* is one of the early-spring plants that flowers before tree-canopy leaves develop, increased levels of quercetin derivatives (**19**,**21**,**23**) are probably a reflection of high light irradiance that up-regulates their biosynthesis [87,88]. On the contrary, higher levels of kaempferol derivatives at the end of the vegetative cycle, when *Pulmonaria* plants usually live under dense tree canopies, may reflect an association of these derivatives with shaded growth conditions [88]. However, flavonoid glycosides with B-ring diols, such as quercetin derivatives, were hypothesized to prevent high-intensity light damage by scavenging reactive oxygen species (ROS). UV-induced ROS generation can also be inhibited by compounds that merely absorb relevant light wavelengths, and phenolic acids appear to be much more suitable for this function than flavonoids [89]. This can be a potential function of several caffeic acid derivatives with high concentrations in spring samples that decline in levels during life cycle progression. In particular, O-caffeoyl threonic and glyceric acids, and globoidnan B may serve UV-B protective functions during the spring development of *Pulmonaria*. Nonetheless, numerous other phenolic acids derivatives, including the main metabolite—rosmarinic acid, appear to accumulate in aerial parts in autumn. An increase in the levels of simple compounds such as caffeic acid (**8**) and danshensu (**1**), can be a result of the turnover and degradation of other derived composite metabolites. However, yunnaneic acid B, a relatively complex molecule, also accumulates nearly 9-fold compared to spring samples. The role of these metabolites at the vegetative stage of *Pulmonaria* life cycle is yet to be explained. 

There is still need to explore the usefulness of many of traditionally used herbs for modern therapy, pharmacy, pharmacology, or medicine. Presented research explores the phytochemical composition of commonly occurring and well known medicinal plant that nowadays is not intensively used for therapeutic purposes. For the first time, specialized metabolites of *Pulmonaria*, especially phenolic compounds, were thoroughly investigated by a combination of NMR, HR-MS, and other spectral techniques, providing full-scale qualitative and quantitative information. The results of this study will contribute to qualitative and quantitative method development and quality control or standardization purposes.

## 3. Materials and Methods 

### 3.1. Chemicals and Reagents

Acetonitrile and methanol, LC-MS grade and HPLC grade respectively, were purchased from Merck (Darmstadt, Germany). MS-grade formic acid was purchased from Sigma Aldrich (Steinheim, Germany). Ultrapure water was prepared using a Milli-Q water purification system (Millipore, Milford, MA, USA).

### 3.2. Plant Material

Dried aerial parts of *P. officinalis* used for compound isolation were purchased from a local herb supplier (Kania, Czestochowa, Poland). The voucher sample (POFF./EXTR/2013/1) has been deposited at the Department of Biochemistry and Crop Quality of the Institute. Quantitative analyses of isolated compounds during the growing season have been carried out for *P. officinalis* L., and rhizomes were donated from The Botanical Garden of Maria Curie-Skłodowska University in Lublin. Rhizomes were planted at the experimental plots of the Institute of Soil Science and Plant Cultivation, Puławy, Poland (N51°24.767′ E021°57.924′). The aerial parts of lungwort were collected from early spring to late autumn at weekly intervals. The meteorological conditions for April and September are presented in Table 7. The voucher samples have also been deposited at the Department of Biochemistry and Crop Quality of the Institute. 

### 3.3. Extraction and Isolation

Aerial parts of *P. officinalis* were finely powdered with an electric grinder and sieved through a 0.5 mm sieve. Plant material was defatted with chloroform in a Soxhlet apparatus. Afterward, it was extracted twice with 80% methanol (*v*/*v*) using ultrasonic bath, at room temperature for 24 h. The obtained extract was filtered through filter paper (Whatmann No.1), concentrated under reduced pressure/vacuum, and freeze-dried. The yield of the extraction was 24.4%. 

The crude methanol extract was then purified in a stepwise manner by different chromatographic methods. First, the extract was applied to a preconditioned RP-C_18_ column (80 × 100 mm, Cosmosil 140C_18_-PREP, 140 µm; Nacalai Tesque, INC., Kyoto, Japan), followed by removal of polar constituents (1% MeOH *v*/*v*), while phenolic-rich fraction was eluted with 50% methanol. Collected fractions were monitored using thin layer chromatography (TLC) techniques. TLC was performed on silica gel plates with a solvent system consisting of MeCN:H_2_O:CHCl_3_:HCOOH (100:10:10:5) as a mobile phase. The plates were visualized under UV light at 254/360 nm, and then sprayed with a methanol/sulfuric acid reagent and heated on a hot plate. The 50% methanol fraction (32.8 g) was further purified by low-pressure chromatography on Sephadex LH-20 (Sigma-Aldrich, Steinheim, Germany) column (48 × 400 mm) and eluted with a gradient of MeOH (5–100%, *v*/*v*). As a result of this separation, 10 fractions (Fr 0–9) were collected. Due to the high similarity of their composition, fractions 4 and 5, as well as 6 and 7, were combined with each other. The fractions were subsequently subjected to a reversed phase column (32 × 300 mm, Cosmosil 40C_18_-PREP, 40 µm; Nacalai Tesque, INC., Kyoto, Japan), which yielded several sub-fractions. The compositions of the fraction and sub-fractions were monitored by LC-MS technique as described below. Individual compounds were further purified by a semi-preparative HPLC chromatographic system. 

It should be noted that rosmarinic acid was a dominant compound of the whole 50% MeOH fraction, constituting 24% of the fraction. Moreover, this compound constituted 23% of Fr. 3, 73% of combined fractions 4 + 5, 63% of fractions 6 + 7, and 45% of fraction 8 respectively. Chromatographic separation of Fr. 0 (4.05 g) yielded compound **22** (7.8 mg). Fr. 1 (11.37 g) yield compounds: **1** (4.2 mg), **2** (393 mg), **7** (4 mg), **36** (98 mg). Fr. 2 (9.53 g), which was divided into 12 subfractions, produced compounds: **3** (7 mg), **4** (5.5 mg), **5** (3.5 mg), **6** (15 mg), **9** (5.5 mg), **10** (4 mg), **11** (5.5 mg), **12** (5.5 mg), **14** (13 mg), **15** (22 mg), **18** (279 mg), **26** (45 mg), **28** (6 mg), **29** (22 mg), **30** (4 mg), **31** (27 mg). Fr. 3 (1.49 g) yielded molecules **23** (9.5 mg), **33** (291 mg) and **34** (3.14 mg). Combined Fr. 4 + 5 (2.09 g) yield compounds **35** (32 mg), and **27** (1289 mg). Fr. 6 + 7 (1.11 g) gave molecules: **8** (5.5 mg), **19** (2.0 mg), **21** (28.7 mg), **32** (14.8 mg), **24** (1.93 mg), **25** (16 mg). Fr. 8 (3.07 g) gave compounds: **37** (27.5 mg), **38** (3 mg), **39** (2.9 mg), **40** (2.69 mg), **41** (1.64 mg), **43** (3.75 mg), and **44** (49.5 mg). Fr. 9 (0.04 g) was not further purified. Tentative identification based on HR-LC-QTOF-MS/MS analysis, fragmentation patterns, and comparison with literature data, as well as its similarity to isolated compounds concerns following compounds: **13** (neochlorogenic acid), **16** (4*-O-p-*coumaroylquinic acid), **17** (5*-O-p-*coumaroylquinic acid), **20** (nicotiflorin isomer), **42** (isosalvianolic acid A isomer), and **45** (lycopic acid C). Some of these compounds were, however, included in quantitative analyzes of spring and autumn *Pulmonaria* extracts (Table 6).

### 3.4. Instruments

#### 3.4.1. Semi-Preparative HPLC

The final purification steps utilized the semi-preparative HPLC Gilson chromatographic system (Gilson Inc., Middleton, WI, USA), equipped with an evaporative light scattering detector (ESLD, Gilson PrepELS II). The drift tube of the ELSD detector was maintained at 65 °C, and the pressure of the nebulizer gas (nitrogen) was 47 psi. Sub-fractions obtained from low-pressure reversed phase liquid chromatography were further purified on a variety of columns: Atlantis T3 Prep OBD (10 × 250 mm, 5 µm, Waters, Milford, MA, USA), COSMOSIL π-NAP (10 × 250 mm, 5 µm, Nacalai Tesque, INC., Kyoto, Japan) or RP-18 Kromasil (10 × 250mm, 5 µm, AkzoNobel, Bohus, Sweden). The conditions of chromatographic separation were individually optimized for each fraction. Separations were carried out in isocratic or in gradient mode, using aqueous acetonitrile or methanol solutions, containing 0.1% formic acid. The mobile phase flow rate was from 3 to 4 mL/min, and the column was held between 35 and 55 °C. The effluent from the HPLC system was diverted through a passive splitter to ELSD with a split ratio of 1:100.

#### 3.4.2. High-Resolution LC-MS and Qualitative Analysis 

For quantitative analyses, harvested plants were freeze-dried, finely powdered, and used for extraction with an automated accelerated solvent extractor, ASE 200 (Dionex, Sunnyvale, CA, USA). One hundred milligrams of each sample was extracted three times with 80% aqueous MeOH (three static cycles, 5 min each), at 1500 psi (10.3 MPa) solvent pressure, 100 °C temperature of extraction cells, flush 150%. Obtained crude extracts were evaporated to dryness, dissolved in 1 mL of Milli-Q water (Millipore Corp., Billerica, MA, USA), containing 25 µg/mL of digoxin (internal standard, IS) and purified by solid phase extraction (SPE) using Oasis HLB columns (500 mg, Waters Corp., Milford, MA, USA). The extracts were loaded on preconditioned cartridges, which were washed with 0.5% MeOH to remove unbound material, and then with 85% MeOH to elute specialized metabolites. These fractions were evaporated and dissolved in 1 mL of 85% MeOH acidified with 0.1% HCOOH. All analyses were performed in triplicate and samples were stored in a freezer at −20 °C before analysis. Before spectrometric analyses samples were centrifuged (15 min, 23,000× g) and appropriately diluted with distilled water.

High-resolution LC-MS analyses, e.g.,: exact masses, MS/MS fragmentation patterns, molecular formulae, as well as quantitative determinations, were performed on a Thermo Scientific Ultimate 3000 RS chromatographic system coupled/hyphenated with a Bruker Impact II HD (Bruker, Billerica, MA, USA) quadrupole time-of-flight (Q-TOF) mass spectrometer.

Chromatographic separations were carried out on a Waters CORTECS T3 column (2.1 × 150 mm, 2.7 µm, Milford, MA USA) equipped with pre-column. The mobile phase A was 0.1% (*v*/*v*) formic acid, and the mobile phase B was acetonitrile containing 0.1% (*v*/*v*) of formic acid. A concave-shaped gradient (Dionex gradient curve nr. 6) from 5% to 60% of phase B over 25 min was used for separation. The flow rate was 0.6 mL/min, and the column was held at 35 °C. Between the injections column was equilibrated with 10 volumes of 5% phase B. Injection volume was 5 µL.

Analyses were carried out in both positive and negative ion mode with electrospray ionization. Measurements in the negative ion mode were accompanied with detection based on charged aerosol (CAD). A flow splitter was used to divert the column effluent in 1:3 proportion between Q-TOF MS and charged aerosol detector connected in parallel. CAD acquisition frequency was 10 Hz. Analyses in the positive ion mode employed additional UV absorbance detection in the 200–600 nm wavelength range with 5 nm bandwidth and 10 Hz acquisition frequency.

Linear (centroid) mass spectra were acquired over a mass range from *m*/*z* 50 to *m*/*z* 2000 with the following parameters of mass spectrometer: positive ion capillary voltage 4.5 kV; negative ion capillary voltage 3.0 kV, dry gas flow 6 L/min; dry gas temperature 200 °C; nebulizer pressure 0.7 bar; collision RF 700.0 V; transfer time 90 μs; prepulse storage 7.0 μs. Two precursor ions with intensities over 2000 counts were fragmented in each scan. The collision energy and the ion isolation width were set automatically depending on the *m/z* of the fragmented ion, in the range of 5 to 100 eV and from 3 to 8 mass units, respectively. The acquired data were calibrated internally with sodium formate introduced to the ion source via a 20 µL loop at the beginning of each separation.

Calibration curves in the range from 0.05 to 50 µg/mL were prepared from the 1 mg/mL methanolic stock solutions of investigated compounds and analyzed in the conditions specified above. Extracted ion chromatograms were made from full scan data with a 0.005 Da width. Smoothing using the Savitzky-Golay algorithm (window width 5 points, one iteration) was applied and peaks corresponding to deprotonated molecules (or, in the case of compounds **2**,**7** and the internal standard, deprotonated formic acid adducts) were integrated. Ratios between the analyte peak area and the IS peak area were used for calculations. Details of calibrations are shown in Table S1 (see Supplementary Materials). Data processing was performed using Bruker DataAnalysis 4.4 SR1 software. All quantitative results were calculated per dry weight (DW).

#### 3.4.3. Untargeted Metabolomics Analyses.

For untargeted metabolomic analyses, data obtained from LC-QTOF-MS/MS runs were processed using Find Molecular Features function of Bruker DataAnalysis ver. 4.4 SR1 software. The following parameters were used: a signal-to-noise threshold of 9, correlation coefficient threshold of 0.7, and the minimum compound length of seven spectra. For each detected compound, in addition to the deprotonated ion, all typical adduct and composite ions were grouped into a single feature. Next, features from the entire dataset were subjected to advanced bucket generation with Bruker ProfileAnalysis ver. 2.3 software, using retention time range 1.2–22.0 min and *m*/*z* range 50–1500. Time alignment was also applied. Resulting data matrix, consisting of intensity values for 283 peaks indexed by feature parameters (*m*/*z*, retention time) and sample name (12 samples), was exported and uploaded to MetaboAnalyst [90]. Data were normalized using internal standard (by the peak intensity of a feature containing *m*/*z* 825.428, corresponding to [M + HCOOH-H]^−^ ion of digoxin at RT 17.48 min). At this stage of data processing, standard *t*-test and fold-change analyses were carried out to provide a preliminary overview of features potentially characteristic for the two phenological stages under study. Next, all missing intensity values and intensities that were equal to zero in the data matrix were replaced by the half of the minimum positive value that was found within the data, and multivariate PCA was applied to investigate systematic variation in the data matrix and to identify potential groups in an unsupervised manner.

The bias related to instrumental drift was minimized by randomization of the sample list injection order. It was achieved using an in-house-developed VBA (Visual Basic for Applications) script in Microsoft Excel.

The performance of the LC-MS and the data processing systems were monitored with two types of quality control (QC) samples. Class-specific QC samples (eight in total, four for each group) were prepared by mixing 10 µL aliquots of each sample within the group and diluting them 10 times, just like normal samples. Class QC samples were analyzed after every six analyses of normal samples. During data processing, features detected in less than 75% of class-specific QC samples were removed from the dataset. The frequency distribution of the relative standard deviation for the peak intensities and peak numbers in the QC samples are given in Figure S107 (see Supplementary Materials). The general QC sample consisted of a 8 µg/mL mixture of each of the 38 compounds from *P. officinalis* that had been isolated in the course of this study, and 25 µg/mL of the internal standard. This QC sample was used to monitor the quality and stability of the data acquisition and was analyzed in replicate after each block of 20 analyses.

#### 3.4.4. NMR Spectroscopy

The 1D and 2D NMR spectra (^1^H, ^13^C DEPTQ, ^1^H–^13^C HSQC, ^1^H–^13^C H2BC, ^1^H–^13^C HMBC, ^1^H–^13^C F2-coupled perfect-CLIP HSQC, ^1^H–^13^C HSQC-TOCSY, ^1^H–^1^H COSY DQF, ^1^H–^1^H TOCSY, ^1^H–^1^H NOESY, ^1^H–^1^H TROESY, 1D–TOCSY, 1D-TROESY, CSSF-1D-NOESY, and CSSF-1D-TOCSY [91] were performed using an Avance III HD 500 MHz spectrometer (Bruker BioSpin, Rheinstetten, Germany), in MeOH-*d*_4_ or MeOH-*d*_4_ with 0.1% TFA. The only exception was shimobashiric acid C, where DMSO-*d*_6_ was applied.

#### 3.4.5. Optical Rotation [α]

The optical rotation of isolated compounds was measured on an automatic polarimeter (P-2000, JASCO, Tokyo, Japan).

#### 3.4.6. Circular Dichroism Spectroscopy 

Circular dichroism measurements of optically active compounds were carried out on a J-815 circular dichroism spectrometer (JASCO, Tokyo, Japan), using a quartz cell of 1 cm path length. Spectra of analyzed molecules were recorded at 21 °C from 220 to 400 nm at a 0.2 nm resolution, with a scan rate of 50 nm/min. Raw data were smoothed using the Savitzky-Golay method with a window of 11 data points. CD spectra are presented in the Supplementary Materials. 

#### 3.4.7. Characteristic Data of Lungwort Compounds

HR-QTOF-MS/MS data in negative ion mode for all compounds has been shown in Table 1.

Danshensu (3,4-dihydroxyphenyl) lactic acid (**1**); white amorphous powder; UV (PDA, MeCN/H_2_O) λ_max_ (nm) 280; HR-QTOF-MS (neg.) *m*/*z* 197.0455 [M − H]^−^ (calc. for C_9_H_10_O_5_ 197.0455).

Menisdaurin (**2**); light brownish amorphous powder; UV (PDA, MeCN/H_2_O) λ_max_ (nm) 260; HR-QTOF-MS (neg.) *m*/*z* 312.1086 [M − H]^−^ (calc. for C_14_H_19_NO_7_ 312.1089).

3-*O*-(*E*)-Caffeoyl-threonic acid (**3**); white amorphous powder; UV (PDA, MeCN/H_2_O) λ_max_ (nm) 325; ${\left[\alpha \right]}_{D}^{21}$ = +67.53 (c 0.73, MeOH); CD (5 × 10^−5^ M, MeOH): [*Θ*]_228_ − 3330, [*Θ*]_252_ − 32, [*Θ*]_259_ − 170_,_ [*Θ*]_297_ + 2385, [*Θ*]_306_ + 2250, [*Θ*]_315_ + 1877, [*Θ*]_322_ + 2185, [*Θ*]_325_ + 2034, [*Θ*]_333_ + 2338, [*Θ*]_342_ + 1962, [*Θ*]_347_ + 2091, [*Θ*]_372_ + 284, [*Θ*]_377_ + 340; HR-QTOF-MS (neg.) *m*/*z* 297.0619 [M − H]^−^ (calc. for C_13_H_14_O_8_ 297.0616). ^1^H and ^13^C-NMR spectroscopic data (Table 2).

2-*O*-(*E*)-Caffeoyl-l-threonic acid (**4**); white amorphous powder; UV (PDA, MeCN/H_2_O) λ_max_ (nm) 325; ${\left[\alpha \right]}_{D}^{21}$ = −2.52 (c 0.52, MeOH); CD (5 × 10^−5^ M, MeOH) [*Θ*]_237_ + 1647, [*Θ*]_338_ − 1574; HR-QTOF-MS (neg.) *m*/*z* 297.0611 [M − H]^−^ (calc. for C_13_H_14_O_8_ 297.0616).

Lycoperodine-1 (**5**); white amorphous powder; UV (PDA, MeCN/H_2_O) λ_max_ (nm) 275; HR-QTOF-MS (neg.) *m*/*z* 215.0825 [M − H]^−^ (calc. for C_12_H_11_N_2_O_2_ 215.0826).

Chlorogenic acid (**6**); white amorphous powder; UV (PDA, MeCN/H_2_O) λ_max_ (nm) 325; HR-QTOF-MS (neg.) *m*/*z* 353.0882 [M − H]^−^ (calc. for C_16_H_18_O_9_ 353.0878).

Actinidioionoside (**7**); white amorphous powder; UV (PDA, MeCN/H_2_O) λ_max_ (nm) 325; HR-QTOF-MS (neg.) *m*/*z* 405.2126 [M − H]^−^ (calc. for C_19_H_34_O_9_ 405.2130).

Caffeic acid (**8**); white amorphous powder; UV (PDA, MeCN/H_2_O) λ_max_ (nm) 325; HR-QTOF-MS (neg.) *m*/*z* 179.0262 [M − H]^−^ (calc. for C_9_H_8_O_4_ 179.0349).

Cryptochlorogenic acid (**9**); white amorphous powder; UV (PDA, MeCN/H_2_O) λ_max_ (nm) 325; HR-QTOF-MS (neg.) *m*/*z* 353.0882 [M − H]^−^ (calc. for C_16_H_18_O_9_ 353.0878).

3′-*O*-(*E*)-Feruloyl-*α*-sorbopyranosyl-(2′→1)-*α*-glucopyranoside (**10**); white amorphous powder; UV (PDA, MeCN/H_2_O) λ_max_ (nm) 325; ${\left[\alpha \right]}_{D}^{21}$ = +2.79 (c 0.19, MeOH); HR-QTOF-MS (neg.) *m*/*z* 517.1572 [M − H]^−^ (calc. for C_22_H_30_O_14_ 517.1563). ^1^H and ^13^C-NMR spectroscopic data (Table 5).

2-*O*-(*E*)-Caffeoyl-d-glyceric acid (**11**); white amorphous powder; UV (PDA, MeCN/H_2_O) λ_max_ (nm) 325; ${\left[\alpha \right]}_{D}^{21}$ = -44.18 (c 0.55, MeOH); CD (5 × 10^−5^ M, MeOH) [*Θ*]_265_ − 144, [*Θ*]_317_ − 5806, [*Θ*]_380_ + 296; HR-QTOF-MS (neg.) *m*/*z* 267.0508 [M − H]^−^ (calc. for C_12_H_12_O_7_ 267.0510).

4-*O*-(*E*)-Caffeoyl-l-threonic acid (**12**); white amorphous powder; UV (PDA, MeCN/H_2_O) λ_max_ (nm) 215, 325; ${\left[\alpha \right]}_{D}^{21}$ = −16.41 (c 0.15, MeOH); CD (5 × 10^−5^ M, MeOH) [*Θ*]_261_−169, [*Θ*]_283_ − 593, [*Θ*]_300_ − 509, [*Θ*]_325_ − 1349, [*Θ*]_384_ + 80, [*Θ*]_380_ + 296; HR-QTOF-MS (neg.) *m*/*z* 297.0616 [M − H]^−^ (calc. for C_13_H_14_O_8_ 297.0616).

Neochlorogenic acid (**13**); tentative identification; UV (PDA, MeCN/H_2_O) λ_max_ (nm) 325; HR-QTOF-MS (neg.) *m*/*z* 353.0878 [M − H]^−^ (calc. for C_16_H_18_O_9_ 353.0878).

3-*O*-(*E*)-Caffeoyl- glyceric acid (**14**); white amorphous powder; UV (PDA, MeCN/H_2_O) λ_max_ (nm) 215, 325; ${\left[\alpha \right]}_{D}^{21}$ = +7.49 (c 0.21, MeOH); CD (5 × 10^−5^ M, MeOH) [*Θ*]_231_ − 1125, [*Θ*]_253_ − 207, [*Θ*]_273_ − 277, [*Θ*]_293_ + 208, [*Θ*]_305_ + 429, [*Θ*]_324_ + 1234, [*Θ*]_343_ + 76, [*Θ*]_358_ − 204, [*Θ*]_373_ − 286; HR-QTOF-MS (neg.) *m*/*z* 267.0509 [M − H]^−^ (calc. for C_12_H_12_O_7_ 267.0510).^1^H and ^13^C-NMR spectroscopic data (Table 2). 

3-*O*-*p*-Coumaroylquinic acid (**15**); white amorphous powder; UV (PDA, MeCN/H_2_O) λ_max_ (nm) 225, 310; ${\left[\alpha \right]}_{D}^{21}$ = −28.45 (c 0.19, MeOH); HR-QTOF-MS (neg.) *m*/*z* 337.0924 [M − H]^−^ (calc. for C_16_H_18_O_8_ 337.0929).

4-*O*-*p*-Coumaroylquinic acid (**16**); tentative identification; UV (PDA, MeCN/H_2_O) λ_max_ (nm) 225, 310; HR-QTOF-MS (neg.) *m*/*z* 337.0926 [M − H]^−^ (calc. for C_16_H_18_O_8_ 337.0929).

5-*O*-*p*-Coumaroylquinic acid (**17**); tentative identification; UV (PDA, MeCN/H_2_O) λ_max_ (nm) 225, 310; HR-QTOF-MS (neg.) *m*/*z* 337.0924 [M − H]^−^ (calc. for C_16_H_18_O_8_ 337.0929).

Globoidnan B (**18**); white amorphous powder; UV (PDA, MeCN/H_2_O) λ_max_ (nm) 255, 345; ${\left[\alpha \right]}_{D}^{21}$ = +114.9 (c 0.25, MeOH); CD (5 × 10^−5^ M, MeOH) [*Θ*]_254_ − 14186, [*Θ*]_292_ + 3337, [*Θ*]_313_ − 4443, [Θ]_351_ + 12263; HR-QTOF-MS (neg.) *m*/*z* 537.1034 [M − H]^−^ (calc. for C_27_H_21_O_12_ 537.1039). ^1^H and ^13^C-NMR spectroscopic data (Table 4).

Rutin (**19**) yellow amorphous powder; UV (PDA, MeCN/H_2_O) λ_max_ (nm) 255, 355; HR-QTOF-MS (neg.) *m*/*z* 609.1464 [M − H]^−^ (calc. for C_27_H_30_O_16_ 609.1461).

Nicotiflorin isomer (**20**); tentative identification; UV (PDA, MeCN/H_2_O) λ_max_ (nm) 345; HR-QTOF-MS (neg.) *m*/*z* 593.1501 [M − H]^−^ (calc. for C_27_H_30_O_15_ 593.1512).

Quercetin 3-*O*-*β*-glucoside (**21**); yellow amorphous powder; UV (PDA, MeCN/H_2_O) λ_max_ (nm) 255, 355; HR-QTOF-MS (neg.) *m*/*z* 463.0892 [M − H]^−^ (calc. for C_21_H_20_O_12_ 463.0882).

Yunnaneic acid E (**22**); white amorphous powder; UV (PDA, MeCN/H_2_O) λ_max_ (nm) 265; ${\left[\alpha \right]}_{D}^{21}$ = +19.43 (c 0.43, MeOH); HR-QTOF-MS (neg.) *m*/*z* 571.1092 [M − H]^−^ (calc. for C_27_H_23_O_14_ 571.1093).

Quercetin 3-*O*-(6″-*O*-malonyl)-*β*-glucoside (**23**); yellow amorphous powder; UV (PDA, MeCN/H_2_O) λ_max_ (nm) 255, 355; HR-QTOF-MS (neg.) *m*/*z* 549.0876 [M − H]^−^ (calc. for C_24_H_22_O_15_ 549.0886).

Nicotiflorin (**24**); yellow amorphous powder; UV (PDA, MeCN/H_2_O) λ_max_ (nm) 265, 345; HR-QTOF-MS (neg.) *m*/*z* 593.1503 [M − H]^−^ (calc. for C_27_H_30_O_15_ 593.1512).

Astragalin (**25**); yellow amorphous powder; UV (PDA, MeCN/H_2_O) λ_max_ (nm) 265, 345; HR-QTOF-MS (neg.) *m*/*z* 447.0940 [M − H]^−^ (calc. for C_21_H_20_O_11_ 447.0933).

Shimobashiric acid C (**26**); light cream amorphous powder; UV (PDA, MeCN/H_2_O) λ_max_ (nm) 285; HR-QTOF-MS (neg.) *m*/*z* 719.1607 [M − H]^−^ (calc. for C_36_H_32_O_16_ 719.1618).

Rosmarinic acid (**27**); light yellowish amorphous powder; UV (PDA, MeCN/H_2_O) λ_max_ (nm) 220, 330; CD (1 × 10^−4^ M, MeOH) [*Θ*]_232_ − 9423, [*Θ*]_254_ − 1004, [*Θ*]_275_ − 3233, [*Θ*]_302_ + 7406, [*Θ*]_313_ + 6634, [*Θ*]_326_ + 7255; HR-QTOF-MS (neg.) *m*/*z* 359.0773 [M − H]^−^ (calc. for C_18_H_16_O_8_ 359.0772).

Kaempferol 3-*O*-(6″-*O*-malonyl)-*β*-glucoside (**28**); yellow amorphous powder; UV (PDA, MeCN/H_2_O) λ_max_ (nm) 265, 345; HR-QTOF-MS (neg.) *m*/*z* 533.0940 [M − H]^−^ (calc. for C_24_H_22_O_14_ 533.0937).

Monardic acid A (**29**); white amorphous powder; UV (PDA, MeCN/H_2_O) λ_max_ (nm) 310; ${\left[\alpha \right]}_{D}^{21}$ = −120.93 (c 0.78, MeOH); CD (5 × 10^−5^ M, MeOH) [*Θ*]_253_ − 70887, [*Θ*]_290_ + 6650, [*Θ*]_332_ − 7130, [*Θ*]_386_ + 290; HR-QTOF-MS (neg.) *m*/*z* 537.1035 [M − H]^−^ (calc. for C_27_H_22_O_12_ 537.1039).

Yunnaneic acid E-1, (R)-2-((3-(3-(carboxycarbonyl)-3′,4′-dihydroxy-[1,1′-biphenyl]-4-yl) propanoyl)oxy)-3-(3,4-dihydroxyphenyl)propanoic acid (**30**); white amorphous powder; UV (PDA, MeCN/H_2_O) λ_max_ (nm) 265; HR-QTOF-MS (neg.) *m*/*z* 509.1094 [M − H]^−^ (calc. for C_26_H_22_O_11_ 509.1089). ^1^H and ^13^C-NMR spectroscopic data (Table 5).

Lithospermic acid A (**31**); white amorphous powder; UV (PDA, MeCN/H_2_O) λ_max_ (nm) 310; ${\left[\alpha \right]}_{D}^{21}$ = +154.88 (c 0.90, MeOH); CD (5 × 10^−5^ M, MeOH) [*Θ*]_253_ + 28451, [*Θ*]_281_ − 3444, [*Θ*]_330_ + 8800; HR-QTOF-MS (neg.) *m*/*z* 537.1054 [M − H]^−^ (calc. for C_27_H_22_O_12_ 537.1039).

Pulmonarioside A (**32**); white amorphous powder; UV (PDA, MeCN/H_2_O) λ_max_ (nm) 325; ${\left[\alpha \right]}_{D}^{21}$ = −73.85 (c 1.44, MeOH); CD (5 × 10^−5^ M, MeOH) [*Θ*]_237_ + 16506, [*Θ*]_246_ + 15155, [*Θ*]_253_ + 15786, [*Θ*]_247_ − 4592, [*Θ*]_294_ + 15809, [*Θ*]_341_ − 47198; HR-QTOF-MS (neg.) *m*/*z* 999.2766 [M − H]^−^ (calc. for C_47_H_52_O_24_ 999.2776). ^1^H and ^13^C-NMR spectroscopic data (Table 3).

Salvianolic acid H, (3′-*O*-(8″-Z-caffeoyl) rosmarinic acid) (**33**); white amorphous powder; UV (PDA, MeCN/H_2_O) λ_max_ (nm) 325; ${\left[\alpha \right]}_{D}^{21}$ = +30.07 (c 1.13, MeOH); HR-QTOF-MS (neg.) *m*/*z* 537.1034 [M − H]^−^ (calc. for C_27_H_22_O_12_ 537.1039).

Lithospermic acid B (**34**); white amorphous powder; UV (PDA, MeCN/H_2_O) λ_max_ (nm) 250; 288; 330; ${\left[\alpha \right]}_{D}^{21}$ = +82.18 (c 0.10, MeOH); CD (5 × 10^−5^ M, MeOH) [*Θ*]_235_ + 14042, [*Θ*]_255_ + 25042, [*Θ*]_280_ − 2869; [*Θ*]_303_ + 9014, [*Θ*]_334_ + 11379; HR-QTOF-MS (neg.) *m*/*z* 717.1444 [M − H]^−^ (calc. for C_36_H_30_O_16_ 717.1461).

Pulmonarioside B (**35**); white amorphous powder; UV (PDA, MeCN/H_2_O) λ_max_ (nm) 325; ${\left[\alpha \right]}_{D}^{21}$ = −68.43 (c 1.55, MeOH); CD (5 × 10^−5^ M, MeOH) [*Θ*]_253_ + 32447, [*Θ*]_274_ − 4384, [*Θ*]_296_ + 20685, [*Θ*]_340_ − 56660; HR-QTOF-MS (neg.) *m*/*z* 1013.2946 [M − H]^−^ (calc. for C_48_H_54_O_24_ 1013.2932). ^1^H and ^13^C-NMR spectroscopic data (Table 3).

Yunnaneic acid B (**36**); white amorphous powder; UV (PDA, MeCN/H_2_O) λ_max_ (nm) 280; ${\left[\alpha \right]}_{D}^{21}$ = +92.69 (c 1.22, MeOH); CD (2.5 × 10^−5^ M, MeOH) [*Θ*]_231_ − 13436, [*Θ*]_252_ − 9737, [*Θ*]_269_ − 11309, [*Θ*]_300_ + 41370; HR-QTOF-MS (neg.) *m*/*z* 1093.2255 [M − H]^−^ (calc. for C_54_H_46_O_25_ 1093.2255).

Globoidnan A (**37**); white amorphous powder; UV (PDA, MeCN/H_2_O) λ_max_ (nm) 260, 320; ${\left[\alpha \right]}_{D}^{21}$ = +77.63 (c 0.42, MeOH); HR-QTOF-MS (neg.) *m*/*z* 491.0979 [M − H]^−^ (calc. for C_26_H_20_O_10_ 491.0984).

Pulmitric acid A (**38**); white amorphous powder; UV (PDA, MeCN/H_2_O) λ_max_ (nm) 325; ${\left[\alpha \right]}_{D}^{21}$ = −116.91 (c 0.24, MeOH); CD (5 × 10^−5^ M, MeOH) [*Θ*]_246_ + 10304, [*Θ*]_284_ − 24134, [*Θ*]_324_ + 26434; HR-QTOF-MS (neg.) *m*/*z* 551.1190 [M − H]^−^ (calc. for C_28_H_24_O_12_ 551.1195).^1^H and ^13^C-NMR spectroscopic data (Table 2).

Pulmitric acid B (**39**); white amorphous powder; UV (PDA, MeCN/H_2_O) λ_max_ (nm) 320; ${\left[\alpha \right]}_{D}^{21}$ = −5.15 (c 0.31, MeOH); HR-QTOF-MS (neg.) *m*/*z* 535.0885 [M − H]^−^ (calc. for C_27_H_20_O_12_ 535.0882); ^1^H and ^13^C-NMR spectroscopic data (Table 2).

Isosalvianolic acid A (**40**); white amorphous powder; UV (PDA, MeCN/H_2_O) λ_max_ (nm) 320; ${\left[\alpha \right]}_{D}^{21}$ = +39.74 (c 0.18, MeOH); CD (5 × 10^−5^ M , MeOH) [*Θ*]_230_ − 7505, [*Θ*]_255_ + 5772, [*Θ*]_276_ − 1307, [*Θ*]_305_ + 15195; HR-QTOF-MS (neg.) *m*/*z* 493.1134 [M − H]^−^ (calc. for C_26_H_22_O_10_ 493.1140); ^1^H and ^13^C-NMR spectroscopic data (Table 3). 

Isosalvianolic acid A-1 (**41**); white amorphous powder; UV (PDA, MeCN/H_2_O) λ_max_ (nm) 320; ${\left[\alpha \right]}_{D}^{21}$ = +18.49 (c 0.08, MeOH); CD (5 × 10^−5^ M, MeOH) [*Θ*]_248_ − 6367, [*Θ*]_305_ + 3958; HR-QTOF-MS (neg.) *m*/*z* 493.1134 [M − H]^−^ (calc. for C_26_H_22_O_10_ 493.1140); ^1^H and ^13^C-NMR spectroscopic data (Table 3). 

Isosalvianolic acid A isomer (**42**); tentative identification; UV (PDA, MeCN/H_2_O) λ_max_ (nm) 320; HR-QTOF-MS (neg.) *m*/*z* 493.1133 [M − H]^−^ (calc. for C_26_H_22_O_10_ 493.1081).

Rosmarinic acid methyl ester (**43**); white amorphous powder; UV (PDA, MeCN/H_2_O) λ_max_ (nm) 330; ${\left[\alpha \right]}_{D}^{21}$ = +26.55 (c 0.25, MeOH); HR-QTOF-MS (neg.) *m*/*z* 373.0932 [M − H]^−^ (calc. for C_19_H_18_O_8_ 373.0929). 

Salvianolic acid H-9″-methylester (3′-O-(8″-Z-caffeoyl)rosmarinic acid-9″-methylester) (**44**); white amorphous powder; UV (PDA, MeCN/H_2_O) λ_max_ (nm) 325; HR-QTOF-MS (neg.) *m*/*z* 551.1189 [M − H]^−^ (calc. for C_28_H_24_O_12_ 551.1195). 

Lycopic acid C (**45**) tentative identification; UV (PDA, MeCN/H_2_O) λ_max_ (nm) 220; HR-QTOF-MS (neg.) *m*/*z* 519.0926 [M − H]^−^ (calc. for C_27_H_19_O_11_ 519.0933).

## 4. Conclusions

To the best of our knowledge, this is the first comprehensive study of specialized metabolites in the aerial parts of *Pulmonaria officinalis*. The presented research may provide insights for the potential applications of lungwort as a dietary supplement or a nutraceutical, and may it also contribute to the broader application of *Pulmonariae Herba*. Extracts of *P. officinalis* may serve as a prominent supply of rosmarinic acid and related compounds, as well as a source of several others metabolites. Among the 45 identified metabolites, we found many compounds with well-established therapeutic properties, although none of them alone can be directly associated with the ethnomedicinal use of lungwort. Our results also show progressive changes in the phytochemical composition of *P. officinalis* during the phenological cycle, presumably reflecting both changes in the physiological state of plants, as well as varying intensity of different abiotic factors.

## Figures and Tables

**Table 1:** Compounds identified in *P. officinalis* 50% MeOH fraction using UHPLC-QTOF-MS/MS.

No	Compound Name	RT (min)	Formula	Error (ppm)	mσ	Observed [M − H]^−^	Major Fragments (%)
1	Danshensu	2.9	C_9_H_10_O_5_	0.3	1	197.0455	179.0350 (46), 135.0445 (27), 123.0456 (23)
2	Menisdaurin	3.7	C_14_H_19_NO_7_	1	7.6	312.1086	132.0378 (100), 294.0830 (4)
3	3-*O*-(*E*)-caffeoyl-threonic acid	4.5	C_13_H_14_O_8_	−1.2	2	297.0619	135.0293 (100), 179.0361 (21), 161.0263 (6)
4	2-*O*-(*E*)-caffeoyl-l-threonic acid	5	C_13_H_14_O_8_	1.6	4.4	297.0611	135.0293 (100), 179.0346 (17), 161.0245 (11)
5	Lycoperodine-1	5.3	C_12_H_12_N_2_O_2_	0.5	2.3	215.0825	171.0926 (28), 142.0655 (5), 116.0509 (6)
6	Chlorogenic acid	5.7	C_16_H_18_O_9_	−1	5.7	353.0882	191.0567 (100)
7	Actinidioionoside	5.9	C_19_H_34_O_9_	1	4.2	405.2126	225.1494 (10); 179.0560 (10); 167.1073 (11)
8	Caffeic acid	6	C_9_H_8_O_4_	−1.6	1.8	179.0262	135.0372 (95)
9	Cryptochlorogenic acid	6.2	C_16_H_18_O_9_	−1.1	8.9	353.0882	191.0567 (100), 179.0355 (88), 173.0459 (83)
10	3′−*O*-(*E*)-feruloyl-α-sorbopyranosyl-(2′→1)-α-glucopyranoside	6.3	C_22_H_30_O_14_	−1.7	7.6	517.1572	341.1105 (24); 175.0407 (100); 160.0172 (57)
11	2-*O*-(*E*)-caffeoyl-d-glyceric acid	6.8	C_12_H_12_O_7_	1	1.4	267.0508	161.0242 (100), 133.0288 (14), 179.0356(11)
12	4-*O*-(*E*)-caffeoyl-l-threonic acid	6.9	C_13_H_14_O_8_	−0.1	8.3	297.0616	135.0293 (100), 179.0355 (44), 161.0237 (9)
13	Neochlorogenic acid	7.0	C_16_H_18_O_9_	0	12.1	353.0882	191.0567 (100)
14	3-*O*-(*E*)-caffeoyl-glyceric acid	7.1	C_12_H_12_O_7_	0.6	7.1	267.0509	179.0352 (24); 161.0244 (100); 135.0446 (21)
15	3-*O*-p-coumaroylquinic acid	7.2	C_16_H_18_O_8_	1.4	10.8	337.0924	191.0560 (100); 163.0398 (5)
16	4-*O*-p-coumaroylquinic acid	7.4	C_16_H_18_O_8_	0.8	19.4	337.0926	191.0552 (16); 173.0455 (100); 163.0410 (20)
17	5-*O*-p-coumaroylquinic acid	8.4	C_16_H_18_O_8_	1.4	17.7	337.0924	191.0561 (100)
18	Globoidnan B	9.7	C_27_H_22_O_12_	−1.3	8.3	537.1046	493.1135 (24); 339.0503 (100); 295.0604 (58)
19	Rutin	10.2	C_27_H_30_O_16_	−0.5	10.9	609.1464	300.0277 (68), 271.0249 (100)
20	Nicotiflorin isomer	10.4	C_27_H_30_O_15_	1.8	8	593.1501	284.0320 (83); 255.0295 (100)
21	Quercetin 3-*O*-*β*-glucoside	10.5	C_21_H_20_O_12_	−2.1	8.4	463.0892	300.0284 (100), 271.0256 (100)
22	Yunnaneic acid E	10.9	C_27_H_24_O_14_	0.2	4.9	571.1092	527.1195 (23), 285.0766 (100), 241.0867 (81)
23	Quercetin 3-*O*-(6″-*O*-malonyl)-*β*-glucoside	11.2	C_24_H_22_O_15_	1.7	5.6	549.0876	505.0976 (70), 300.0273 (88)
24	Nicotiflorin	11.4	C_27_H_30_O_15_	1.5	13.1	593.1503	284.0317 (65), 255.0290 (88)
25	Astragalin	11.7	C_21_H_20_O_11_	−1.6	5.6	447.094	284.0328 (42), 255.0301 (93)
26	Shimobashiric acid C	11.9	C_36_H_32_O_16_	1.5	21.6	719.1607	359.0766 (100), 161.0239 (11), 539.1191 (6)
27	Rosmarinic acid	12.4	C_18_H_16_O_8_	−0.2	6.4	359.0773	161.0247 (100), 197.0455 (87), 179.0345 (33)
28	Kaempferol 3-*O*-(6″-*O*-malonyl)-*β*-glucoside	12.6	C_24_H_22_O_14_	−0.5	8.1	533.094	489.1044 (54), 284.0328 (89)
29	Monardic acid A	12.7	C_27_H_22_O_12_	0.6	15	537.1035	493.1128 (4); 295.0628 (100); 185.0240 (25)
30	Yunnaneic acid E-1	12.9	C_26_H_22_O_11_	−1	9	509.1094	329.0672 (40); 285.0768 (100); 135.0445 (38)
31	Lithospermic acid A	13	C_27_H_22_O_12_	−2.9	5.6	537.1054	493.1131 (6); 295.0601 (100); 185.0240 (25)
32	Pulmonarioside A	13.3	C_47_H_52_O_24_	1	15.9	999.2766	853.2179 (100), 809.2258 (16), 485.1282 (37)
33	Salvianolic acid H	13.5	C_27_H_22_O_12_	0.9	17	537.1034	493.1123 (22); 359.0763 (69); 295.0605 (100)
34	Lithospermic acid B	13.7	C_36_H_30_O_16_	2.4	9.9	717.1444	519.0915 (63); 321.0392 (100)
35	Pulmonarioside B	13.9	C_48_H_54_O_24_	−1.3	15.2	1013.2946	867.2370 (89); 823.247 (98); 499.1469 (53)
36	Yunnaneic acid B	14	C_54_H_46_O_25_	0	62.2	1093.2255	537.1043 (100); 555.1151 (40); 295.0613 (8)
37	Globoidnan A	14.9	C_26_H_20_O_10_	1	11.6	491.0979	311.0557 (100), 267.0658 (79)
38	Pulmitric acid A	15	C_28_H_24_O_12_	0.9	42.8	551.119	463.1394 (34); 295.0608 (100); 255.0657 (60)
39	Pulmitric acid B	15.3	C_27_H_20_O_12_	−0.5	13.6	535.0885	359.0768 (38); 177.0197 (100)
40	Isosalvianolic acid A	15.6	C_26_H_22_O_10_	2.3	18.9	493.1134	295.0601 (100); 185.0250 (15)
41	Isosalvianolic acid A-1	15.7	C_26_H_22_O_10_	1.3	4.5	493.1134	295.0601 (100); 185.0250 (21)
42	Isosalvianolic acid A isomer	15.8	C_26_H_22_O_10_	−10.4	39	493.1134	295.0601 (100); 359.0775 (36); 185.0250 (11)
43	Rosmarinic acid methyl ester	15.9	C_19_H_18_O_8_	−0.9	10.5	373.0932	179.0353 (53); 135.0445 (25)
44	Salvianolic acid H-9″-methylester	16.4	C_28_H_24_O_12_	1	10.9	551.1189	519.0919 (15); 359.0766 (48); 193.0502 (100)
45	Lycopic acid C	20.6	C_27_H_19_O_11_	1.6	25.6	519.0933	339.0499 (100),161.0227 (14), 179.0337 (5)

**Table 2:** ^1^H and ^13^C-NMR data (MeOH-*d*_4_, 500/125 MHz) for compounds **3** and **14**.

Position	3		14	
	δ_H_ (*J* in Hz)	δ_C_	δ_H_ (*J* in Hz)	δ_C_
1		175.4		174.8
2	4.47 d (2.5)	70.5	4.44 br d (6.0)	70.3
3	5.34 ddd (6.8, 6.3, 2.5)	75.8	4.46 m, 4.38 m	66.9
4	3.81 dd (11.2, 6.8) 3.75 dd (11.2, 6.3)	60.9		
1′		127.8		127.7
2′	7.04 d (2.1)	115.1	7.04 d (2.1)	115.1
3′		146.8		146.8
4′		149.6		149.7
5′	6.77 d (8.2)	116.5	6.78 d (8.2)	116.5
6′	6.94 dd (8.2, 2.1)	123.0	6.94 dd (8.2, 2.1)	123.0
7′	7.59 d (15.9)	147.5	7.58 d (15.9)	147.4
8′	6.26 d (15.9)	114.8	6.27 d (15.9)	114.6
9′		168.3		168.9

**Table 3:** ^1^H and ^13^C-NMR data (MeOH-*d*_4_ + 0.1% TFA, 500/125 MHz) for compounds **38**–**41**.

Position	38		39		40/41	
	δ_H_ (*J* in Hz)	δ_C_	δ_H_ (*J* in Hz)	δ_C_	δ_H_ (*J* in Hz)	δ_C_
1		137.2		128.2		134.7
2	6.98 br s	115.3	7.35 d (2.1)	121.5	6.87 d (1.8)	114.1
3		145.6		144.1		146.5
4		145.9		152.4		146.5
5	6.67 d (8.1)	116.7	6.99 d (8.3)	118.8	6.76 d (8.2)	116.3
6	6.84 dd (8.1, 2.1)	123.9	7.37 dd (8.3, 2.1)	127.9	6.78 dd (8.2, 1.8)	118.9
7	2.99 dd (15.4, 8.5) 2.96 dd (15.4, 7.6)	40.1	7.59 d (15.9)	146.4	5.71 dd (9.4,8.1)	86.2
8	4.83 t (8.2)	39.3	6.36 d (15.9)	115.9	3.74 dd (15.9, 9.4) 3.26 dd (15.9, 8.1)	39.3
9		174.5		168.1	-	-
9-OMe	3.54 s	52.1				
1′		129.4		129.2		123.8
2′	6.52 d (1.9)	114.8	6.73 d (2.1)	117.6		129.8
3′		146.4		146.1		148.3
4′		142.6		145.3		145.0
5′		132.8	6.68 d (8.1)	116.3	6.72 d (8.4)	117.1
6′	6.72 br s	120.0	6.60 dd (8.1, 2.1)	121.8	7.06 d (8.4)	123.0
7′	3.15 dd (14.2, 1.6) 2.98 dd (14.2, 11.4)	38.8	3.08 dd (14.2, 4.3) 2.99 dd (14.2, 8.3)	37.9	7.62 d (16.0)	145.2
8′	5.23 dd (11.4, 1.6)	75.3	5.18 dd (8.3, 4.3)	74.7	6.25 d (16.0)	115.9
9′		172.7		173.4		168.3
1′′		126.0		112.6		129.2
2′′	7.40 d (2.1)	118.0		146.8	6.74 d (2.1)	117.6
3′		146.3	6.79 s	103.5		146.2
4′′		148.5		149.8		145.3
5′′	6.79 d (8.3)	116.4		144.8	6.69 d (8.1)	116.3
6′′	7.16 dd (8.3, 2.1)	124.7	6.81 s	112.4	6.60 dd (8.1, 2.1)	121.8
7′′	7.13 s	127.0	7.03 s	123.8	3.09 dd (14.3, 4.3) 3.00 dd (14.3, 8.4)	37.9
8′′		140.2		140.9	5.19 dd (8.4, 4.3)	74.6
9′′		164.2		160.2		173.4

**Table 4:** ^1^H and ^13^C-NMR data (MeOH-*d*_4_, 500/125 MHz) for compounds **18**, **32** and **35**.

Position	18		32		35	
	δ_H_ (*J* in Hz)	δ_C_	δ_H_ (*J* in Hz)	δ_C_	δ_H_ (*J* in Hz)	δ_C_
1	4.40 d (2.8)	46.9	4.34 dd (15.3, 1.0)	48.1	4.34 dd (15.0, 1.4)	48.1
2	3.84 d (2.8)	48.6	4.21 dd (15.3, 2.5)	51.7	4.21 dd (15.2, 2.5)	51.6
3		122.7		126.2		126.1
4	7.58 s	140.3	7.39 d (2.5)	140.3	7.39 d (2.5)	140.3
4a		124.9		124.1		124.0
5	6.83 s	117.2	6.73 s	113.6	6.68 s	113.6
6		145.6		147.7		147.7
7		149.2		150.1		150.2
8	6.55 br s	117.2	6.11 s	116.1	6.11 t (0.9)	116.2
8a		131.6		134.7		134.7
9		168.0		168.1		168.0
10		176.2		176.4		176.3
6-OMe			3.79 s	56.6	3.77 s	56.6
1′		136.3		133.5		133.5
2′	6.43 d (2.2)	115.8	6.87 d (2.0)	114.5	6.87 d (2.0)	114.4
3′		146.0		149.0		149.0
4′		144.9		146.7		146.7
5′	6.62 d (8.2)	116.3	6.90 d (8.1)	116.9	6.89 d (8.1)	116.9
6′	6.39 dd (8.2, 2.2)	119.9	6.81 dd (8.1, 2.0)	123.3	6.81 dd (8.1, 2.0)	123.4
3′-OMe	-	-	3.84 s	56.8	3.84 s	56.8
1′′		129.1	3.90 d (12.4) 3.72 d (12.4)	63.2	3.89 d (12.4) 3.72 d (12.4)	63.3
2′′	6.71 d (2.1)	117.6		110.1		110.1
3′		146.1	4.63 o	81.9	4.62 s	82.0
4′′		145.2	4.63 o	73.5	4.66 d (0.9)	73.5
5′′	6.68 d (8.1)	116.4	4.24 br d (2.4)	87.5	4.24 t (1.4)	87.5
6′′	6.56 dd (8.1, 2.1)	122.0	4.71 dd (12.4, 2.4) 4.08 d (12.4)	66.4	4.69 dd (12.2, 2.5) 4.09 d (12.2)	66.4
7′′	3.04 dd (14.3, 5.3) 3.00 dd (14.3, 7.2)	37.9				
8′′	5.12 dd (7.2, 5.2)	74.9				
9′′						
1′′′			5.35 d (3.6)	94.4	5.35 d (3.6)	94.4
2′′′			3.46 dd (9.6, 3.6)	73.4	3.46 dd (9.6, 3.6)	73.4
3′′′			3.64 t (9.2)	75.0	3.65 t (9.2)	75.0
4′′′			3.31 dd (10.0, 8.9)	72.0	3.29 dd (10.0, 9.0)	72.2
5′′′			4.32 ddd (9.6, 6.5, 2.2)	72.3	4.33 ddd (9.8, 7.0, 2.4)	72.3
6′′′			4.48 dd (12.0, 2.2) 4.16 dd (12.0, 6.5)	65.5	4.47 dd (11.9, 2.4) 4.16 dd (11.9, 7.0)	65.8
1′′′′				130.6		130.8
2′′′′			7.07 d (2.1)	116.5	7.26 d (2.0)	112.4
3′′′′				148.7		152.0
4′′′′				147.9		149.0
5′′′′			7.18 d (8.4)	118.0	7.17 d (8.4)	118.6
6′′′′			7.01 dd (8.4, 2.1)	122.2	7.09 dd (8.4, 2.0)	123.7
7′′′′			7.47 d (15.9)	146.3	7.53 d (15.9)	146.3
8′′′′			6.21 d (15.9)	116.8	6.29 d (15.9)	117.0
9′′′′				168.9		168.9
3′′″-OMe					3.92 s	56.7
1′′′′′			5.50 d (1.8)	100.7	5.48 d (1.8)	100.8
2′′′′′			4.14 dd (3.5, 1.8)	71.8	4.10 dd (3.5, 1.8)	71.9
3′′′′′			3.98 dd (9.5, 3.5)	72.1	3.92 dd (9.5, 3.5)	72.2
4′′′′′			3.49 t (9.5)	73.8	3.48 t (9.5)	73.7
5′′′′′			3.74 dq (9.5, 6.2)	70.9	3.76 dq (9.5, 6.2)	71.0
6′′′′′			1.25 d (6.2)	18.0	1.24 d (6.2)	18.0

**Table 5:** ^1^H and ^13^C-NMR data (MeOH-*d*_4_, 500/125 MHz) for compounds **10** and **30**.

Position	10		30 *	
	δ_H_ (*J* in Hz)	δ_C_	δ_H_ (*J* in Hz)	δ_C_
1	5.44 d (3.7)	93.3		141.2
2	3.43 dd (9.8, 3.7)	73.2	7.87 d (2.0)	131.4
3	3.66 t (9.4)	75.0		132.5
4	3.40 dd (9.9, 8.9)	71.2		142.1
5	3.93 ddd (9.9, 4.5, 2.5)	74.6	7.19 d (8.0)	133.3
6	3.85 dd (12.0, 2.5) 3.77 dd (12.0, 4.5)	62.4	7.67 dd (8.0, 2.0)	132.7
7			3.25 m, 3.17 m	29.8
8			2.70 m	36.1
9				173.8
10				192.0
11				167.8
1′	3.66 d (12.2) 3.59 d (12.2)	65.4		132.4
2′		104.8	7.04 d (2.2)	114.8
3′	5.46 d (7.9)	79.7		146.9
4′	4.38 t (7.9)	73.9		146.8
5′	3.93 ddd (7.9, 5.6, 3.5)	84.2	6.85 d (8.2)	116.9
6′	3.84 dd (12.3, 5.6) 3.80 dd (12.3, 3.5)	62.9	6.94 dd (8.2, 2.2)	119.4
1′′		127.7		129.2
2′′	7.23 d (2.0)	112.1	6.73 d (2.1)	117.5
3′′		149.4		146.2
4′′		150.7		145.4
5′′	6.81 d (8.3)	116.5	6.70 d (8.1)	116.4
6′′	7.14 dd (8.3, 2.0)	124.2	6.59 dd (8.1, 2.1)	121.9
7′′	7.71 d (15.9)	147.7	3.07 dd (14.3, 4.0) 2.93 dd (14.3, 8.9)	37.8
8′′	6.43 d (15.9)	115.1	5.10 dd (8.9, 4.0)	74.8
9′′		168.3		173.2
3″-OMe	3.91 s	56.5		

* analyzed with an addition of 0.1% TFA.

**Table 6:** Comparison of metabolite content observed in spring and autumn extracts of *P. officinalis* aerial parts.

No.	Compound Name	Contents [µg/g DW] (Mean ± SD, *n* = 3)	
		Spring	Autumn
1	Danshensu	20.6 ± 0.2	59.5 ± 4.6
2	Menisdaurin	107.3 ± 3.6	ND
3	3-*O*-(*E*)-caffeoyl-threonic acid	90.4 ± 1.8	27.7 ± 2.0
4	2-*O*-(*E*)-caffeoyl-l-threonic acid	567.4 ± 33.1	123.5 ± 19.7
5	Lycoperodine-1	8.1 ± 0.8	ND
6	Chlorogenic acid	240.9 ± 12.3	330.7 ± 16.6
7	Actinidioionoside	26.1 ± 6.8	397.0 ± 15.4
8	Caffeic acid	23.3 ± 0.8	119.5 ± 8.8
9	Cryptochlorogenic acid	5.5 ± 0.8	30.9 ± 2.4
10	3′-*O*-(*E*)-feruloyl-α-sorbopyranosyl-(2′→1)-α-glucopyranoside	14.8 ± 0.8	23.0 ± 1.4
11	2-*O*-(*E*)-caffeoyl-d-glyceric acid	465.3 ± 22.7	227.8 ± 11.2
12	4-*O*-(*E*)-caffeoyl-l-threonic acid	48.6 ± 3.0	20.4 ± 1.4
13	Neochlorogenic acid	28.4 ± 1.7	37.8 ± 3.7
14	3-*O*-(*E*)-caffeoyl- glyceric acid	18.4 ± 1.5	7.1 ± 1.1
15	3-*O*-*p*-coumaroylquinic acid	111.6 ± 6.3	362.9 ± 24.1
16	4-*O*-*p*-coumaroylquinic acid	TR	13.9 ± 0.8
17	5*-O*-*p*-coumaroylquinic acid	152.6 ± 10.7	420.4 ± 29.8
18	Globoidnan B	6843.6 ± 853.4	3797.6 ± 845.4
19	Rutin	369.9 ± 9.4	57.1 ± 18.4
20	Nicotiflorin isomer	3.1 ± 0.3	ND
21	Quercetin 3-*O*-*β*-glucoside	253.6 ± 7.1	227.7 ± 10.5
22	Yunnaneic acid E	103.0 ± 3.8	183.1 ± 33.7
23	Quercetin 3-*O*-(6″-*O*-malonyl)-*β*-glucoside	1563.4 ± 109.2	858.8 ± 44.5
24	Nicotiflorin	184.8 ± 4.5	69.7 ± 4.8
25	Astragalin	146.6 ± 3.2	513.3 ± 28.2
26	Shimobashiric acid C	1188.0 ± 46.2	1797.8 ± 115.0
27	Rosmarinic acid	7002.1 ± 345.8	12201.5 ± 503.2
28	Kaempferol 3*-O*-(6″-*O*-malonyl)-*β*-glucoside	731.6 ± 45.5	1567.2 ± 86.3
29	Monardic acid A	806.8 ±168.5	971.7 ± 75.0
30	Yunnaneic acid E-1	NA	NA
31	Lithospermic acid A	609.7 ± 110.7	576.3 ± 37.8
32	Pulmonarioside A	18.0 ± 1.5	91.5 ± 5.1
33	Salvianolic acid H	29.6 ± 9.3	261.9 ± 17.3
34	Lithospermic acid B	NA	NA
35	Pulmonarioside B	147.5 ± 12.8	199.6 ± 5.7
36	Yunnaneic acid B	216.8 ± 29.3	1834.6 ± 40.5
37	Globoidnan A	21.7 ± 3.5	27.1 ± 2.2
38	Pulmitric acid A	TR	TR
39	Pulmitric acid B	TR	TR
40	Isosalvianolic acid A	TR	0.7 ±0.1
41	Isosalvianolic acid A-1	TR	TR
42	Isosalvianolic acid A isomer	TR	1.8 ± 0.3
43	Rosmarinic acid methyl ester	15.4 ± 0.9	15.8 ± 1.4
44	Salvianolic acid H-9″-methylester	5.6 ± 2.9	31.5 ± 4.1
45	Lycopic acid C	NA	NA

TR—traces, indicates level below the limit of quantification; NA—not analyzed; ND—not detected.

**Table 7:** Meteorological data for April and September of 2015.

Meteorological Data for GPS Coordinates: 51°24′47.5″ N, 21°57′54.7″ E		
	April 2015	September 2015
Average temperature (°C)	8.6	15.3
Average minimal (°C)	4.0	11.8
Average maximal (°C)	13.9	20.2
Rainfall (mm)	28.5	126
Humidity (%)	78	89

## Supplementary Materials

The Supplementary Materials are available online.
