# Supplementary material for: Novel Phenolic Constituents of Pulmonaria officinalis L. LC-MS/MS Comparison of Spring and Autumn Metabolite Profiles
Source: Molecules. 2018 Sep 6;23(9):2277. doi: 10.3390/molecules23092277 (PMC6225171; doi:10.3390/molecules23092277)
Supplement: Supplementary file 1 [file molecules-23-02277-s001.pdf]

SUPPLEMENTARY MATERIALS

Novel phenolic constituents of *Pulmonaria officinalis* L. LC-MS/MS comparison of spring and autumn metabolite profiles

Justyna Krzyżanowska-Kowalczyk[1*], Łukasz Pecio[1], Jarosław Mołdoch[1], Agnieszka Ludwiczuk[2], Mariusz Kowalczyk[1]

[1]Department of Biochemistry and Crop Quality, Institute of Soil Science and Plant Cultivation - State Research Institute, Czartoryskich 8, 24-100 Puławy, Poland; jkrzyzanowska@iung.pulawy.pl (J.K.K.); lpecio@iung.pulawy.pl (Ł.P); jmoldoch@iung.pulawy.pl (J.M.); mkowalczyk@iung.pulawy.pl (M.K.)

[2]Department of Pharmacognosy with Medicinal Plant Unit, Medical University of Lublin, Chodzki Str.1, 20-093 Lublin, Poland; aludwiczuk@pharmacognosy.org (A.L.)

***Correspondence**:jkrzyzanowska@iung.pulawy.pl; Tel.: +48-81-4786-881

Spis treści

Figure 1S. ^{1}H (500 MHz) and ^{13}C (125 MHz) NMR data of compound 1 in CD_3OD, 25°C; on-line PDA UV spectrum in MeCN/H_2O 6

Figure 2S. ^{1}H (500 MHz) and ^{13}C (125 MHz) NMR data of compound 2 in CD_3OD, 25°C; on-line PDA UV spectrum in MeCN/H_2O 6

Figure 3S. ^{1}H (500 MHz) and ^{13}C (125 MHz) NMR data of compound 3 in CD_3OD, 25°C; on-line PDA UV spectrum in MeCN/H_2O, ECD spectrum in MeOH 7

Figure 4S. ^{1}H NMR spectrum of compound 3 7

Figure 5S. ^{13}C UDEFT NMR spectrum of compound 3 8

Figure 6S. ^{1}H-^{1}H COSY NMR spectrum of compound 3 8

Figure 7S. ^{1}H-^{1}H TROESY (250 ms) NMR spectrum of compound 3 9

Figure 8S. ^{1}H-^{13}C HSQC NMR spectrum of compound 3 9

Figure 9S. ^{1}H-^{13}C H2BC NMR spectrum of compound 3 10

Figure 10S. ^{1}H-^{13}C HMBC (8 Hz) NMR spectrum of compound 3 10

Figure 11S. ^{1}H (500 MHz) and ^{13}C (125 MHz) NMR data of compound 4 in CD_3OD, 25°C; on-line PDA UV spectrum in MeCN/H_2O, ECD spectrum in MeOH 11

Figure 12S. ^{1}H (500 MHz) ^{13}C (125 MHz) and ^{15}N (50 MHz) NMR data of compound 5 in CD_3OD, 25°C....11

Figure 13S. ^{1}H (500 MHz) and ^{13}C (125 MHz) NMR data of compound 7 in CD_3OD, 25°C....12

Figure 14S. ^{1}H (500 MHz) and ^{13}C (125 MHz) NMR data of compound 10 in CD_3OD, 25°C; on-line PDA UV spectrum in MeCN/H_2O 12

Figure 15S. ^{1}H NMR spectrum of compound 10....13

Figure 16S. ^{13}C DEPTQ NMR spectrum of compound 10....13

Figure 17S. ^{1}H-^{1}H COSY NMR spectrum of compound 10 14

Figure 18S. ^{1}H-^{1}H TROESY (250 ms) NMR spectrum of compound 10 14

Figure 19S. ^{1}H-^{13}C HSQC NMR spectrum of compound 10....15

Figure 20S. ^{1}H-^{13}C H2BC NMR spectrum of compound 10 15

Figure 21S. ^{1}H-^{13}C HMBC (8 Hz) NMR spectrum of compound 10 16

Figure 22S. ^{1}H (500 MHz) and ^{13}C (125 MHz) NMR data of compound 11 in CD_3OD, 25°C; on-line PDA UV spectrum in MeCN/H_2O; ECD spectrum in MeOH 16

Figure 23S. ^{1}H (500 MHz) and ^{13}C (125 MHz) NMR data of compound 12 in CD_3OD, 25°C; on-line PDA UV spectrum in MeCN/H_2O; ECD spectrum in MeOH 17

Figure 24S. ^{1}H (500 MHz) and ^{13}C (125 MHz) NMR data of compound 14 in CD_3OD, 25°C; on-line PDA UV spectrum in MeCN/H_2O; ECD spectrum in MeOH 17

Figure 25S. ^{1}H NMR spectrum of compound 14....18

Figure 26S. ^{13}C DEPTQ NMR spectrum of compound 14....18

Figure 27S. ^{1}H-^{1}H COSY NMR spectrum of compound 14 19

Figure 28S. ^{1}H-^{1}H NOESY (400 ms) NMR spectrum of compound 14....19

Figure 29S. ^{1}H-^{13}C HSQC NMR spectrum of compound 14....20

Figure 30S. ^{1}H-^{13}C H2BC NMR spectrum of compound 14 20

Figure 31S. ^{1}H-^{13}C HMBC (8 Hz) NMR spectrum of compound 14 21

Figure 32S. ^{1}H (500 MHz) and ^{13}C (125 MHz) NMR data of compound 15 in CD_3OD, 25°C; on-line PDA UV spectrum in MeCN/H_2O 21

Figure 33S. ^{1}H (500 MHz) and ^{13}C (125 MHz) NMR data of compound 18 in CD_3OD, 25°C; on-line PDA UV spectrum in $MeCN/H_2O$; ECD spectrum in MeOH 22

Figure 34S. ^{1}H NMR spectrum of compound 18 22

Figure 35S. ^{13}C DEPTQ NMR spectrum of compound 18 23

Figure 36S. ^{1}H-^{1}H COSY NMR spectrum of compound 18 23

Figure 37S. ^{1}H-^{1}H TROESY (250 ms) NMR spectrum of compound 18 24

Figure 38S. ^{1}H-^{13}C HSQC NMR spectrum of compound 18 24

Figure 39S. ^{1}H-^{13}C 1,1-ADEQUATE NMR spectrum of compound 18 25

Figure 40S. ^{1}H-^{13}C HMBC (8 Hz) NMR spectrum of compound 18 25

Figure 41S. ^{1}H (500 MHz) and ^{13}C (125 MHz) NMR data of compound 19 in CD_3OD, 25°C; on-line PDA UV spectrum in $MeCN/H_2O$ 26

Figure 42S. ^{1}H (500 MHz) and ^{13}C (125 MHz) NMR data of compound 21 in CD_3OD, 25°C; on-line PDA UV spectrum in $MeCN/H_2O$ 26

Figure 43S. ^{1}H (500 MHz) and ^{13}C (125 MHz) NMR data of compound 22 in CD_3OD, 25°C; on-line PDA UV spectrum in $MeCN/H_2O$ 27

Figure 44S. ^{1}H (500 MHz) and ^{13}C (125 MHz) NMR data of compound 23 in CD_3OD, 25°C; on-line PDA UV spectrum in $MeCN/H_2O$ 27

Figure 45S. ^{1}H (500 MHz) and ^{13}C (125 MHz) NMR data of compound 24 in CD_3OD, 25°C; on-line PDA UV spectrum in $MeCN/H_2O$ 28

Figure 46S. ^{1}H (500 MHz) and ^{13}C (125 MHz) NMR data of compound 25 in CD_3OD, 25°C; on-line PDA UV spectrum in $MeCN/H_2O$ 28

Figure 47S. ^{1}H (500 MHz) and ^{13}C (125 MHz) NMR data of compound 26 in DMSO-d_6, 25°C; on-line PDA UV spectrum in $MeCN/H_2O$ 29

Figure 48S. ^{1}H (500 MHz) and ^{13}C (125 MHz) NMR data of compound 27 in CD_3OD, 25°C; on-line PDA UV spectrum in $MeCN/H_2O$; ECD spectrum in MeOH 29

Figure 49S. ^{1}H (500 MHz) and ^{13}C (125 MHz) NMR data of compound 28 in CD_3OD, 25°C; on-line PDA UV spectrum in $MeCN/H_2O$ 30

Figure 50S. ^{1}H (500 MHz) and ^{13}C (125 MHz) NMR data of compound 29 in CD_3OD, 25°C; on-line PDA UV spectrum in $MeCN/H_2O$; ECD spectrum in MeOH 30

Figure 51S. ^{1}H (500 MHz) and ^{13}C (125 MHz) NMR data of compound 30 in CD_3OD, 25°C; on-line PDA UV spectrum in $MeCN/H_2O$ 31

Figure 52S. ^{1}H NMR spectrum of compound 30 31

Figure 53S. ^{13}C DEPTQ NMR spectrum of compound 30 32

Figure 54S. ^{1}H-^{1}H COSY NMR spectrum of compound 30 32

Figure 55S. ^{1}H-^{1}H TROESY (250 ms) NMR spectrum of compound 30 33

Figure 56S. ^{1}H-^{13}C HSQC NMR spectrum of compound 30 33

Figure 57S. ^{1}H-^{13}C H2BC NMR spectrum of compound 30 34

Figure 58S. ^{1}H-^{13}C HMBC (8 Hz) NMR spectrum of compound 30 34

Figure 59S. ^{1}H (500 MHz) and ^{13}C (125 MHz) NMR data of compound 31 in CD_3OD, 25°C; on-line PDA UV spectrum in $MeCN/H_2O$ 35

Figure 60S. ^{1}H (500 MHz) and ^{13}C (125 MHz) NMR data of compound 32 in CD_3OD, 25°C; on-line PDA UV spectrum in $MeCN/H_2O$ 35

Figure 61S. ^{1}H NMR spectrum of compound 32 36

Figure 62S. ^{13}C DEPTQ NMR spectrum of compound 32 36

Figure 63S. ^{1}H-^{1}H COSY NMR spectrum of compound 32 37

Figure 64S. ^{1}H-^{1}H TROESY (250 ms) NMR spectrum of compound 32 37

Figure 65S. ^{1}H-^{13}C HSQC NMR spectrum of compound 32 38

Figure 66S. ^{1}H-^{13}C H2BC NMR spectrum of compound 32 38

Figure 67S. ^{1}H-^{13}C HMBC (8 Hz) NMR spectrum of compound 32 39

Figure 68S. ^{1}H (500 MHz) and ^{13}C (125 MHz) NMR data of compound 33 in CD_3OD, 25°C; on-line PDA UV spectrum in $MeCN/H_2O$ 39

Figure 69S. ^{1}H (500 MHz) and ^{13}C (125 MHz) NMR data of compound 34 in CD_3OD, 25°C; on-line PDA UV spectrum in $MeCN/H_2O$; ECD spectrum in MeOH 40

Figure 70S. ^{1}H (500 MHz) and ^{13}C (125 MHz) NMR data of compound 35 in CD_3OD, 25°C; on-line PDA UV spectrum in $MeCN/H_2O$; ECD spectrum in MeOH 40

Figure 71S. ^{1}H NMR spectrum of compound 35 41

Figure 72S. ^{13}C DEPTQ NMR spectrum of compound 35 41

Figure 73S. ^{1}H-^{1}H COSY NMR spectrum of compound 35 42

Figure 74S. ^{1}H-^{1}H TROESY (250 ms) NMR spectrum of compound 35 42

Figure 75S. ^{1}H-^{13}C HSQC NMR spectrum of compound 35 43

Figure 76S. ^{1}H-^{13}C H2BC NMR spectrum of compound 35 43

Figure 77S. ^{1}H-^{13}C HMBC (8 Hz) NMR spectrum of compound 35 44

Figure 78S. ^{1}H (500 MHz) and ^{13}C (125 MHz) NMR data of compound 36 in CD_3OD, 25°C; on-line PDA UV spectrum in $MeCN/H_2O$; ECD spectrum in MeOH 44

Figure 79S. ^{1}H (500 MHz) and ^{13}C (125 MHz) NMR data of compound 37 in CD_3OD, 25°C; on-line PDA UV spectrum in $MeCN/H_2O$ 45

Figure 80S. ^{1}H (500 MHz) and ^{13}C (125 MHz) NMR data of compound 38 in CD_3OD, 25°C; on-line PDA UV spectrum in $MeCN/H_2O$ 45

Figure 81S. ^{1}H NMR spectrum of compound 38 46

Figure 82S. ^{13}C DEPTQ NMR spectrum of compound 38 46

Figure 83S. ^{1}H-^{1}H COSY NMR spectrum of compound 38 47

Figure 84S. ^{1}H-^{1}H NOESY (300 ms) NMR spectrum of compound 38 47

Figure 85S. ^{1}H-^{13}C HSQC NMR spectrum of compound 38 48

Figure 86S. ^{1}H-^{13}C H2BC NMR spectrum of compound 38 48

Figure 87S. ^{1}H-^{13}C HMBC (8 Hz) NMR spectrum of compound 38 49

Figure 88S. ^{1}H (500 MHz) and ^{13}C (125 MHz) NMR data of compound 39 in CD_3OD, 25°C 49

Figure 89S. ^{1}H NMR spectrum of compound 39 50

Figure 90S. ^{13}C DEPTQ NMR spectrum of compound 39 50

Figure 91S. ^{1}H-^{1}H COSY NMR spectrum of compound 39 51

Figure 92S. ^{1}H-^{1}H NOESY (300 ms) NMR spectrum of compound 39 51

Figure 93S. ^{1}H-^{13}C HSQC NMR spectrum of compound 39 52

Figure 94S. ^{1}H-^{13}C H2BC NMR spectrum of compound 39 52

Figure 95S. ^{1}H-^{13}C HMBC (8 Hz) NMR spectrum of compound 39 53

Figure 96S. ^{1}H (500 MHz) and ^{13}C (125 MHz) NMR data of compound 40 in CD_3OD, 25°C; on-line PDA UV spectrum in $MeCN/H_2O$; ECD spectrum in MeOH 53

Figure 97S. ^{1}H (500 MHz) and ^{13}C (125 MHz) NMR data of compound 41 in CD_3OD, 25°C; on-line PDA UV spectrum in $MeCN/H_2O$; ECD spectrum in MeOH 54

Figure 98S. ^{1}H NMR spectrum of compound 41 54

Figure 99S. ^{13}C DEPTQ NMR spectrum of compound 41 55

Figure 100S. ^{1}H-^{1}H COSY NMR spectrum of compound 41 55

Figure 101S. ^{1}H-^{1}H TROESY (250 ms) NMR spectrum of compound 41 56

Figure 102S. ^{1}H-^{13}C HSQC NMR spectrum of compound 41 56

Figure 103S. ^{1}H-^{13}C H2BC NMR spectrum of compound 41 57

Figure 104S. ^{1}H-^{13}C HMBC (8 Hz) NMR spectrum of compound 41 57

Figure 105S. ^{1}H (500 MHz) and ^{13}C (125 MHz) NMR data of compound 43 in CD_3OD, 25°C; on-line PDA UV spectrum in $MeCN/H_2O$ 58

Figure 106S. ^{1}H (500 MHz) and ^{13}C (125 MHz) NMR data of compound 44 in CD_3OD, 25°C; on-line PDA UV spectrum in $MeCN/H_2O$ 58

Table S1. 59

Figure 107S. Frequency distribution of the relative standard deviation for the peak intensities and peak numbers in QC samples 60

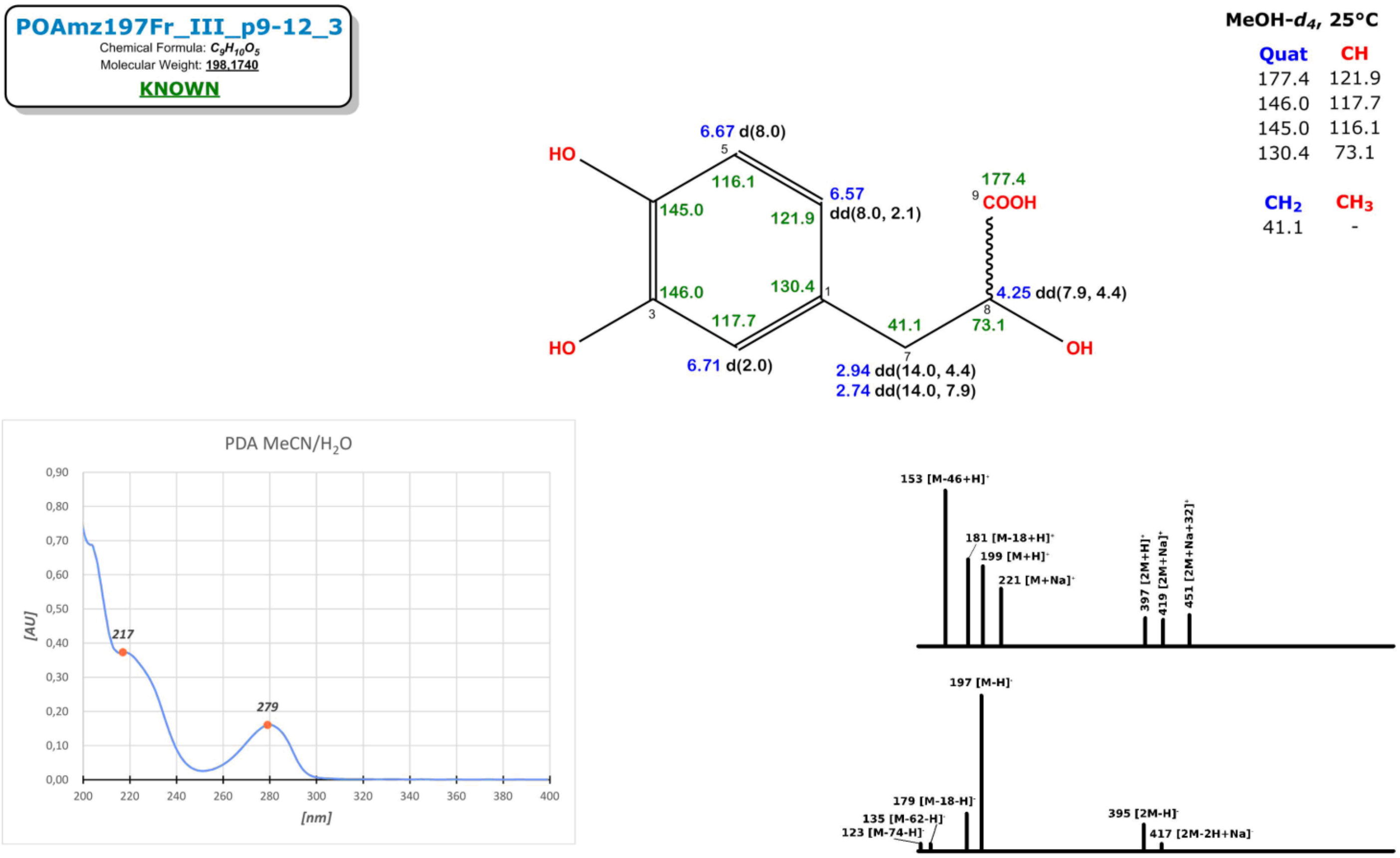

Danshensu
(3,4-dihydroxyphenyl)lactic acid

Figure 1S. ^{1}H (500 MHz) and ^{13}C (125 MHz) NMR data of compound 1 in CD_3OD, 25°C; on-line PDA UV spectrum in MeCN/H_2O

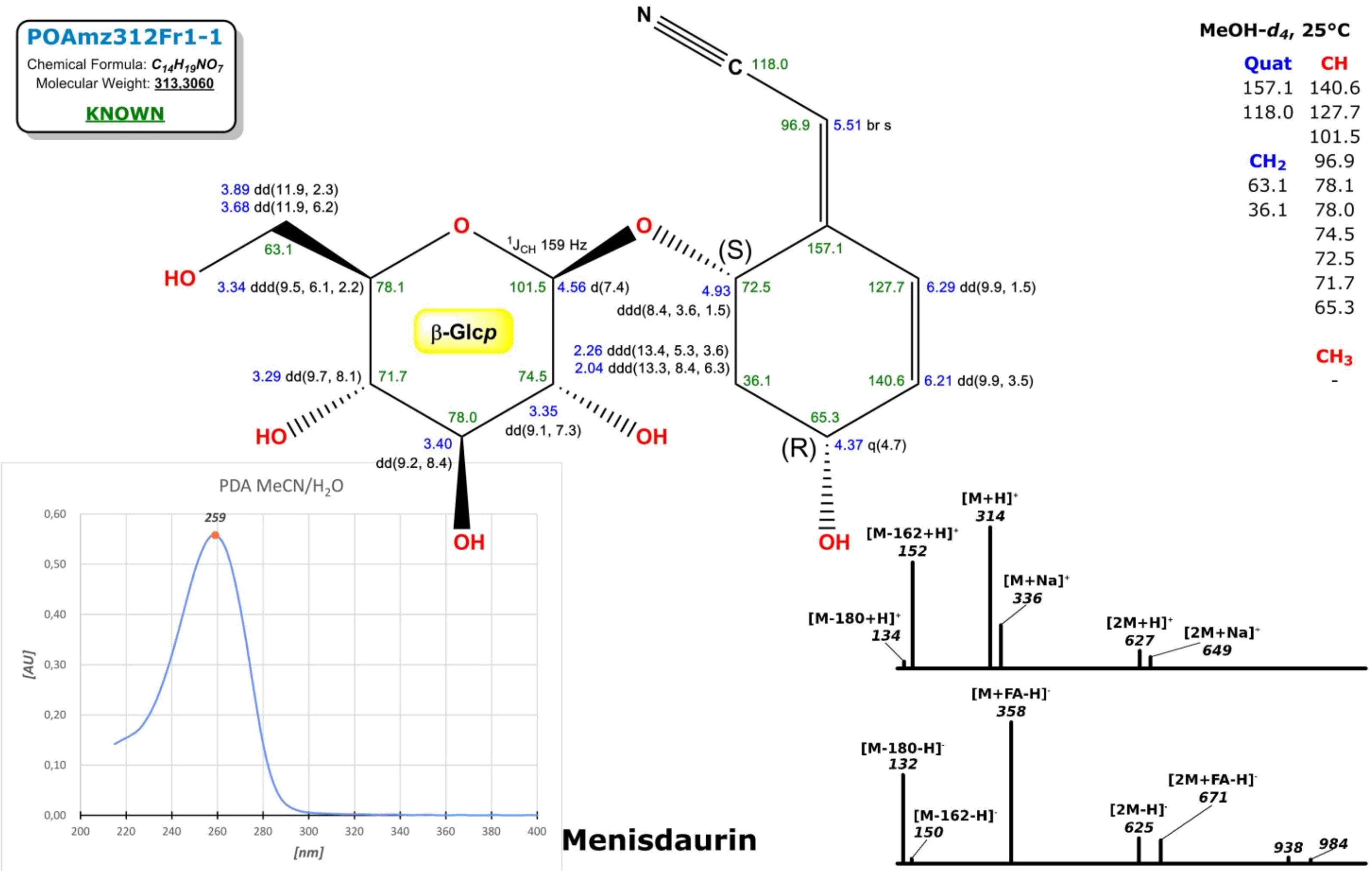

Menisdaurin

1. Seigler, D.S., Pauli, G.F., Fröhlich, R., Wegelius, E., Nahrstedt, A., Glander, K.E., Ebinger, J.E., 2005. Cyanogenic glycosides and menisdaurin from Guazuma ulmifolia, Ostrya virginiana, Tiquilia plicata, and Tiquilia canescens. Phytochemistry 66, 1567–1580. doi:10.1016/j.phytochem.2005.02.021

Figure 2S. ^{1}H (500 MHz) and ^{13}C (125 MHz) NMR data of compound 2 in CD_3OD, 25°C; on-line PDA UV spectrum in MeCN/H_2O

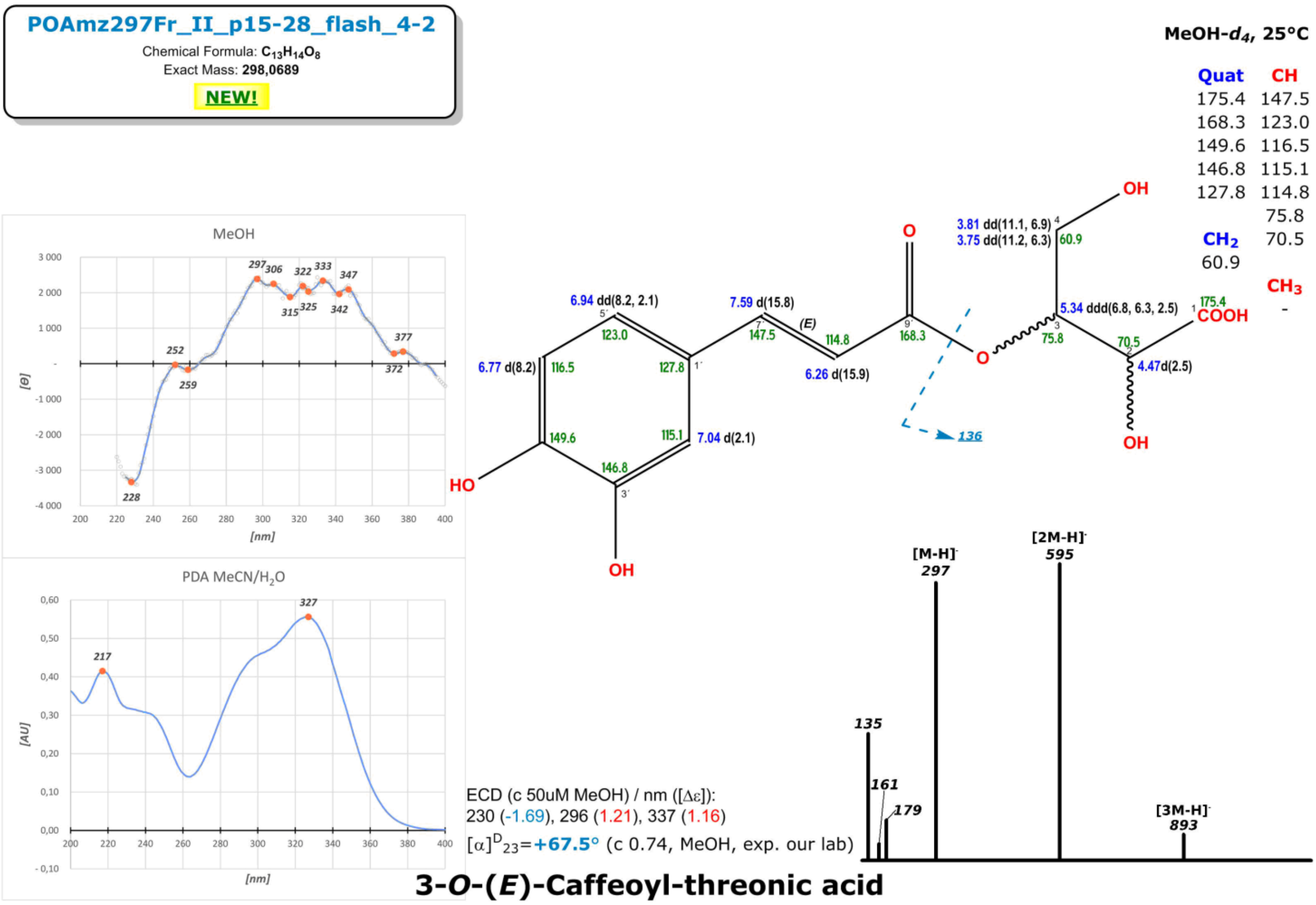

Figure 3S. ^{1}H (500 MHz) and ^{13}C (125 MHz) NMR data of compound 3 in CD_3OD, 25°C; on-line PDA UV spectrum in $MeCN/H_2O$, ECD spectrum in MeOH

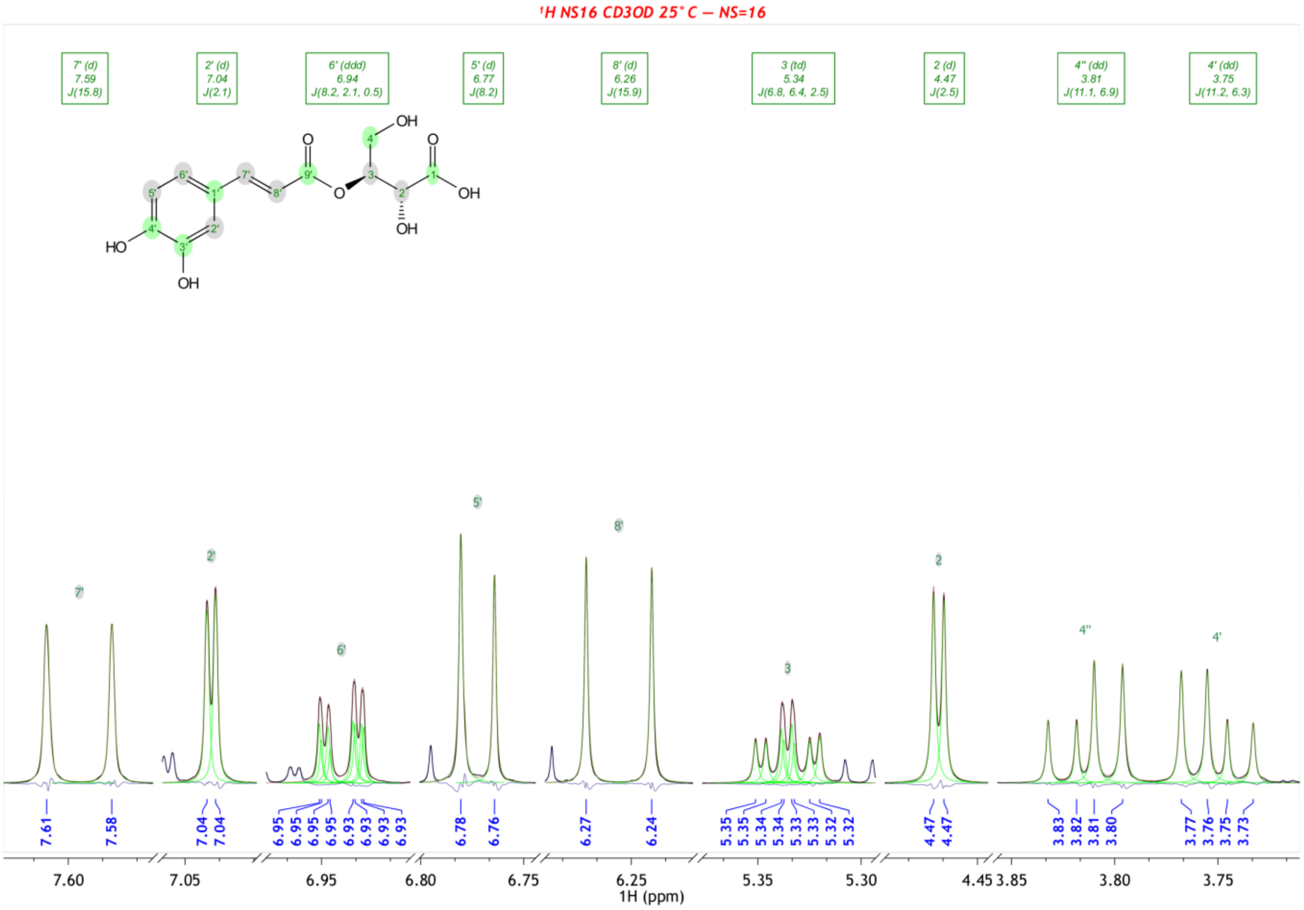

Figure 4S. ^{1}H NMR spectrum of compound 3

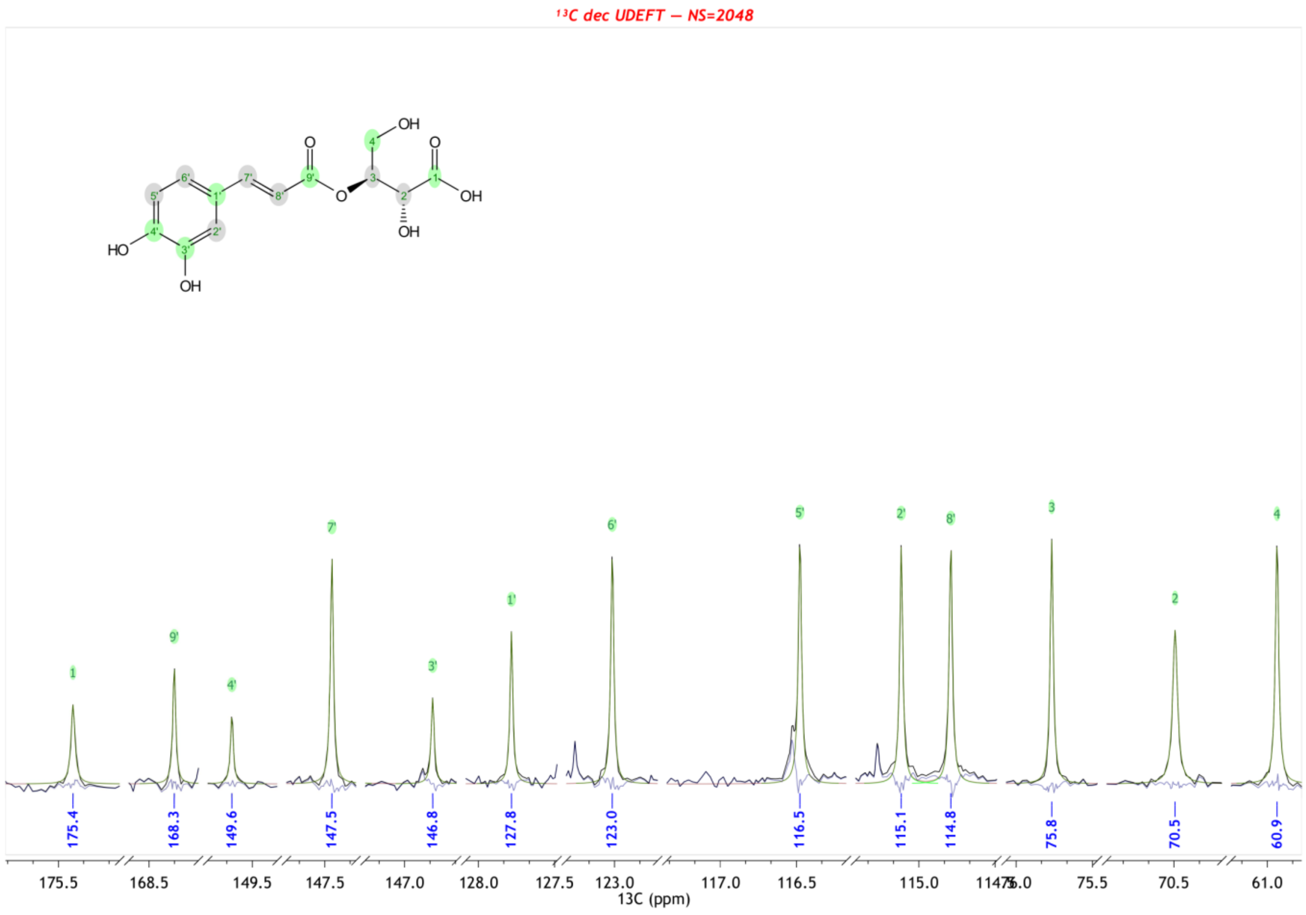

Figure 5S. ^{13}C UDEFT NMR spectrum of compound 3

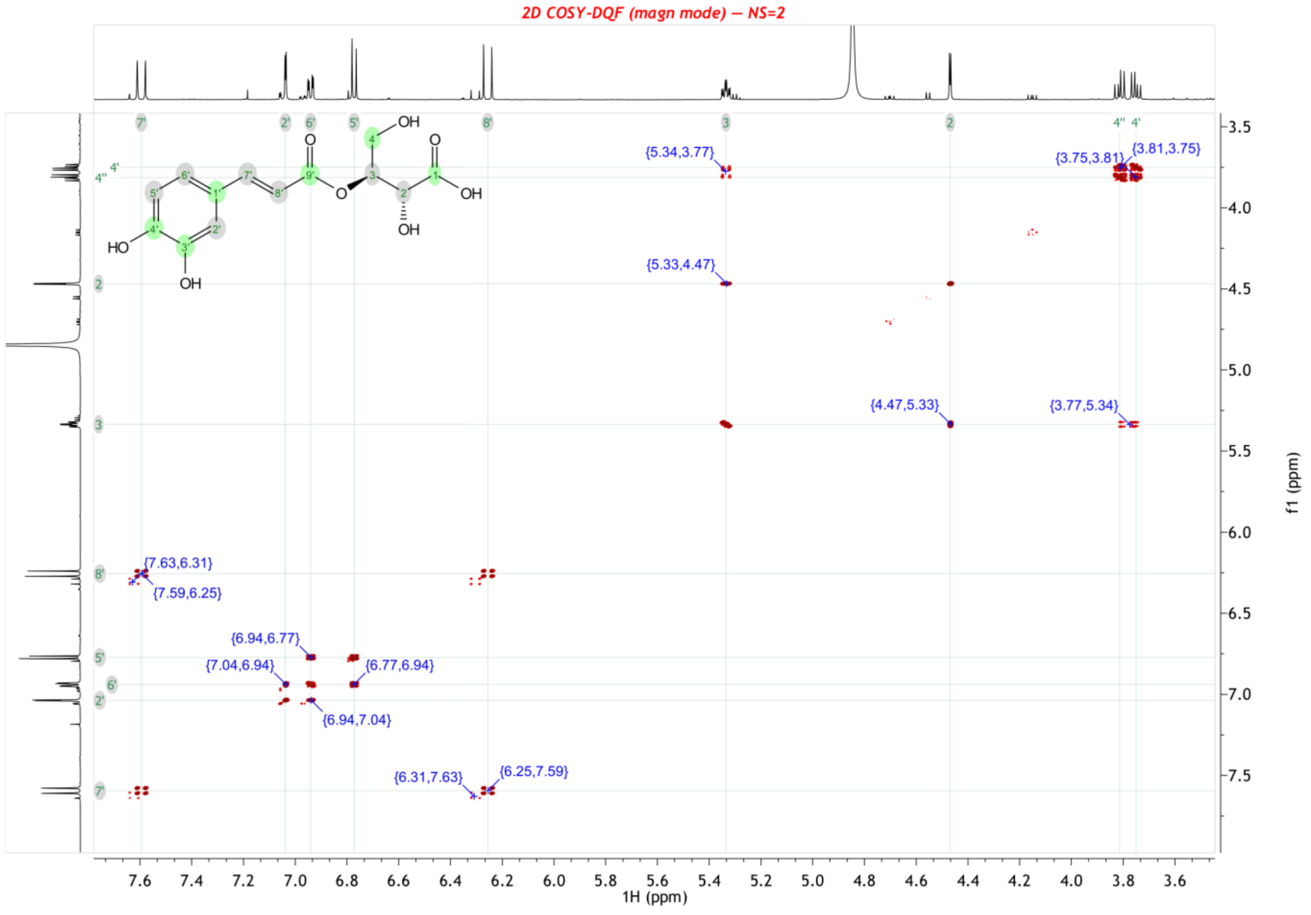

Figure 6S. ^{1}H-^{1}H COSY NMR spectrum of compound 3

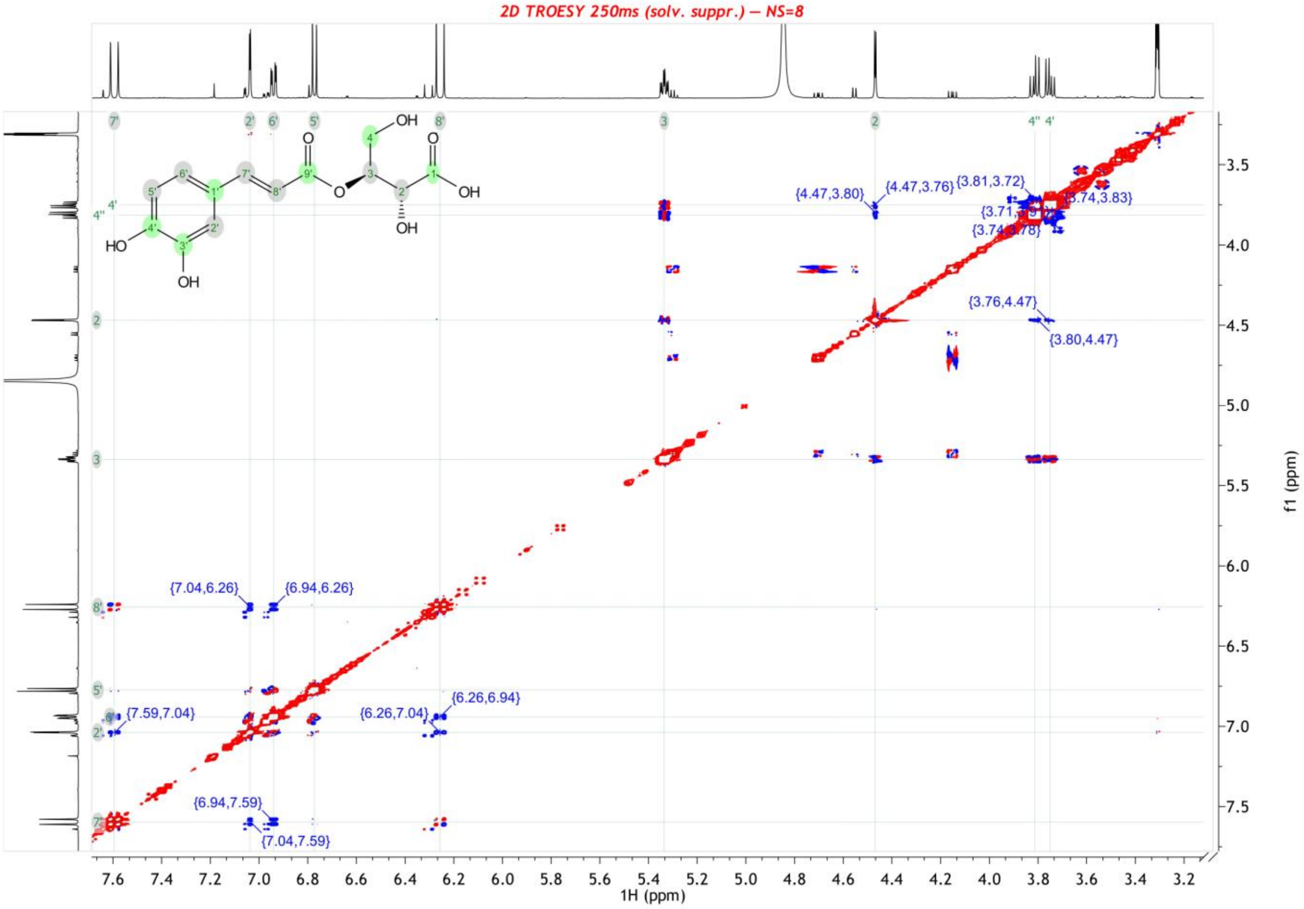

Figure 7S. ^{1}H-^{1}H TROESY (250 ms) NMR spectrum of compound 3

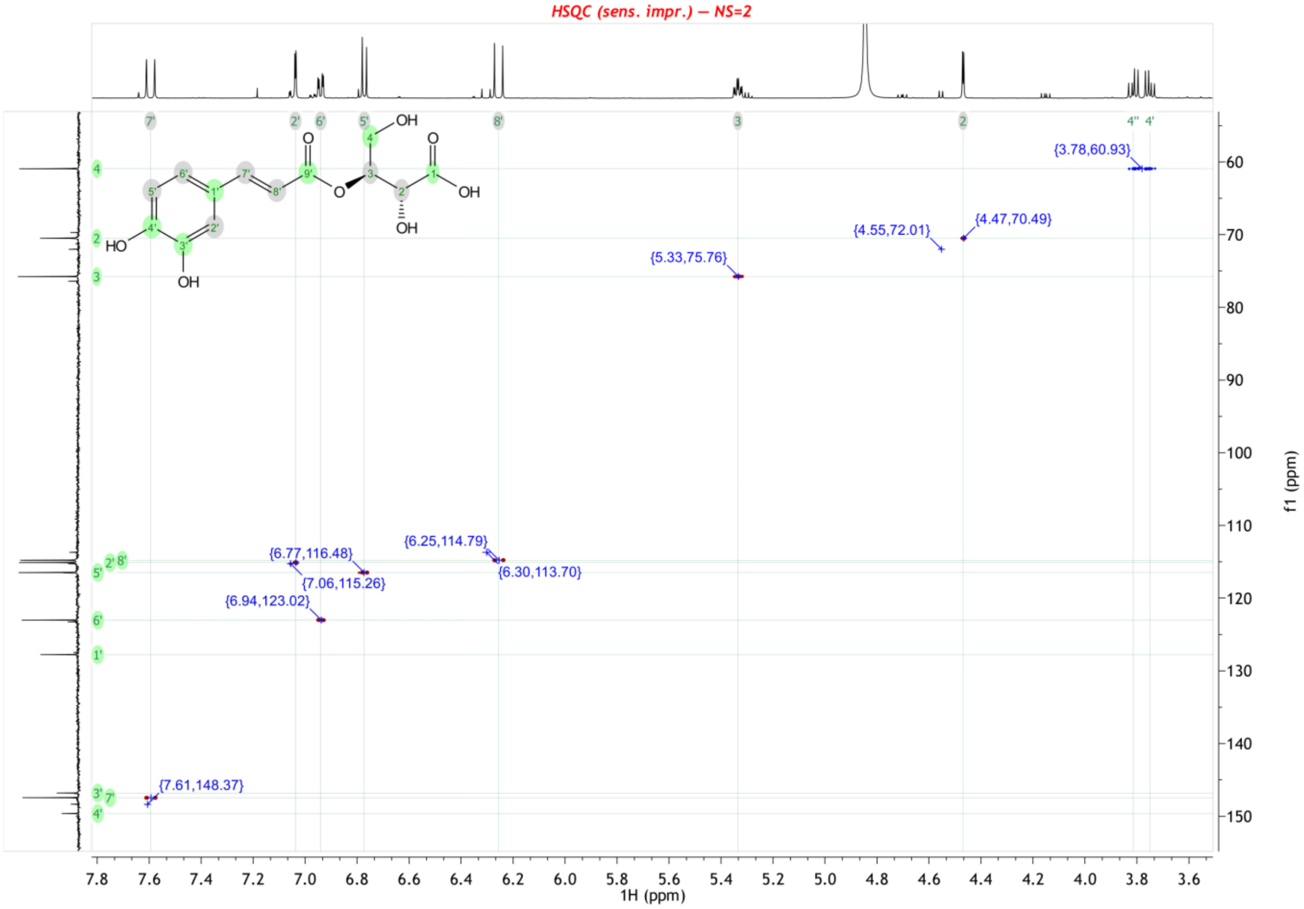

Figure 8S. ^{1}H-^{13}C HSQC NMR spectrum of compound 3

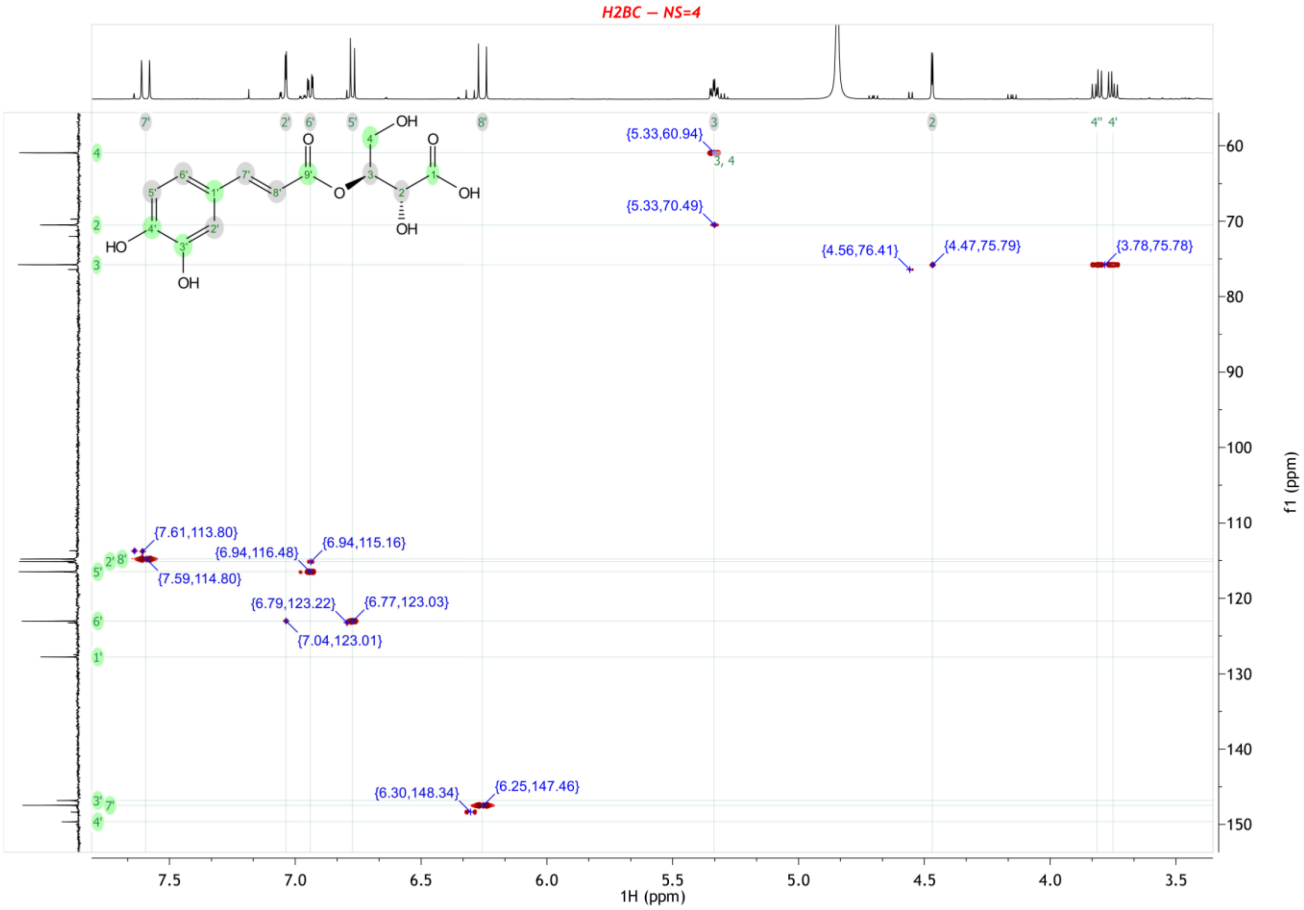

Figure 9S. ^{1}H-^{13}C H2BC NMR spectrum of compound 3

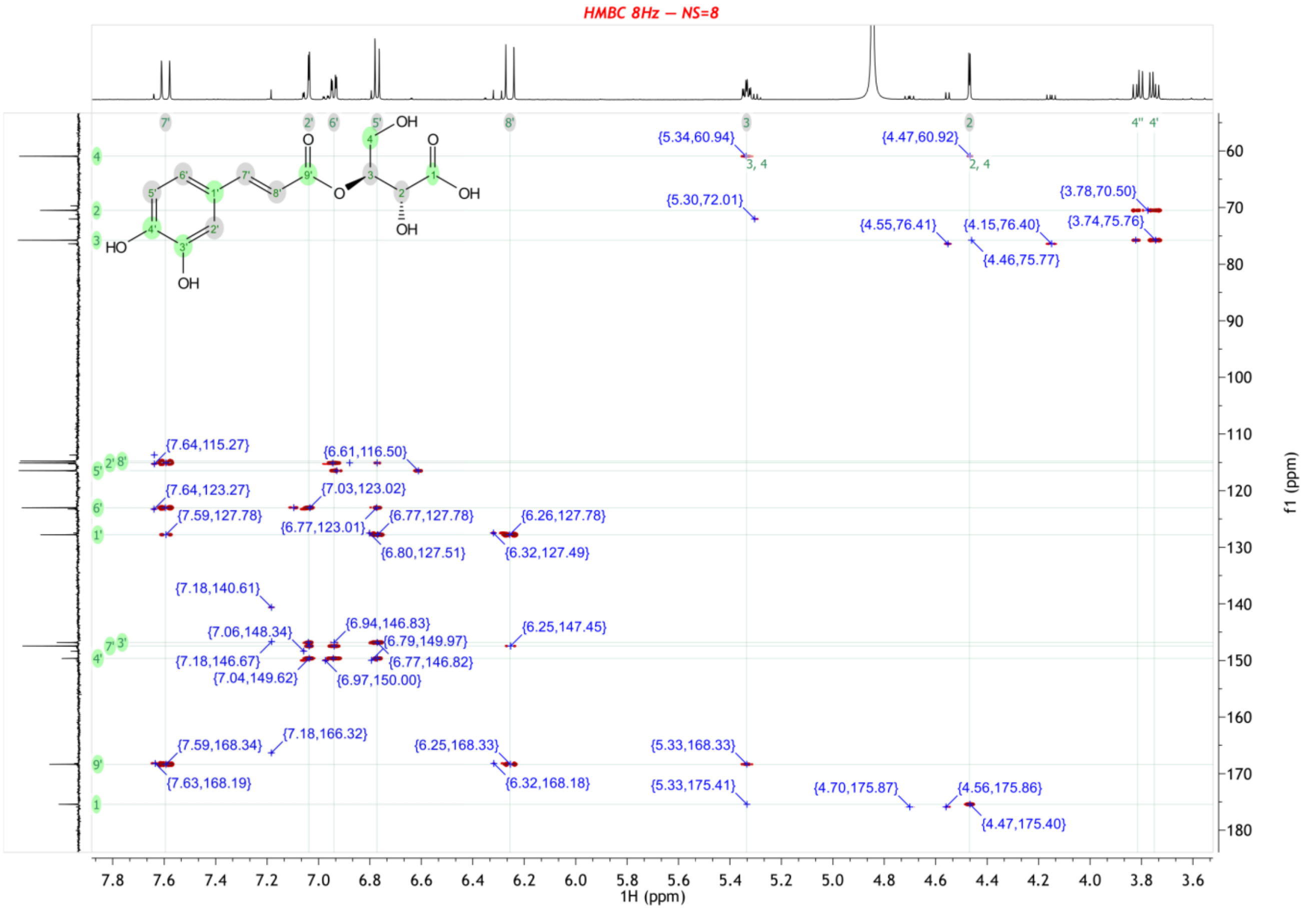

Figure 10S. ^{1}H-^{13}C HMBC (8 Hz) NMR spectrum of compound 3

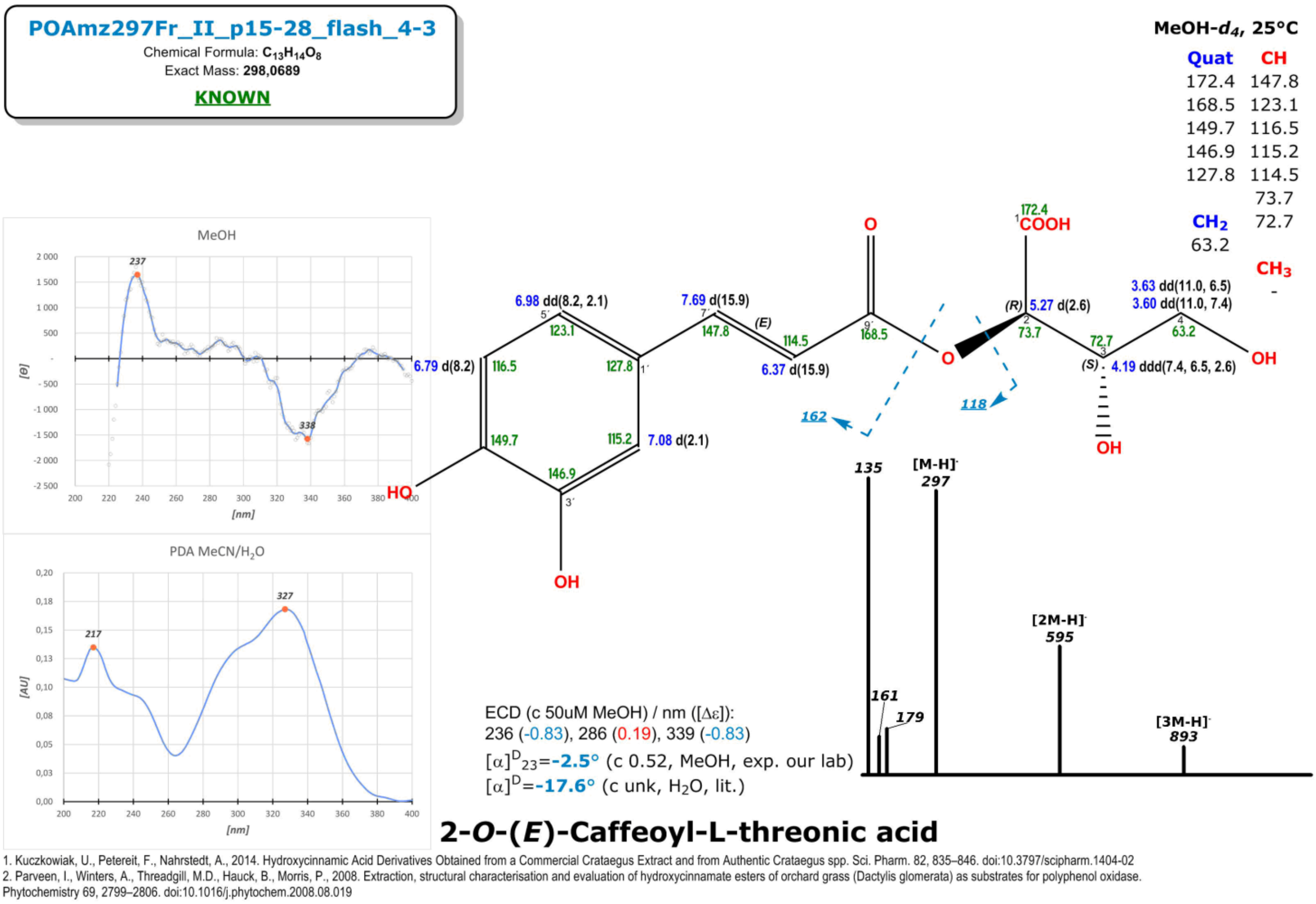

Figure 11S. ^{1}H (500 MHz) and ^{13}C (125 MHz) NMR data of compound 4 in CD_3OD, 25°C; on-line PDA UV spectrum in $MeCN/H_2O$, ECD spectrum in MeOH

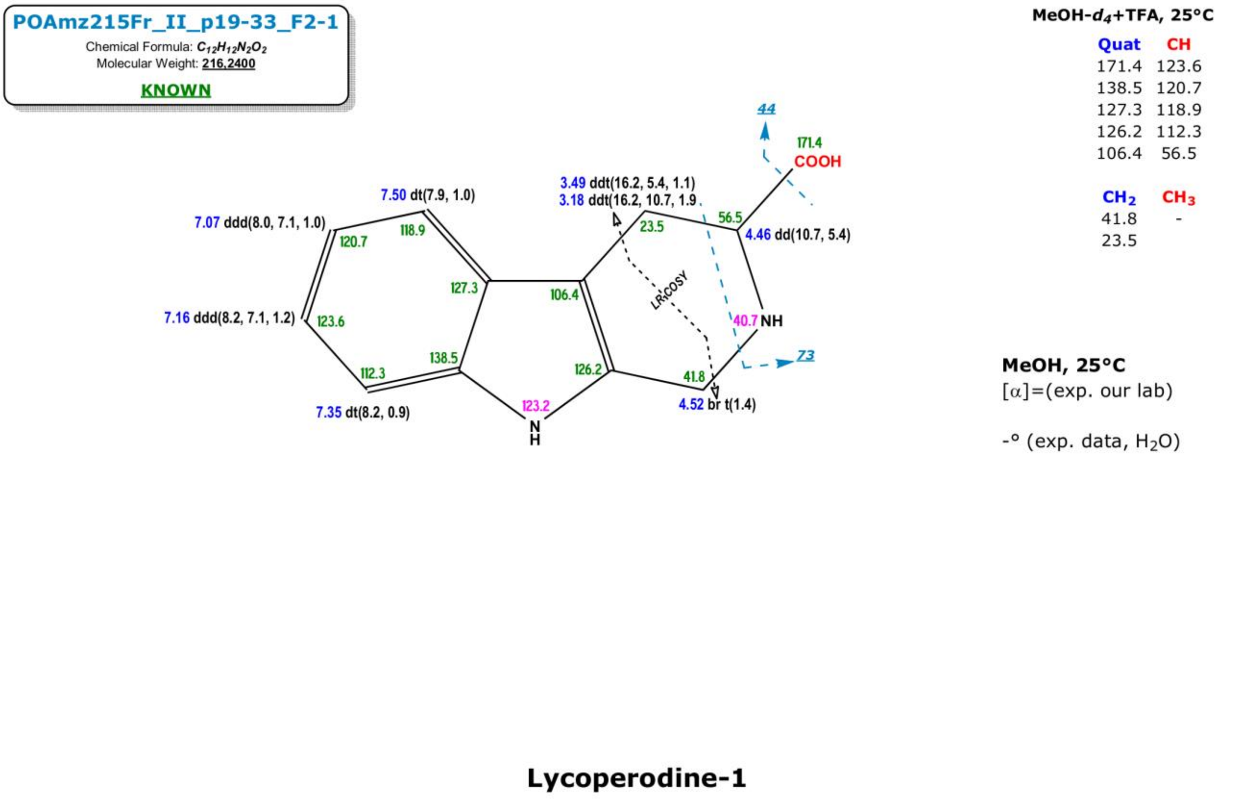

Figure 12S. ^{1}H (500 MHz) ^{13}C (125 MHz) and ^{15}N (50 MHz) NMR data of compound 5 in CD_3OD, 25°C

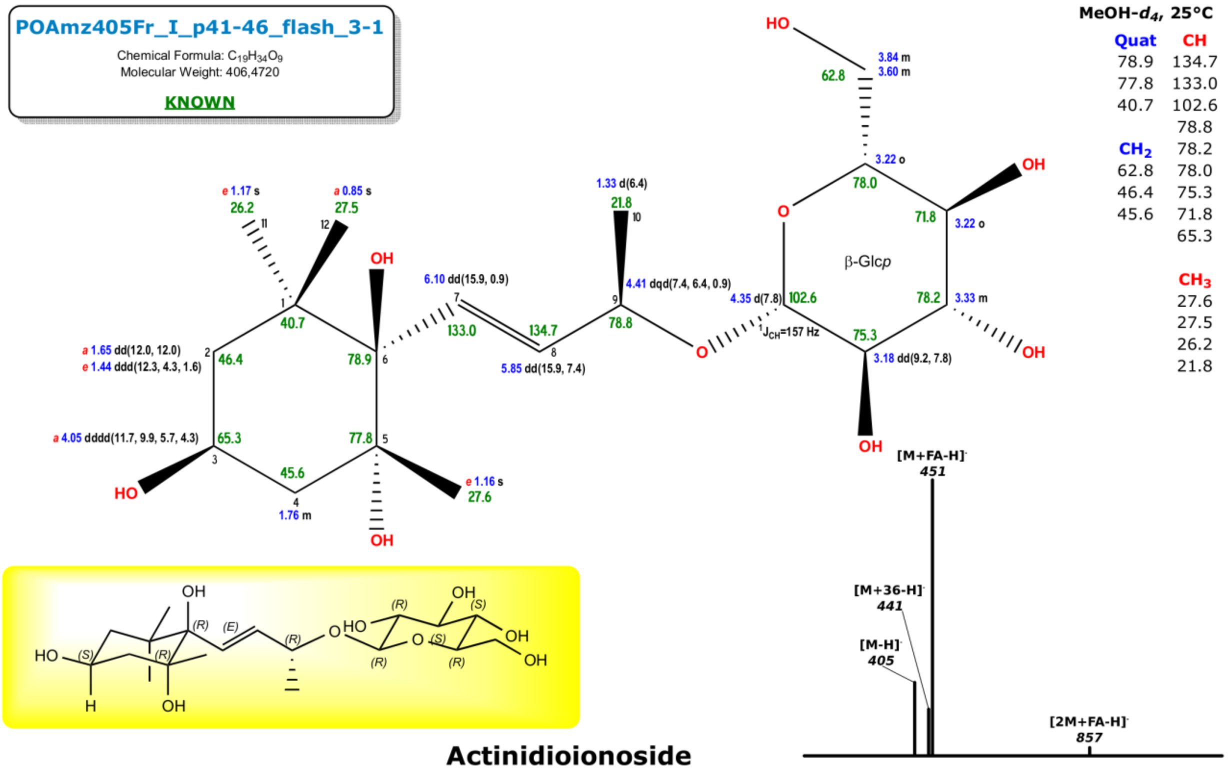

1. Murai, F., Tagawa, M., 1989. Relationship between ionone glycosides and terpenoids in Actinidia polygama, in: Abstract Papers of the 33rd Symposium on the Chemistry of Terpenes, Essential Oils, and Aromatics (TEAC), Sendai. pp. 68–70.
2. Otsuka, H., Hirata, E., Shinzato, T., Takeda, Y., 2003. Stereochemistry of megastigmane glucosides from Glochidion zeylanicum and Alangium premnifolium. Phytochemistry 62, 763–768. doi:10.1016/S0031-9422(02)00614-3

Figure 13S. ^{1}H (500 MHz) and ^{13}C (125 MHz) NMR data of compound 7 in CD_3OD, 25°C

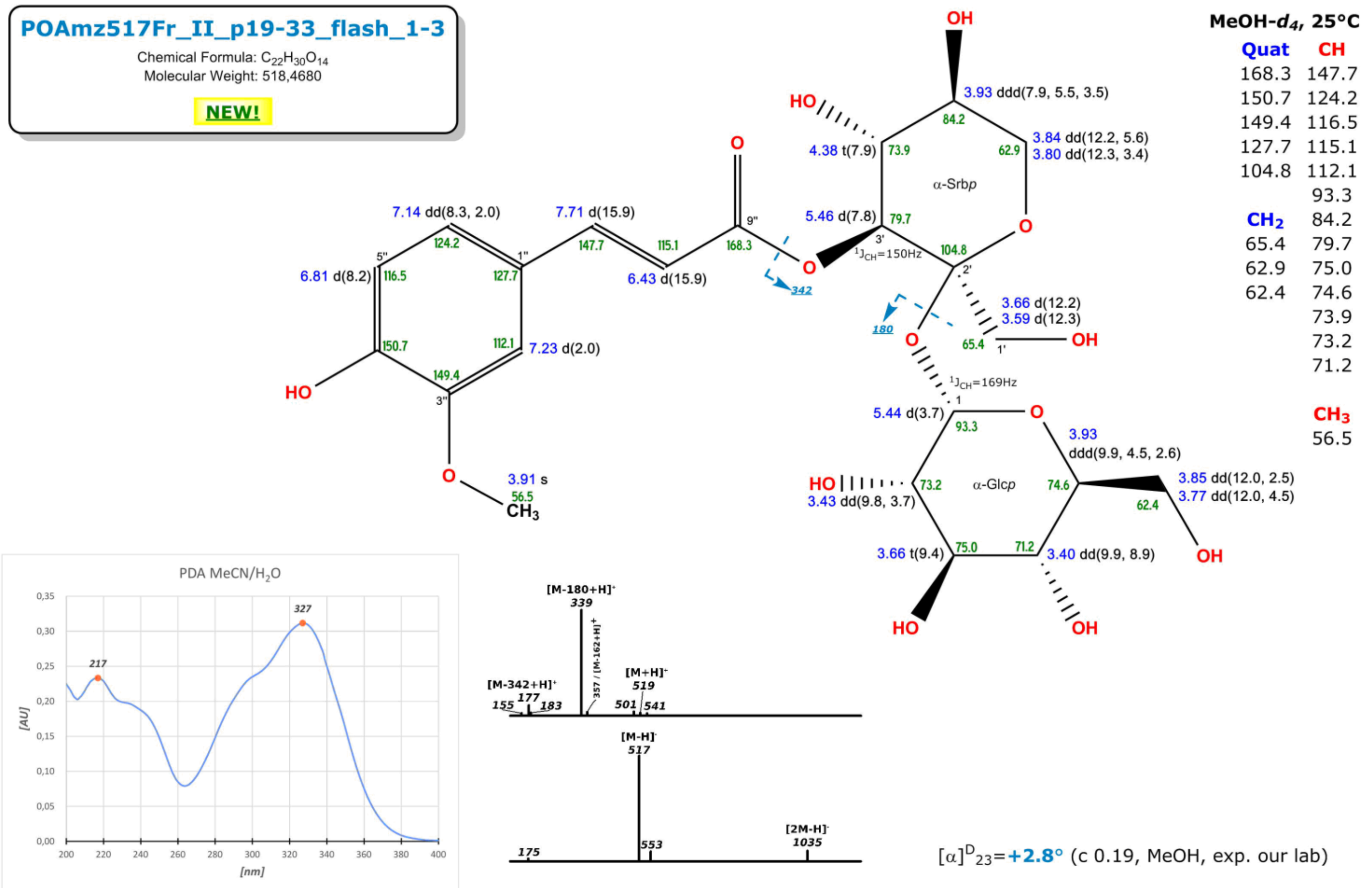

3'-O-(E)-Feruoyl-α-sorbopyranosyl-(2'→1)-α-glucopyranoside

Figure 14S. ^{1}H (500 MHz) and ^{13}C (125 MHz) NMR data of compound 10 in CD_3OD, 25°C; on-line PDA UV spectrum in $MeCN/H_2O$

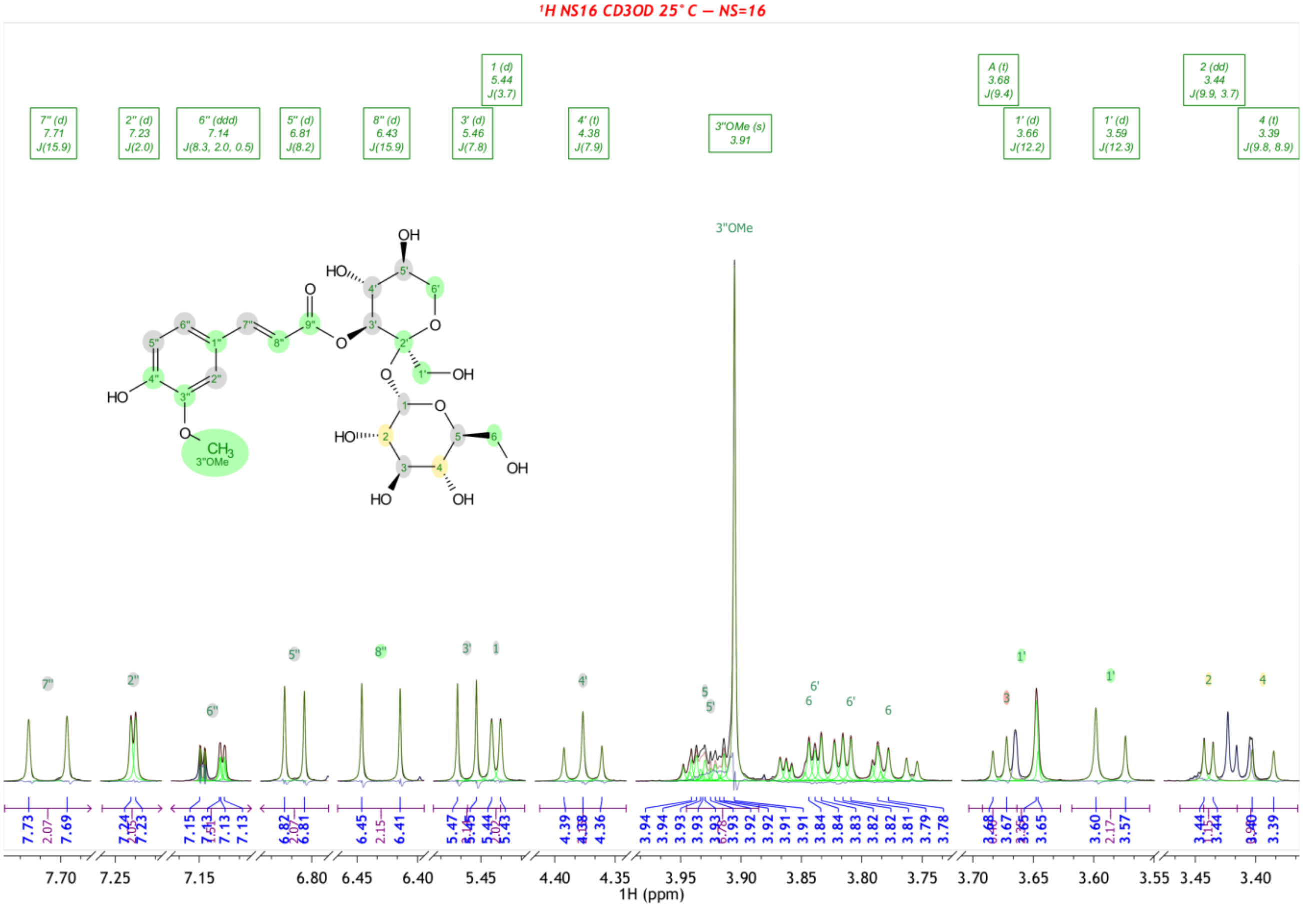

Figure 15S. ^{1}H NMR spectrum of compound 10

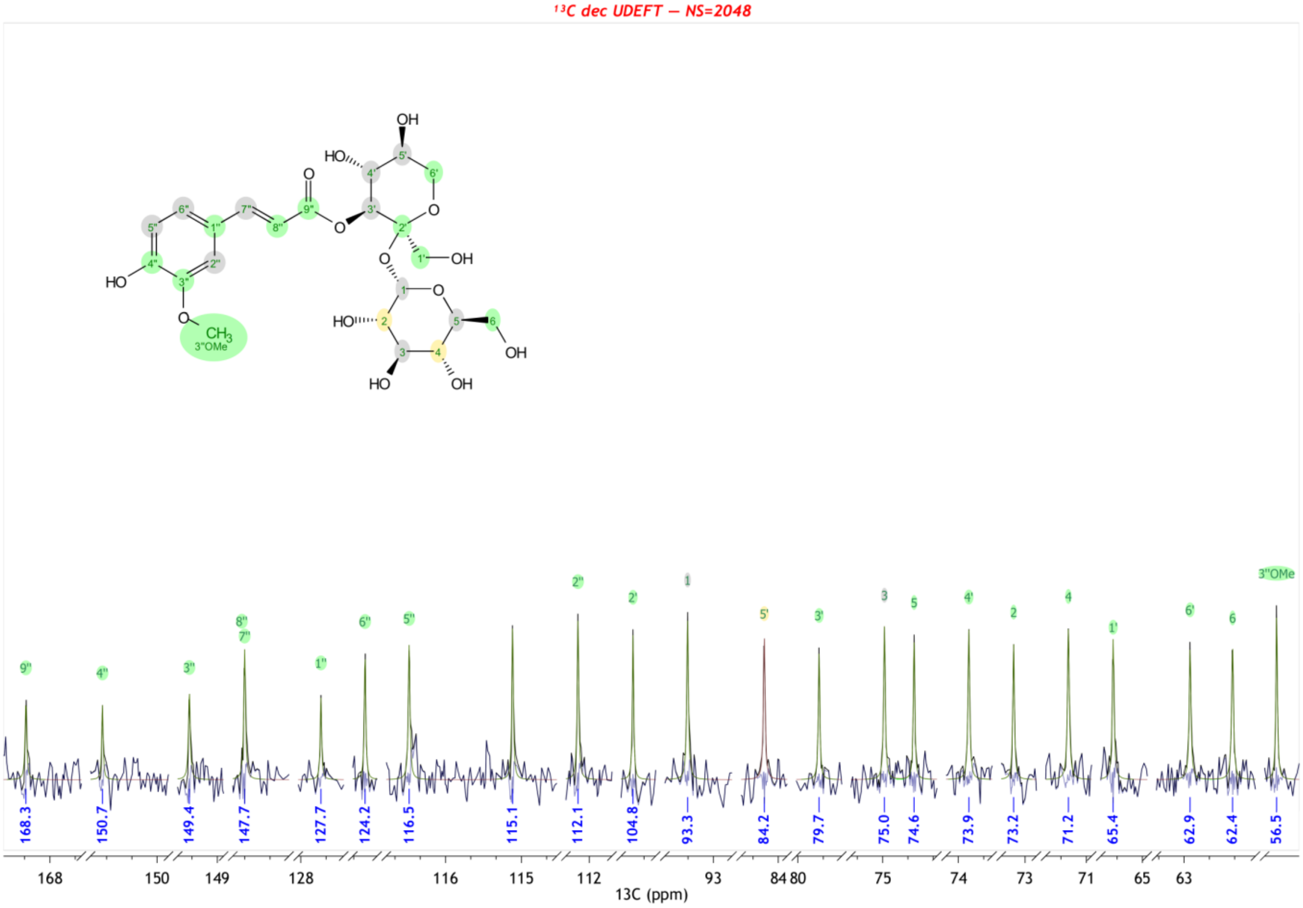

Figure 16S. ^{13}C DEPTQ NMR spectrum of compound 10

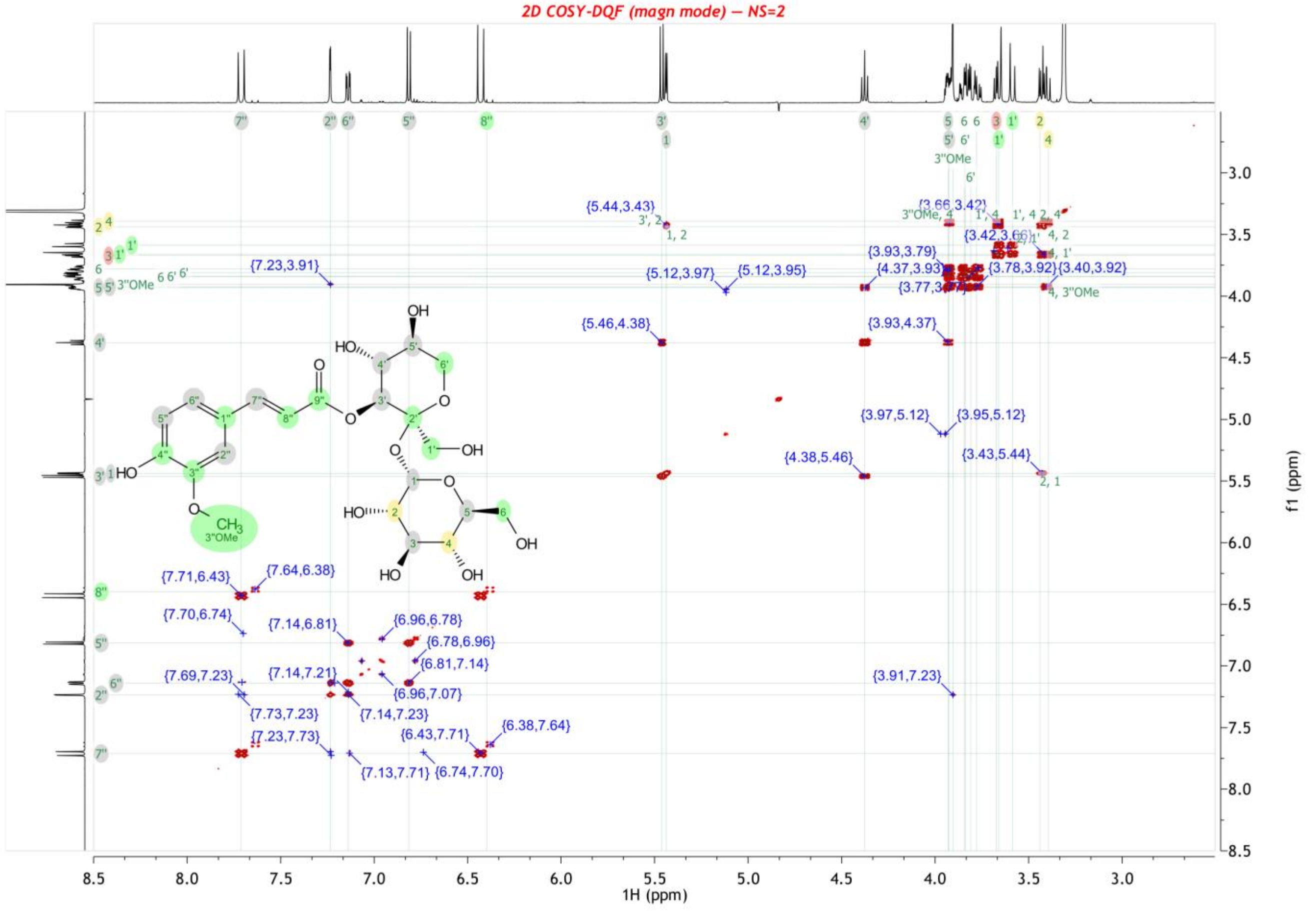

Figure 17S. ^{1}H-^{1}H COSY NMR spectrum of compound 10

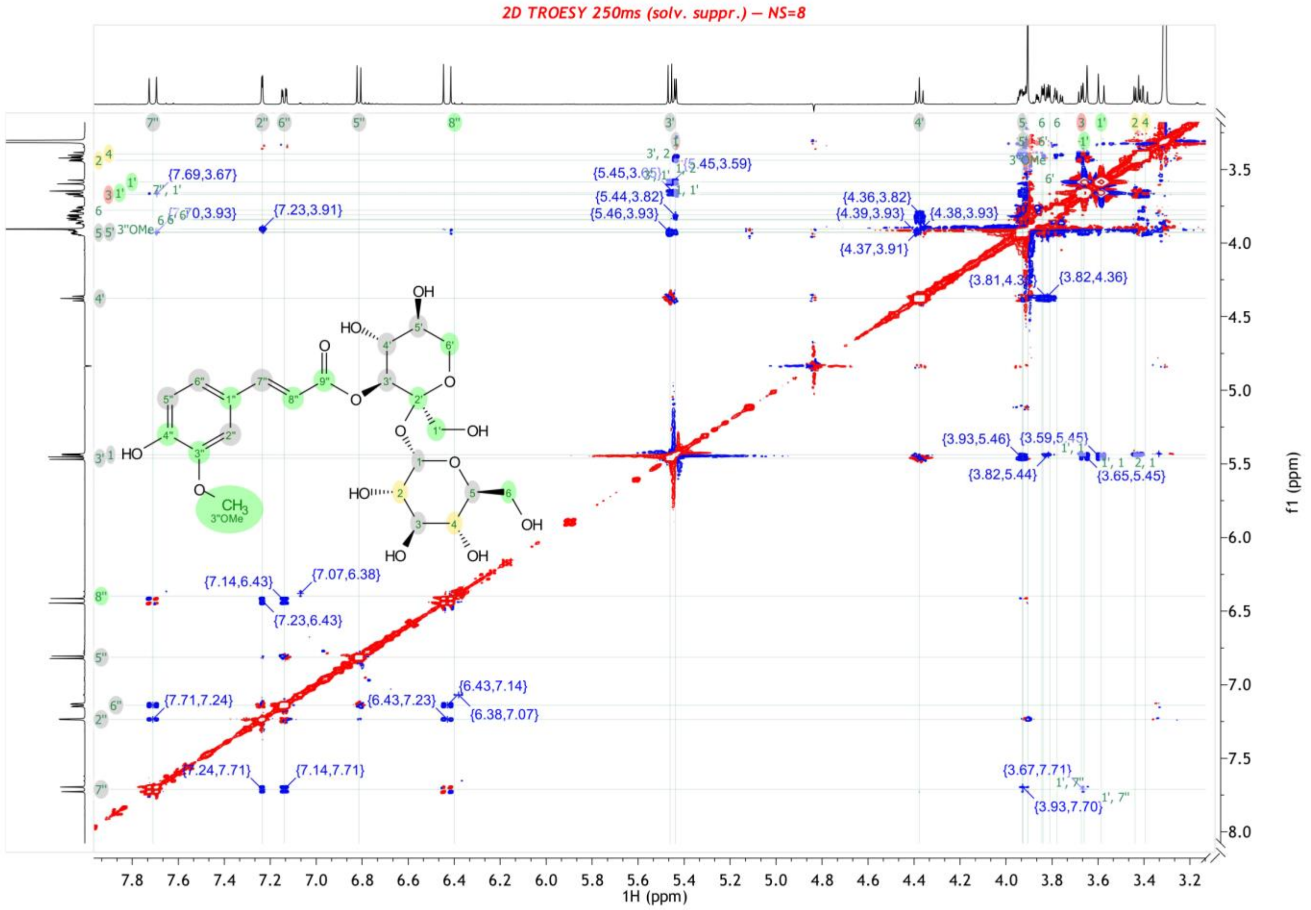

Figure 18S. ^{1}H-^{1}H TROESY (250 ms) NMR spectrum of compound 10

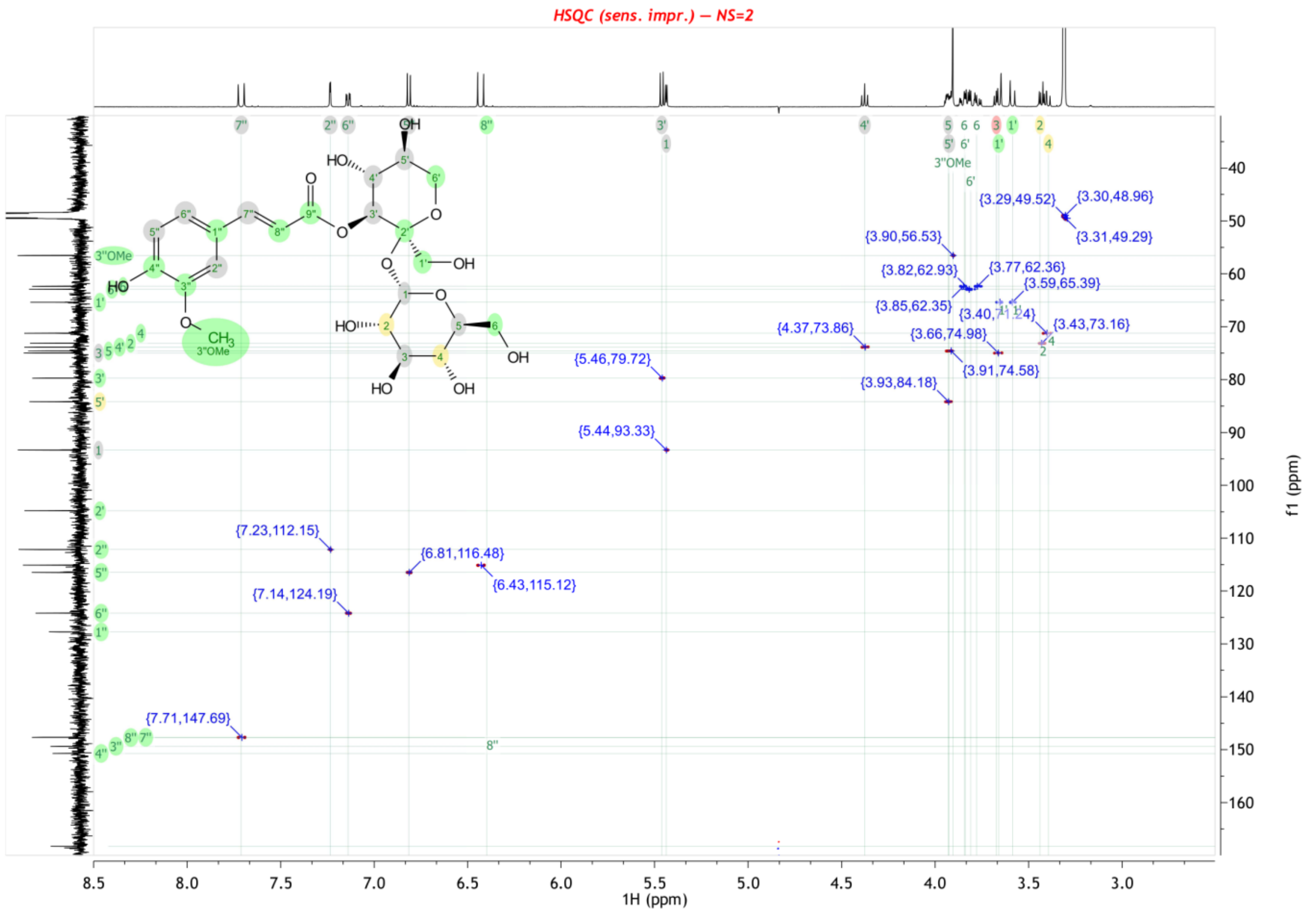

Figure 19S. ^{1}H-^{13}C HSQC NMR spectrum of compound 10

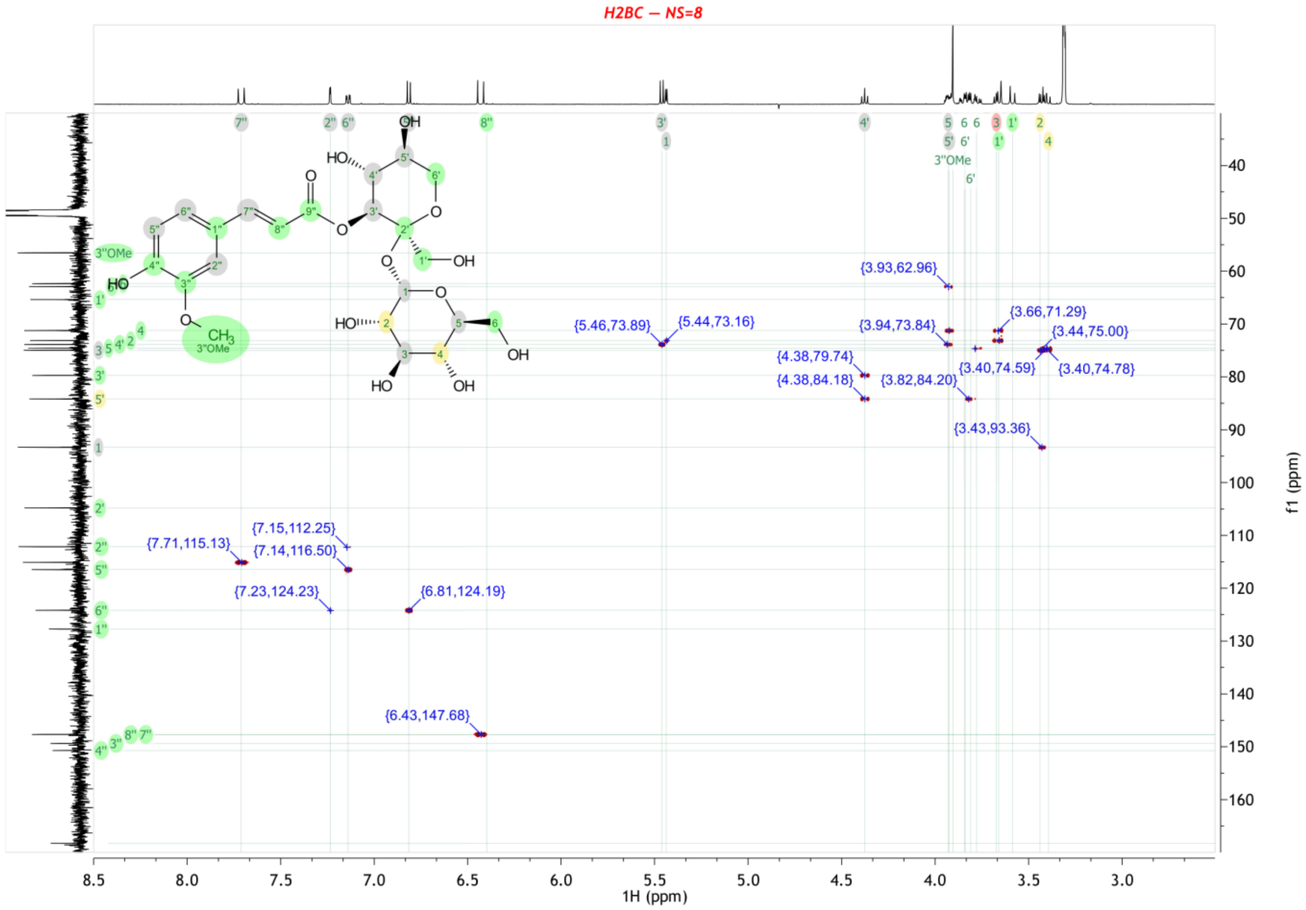

Figure 20S. ^{1}H-^{13}C H2BC NMR spectrum of compound 10

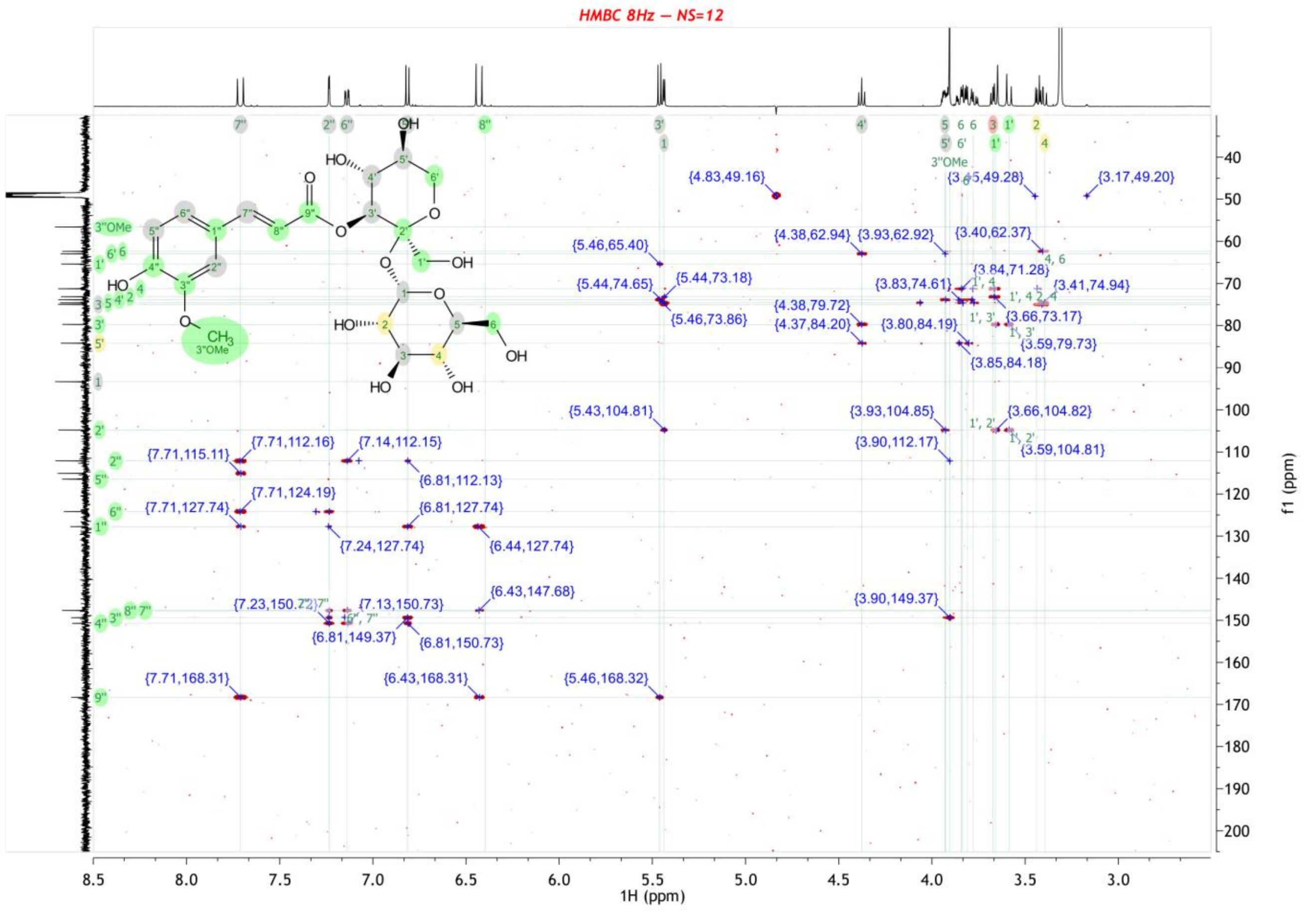

Figure 21S. ^{1}H-^{13}C HMBC (8 Hz) NMR spectrum of compound 10

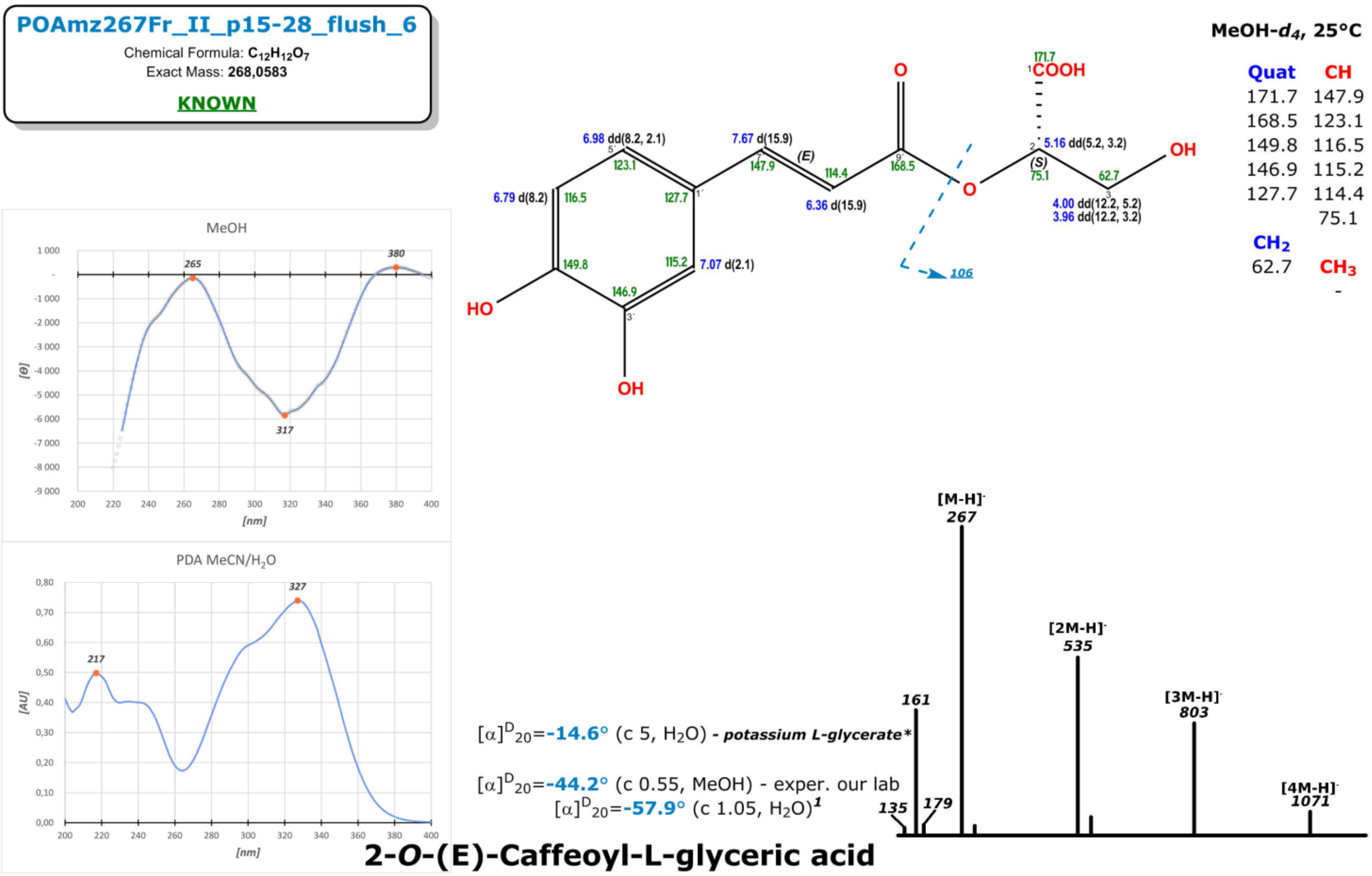

1. Hahn, R., Nahrstedt, A., 1993. Hydroxycinnamic acid derivatives, caffeoylmalic and new caffeoylaldonic acid esters, from Chelidonium majus. Planta Med. 59, 71–75. doi:10.1055/s-2006-959608
* http://www.drugfuture.com/chemdata/glyceric-acid.html

Figure 22S. ^{1}H (500 MHz) and ^{13}C (125 MHz) NMR data of compound 11 in CD_3OD, 25°C; on-line PDA UV spectrum in $MeCN/H_2O$; ECD spectrum in MeOH

POAmz297Fr_II_p19-34_flash_2-p23

Chemical Formula: $C_{13}H_{14}O_8$
Exact Mass: 298,0689

KNOWN

MeOH-d_4, 25°C

Quat	CH
175.9	147.2
169.0	123.0
149.6	116.5
146.8	115.2
127.7	114.8
	72.3
	71.4

CH2	CH3
66.0	-

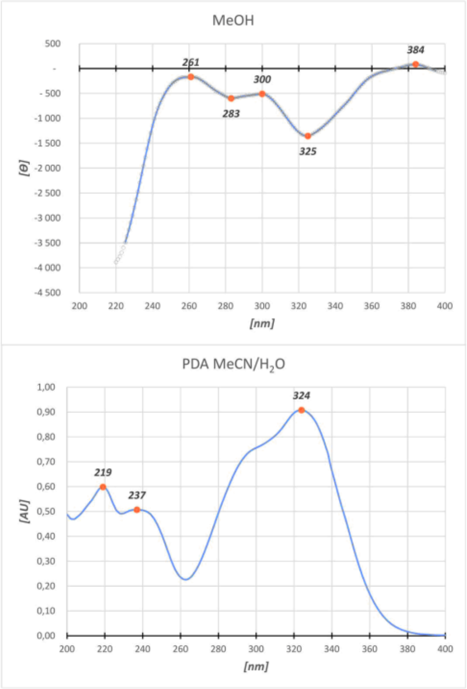

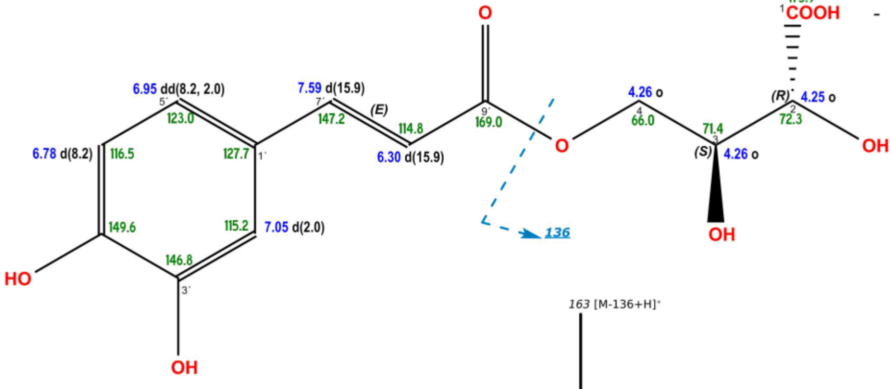

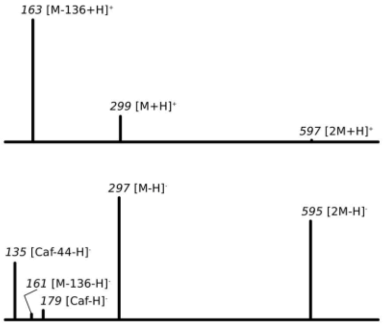

[α]$^D_{23}$=**-16.4°** (c 0.15, MeOH, exp. our lab)

4-*O*-(*E*)-Caffeoyl-L-threonic acid

Hahn, R., Nahrstedt, A., 1993. Hydroxycinnamic acid derivatives, caffeoylmalic and new caffeoylaldonic acid esters, from Chelidonium majus. Planta Med. 59, 71–75. doi:10.1055/s-2006-959608

Figure 23S. ^{1}H (500 MHz) and ^{13}C (125 MHz) NMR data of compound 12 in CD_3OD, 25°C; on-line PDA UV spectrum in MeCN/H_2O; ECD spectrum in MeOH

POAmz267Fr_II_p19-33_2-3-1

Chemical Formula: $C_{12}H_{12}O_7$
Exact Mass: 268,0583

NEW!

MeOH-d_4, 25°C

Quat	CH
174.8	147.4
168.9	123.0
149.7	116.5
146.8	115.1
127.7	114.6
	70.3

CH2	CH3
66.9	-

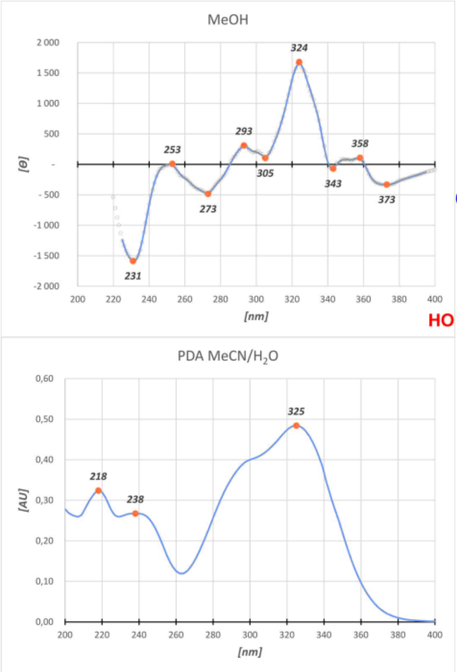

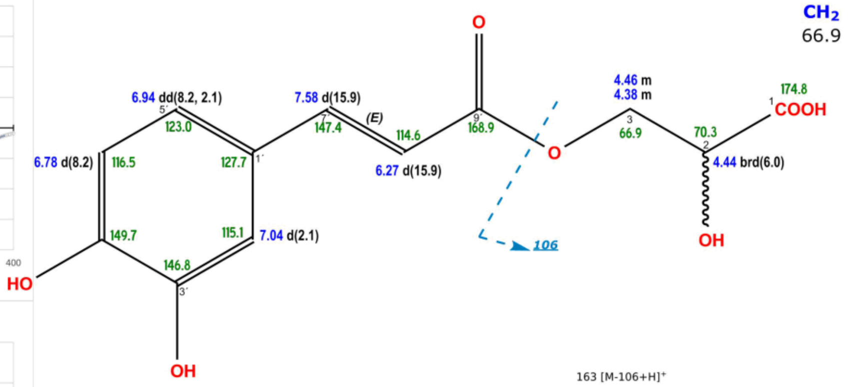

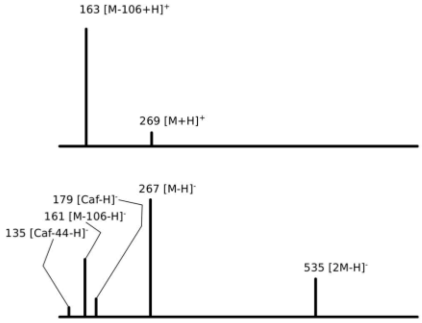

[α]$^D_{23}$=**+7.5°** (c 0.21, MeOH) - exp. our lab

3-*O*-(E)-Caffeoyl-glyceric acid

Figure 24S. ^{1}H (500 MHz) and ^{13}C (125 MHz) NMR data of compound 14 in CD_3OD, 25°C; on-line PDA UV spectrum in MeCN/H_2O; ECD spectrum in MeOH

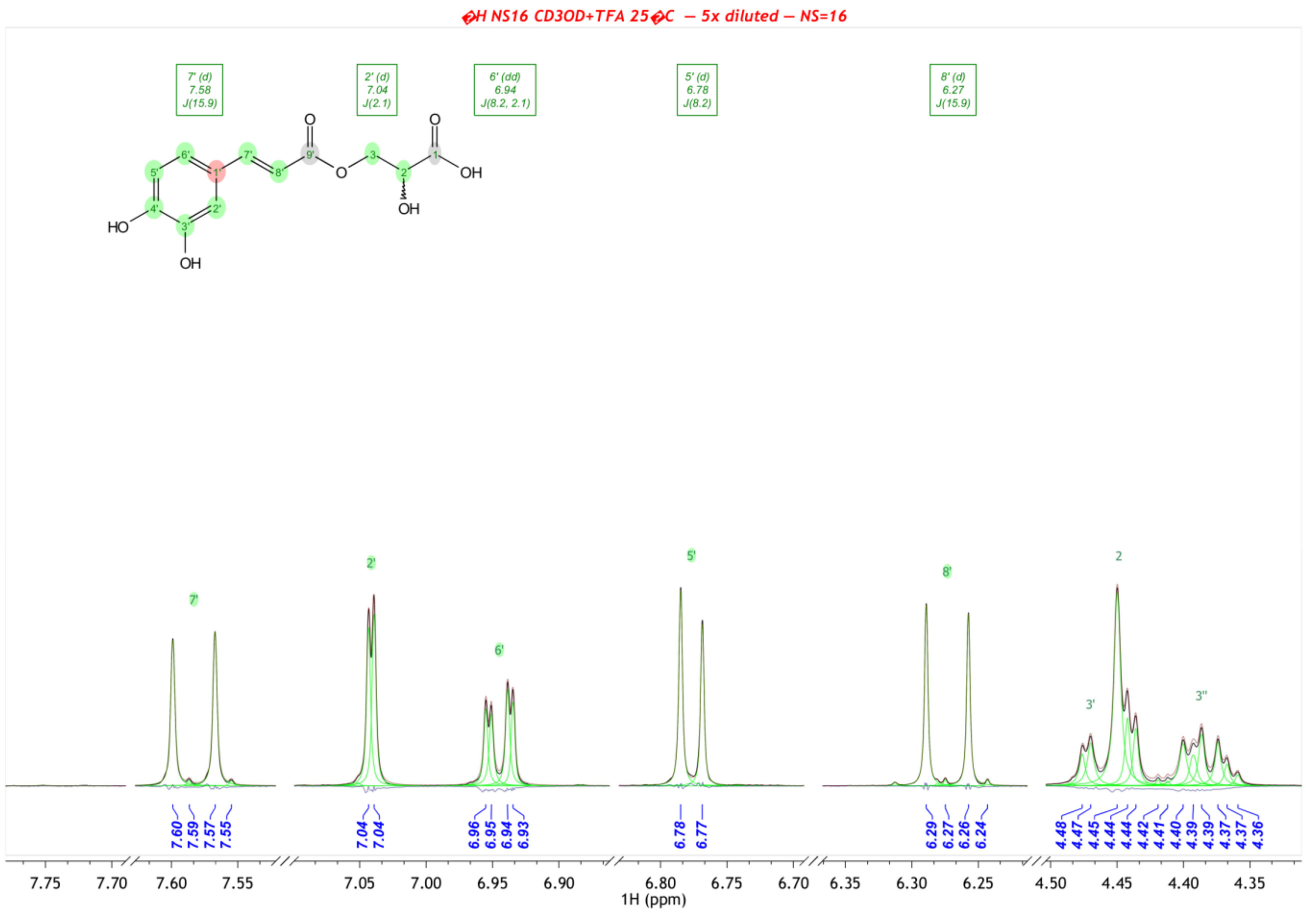

Figure 25S. ^{1}H NMR spectrum of compound 14

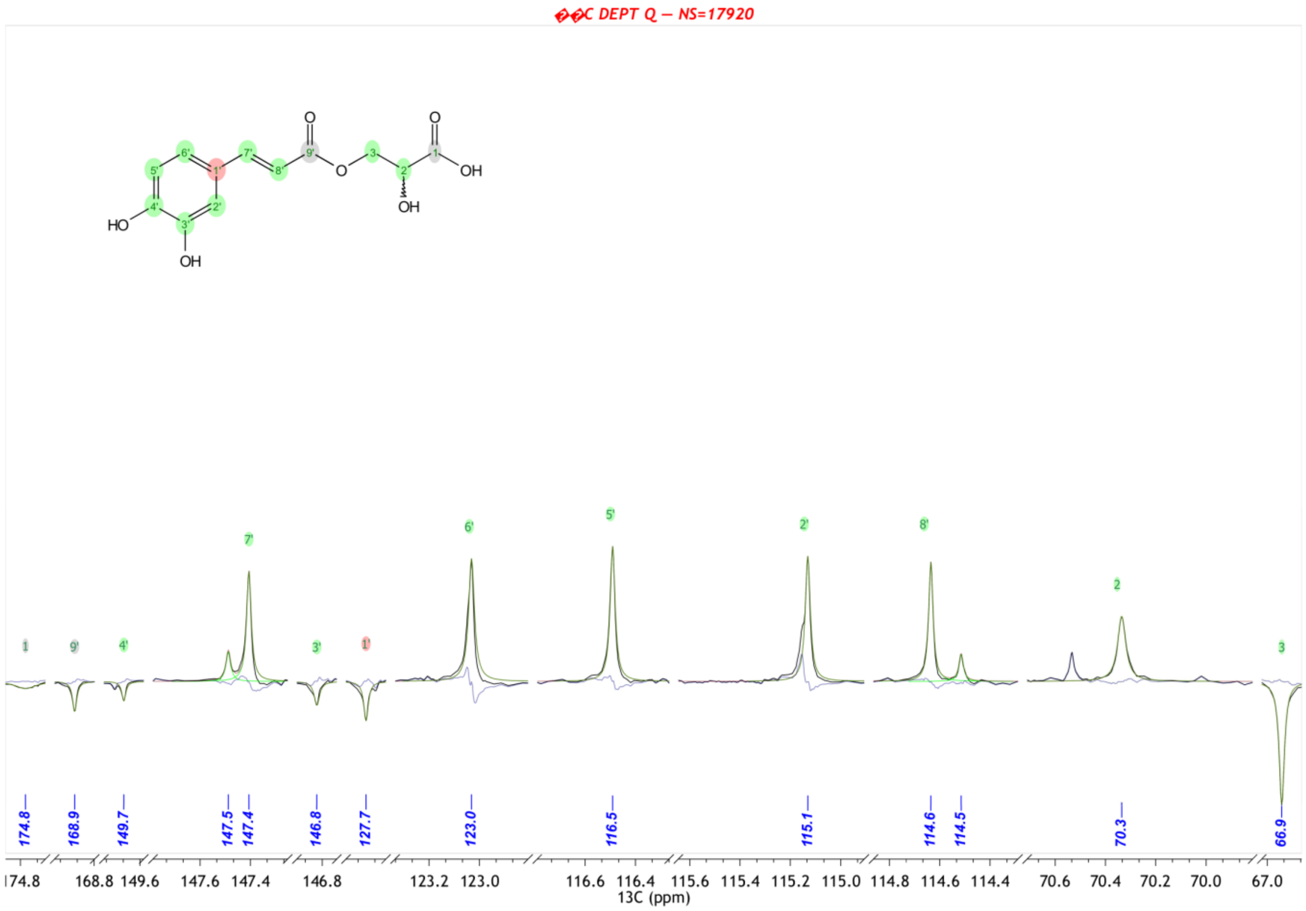

Figure 26S. ^{13}C DEPTQ NMR spectrum of compound 14

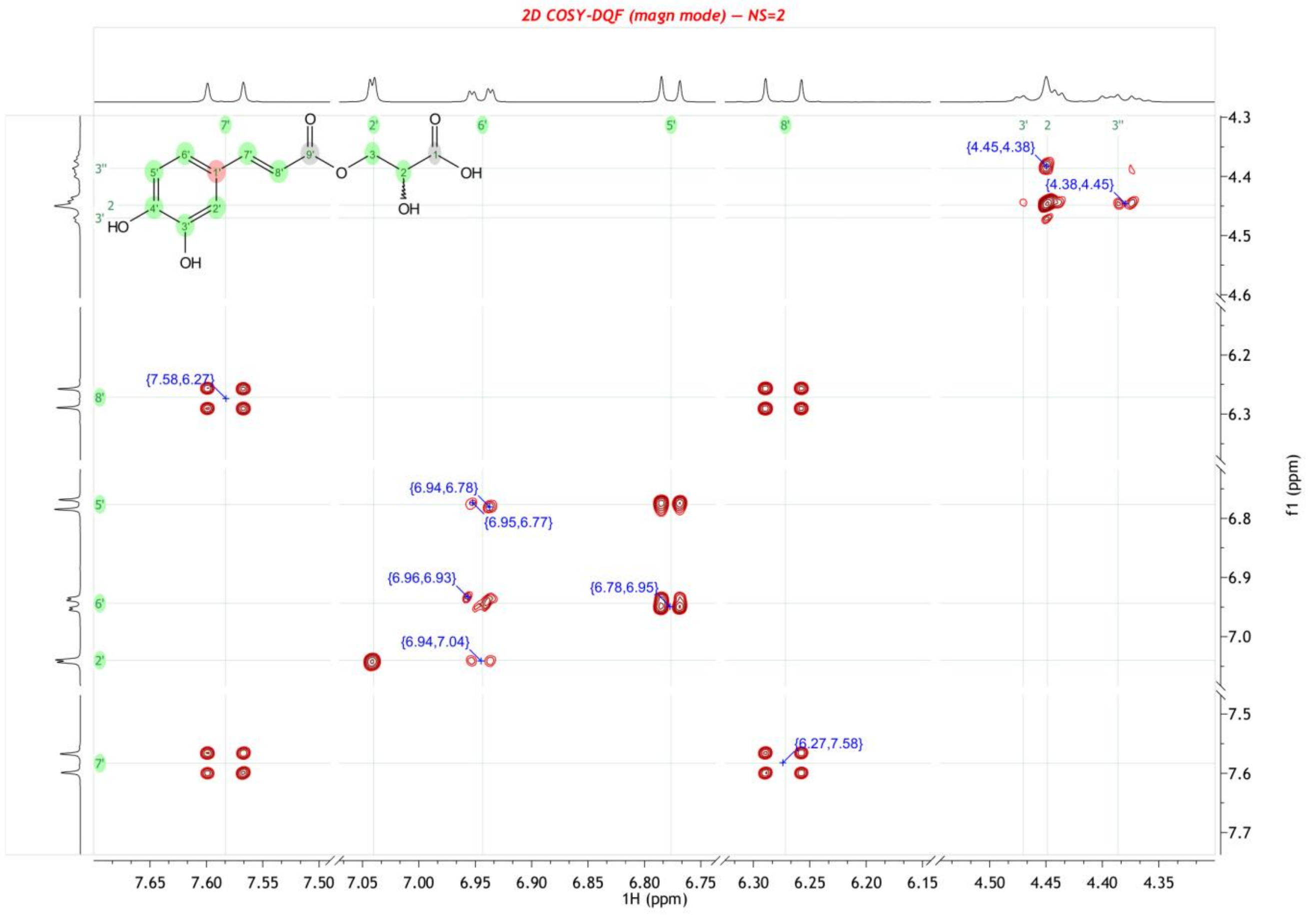

Figure 27S. ^{1}H-^{1}H COSY NMR spectrum of compound 14

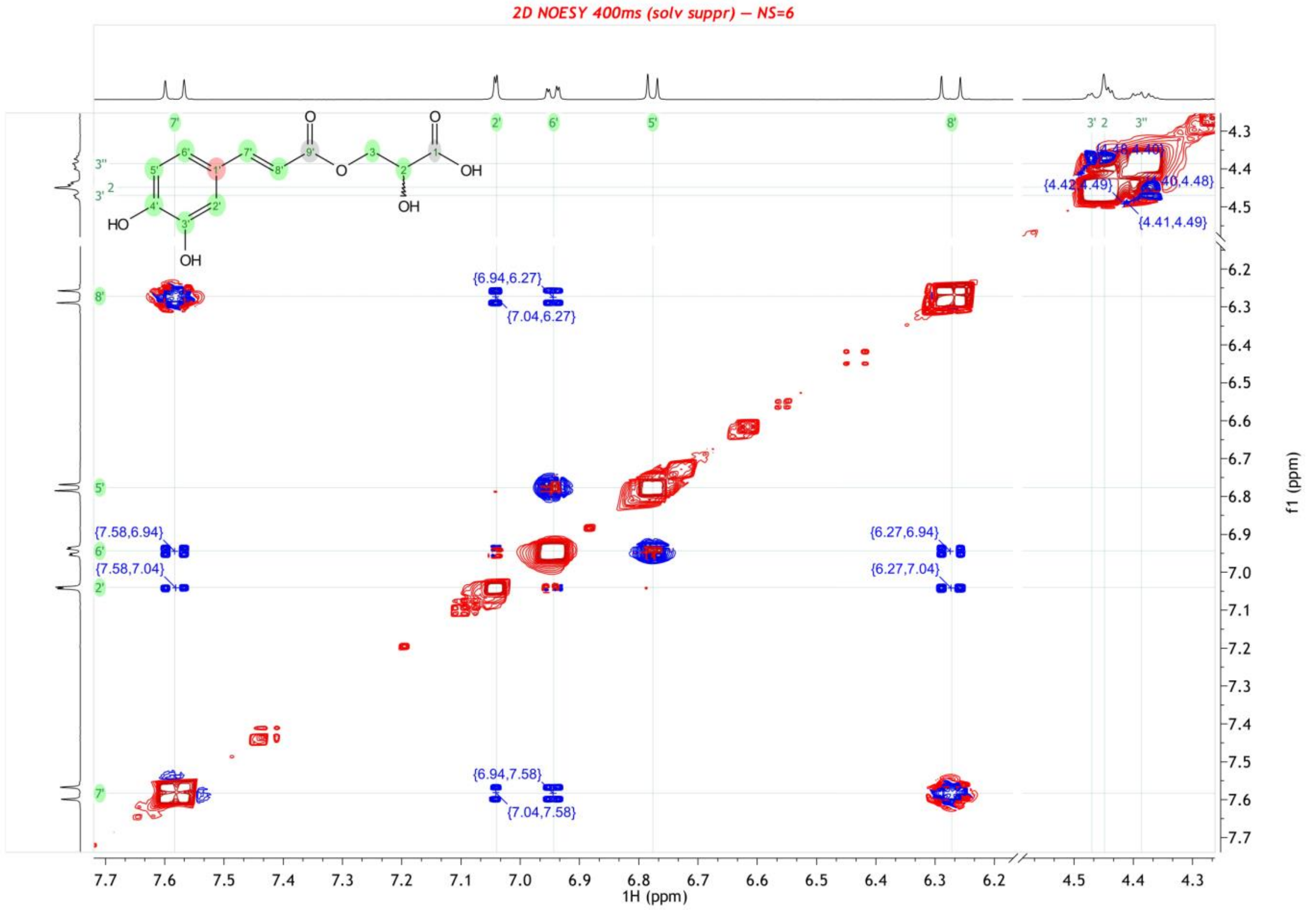

Figure 28S. ^{1}H-^{1}H NOESY (400 ms) NMR spectrum of compound 14

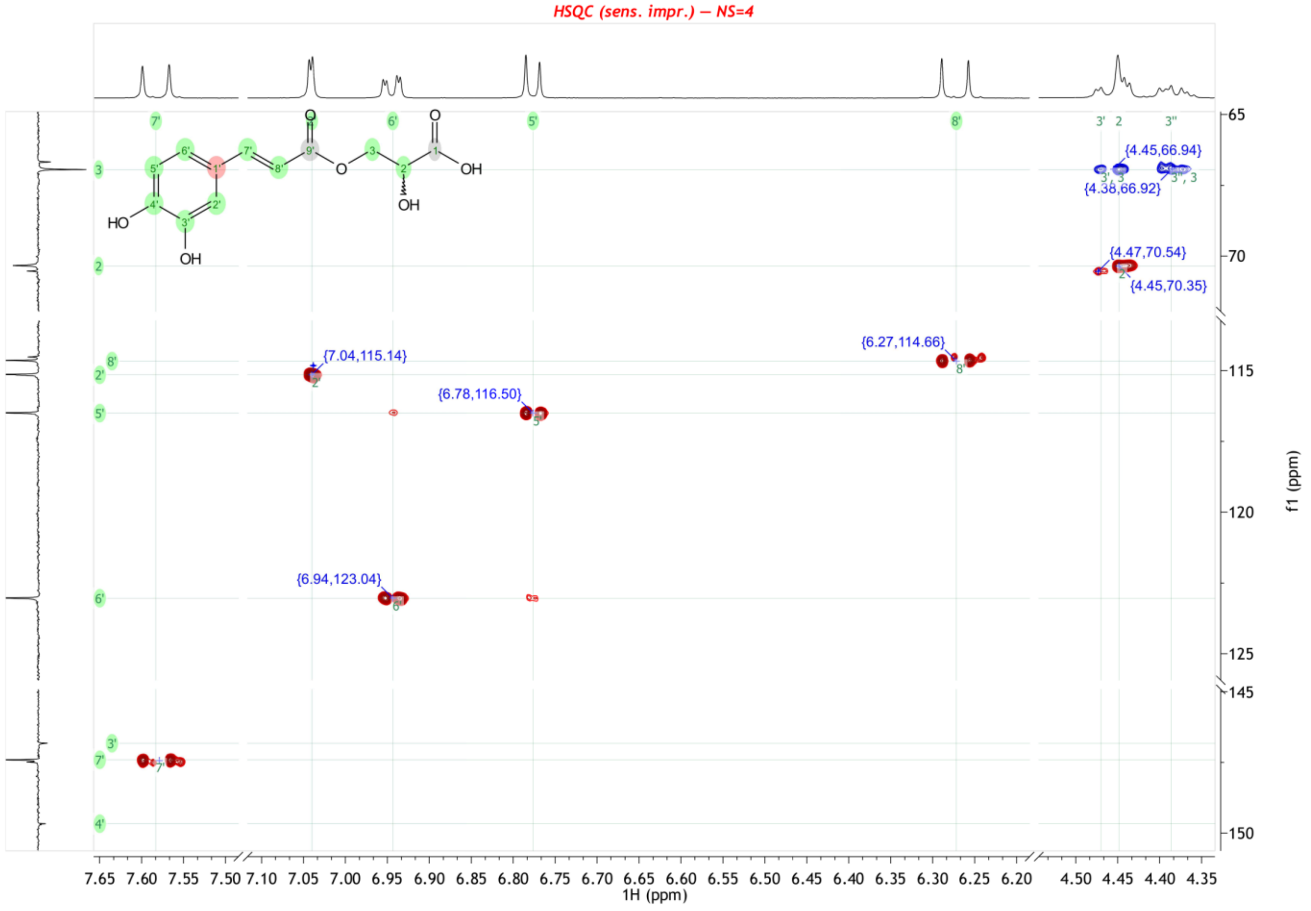

Figure 29S. ^{1}H-^{13}C HSQC NMR spectrum of compound 14

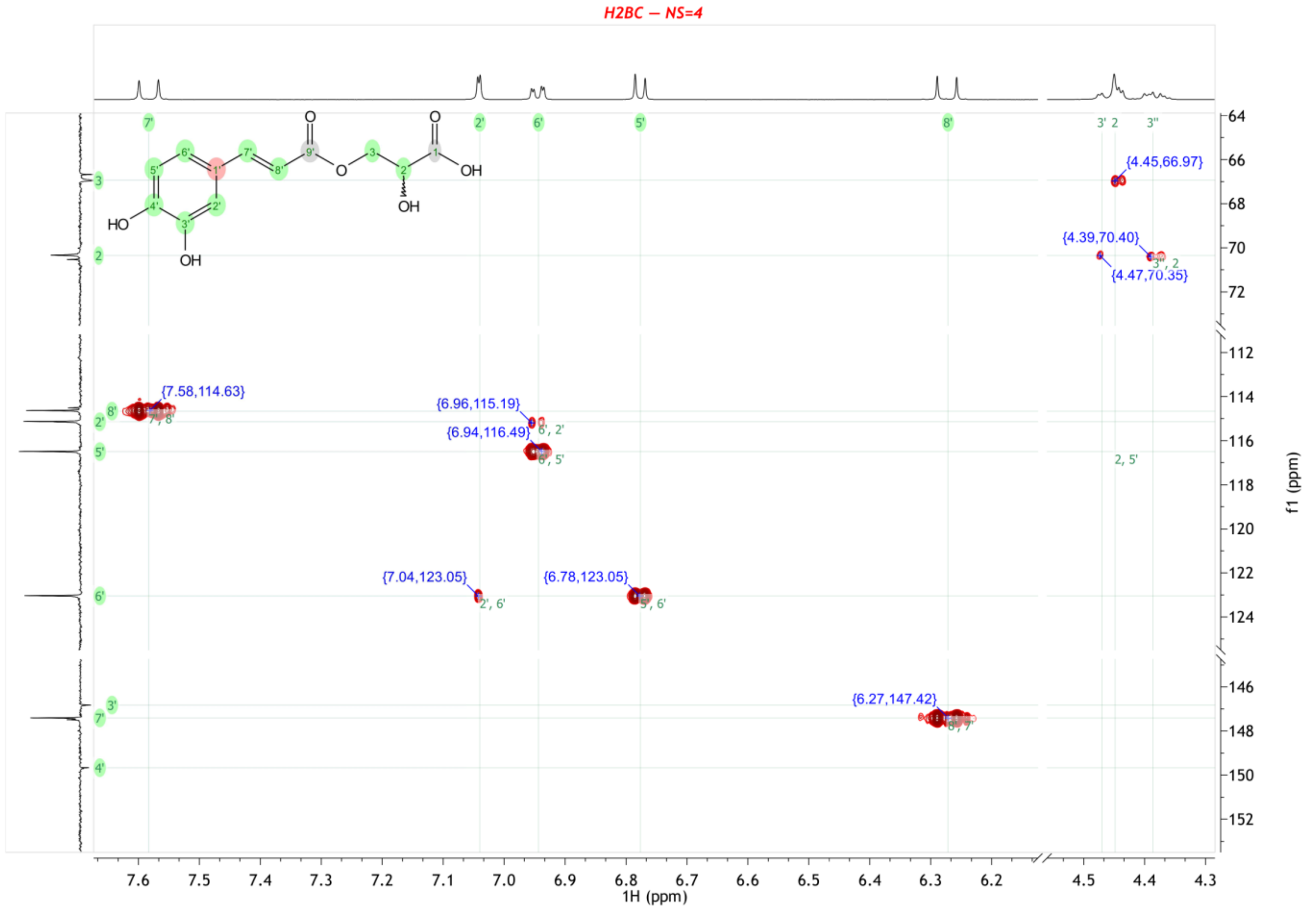

Figure 30S. ^{1}H-^{13}C H2BC NMR spectrum of compound 14

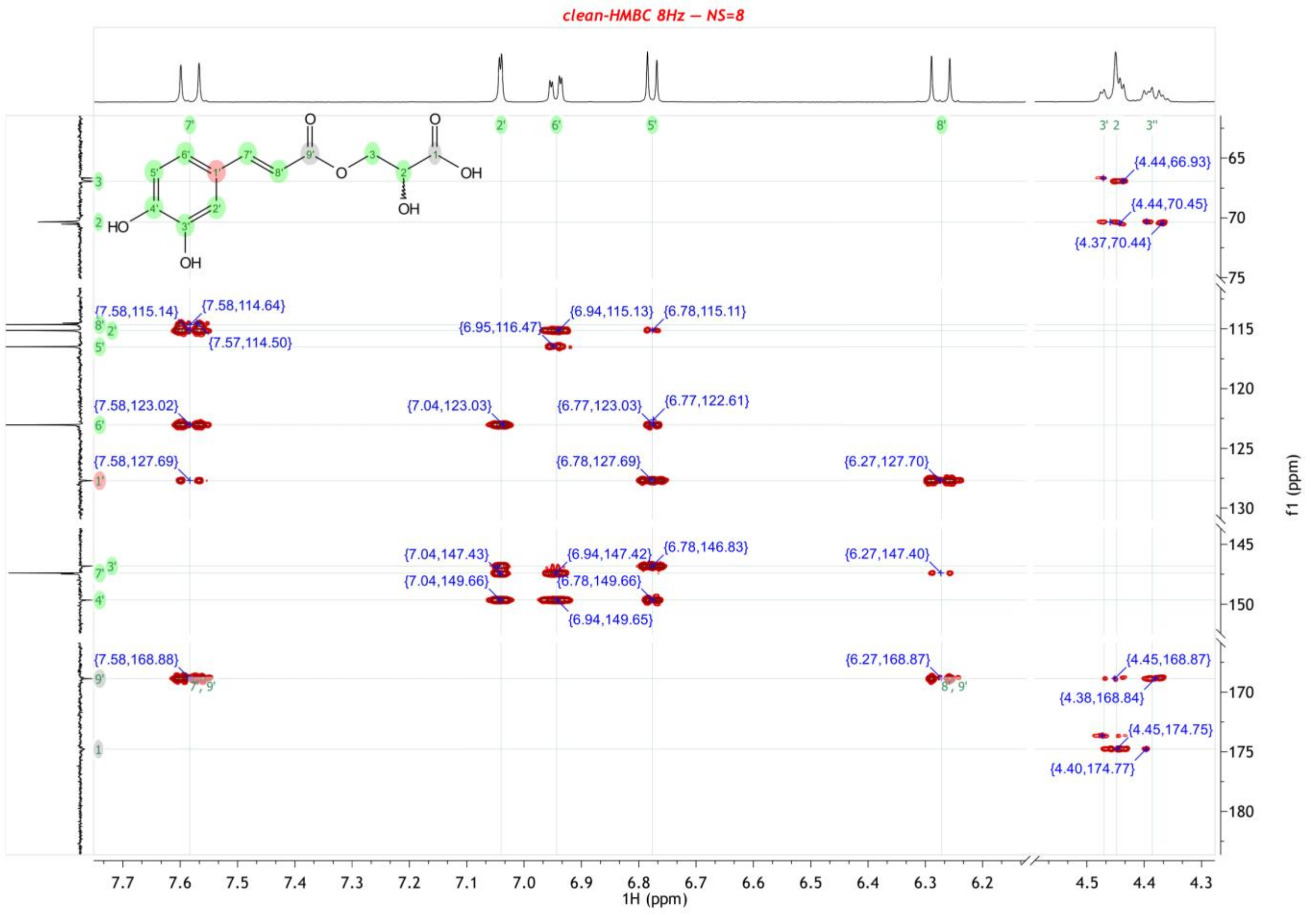

Figure 31S. ^{1}H-^{13}C HMBC (8 Hz) NMR spectrum of compound 14

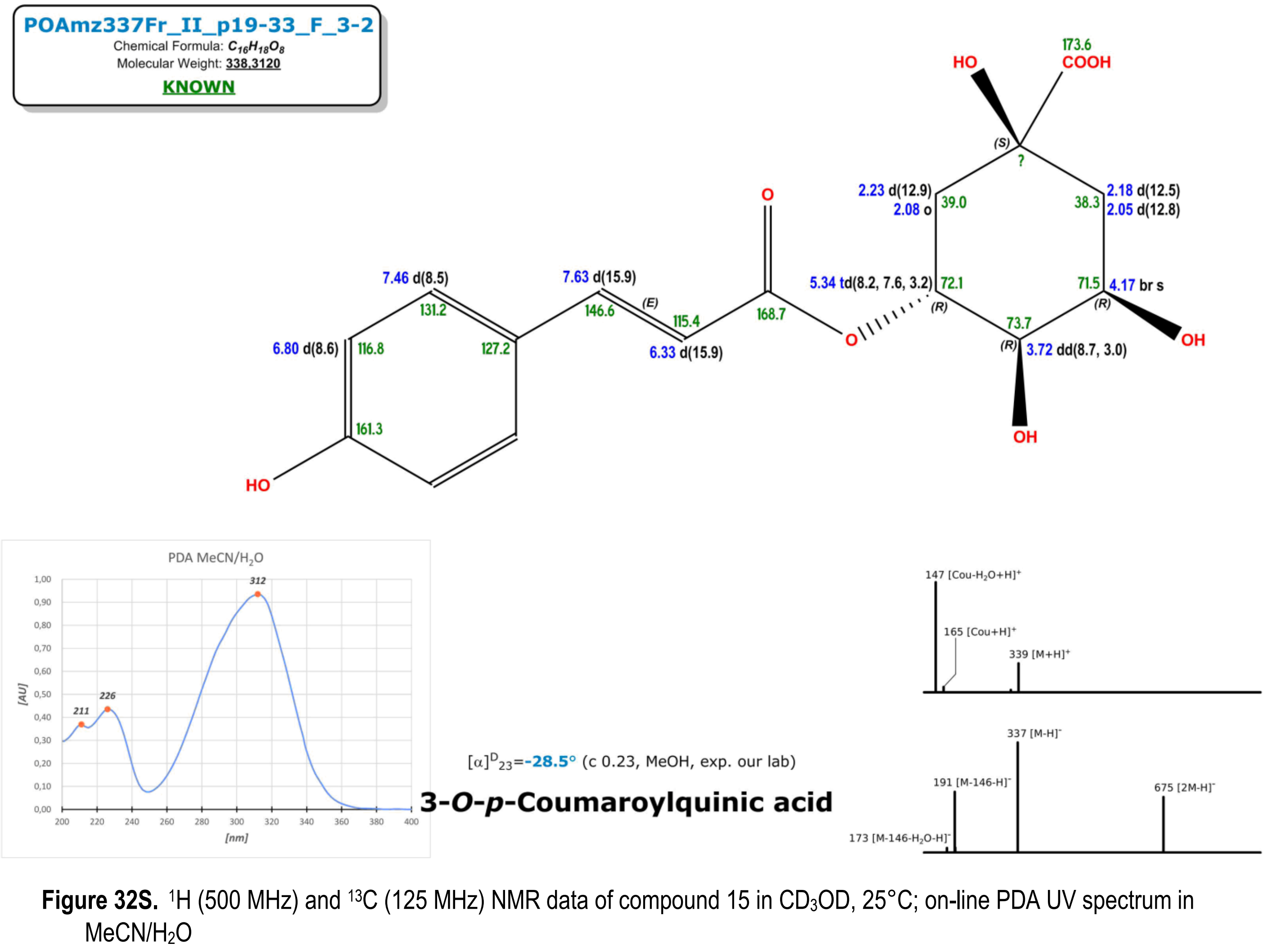

Figure 32S. ^{1}H (500 MHz) and ^{13}C (125 MHz) NMR data of compound 15 in CD_3OD, 25°C; on-line PDA UV spectrum in MeCN/H_2O

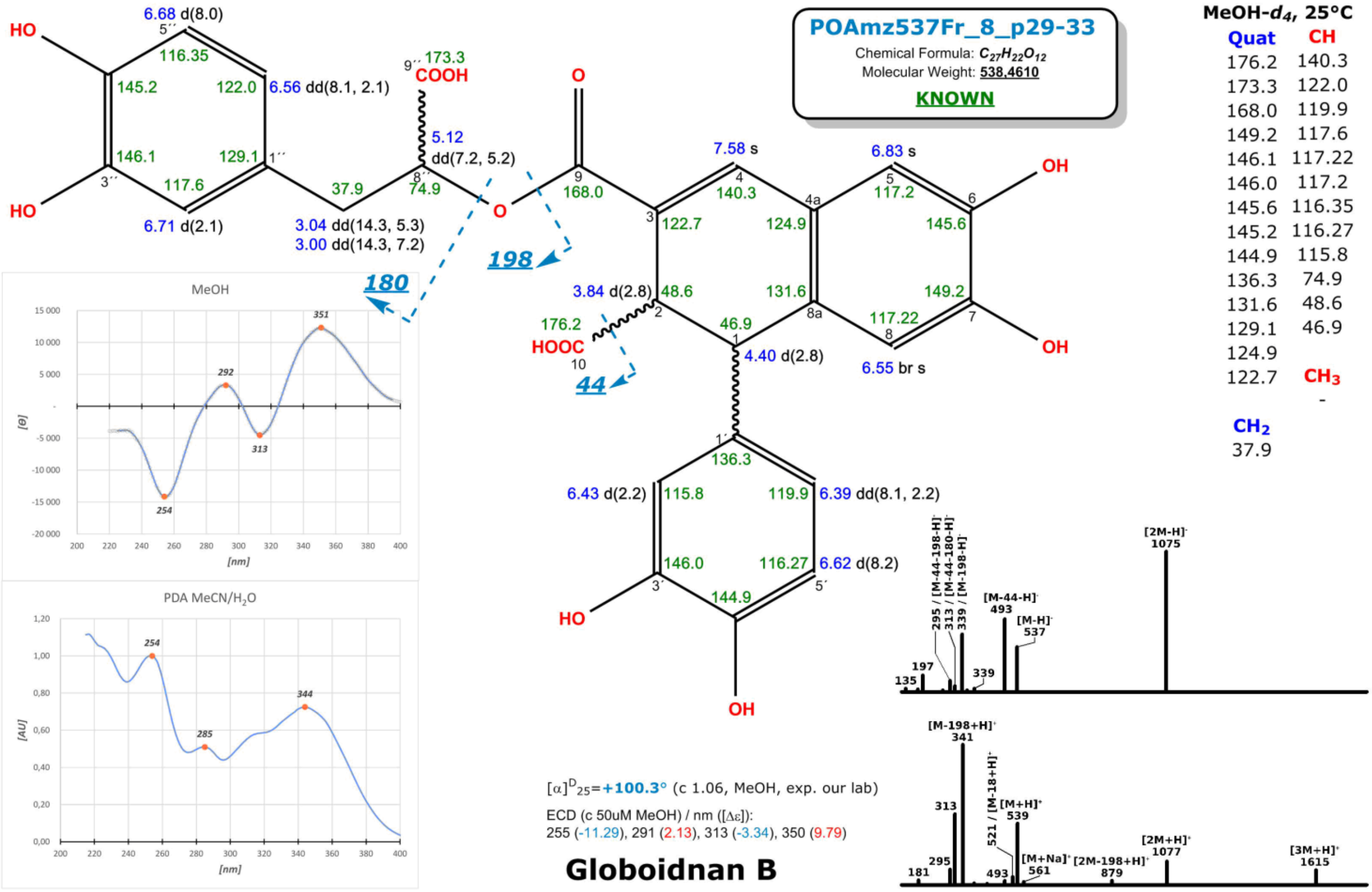

1. Basli, A., Delaunay, J.-C., Pedrot, E., Bernillon, S., Madani, K., Monti, J.-P., Mérillon, J.M., Chibane, M., Richard, T., 2014. New Cyclolignans from Origanum glandulosum Active Against ß-amyloid Aggregation. Rec. Nat. Prod. 8, 208–216.
2. Assoumatine, T., Datta, P.K., Hooper, T.S., Yvon, B.L., Charlton, J.L., 2004. A Short Asymmetric Synthesis of (+)-Lyoniresinol Dimethyl Ether. J. Org. Chem. 69, 4140–4144. doi:10.1021/jo0497454

Figure 33S. ^{1}H (500 MHz) and ^{13}C (125 MHz) NMR data of compound 18 in CD_3OD, 25°C; on-line PDA UV spectrum in MeCN/H_2O; ECD spectrum in MeOH

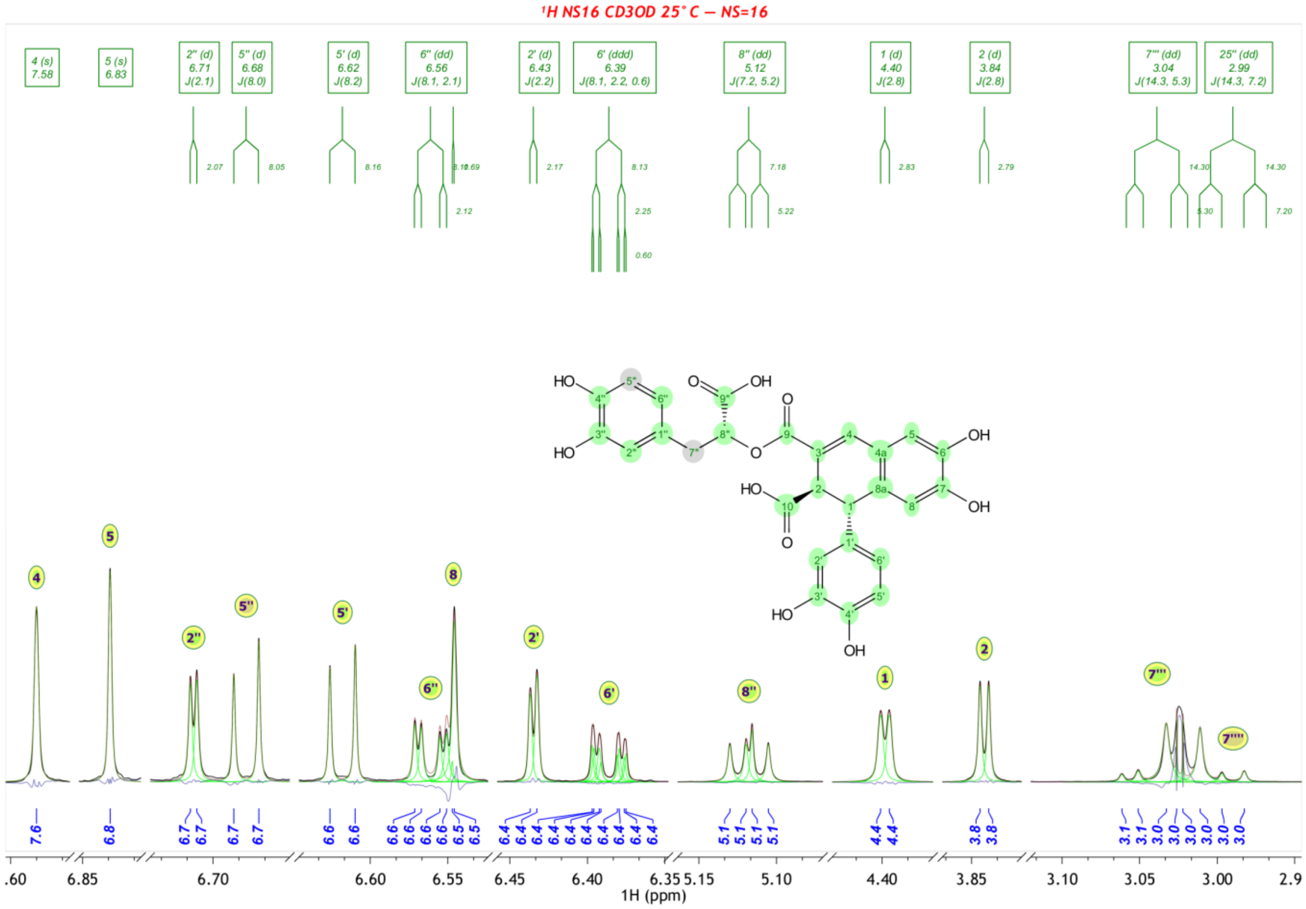

Figure 34S. ^{1}H NMR spectrum of compound 18

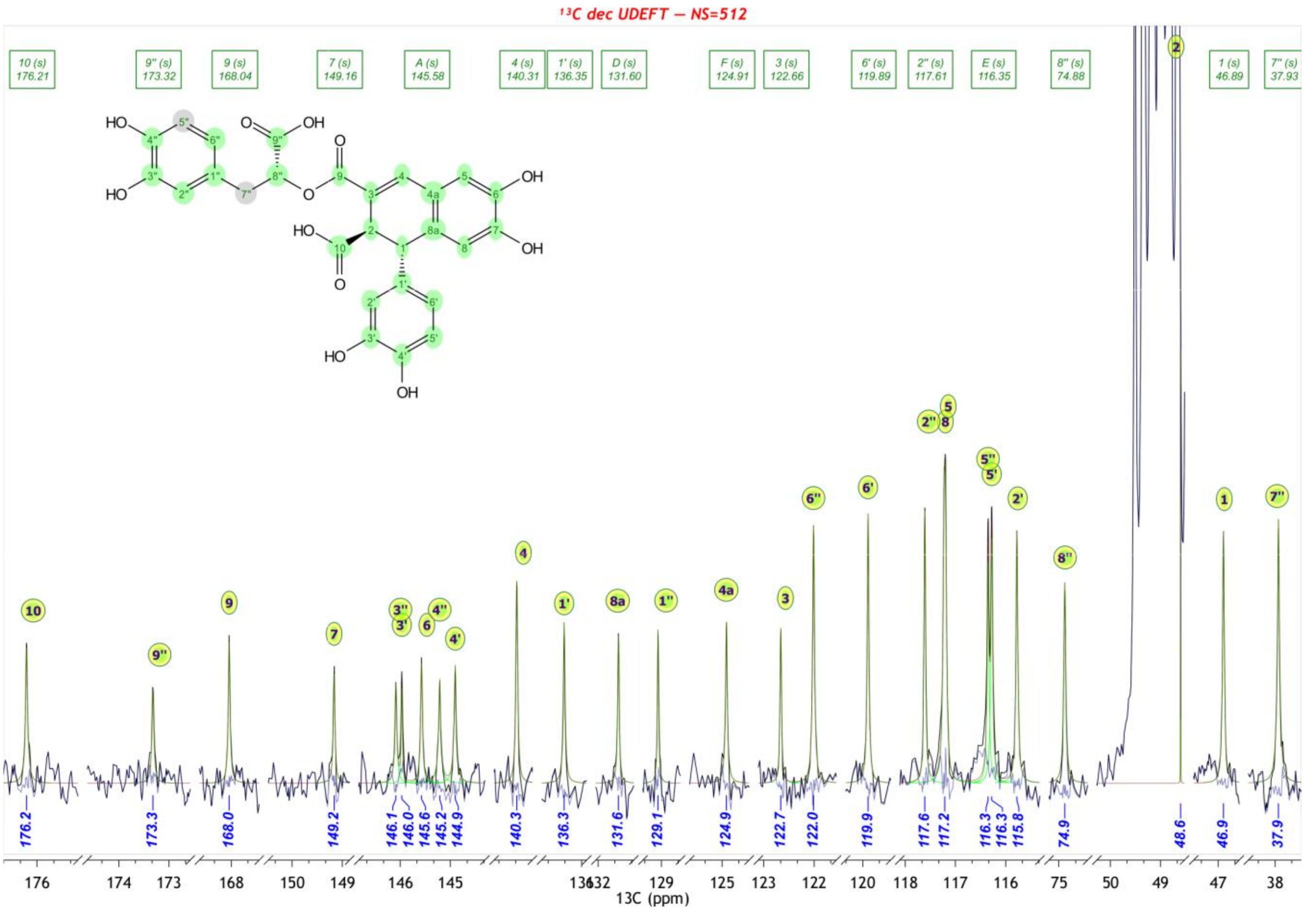

Figure 35S. ^{13}C DEPTQ NMR spectrum of compound 18

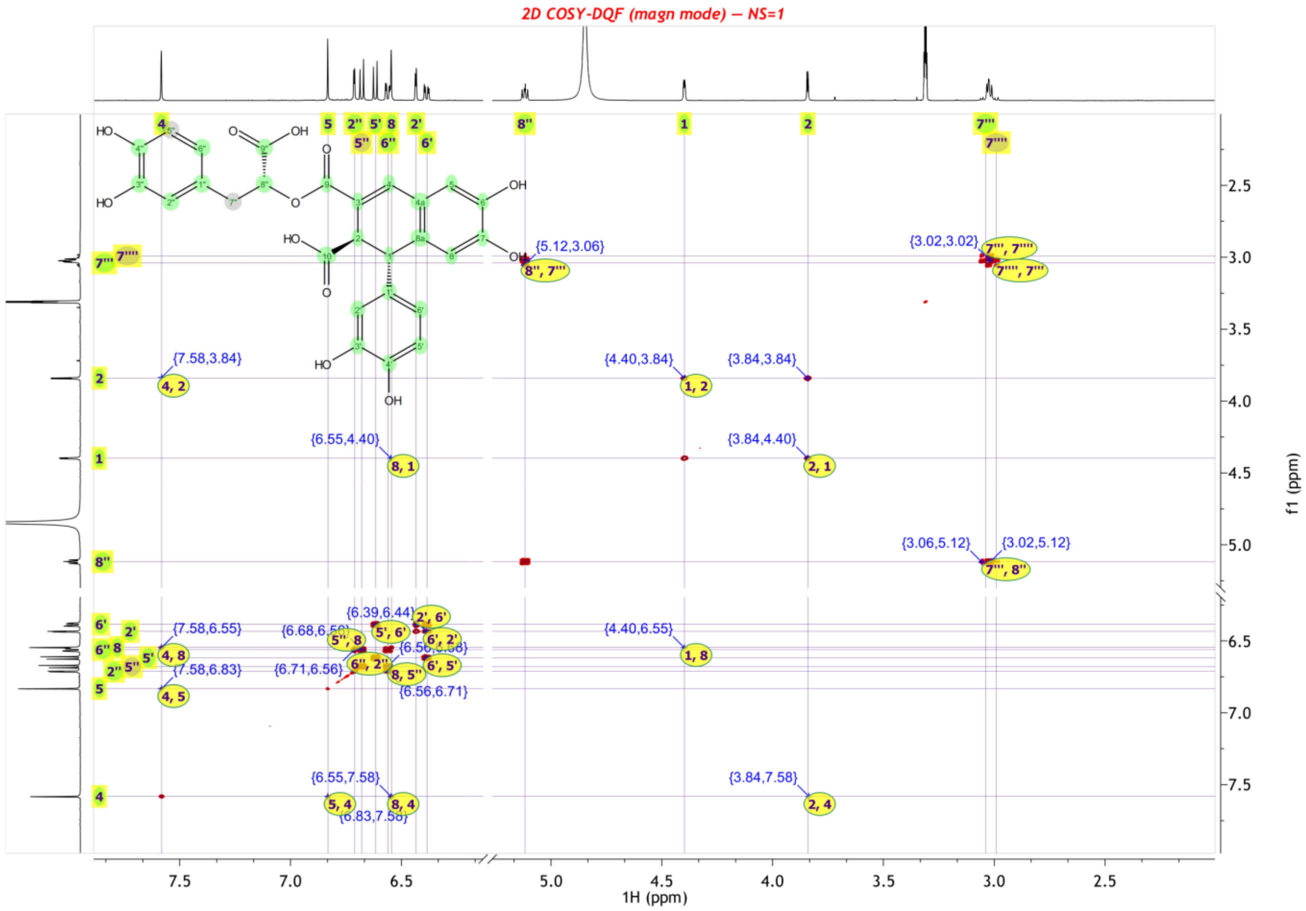

Figure 36S. ^{1}H-^{1}H COSY NMR spectrum of compound 18

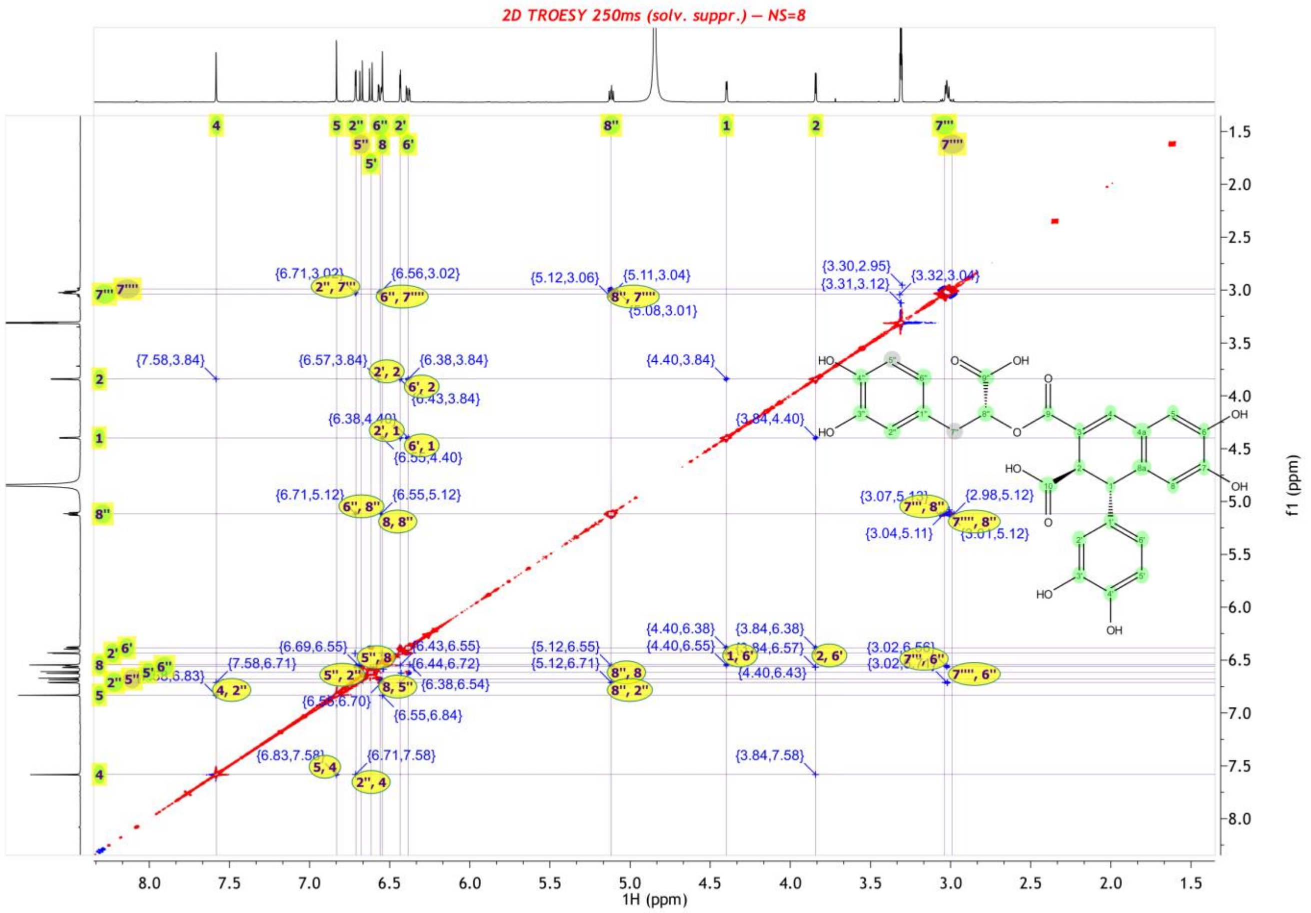

Figure 37S. ^{1}H-^{1}H TROESY (250 ms) NMR spectrum of compound 18

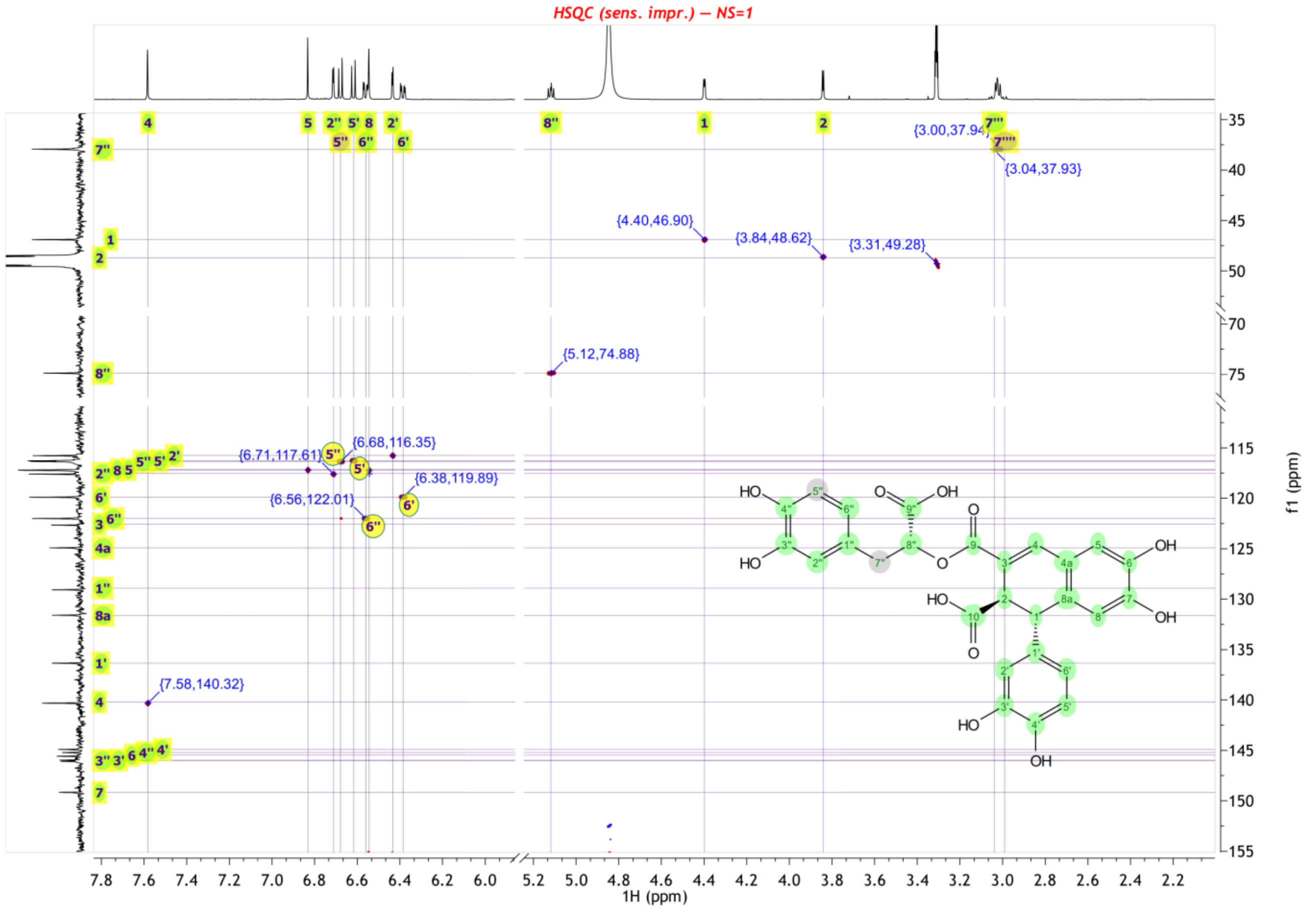

Figure 38S. ^{1}H-^{13}C HSQC NMR spectrum of compound 18

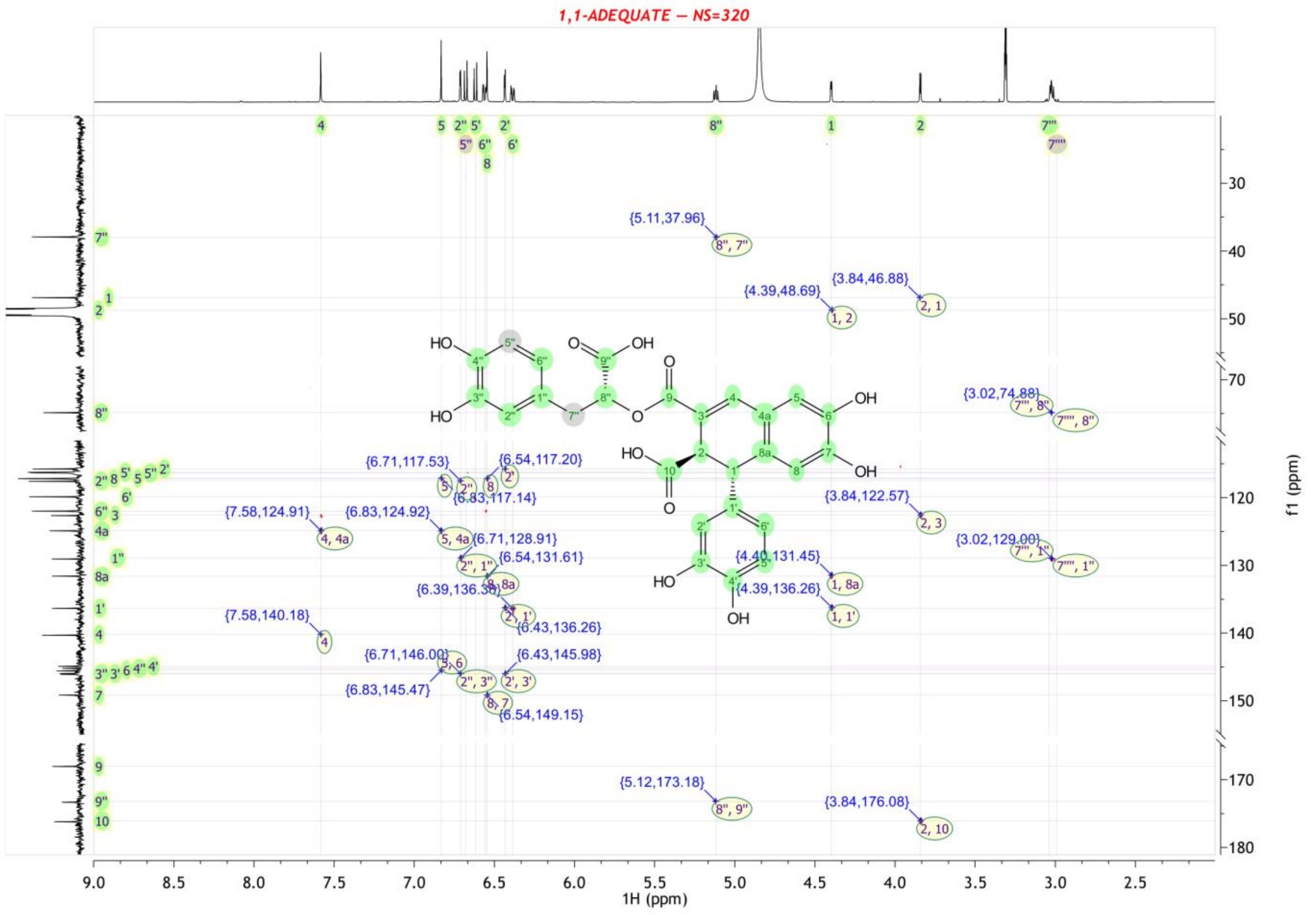

Figure 39S. ^{1}H-^{13}C 1,1-ADEQUATE NMR spectrum of compound 18

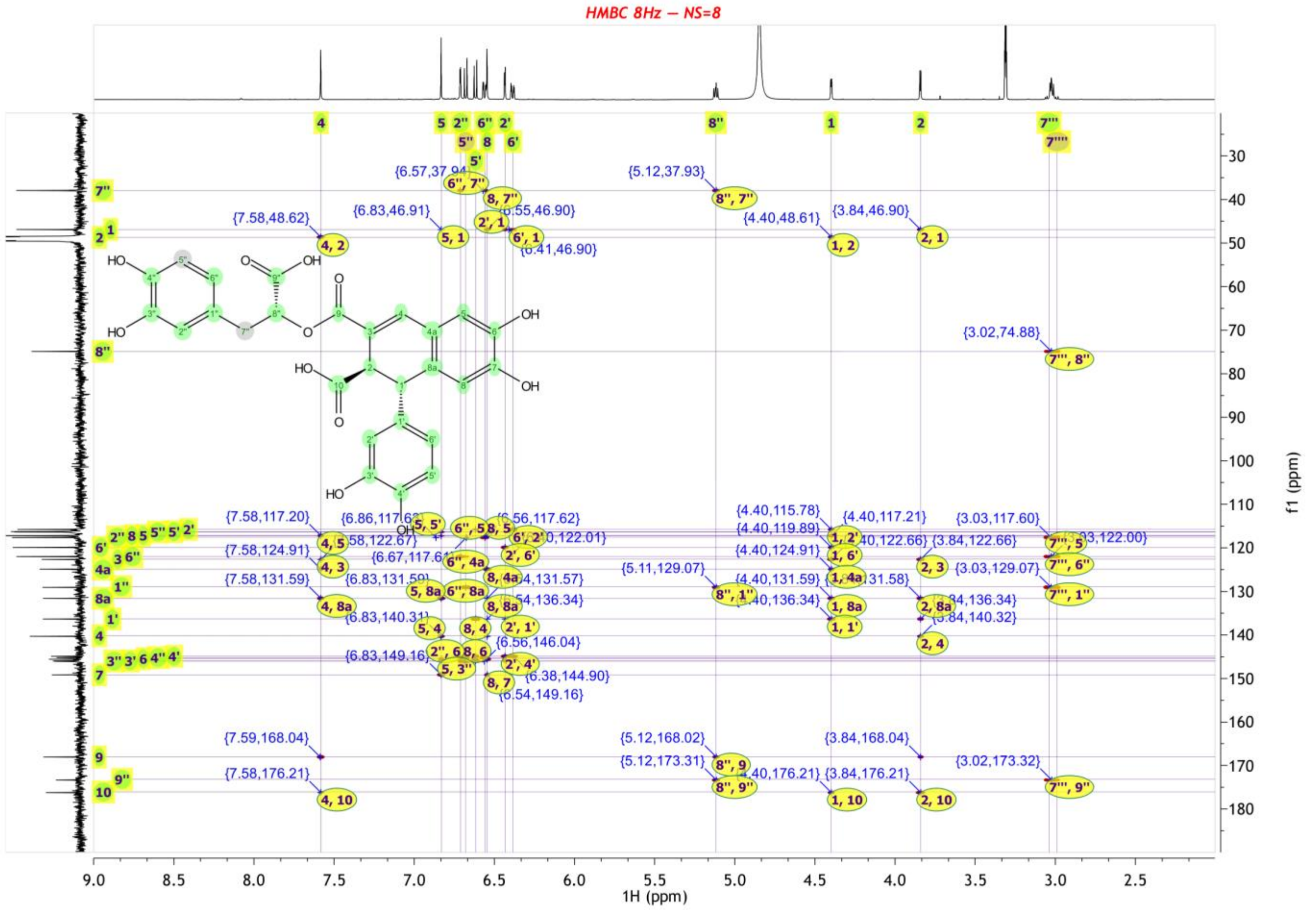

Figure 40S. ^{1}H-^{13}C HMBC (8 Hz) NMR spectrum of compound 18

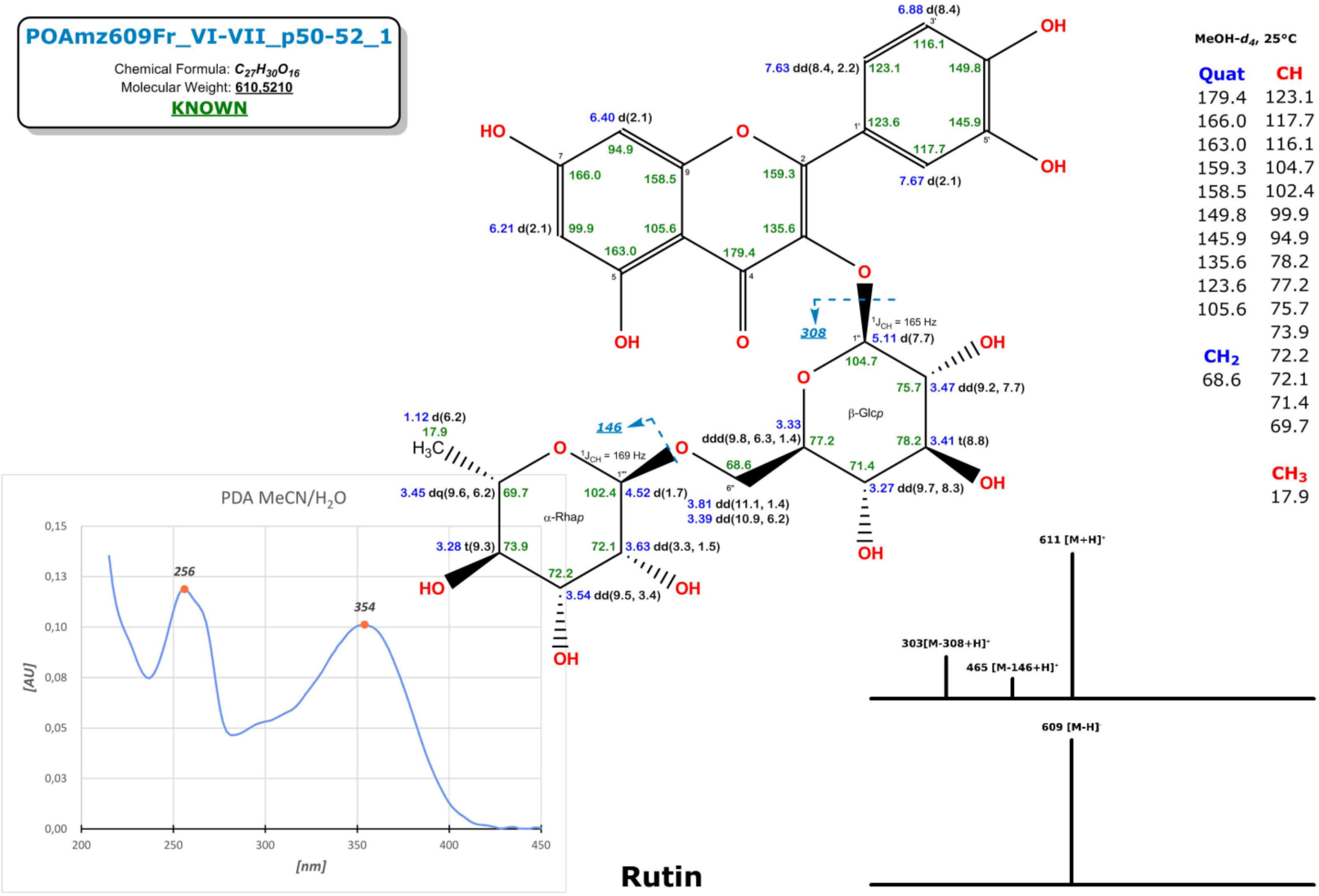

Figure 41S. ^{1}H (500 MHz) and ^{13}C (125 MHz) NMR data of compound 19 in CD_3OD, 25°C; on-line PDA UV spectrum in MeCN/H_2O

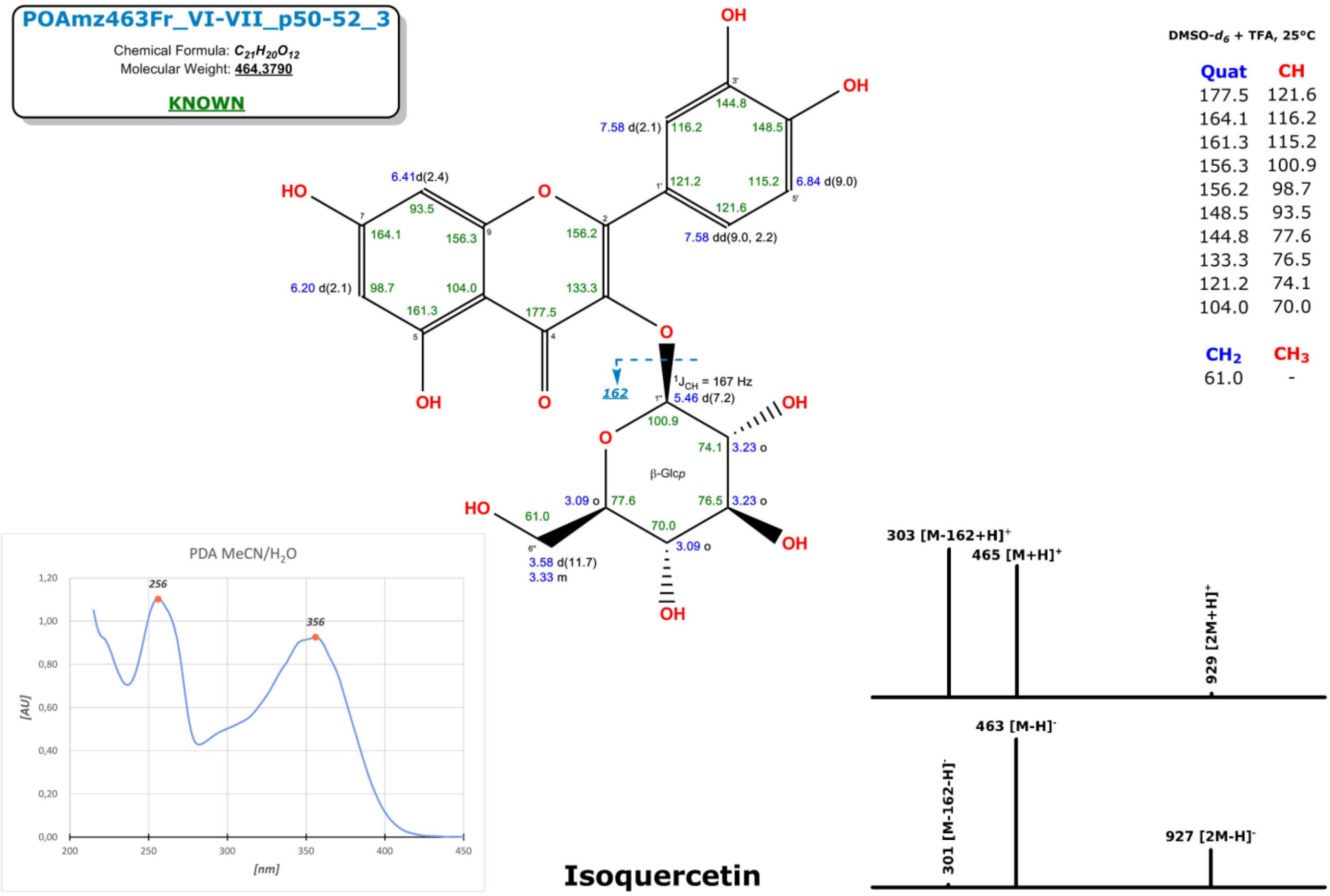

Figure 42S. ^{1}H (500 MHz) and ^{13}C (125 MHz) NMR data of compound 21 in CD_3OD, 25°C; on-line PDA UV spectrum in MeCN/H_2O

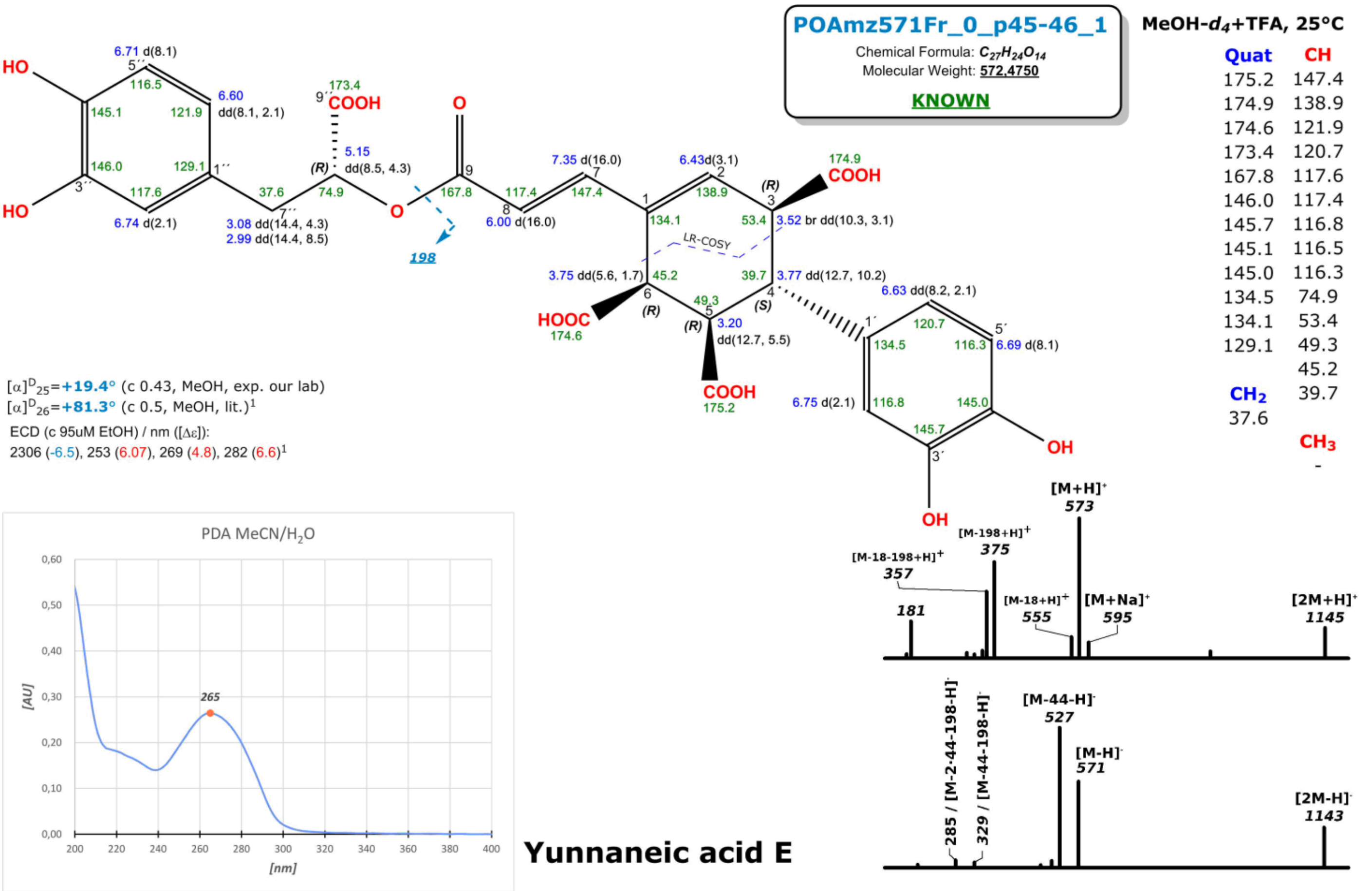

1. TANAKA, T., NISHIMURA, A., KOUNO, I., NONAKA, G., YANG, C.-R., 1997. Four New Caffeic Acid Metabolites, Yunnaneic Acids E-H, from Salvia yunnanensis. Chem. Pharm. Bull. (Tokyo). 45, 1596–1600. doi:10.1248/cpb.45.1596

Figure 43S. ^{1}H (500 MHz) and ^{13}C (125 MHz) NMR data of compound 22 in CD_3OD, 25°C; on-line PDA UV spectrum in $MeCN/H_2O$

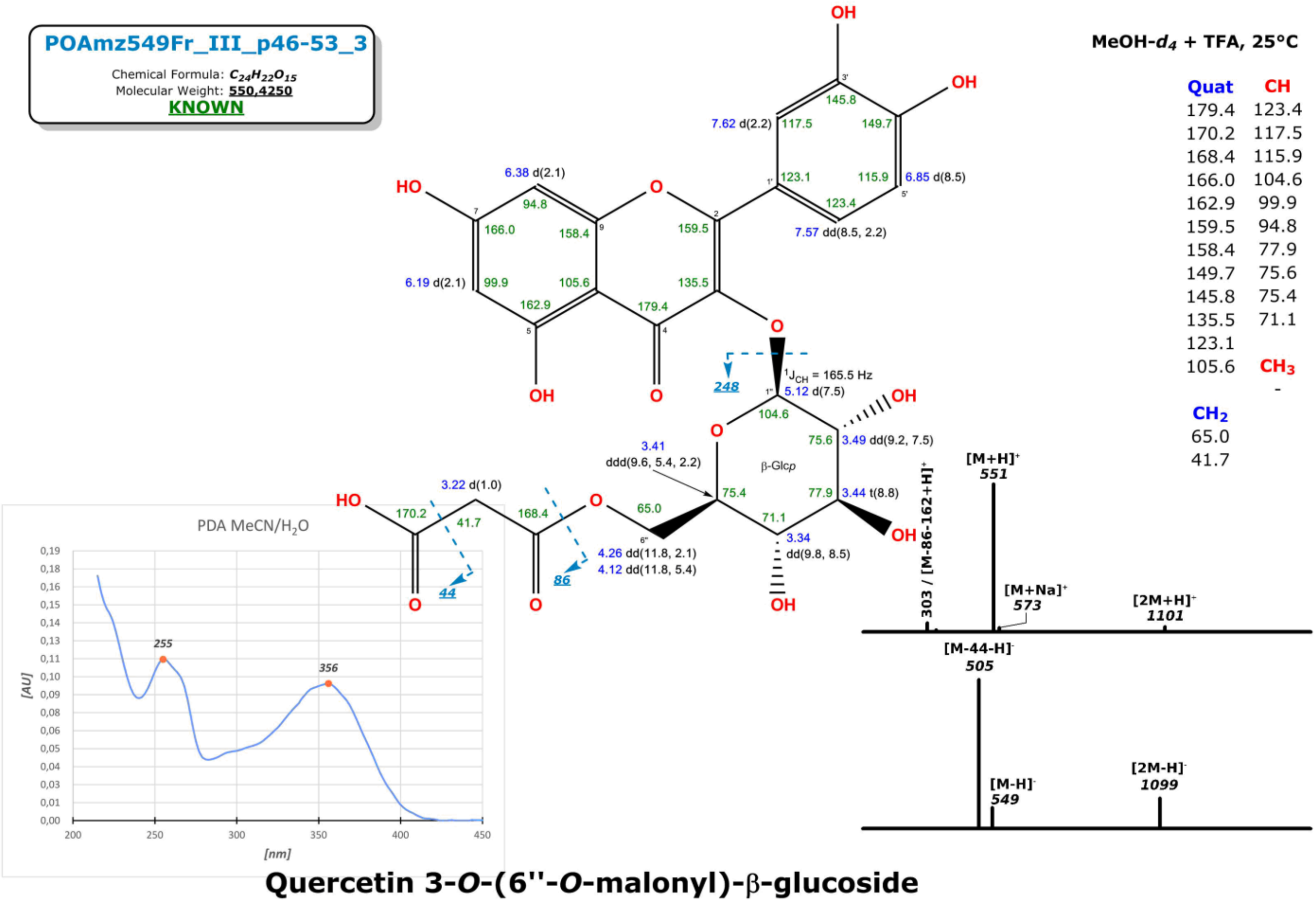

Figure 44S. ^{1}H (500 MHz) and ^{13}C (125 MHz) NMR data of compound 23 in CD_3OD, 25°C; on-line PDA UV spectrum in $MeCN/H_2O$

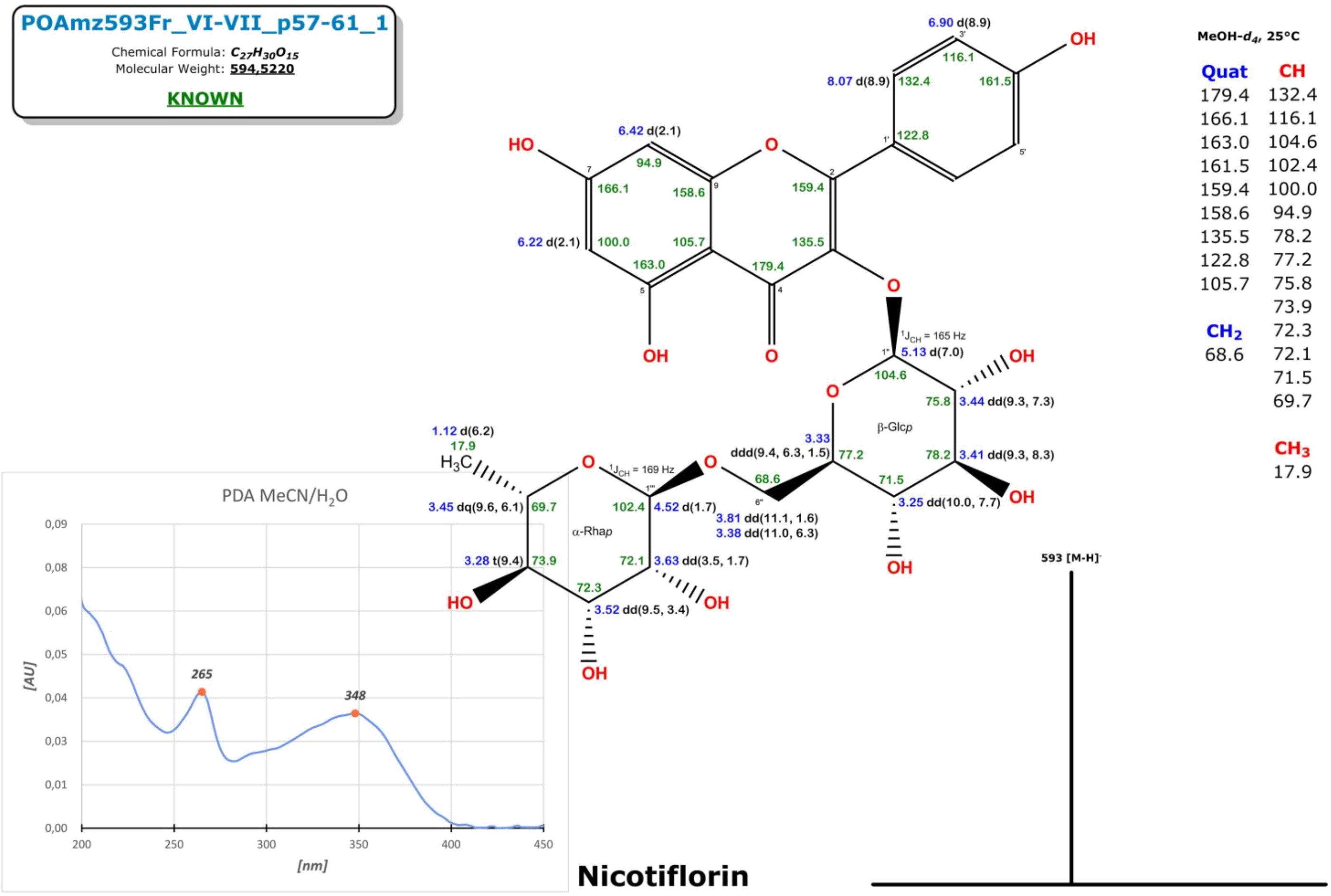

Figure 45S. ^{1}H (500 MHz) and ^{13}C (125 MHz) NMR data of compound 24 in CD_3OD, 25°C; on-line PDA UV spectrum in $MeCN/H_2O$

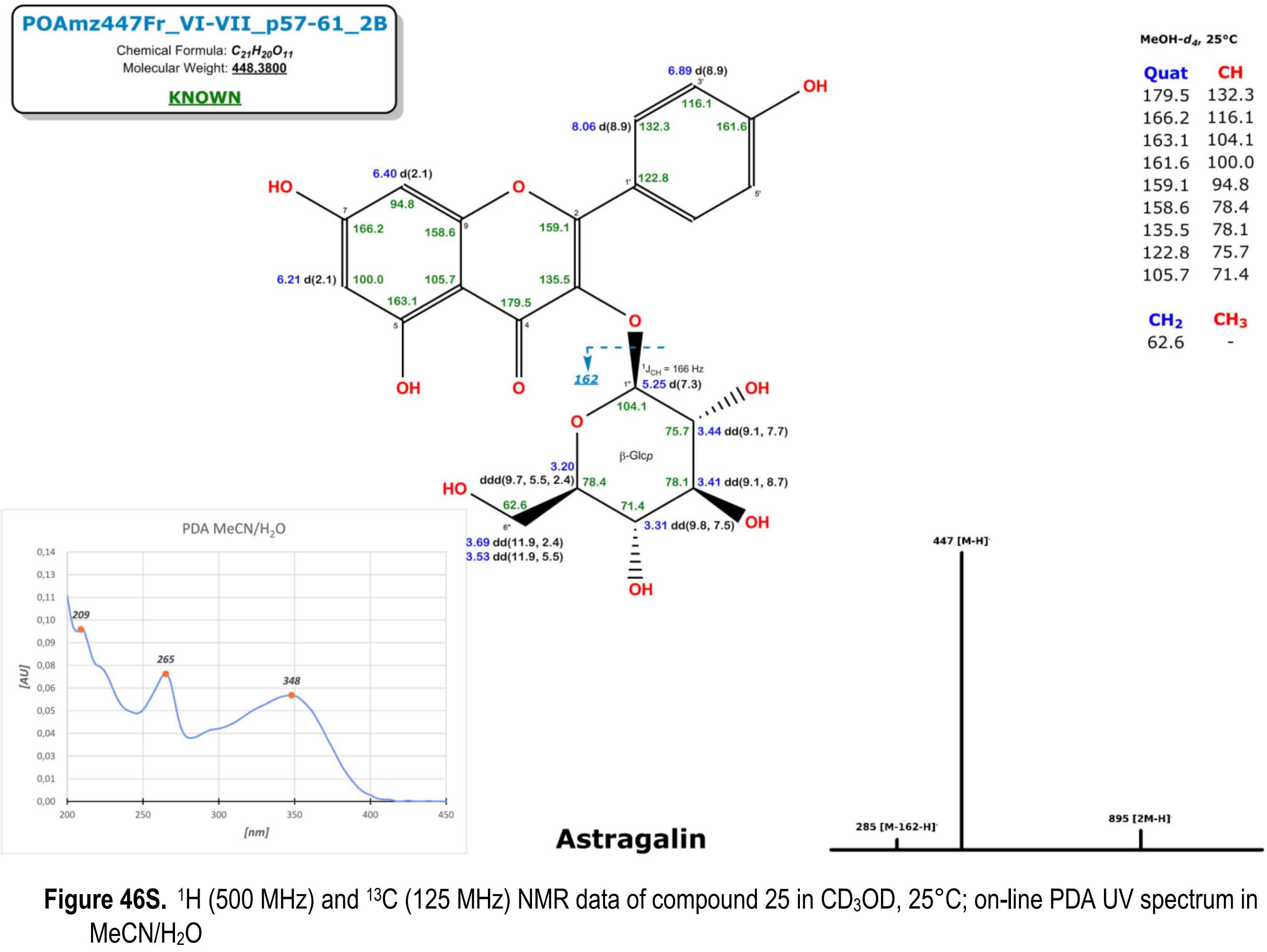

Figure 46S. ^{1}H (500 MHz) and ^{13}C (125 MHz) NMR data of compound 25 in CD_3OD, 25°C; on-line PDA UV spectrum in $MeCN/H_2O$

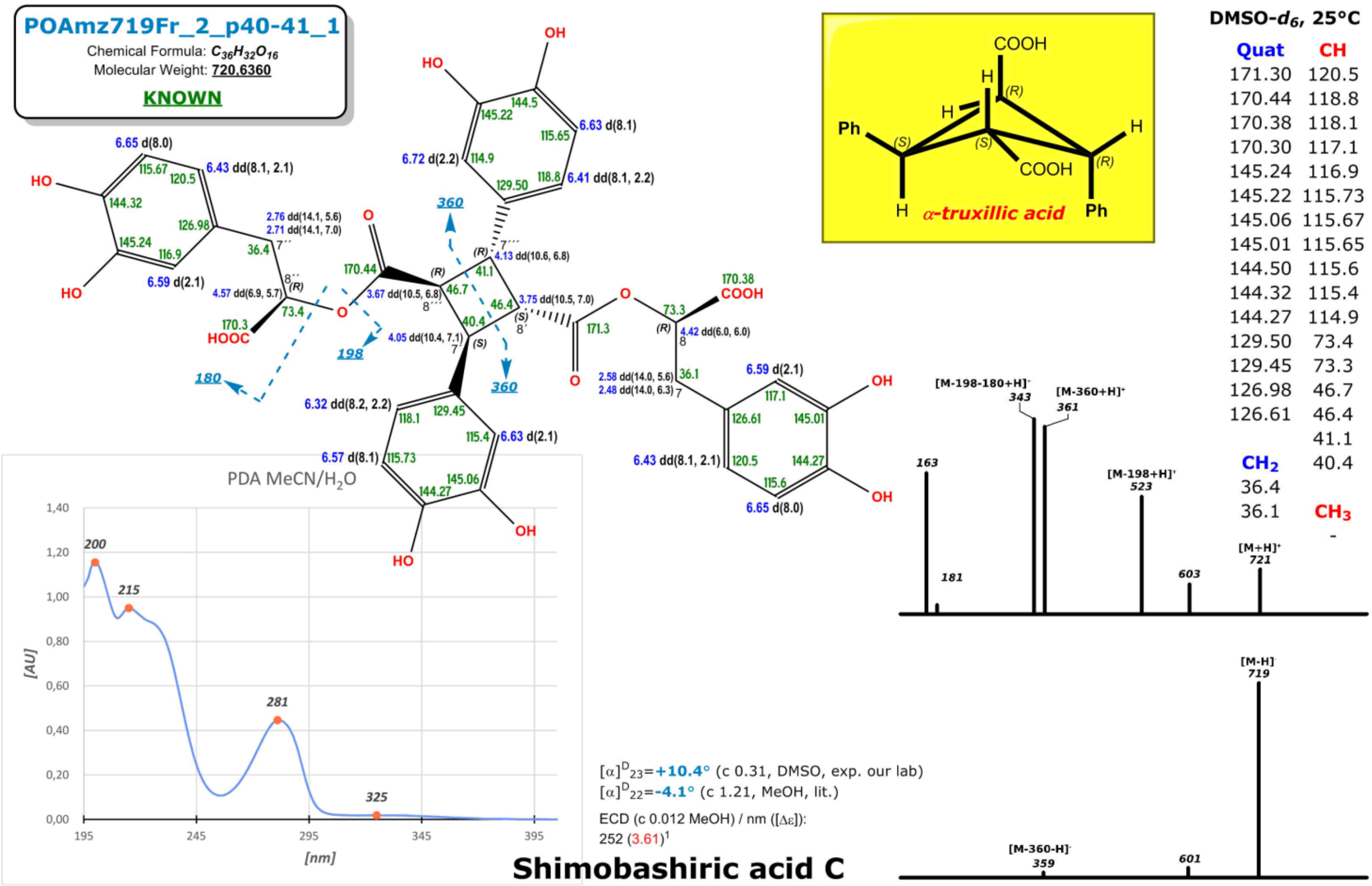

1. Murata, T., Miyase, T., Yoshizaki, F., 2012. Hyaluronidase Inhibitors from Keiskea japonica. Chem. Pharm. Bull. (Tokyo). 60, 121–128. doi:10.1248/cpb.60.121
2. Chen, Y.-S., Yu, H.-M., Shie, J.-J., Cheng, T.-J.R., Wu, C.-Y., Fang, J.-M., Wong, C.-H., 2014. Chemical constituents of Plectranthus amboinicus and the synthetic analogs possessing anti-inflammatory activity. Bioorg. Med. Chem. 22, 1766–1772. doi:10.1016/j.bmc.2014.01.009

Figure 47S. ^1H (500 MHz) and ^{13}C (125 MHz) NMR data of compound 26 in DMSO-d_6, 25°C; on-line PDA UV spectrum in MeCN/H_2O

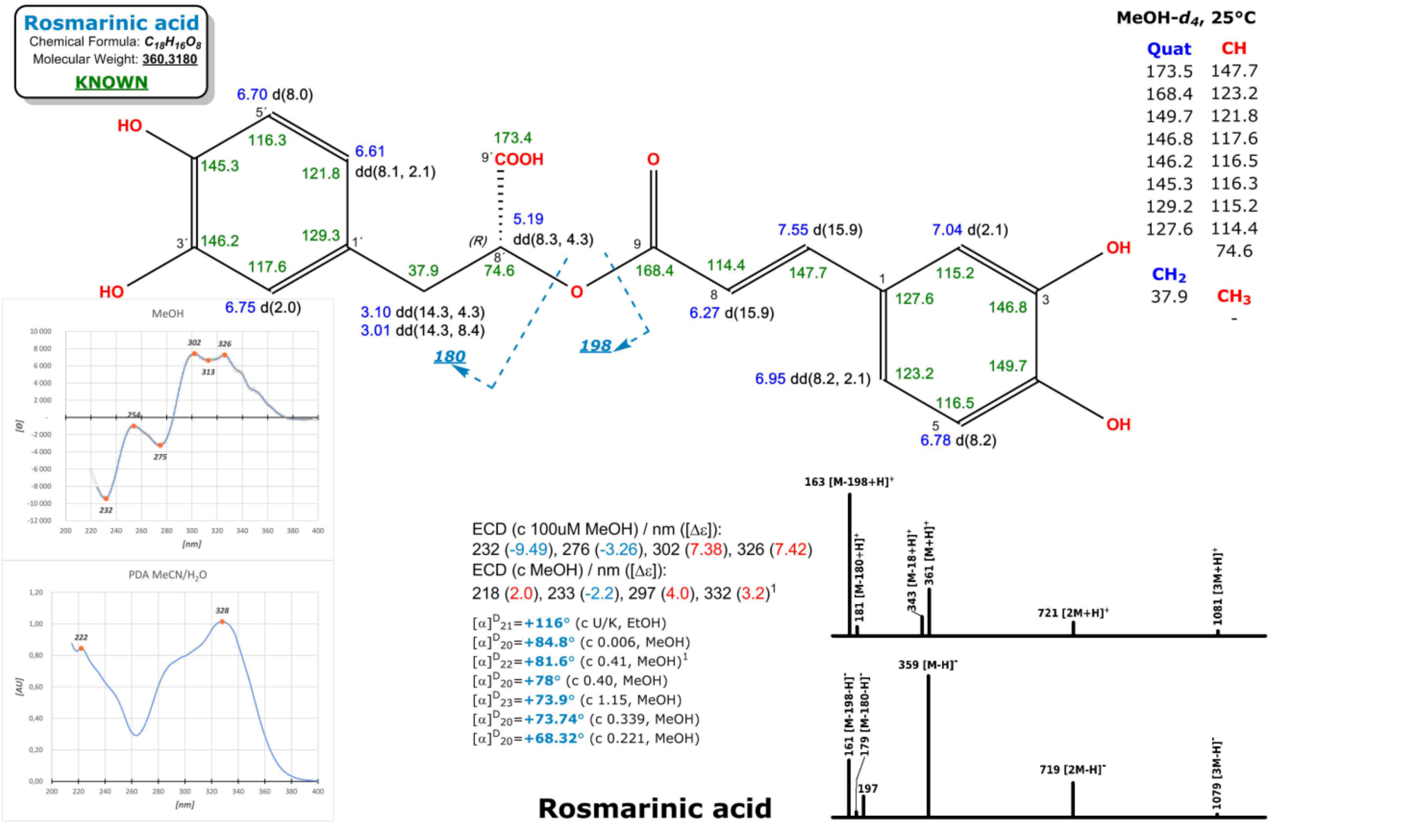

1. Dapkevicius, A., van Beek, T.A., Lelyveld, G.P., van Veldhuizen, A., de Groot, A., Linssen, J.P.H., Venskutonis, R., 2002. Isolation and Structure Elucidation of Radical Scavengers from Thymus vulgaris Leaves. J. Nat. Prod. 65, 892–896. doi:10.1021/np010636j

Figure 48S. ^1H (500 MHz) and ^{13}C (125 MHz) NMR data of compound 27 in CD_3OD, 25°C; on-line PDA UV spectrum in MeCN/H_2O; ECD spectrum in MeOH

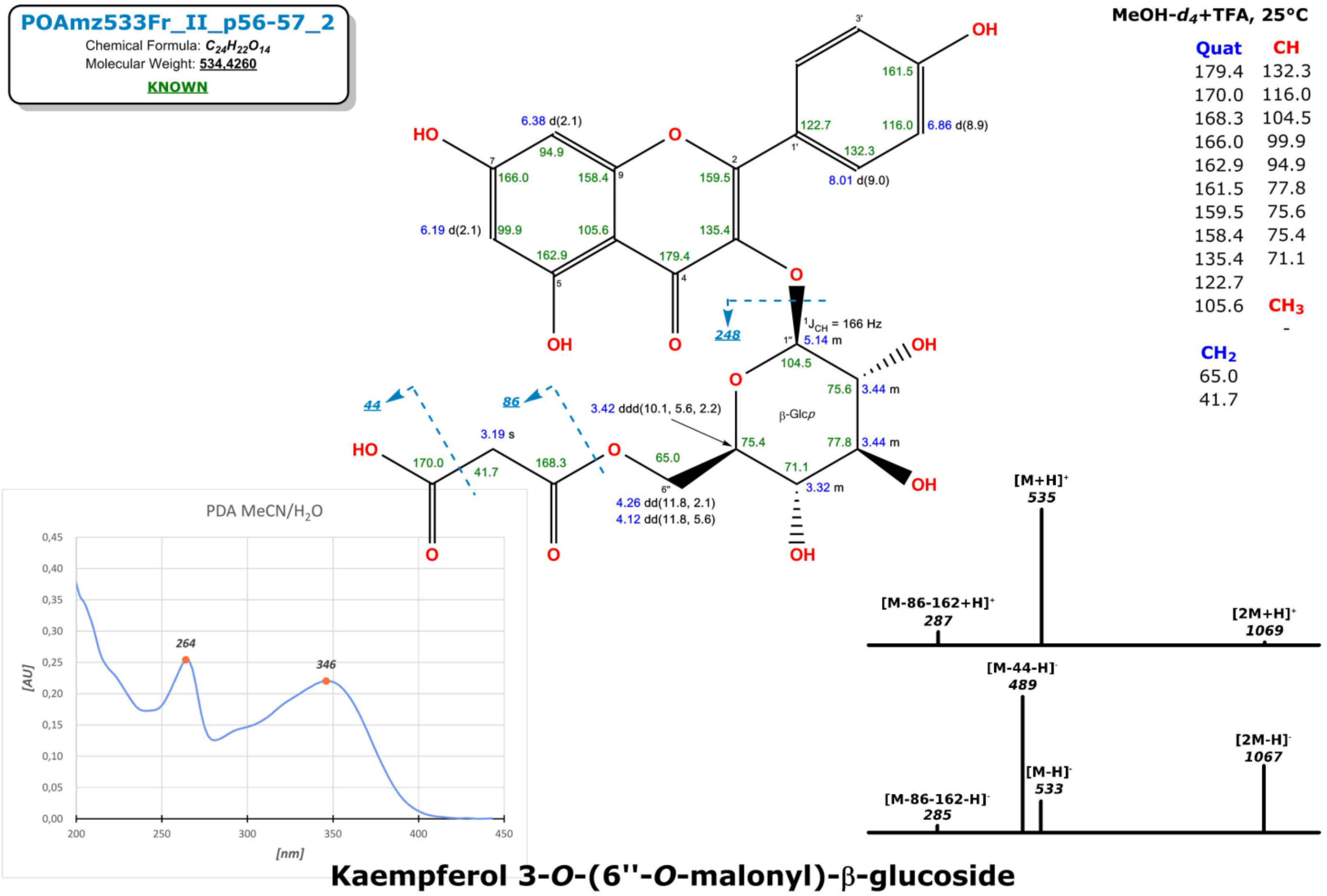

Figure 49S. ^{1}H (500 MHz) and ^{13}C (125 MHz) NMR data of compound 28 in CD_3OD, 25°C; on-line PDA UV spectrum in MeCN/H_2O

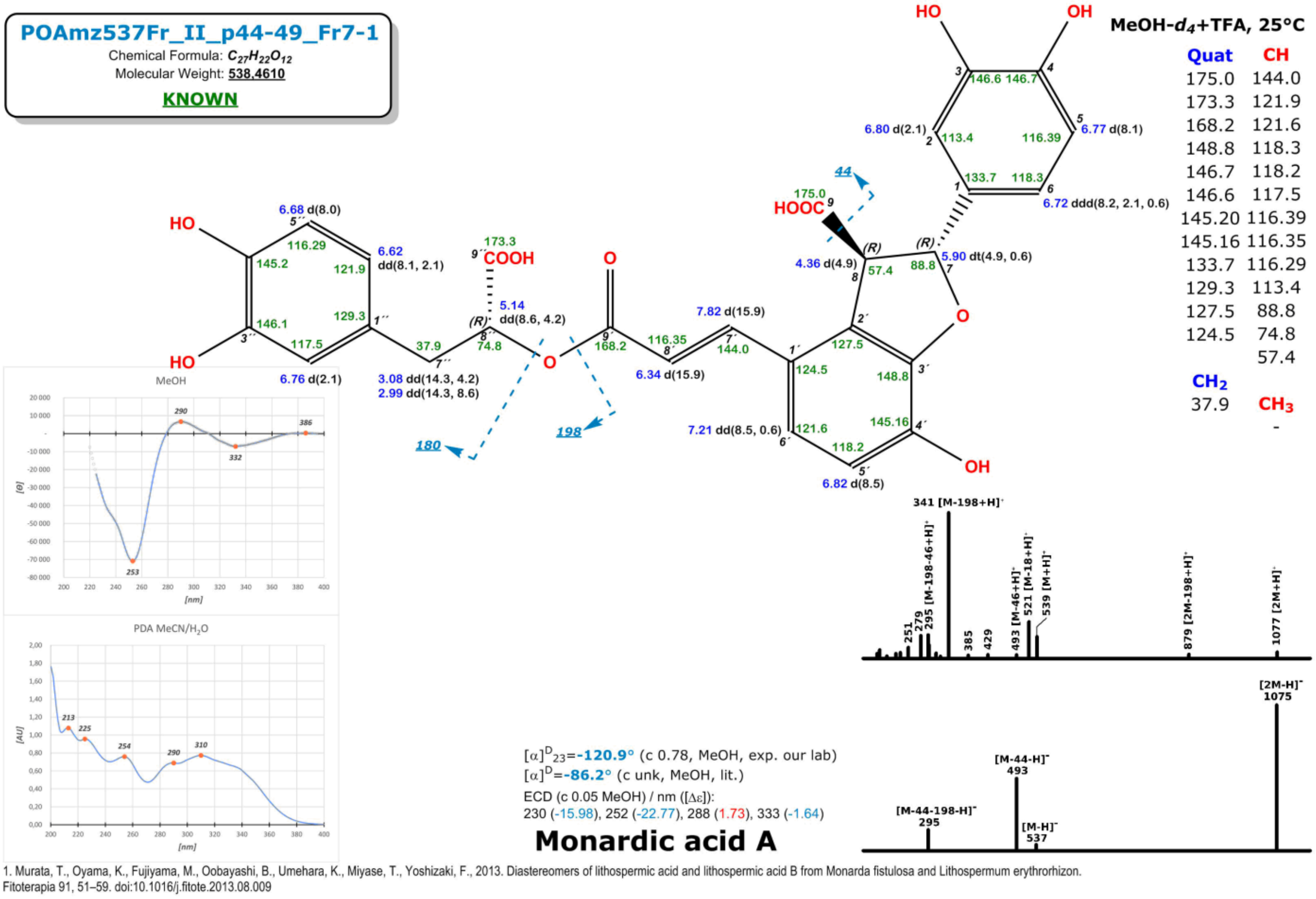

1. Murata, T., Oyama, K., Fujiyama, M., Oobayashi, B., Umehara, K., Miyase, T., Yoshizaki, F., 2013. Diastereomers of lithospermic acid and lithospermic acid B from Monarda fistulosa and Lithospermum erythrorhizon. Fitoterapia 91, 51–59. doi:10.1016/j.fitote.2013.08.009

Figure 50S. ^{1}H (500 MHz) and ^{13}C (125 MHz) NMR data of compound 29 in CD_3OD, 25°C; on-line PDA UV spectrum in MeCN/H_2O; ECD spectrum in MeOH

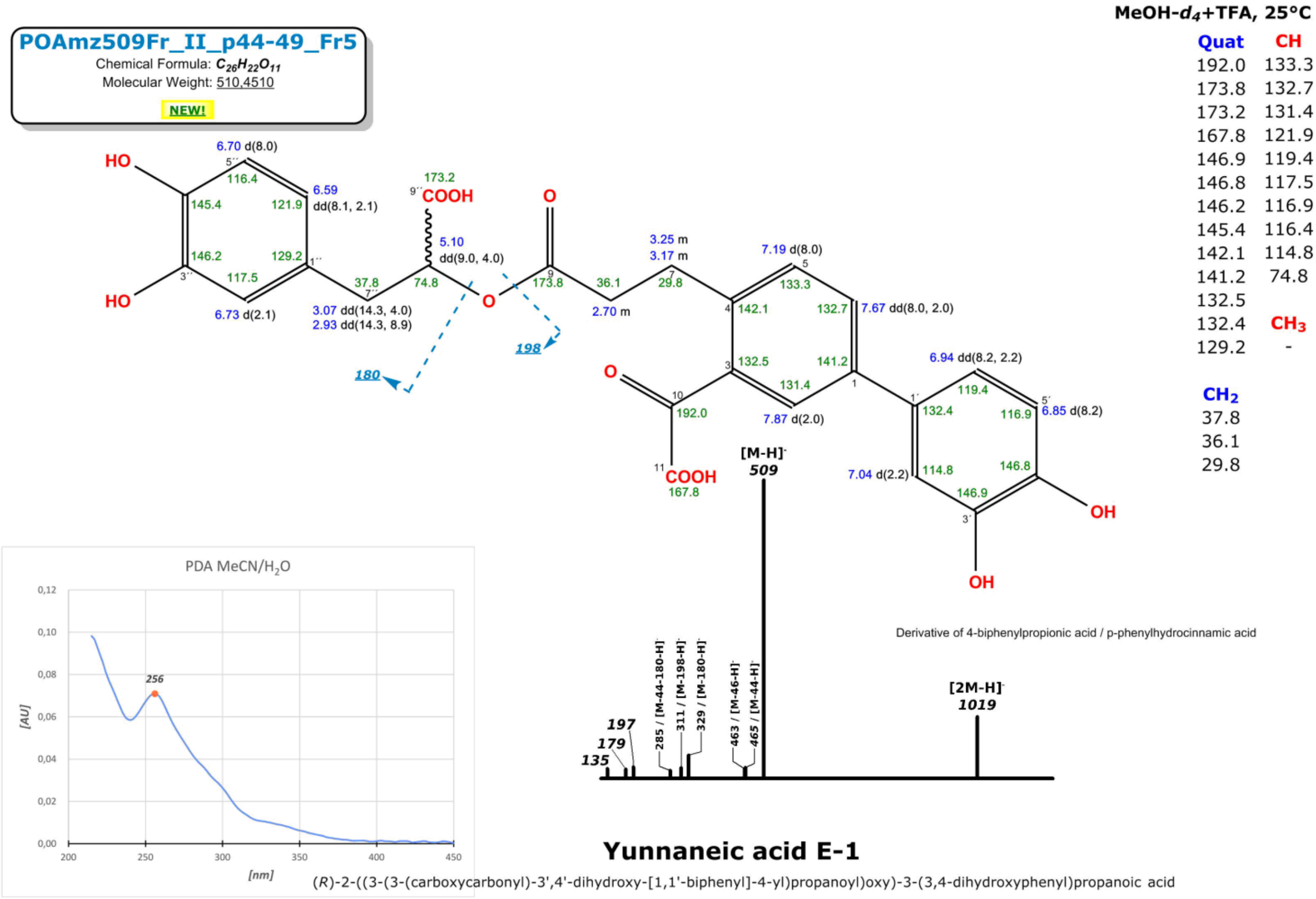

Figure 51S. ^{1}H (500 MHz) and ^{13}C (125 MHz) NMR data of compound 30 in CD_3OD, 25°C; on-line PDA UV spectrum in $MeCN/H_2O$

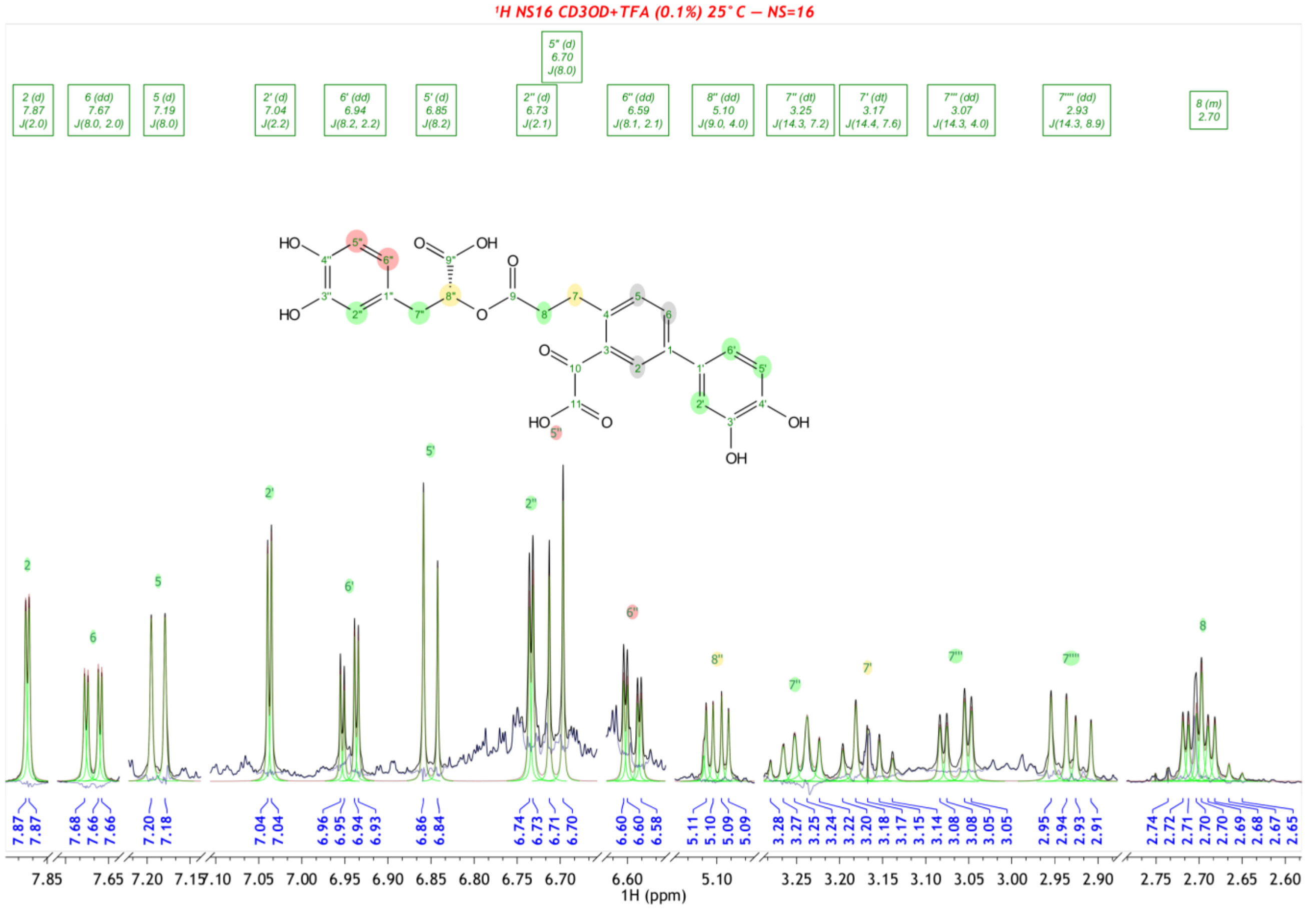

Figure 52S. ^{1}H NMR spectrum of compound 30

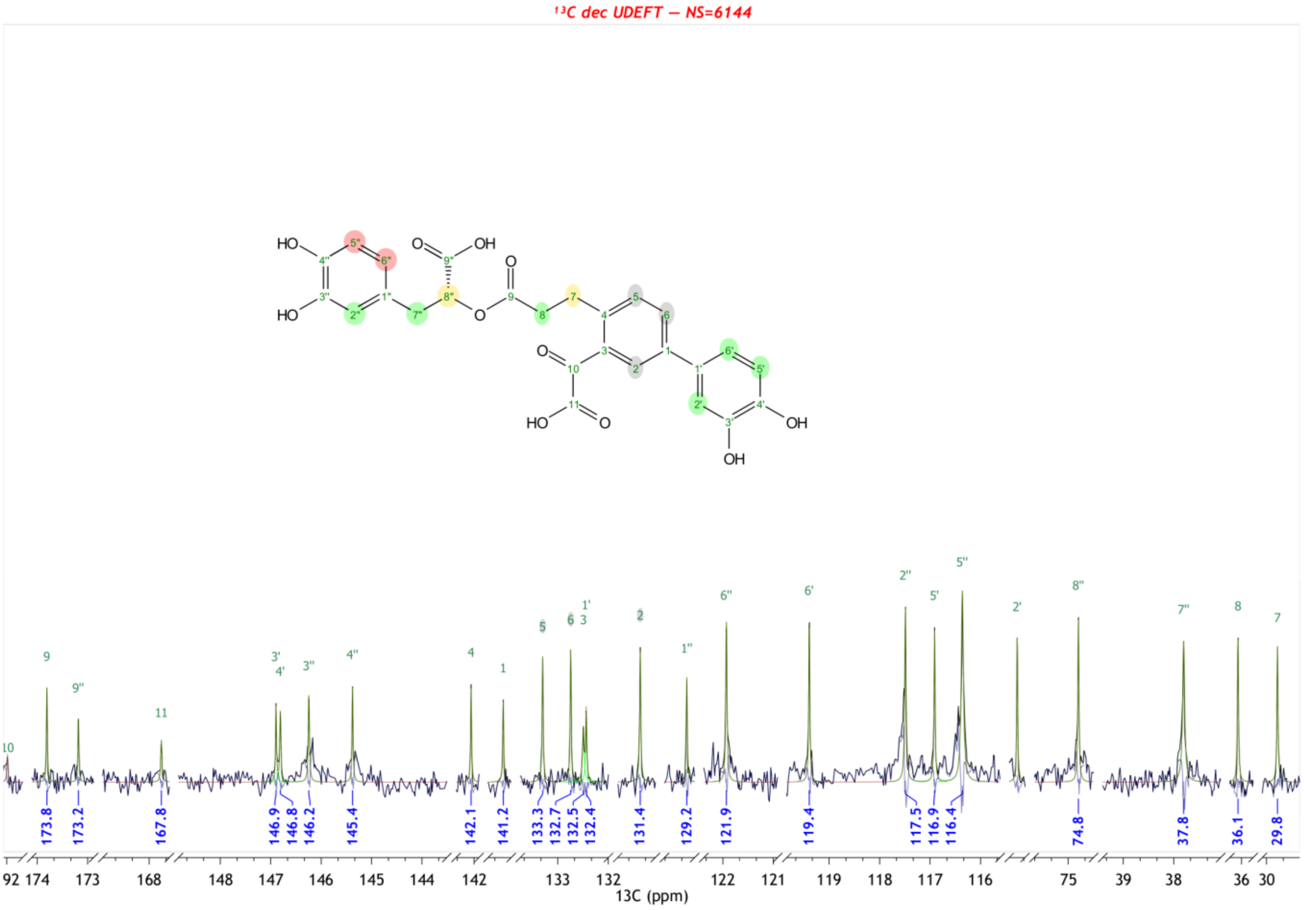

Figure 53S. ^{13}C DEPTQ NMR spectrum of compound 30

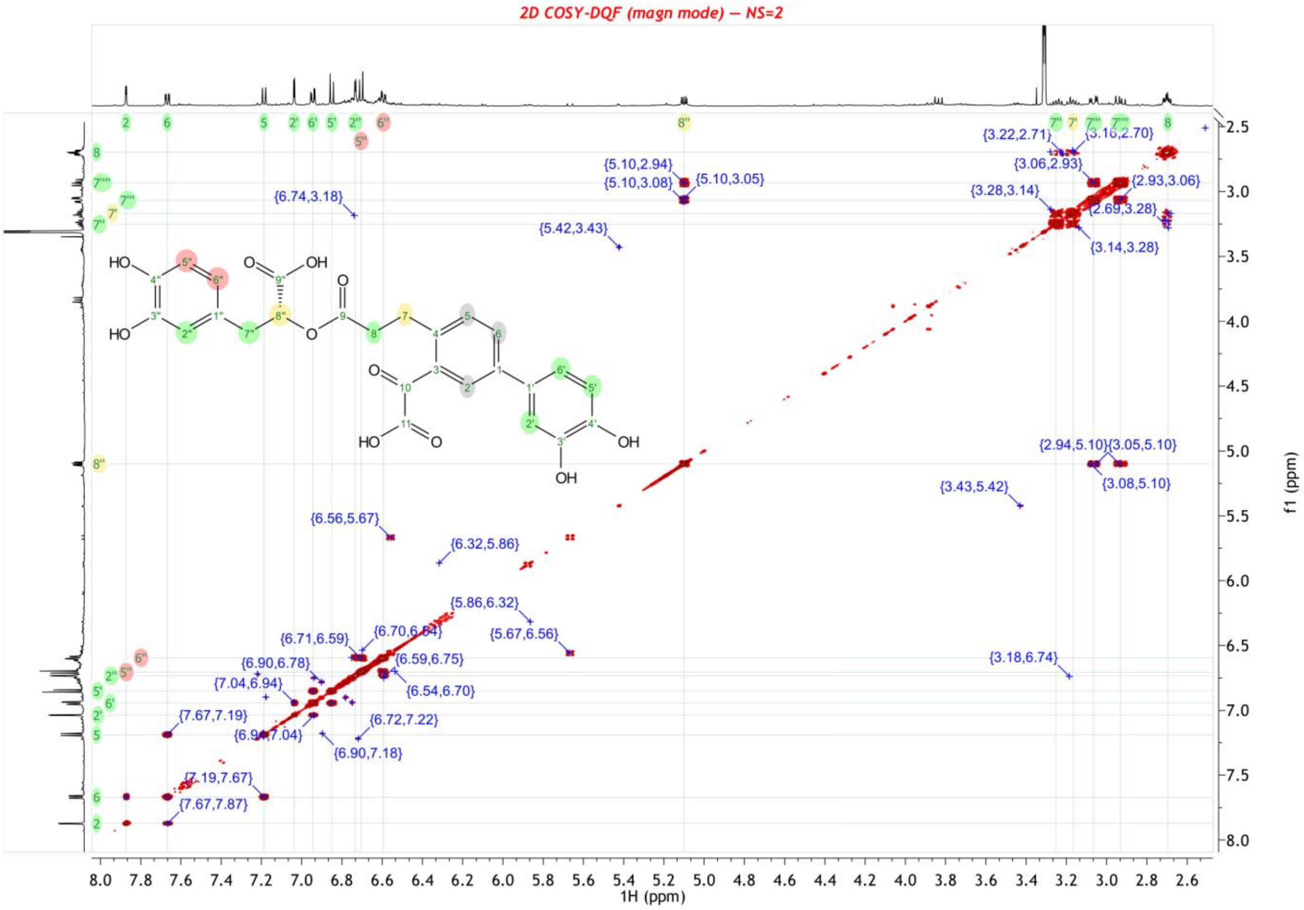

Figure 54S. ^{1}H-^{1}H COSY NMR spectrum of compound 30

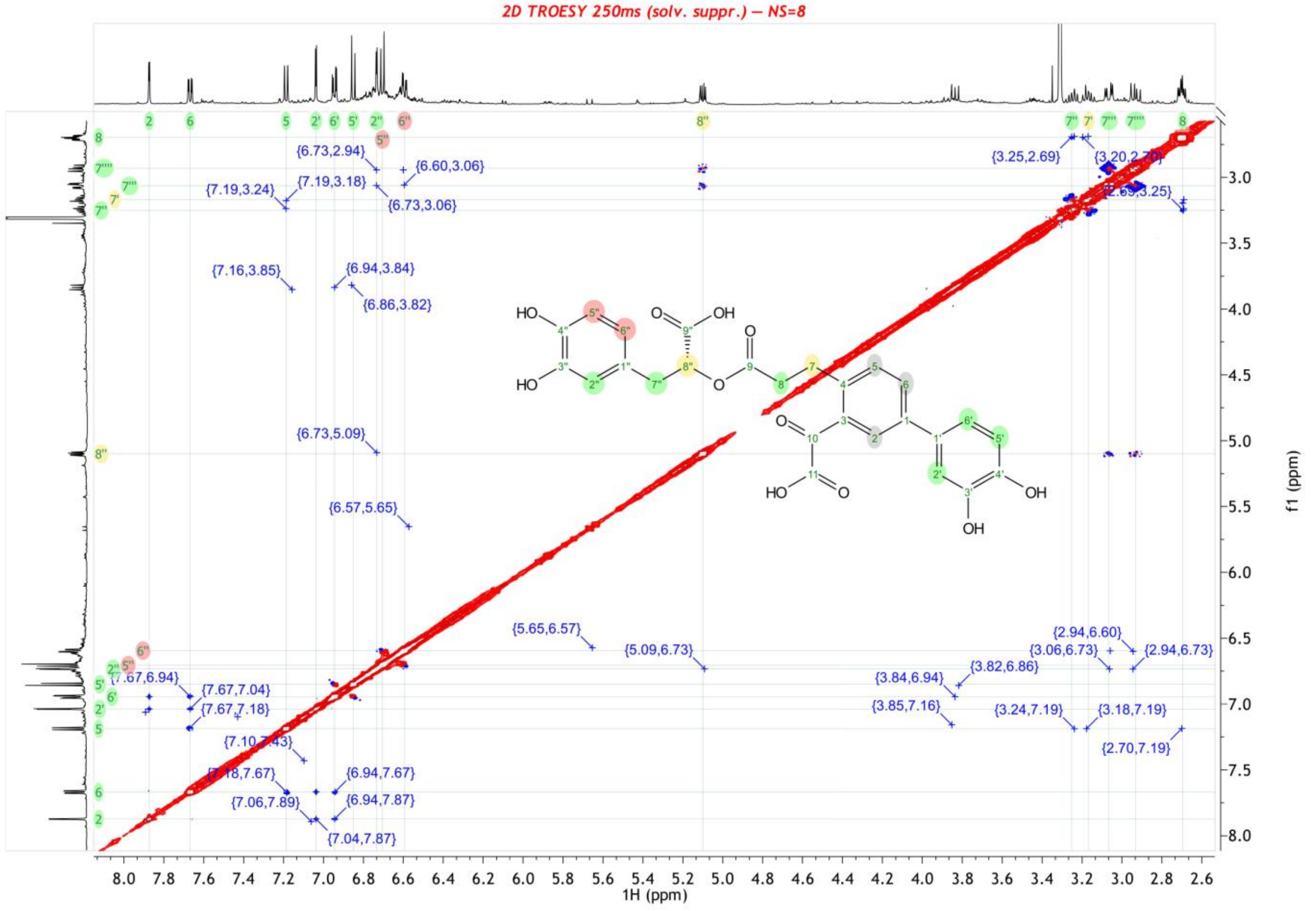

Figure 55S. ^{1}H-^{1}H TROESY (250 ms) NMR spectrum of compound 30

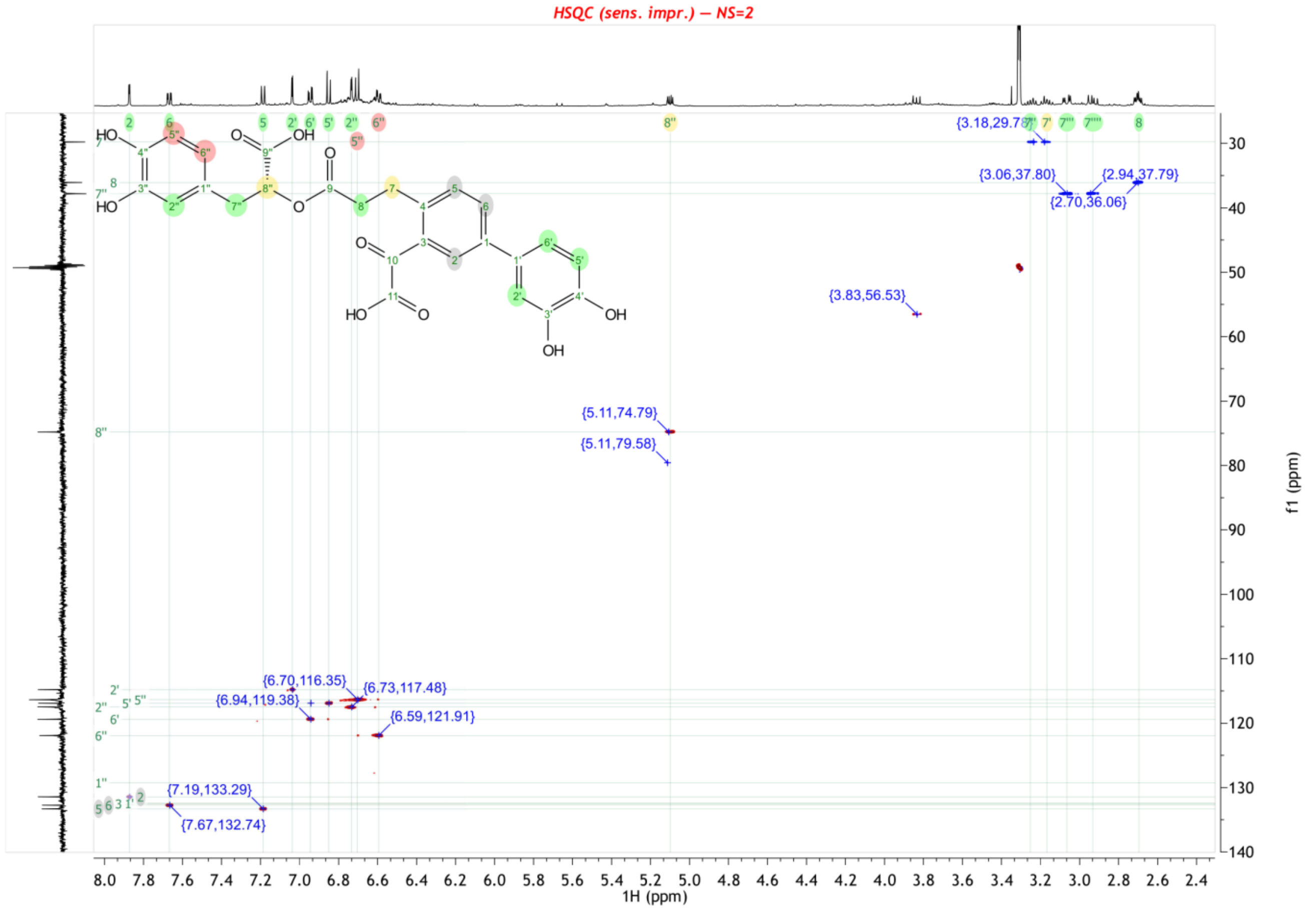

Figure 56S. ^{1}H-^{13}C HSQC NMR spectrum of compound 30

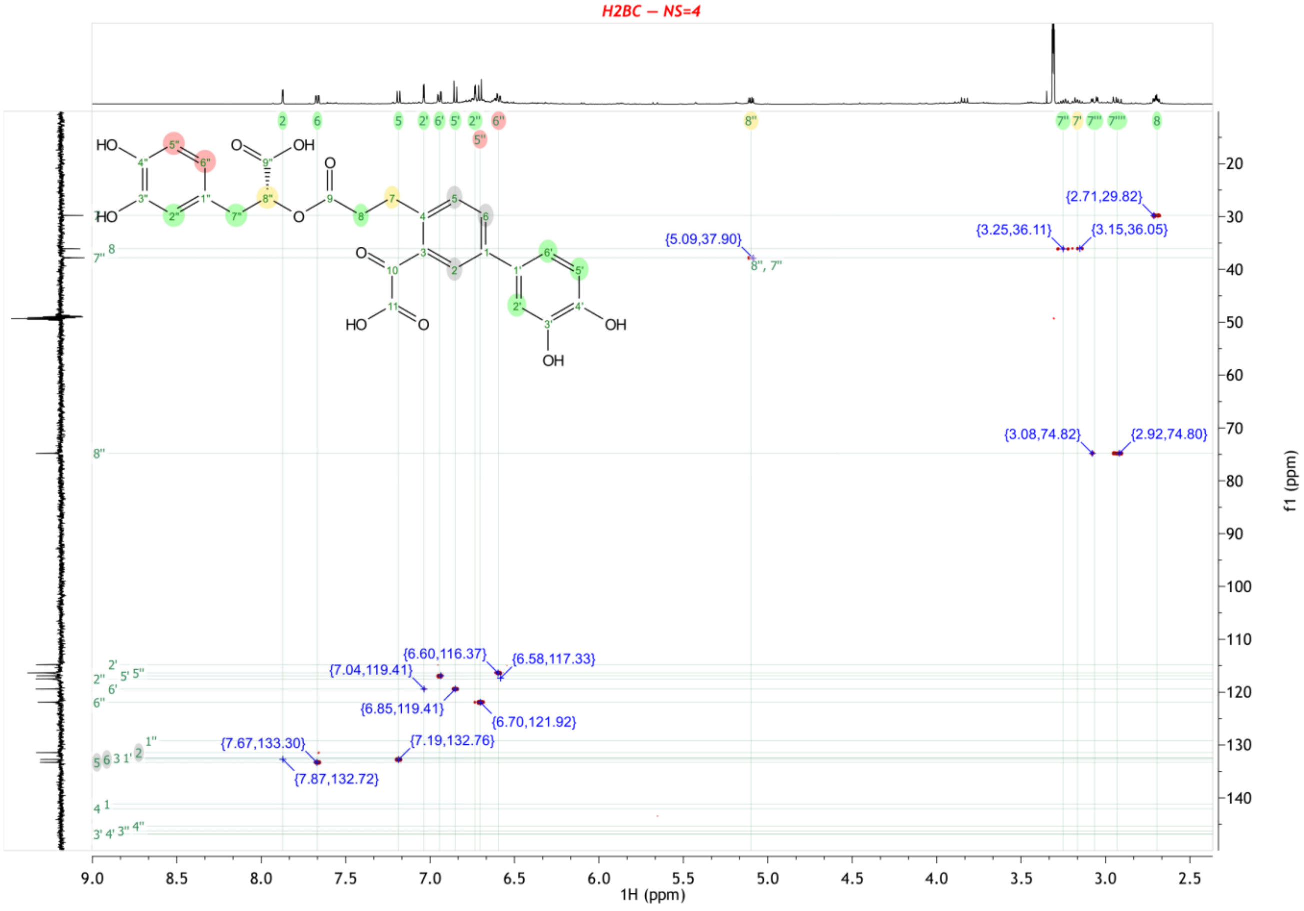

Figure 57S. ^{1}H-^{13}C H2BC NMR spectrum of compound 30

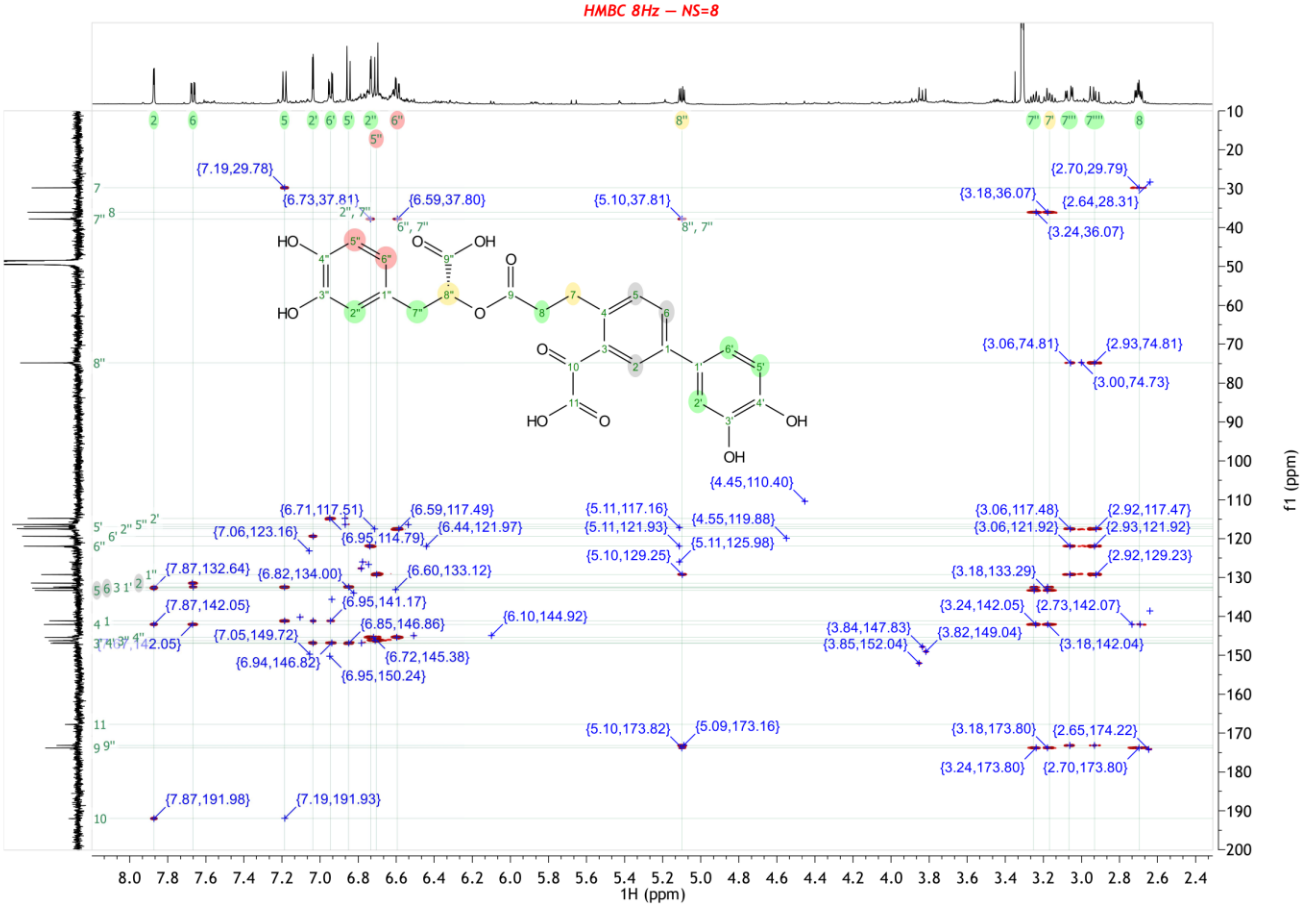

Figure 58S. ^{1}H-^{13}C HMBC (8 Hz) NMR spectrum of compound 30

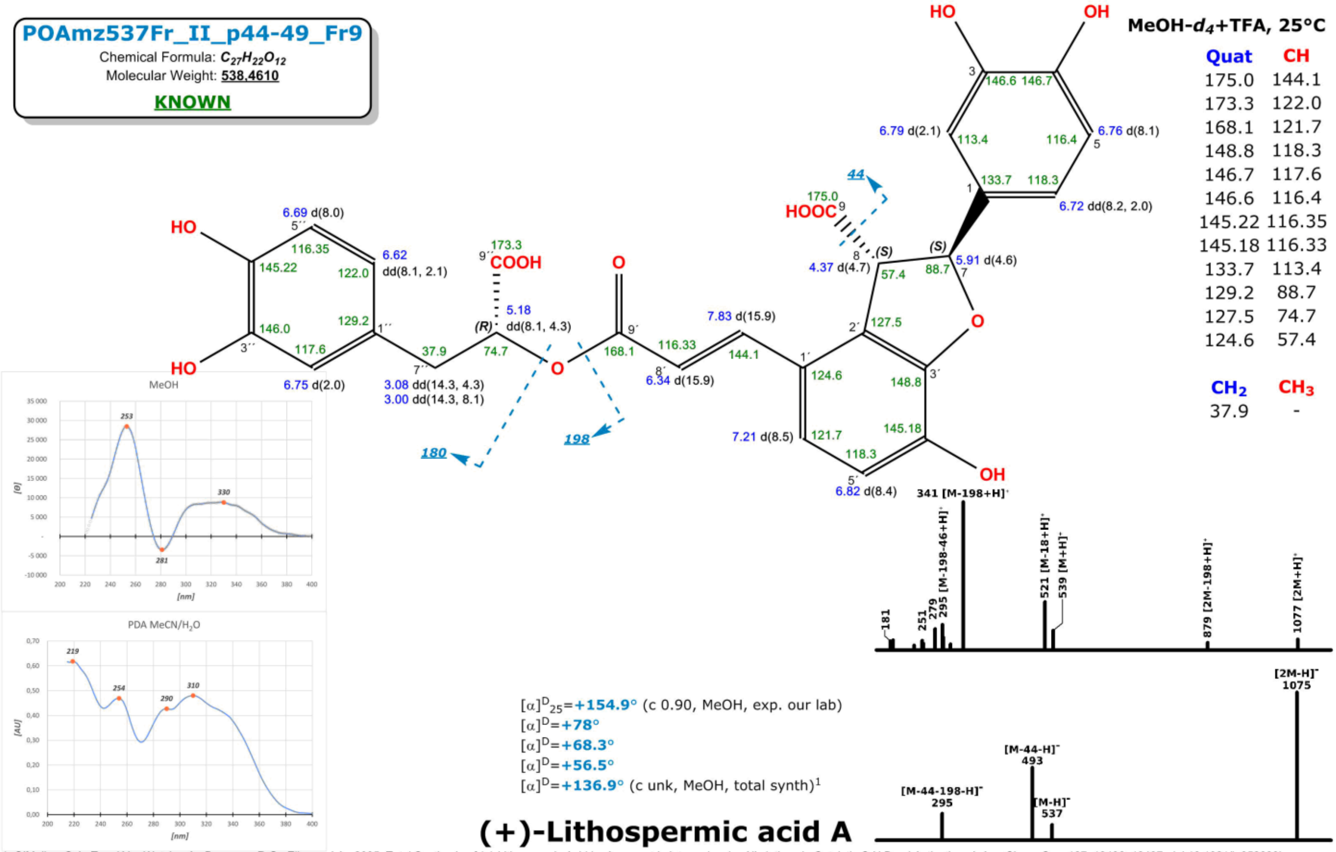

1. O'Malley, S.J., Tan, K.L., Watzke, A., Bergman, R.G., Ellman, J.A., 2005. Total Synthesis of (+)-Lithospermic Acid by Asymmetric Intramolecular Alkylation via Catalytic C-H Bond Activation. J. Am. Chem. Soc. 127, 13496–13497. doi:10.1021/ja052680h
2. Murata, T., Oyama, K., Fujiyama, M., Oobayashi, B., Umehara, K., Miyase, T., Yoshizaki, F., 2013. Diastereomers of lithospermic acid and lithospermic acid B from Monarda fistulosa and Lithospermum erythrorhizon. Fitoterapia 91, 51–59. doi:10.1016/j.fitote.2013.08.009

Figure 59S. ^{1}H (500 MHz) and ^{13}C (125 MHz) NMR data of compound 31 in CD_3OD, 25°C; on-line PDA UV spectrum in $MeCN/H_2O$

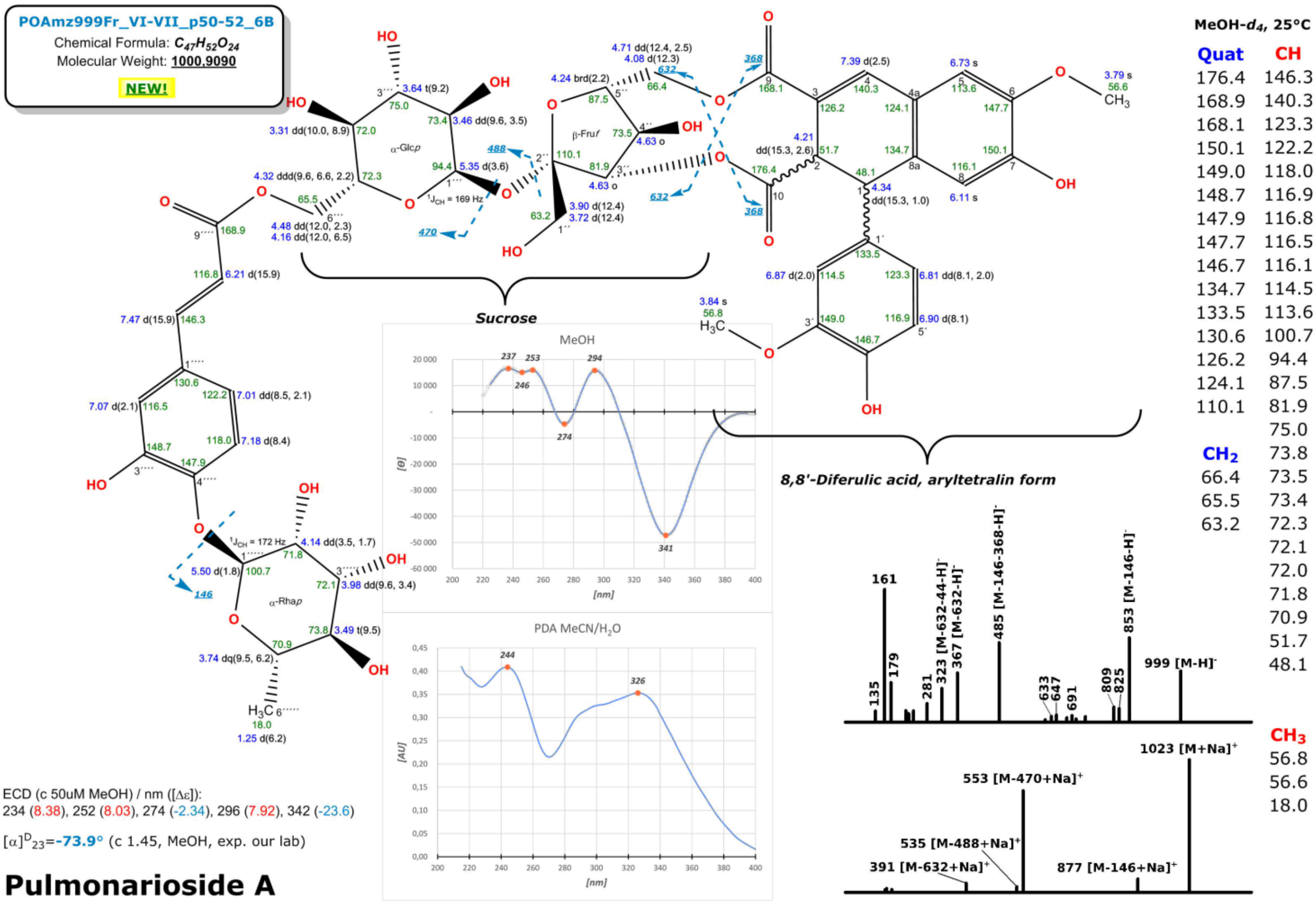

Figure 60S. ^{1}H (500 MHz) and ^{13}C (125 MHz) NMR data of compound 32 in CD_3OD, 25°C; on-line PDA UV spectrum in $MeCN/H_2O$

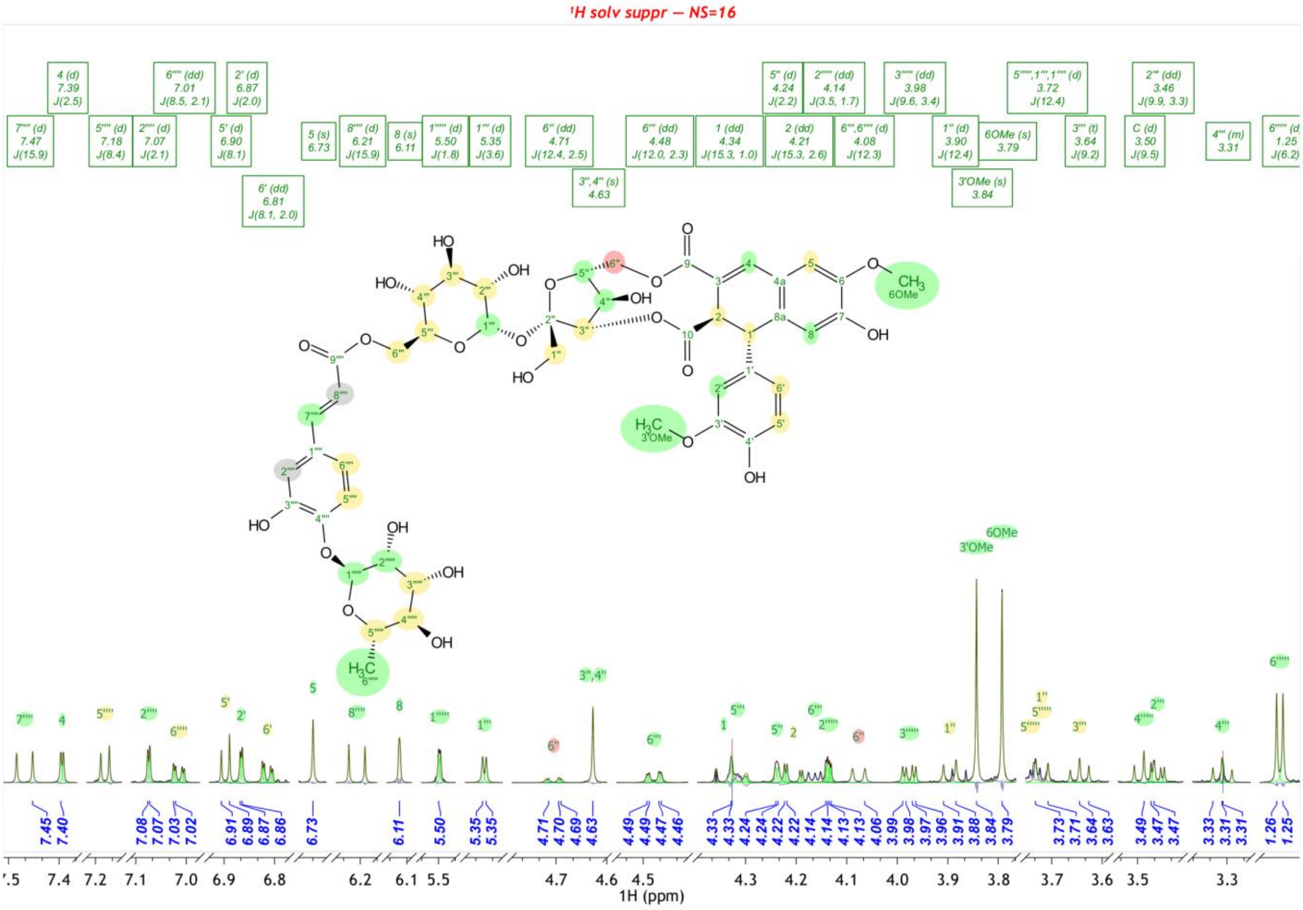

Figure 61S. ^{1}H NMR spectrum of compound 32

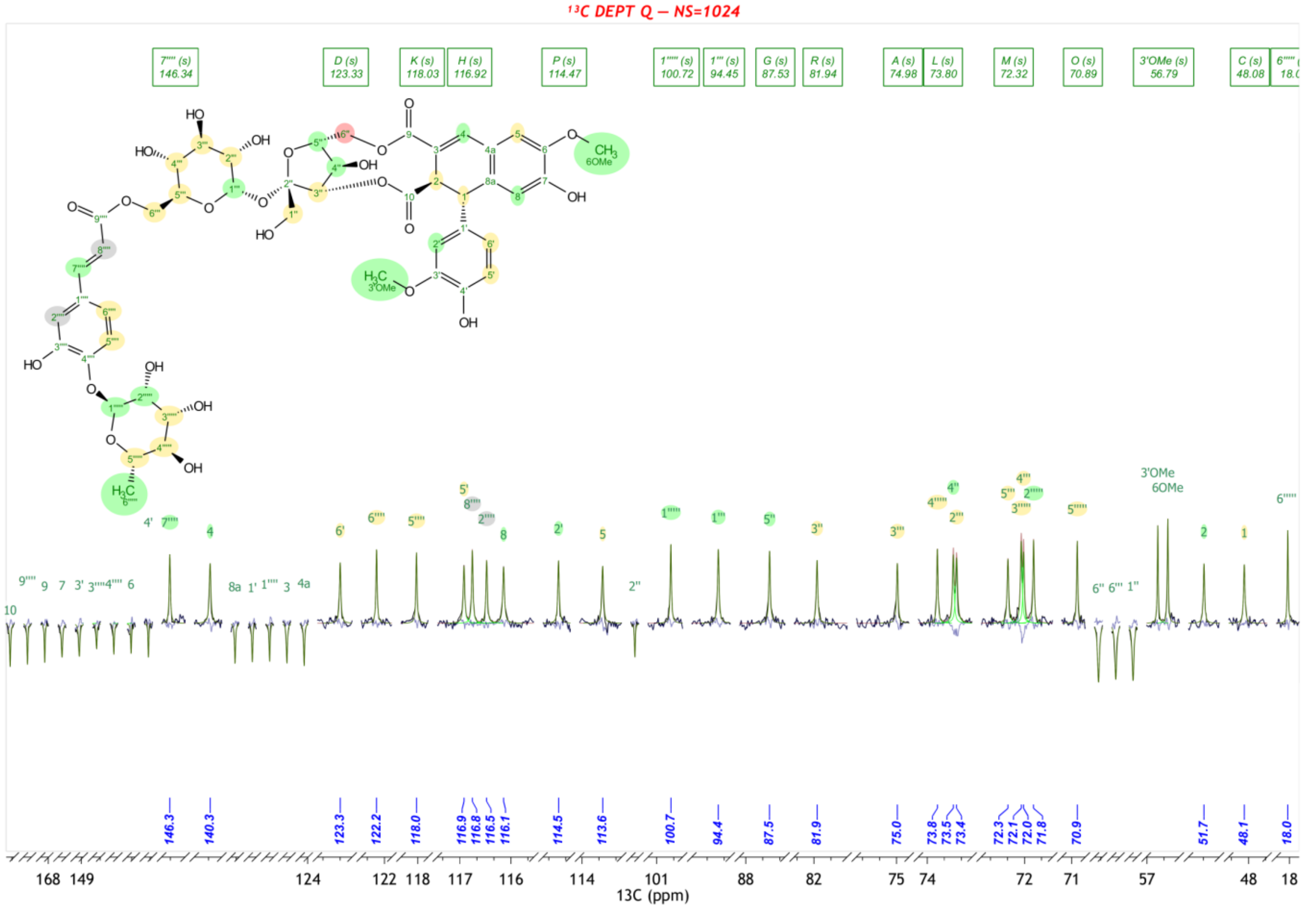

Figure 62S. ^{13}C DEPTQ NMR spectrum of compound 32

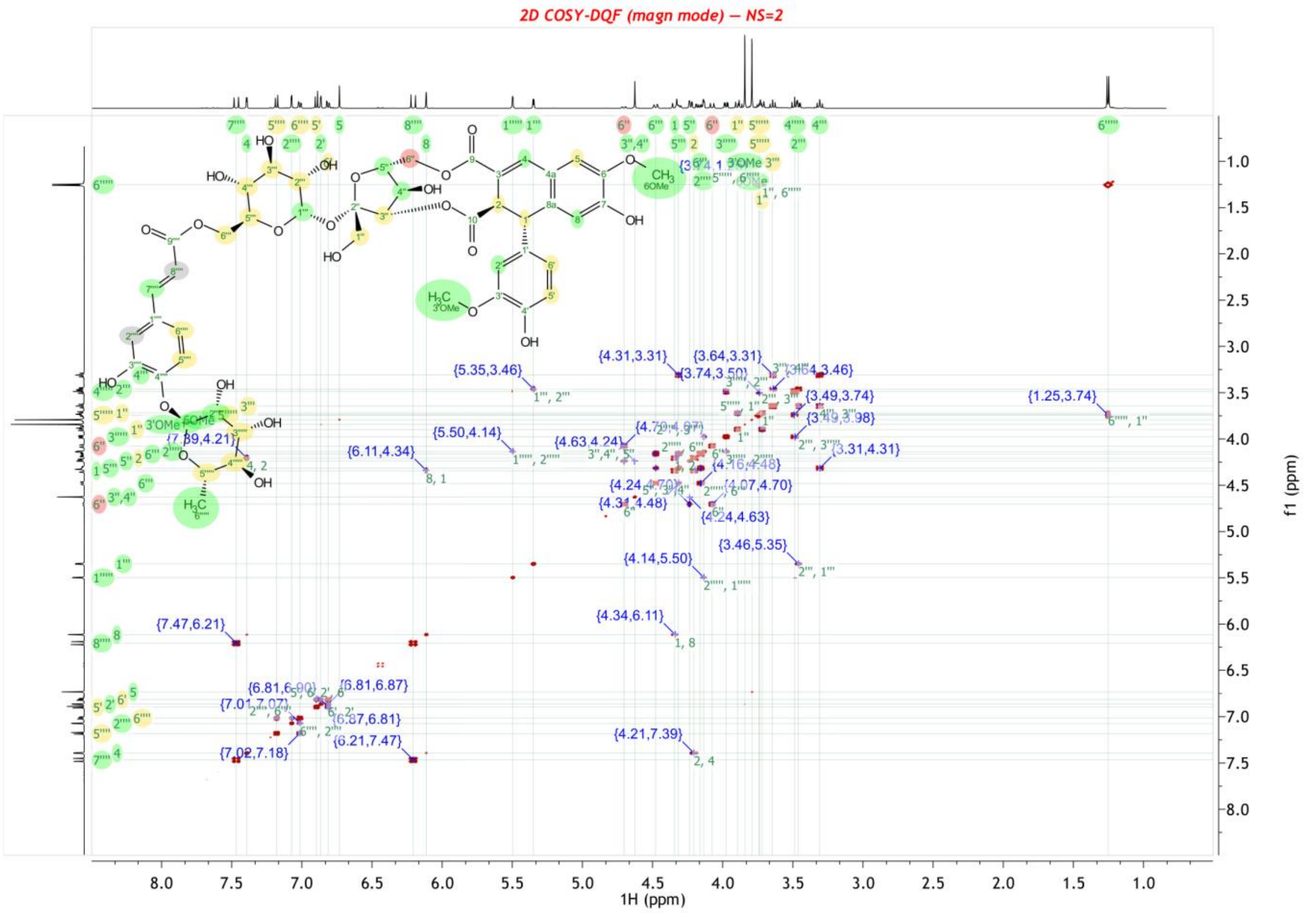

Figure 63S. ^1H-^1H COSY NMR spectrum of compound 32

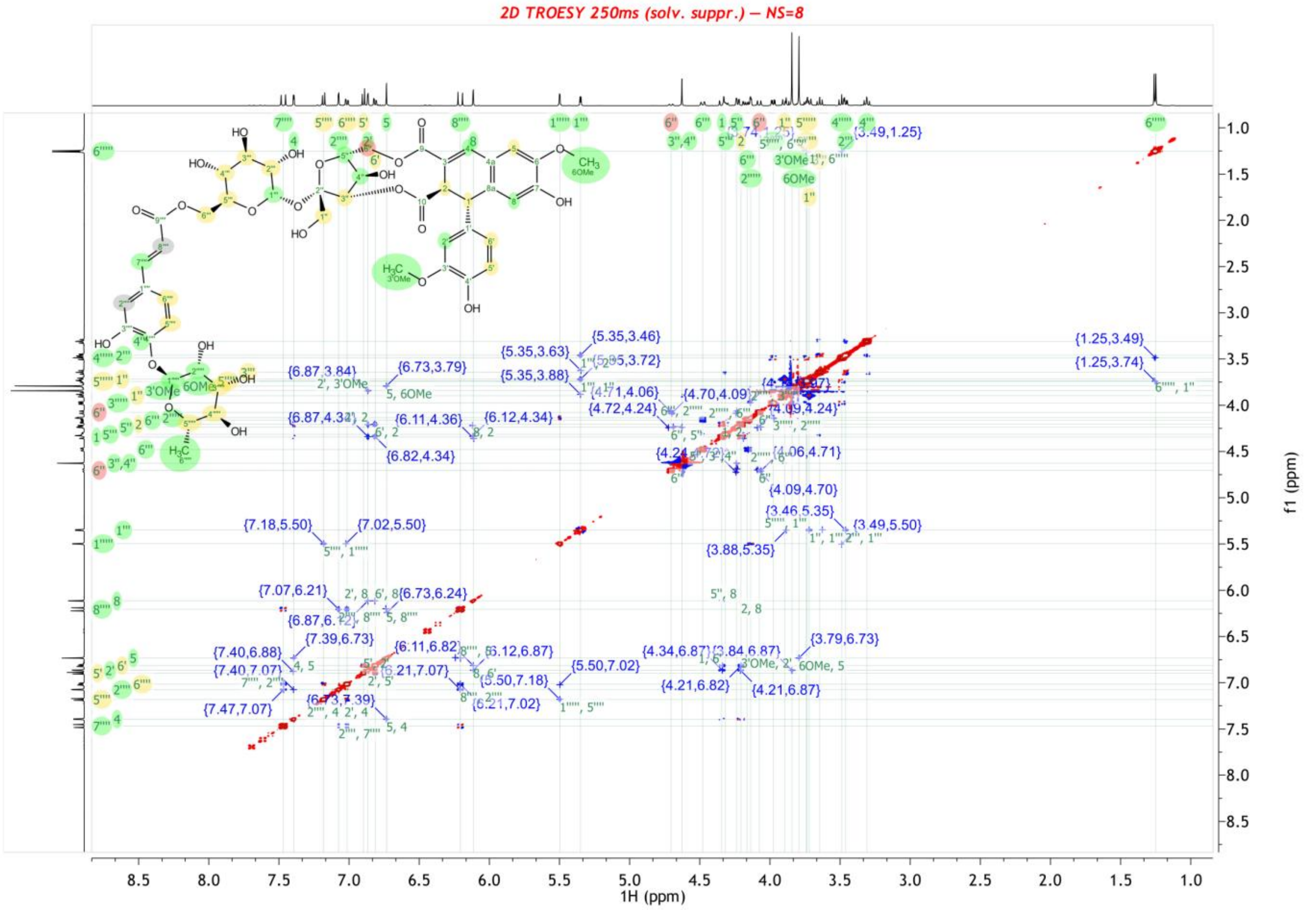

Figure 64S. ^1H-^1H TROESY (250 ms) NMR spectrum of compound 32

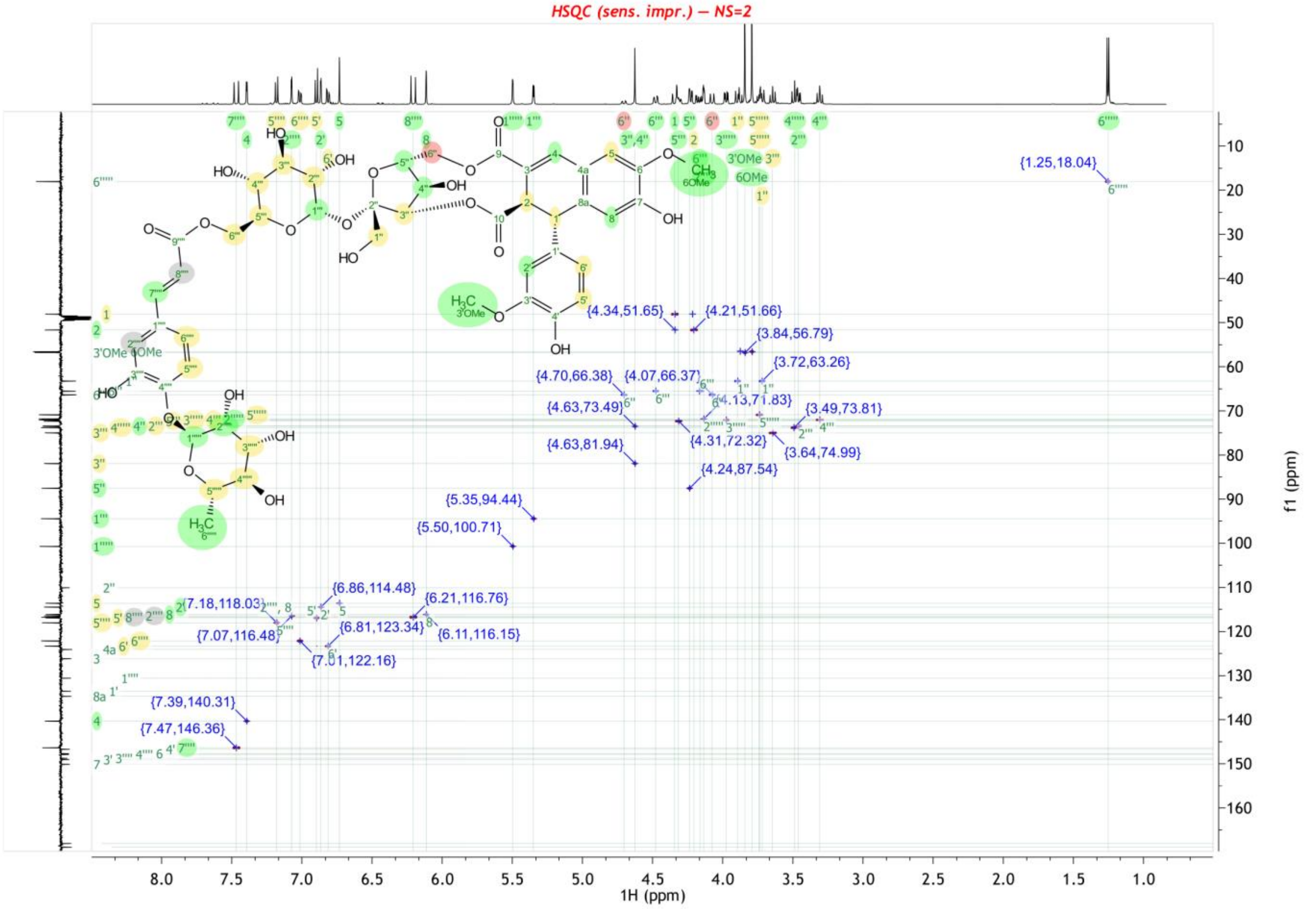

Figure 65S. ^{1}H-^{13}C HSQC NMR spectrum of compound 32

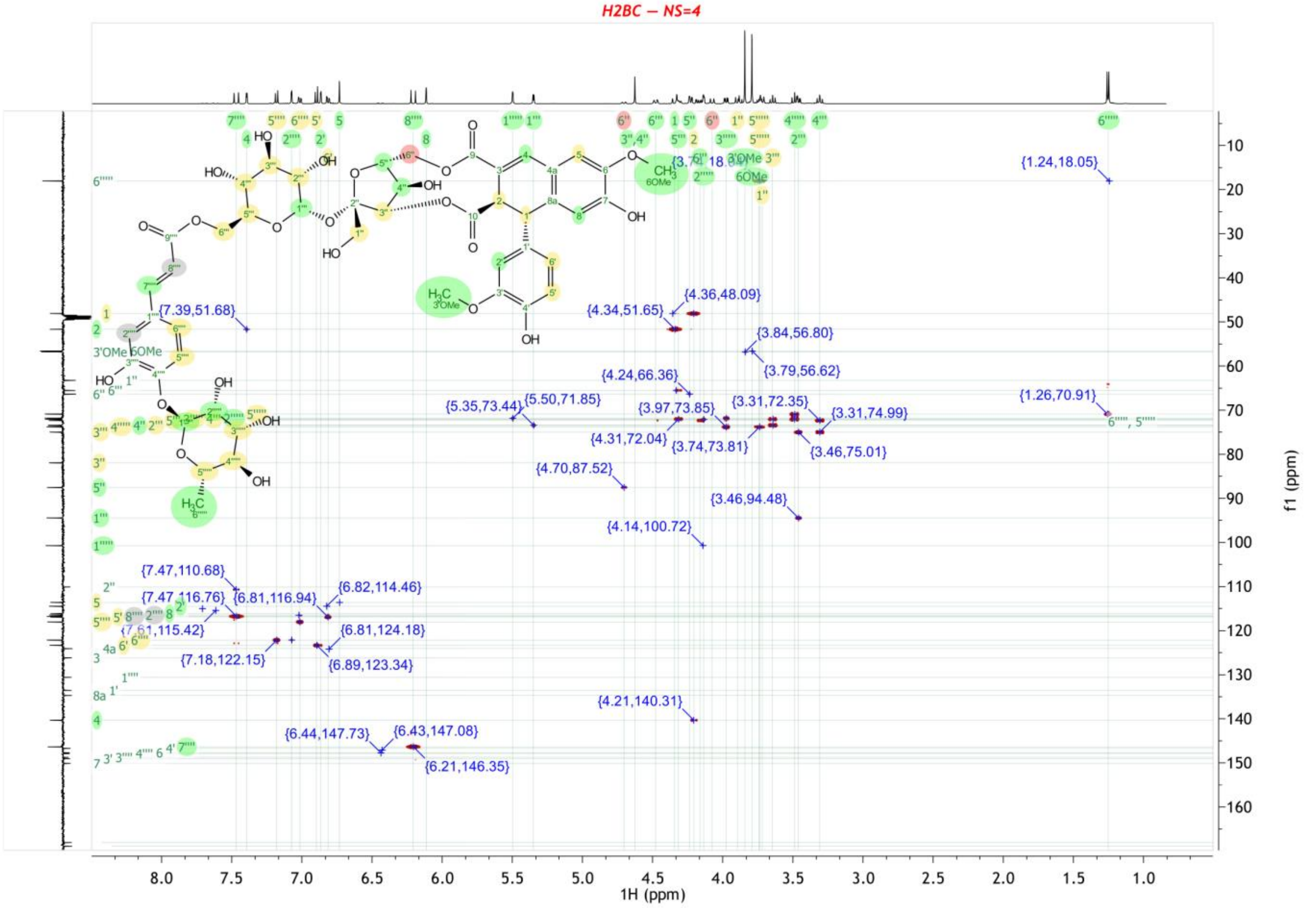

Figure 66S. ^{1}H-^{13}C H2BC NMR spectrum of compound 32

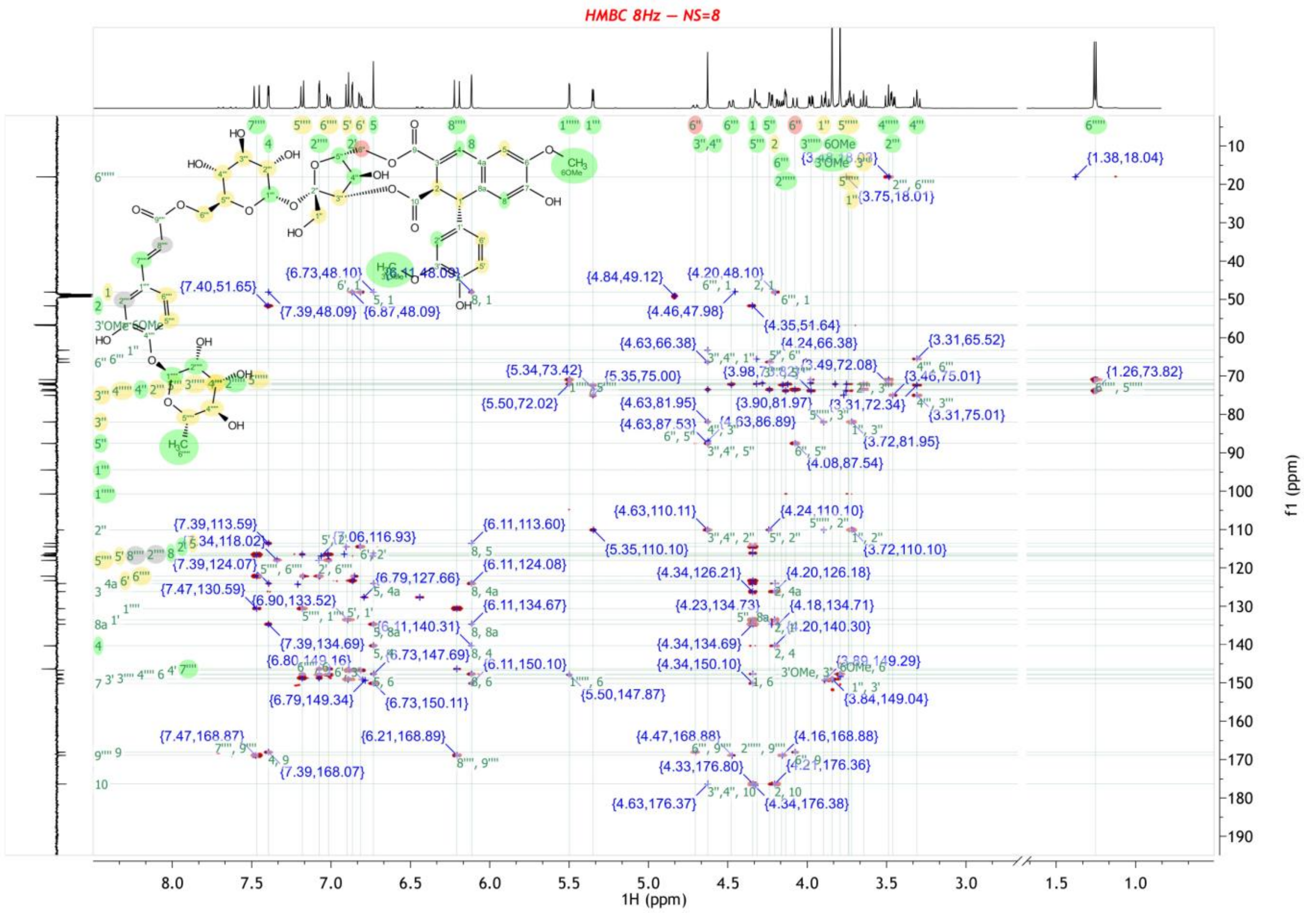

Figure 67S. ^{1}H-^{13}C HMBC (8 Hz) NMR spectrum of compound 32

POAmz537Fr_III_p46-53_6
Chemical Formula: $C_{27}H_{22}O_{12}$
Molecular Weight: 538.4610
KNOWN

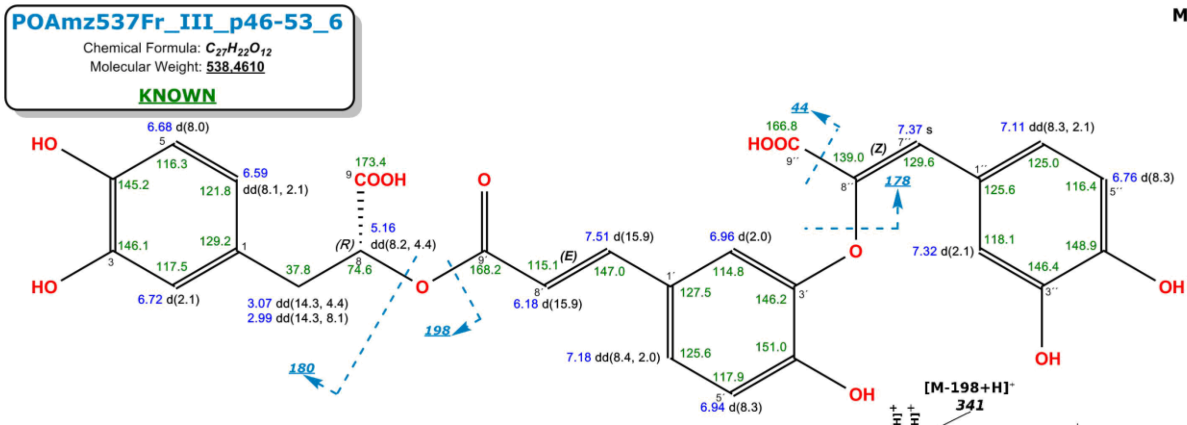

MeOH-d_4, 25°C

Quat	CH
173.4	147.0
168.2	129.6
166.8	125.6
151.0	125.0
148.9	121.8
146.4	118.1
146.2	117.9
146.1	117.5
145.2	116.4
139.0	116.3
129.2	115.1
127.5	114.8
125.8	74.6

CH2	CH3
37.8	-

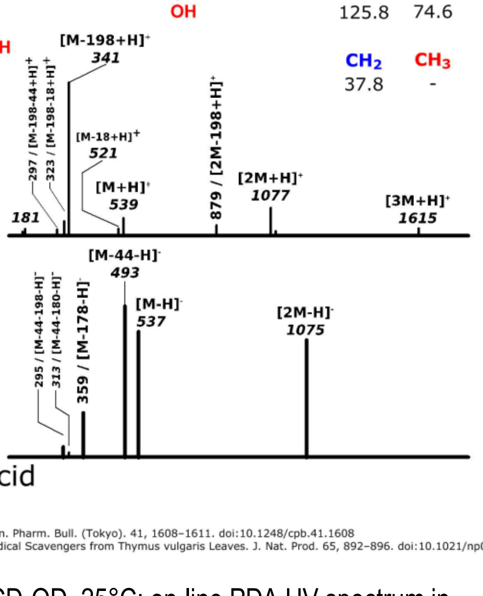

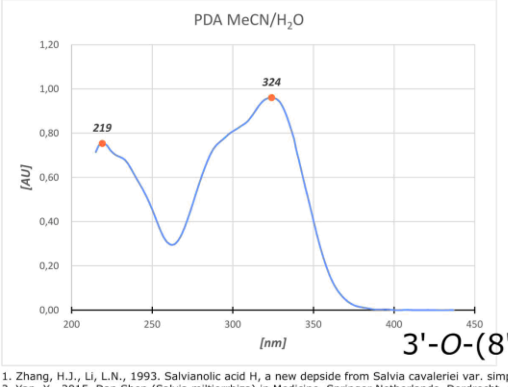

$[\alpha]^{D}_{25}$=**+30.1°** (c 1.13, MeOH, exp. our lab)
$[\alpha]^{D}$=**+63°** (c unk, EtOH)
$[\alpha]^{D}_{22}$=**+40.3°** (c 1.2, MeOH)[4]
ECD (c MeOH) / nm ([Δε]):
221 (0.9), 233 (-1.4), 252 (1.8), 275 (1.0), 297 (8.0), 327 (7.9)[4]

Salvianolic acid H

3'-*O*-(8''-*Z*-caffeoyl)-rosmarinic acid

1. Zhang, H.J., Li, L.N., 1993. Salvianolic acid H, a new depside from Salvia cavaleriei var. simplicifolia. Chinese Chem. Lett 4, 501–504.
2. Yan, X., 2015. Dan Shen (Salvia miltiorrhiza) in Medicine. Springer Netherlands, Dordrecht. doi:10.1007/978-94-017-9463-3
3. AGATA, I., KUSAKABE, H., HATANO, T., NISHIBE, S., OKUDA, T., 1993. Melitric Acids A and B, New Trimeric Caffeic Acid Derivatives from Melissa officinalis. Chem. Pharm. Bull. (Tokyo). 41, 1608–1611. doi:10.1248/cpb.41.1608
4. Dapkevicius, A., van Beek, T.A., Lelyveld, G.P., van Veldhuizen, A., de Groot, A., Linssen, J.P.H., Venskutonis, R., 2002. Isolation and Structure Elucidation of Radical Scavengers from Thymus vulgaris Leaves. J. Nat. Prod. 65, 892–896. doi:10.1021/np010636j

Figure 68S. ^{1}H (500 MHz) and ^{13}C (125 MHz) NMR data of compound 33 in CD_3OD, 25°C; on-line PDA UV spectrum in $MeCN/H_2O$

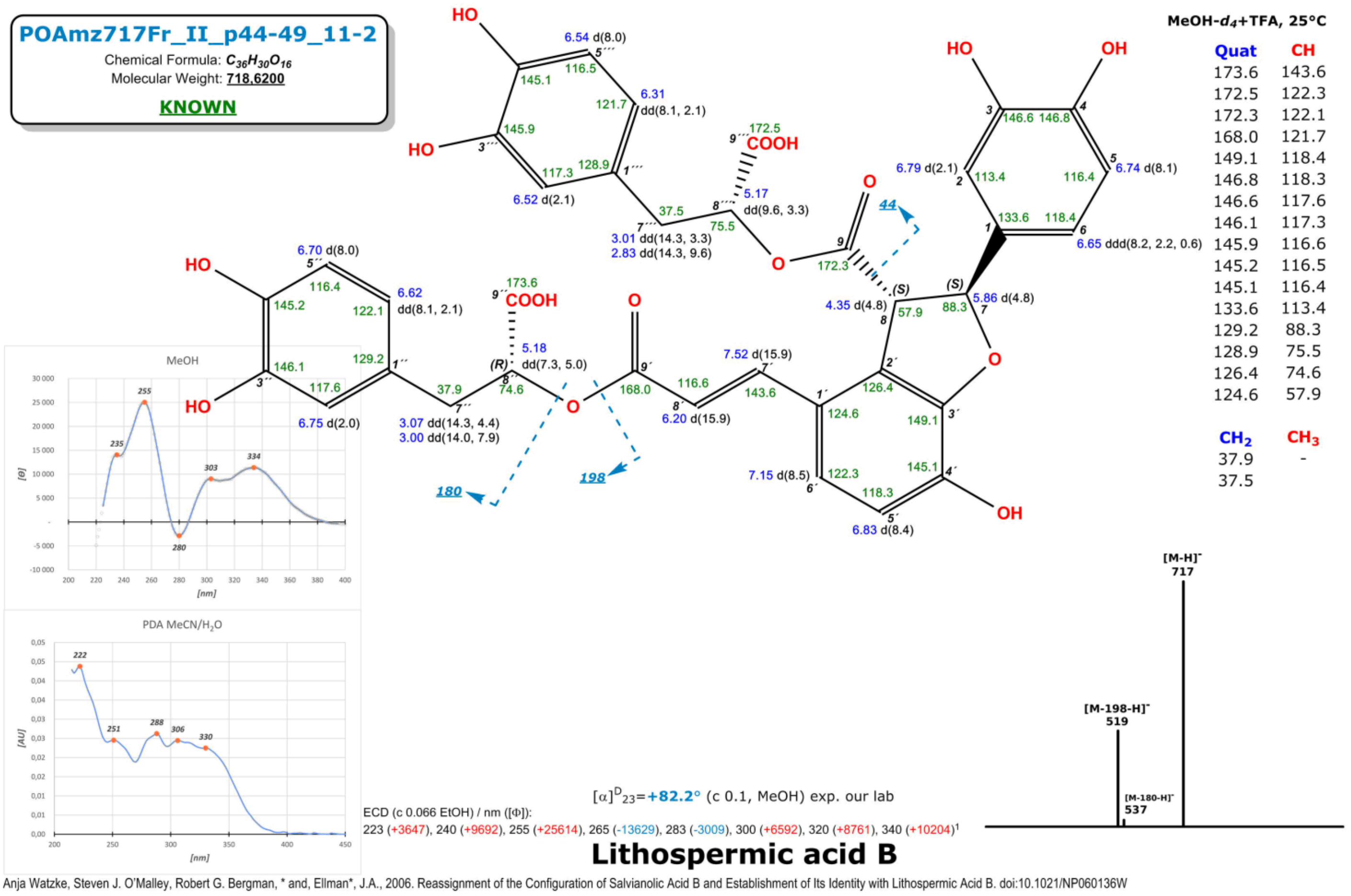

Figure 69S. ^{1}H (500 MHz) and ^{13}C (125 MHz) NMR data of compound 34 in CD_3OD, 25°C; on-line PDA UV spectrum in $MeCN/H_2O$; ECD spectrum in MeOH

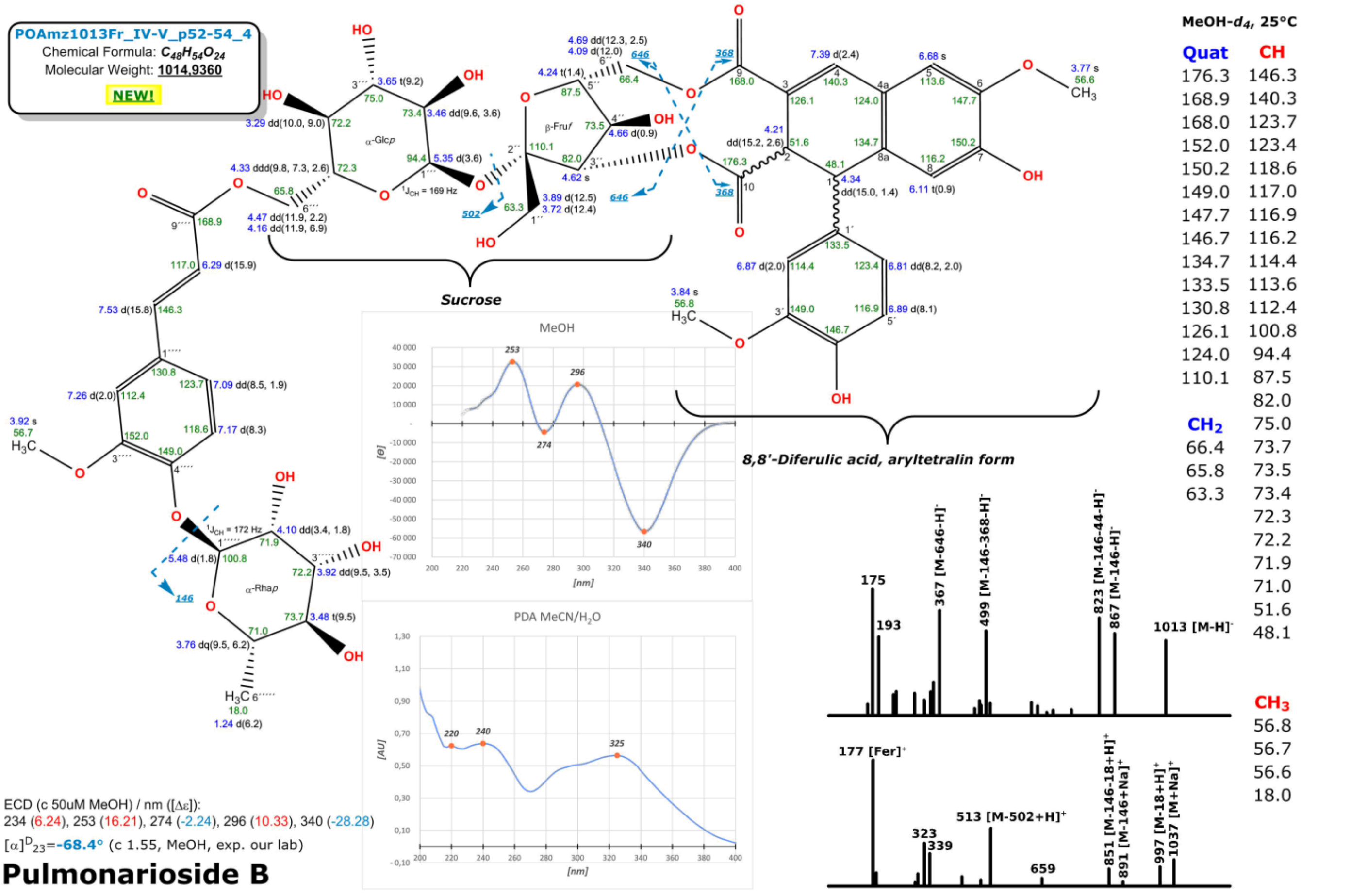

Figure 70S. ^{1}H (500 MHz) and ^{13}C (125 MHz) NMR data of compound 35 in CD_3OD, 25°C; on-line PDA UV spectrum in $MeCN/H_2O$; ECD spectrum in MeOH

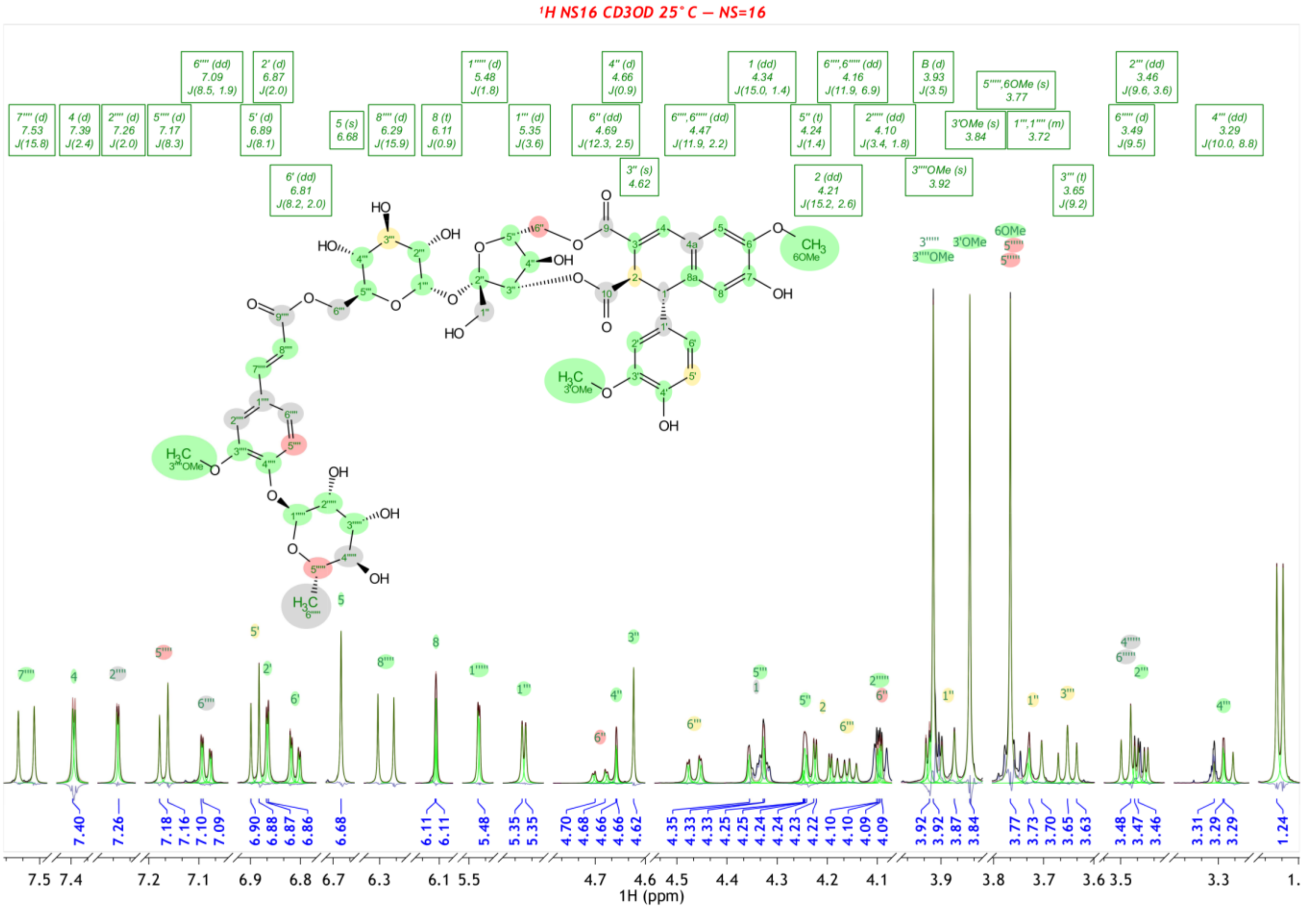

Figure 71S. ^{1}H NMR spectrum of compound 35

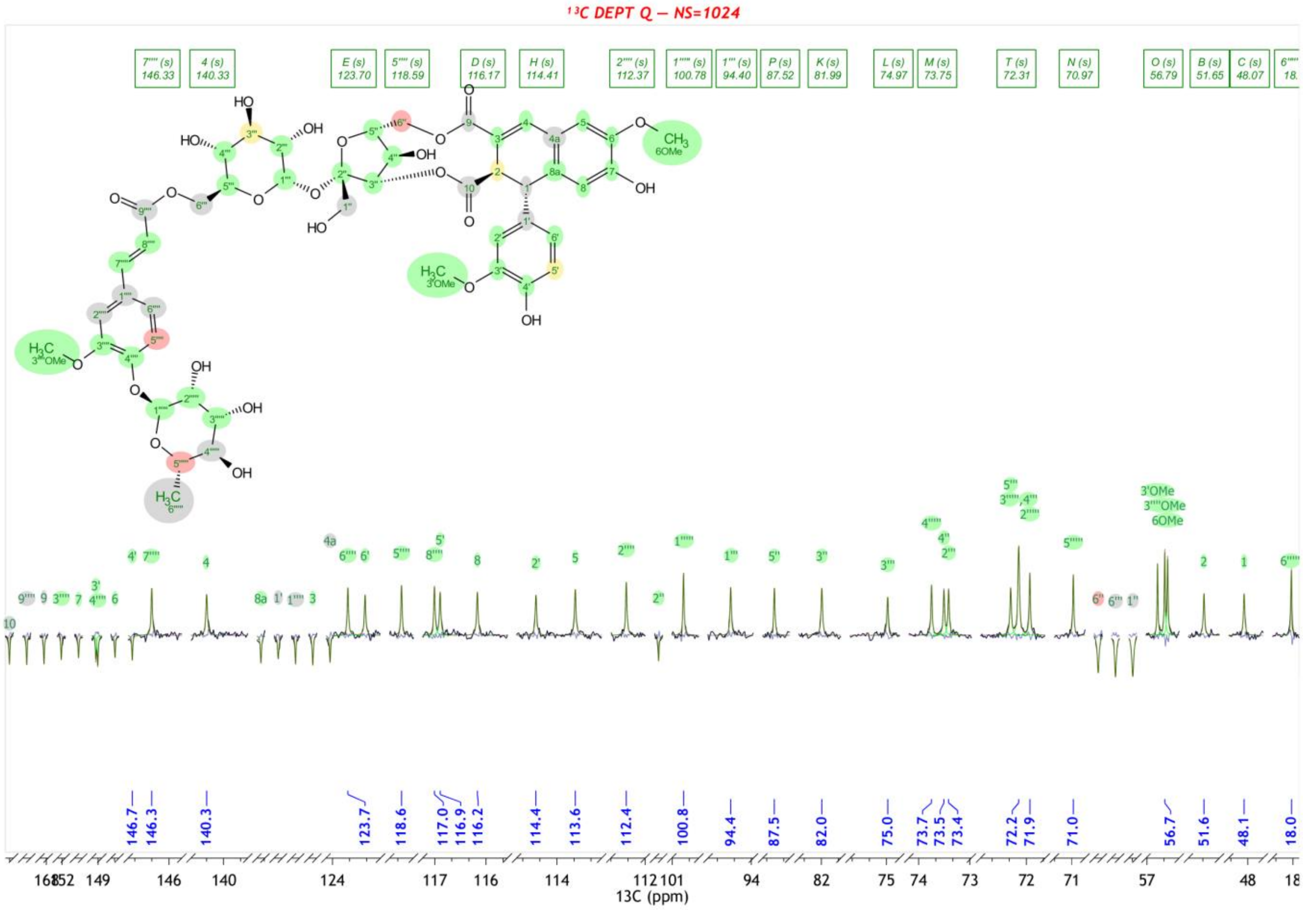

Figure 72S. ^{13}C DEPTQ NMR spectrum of compound 35

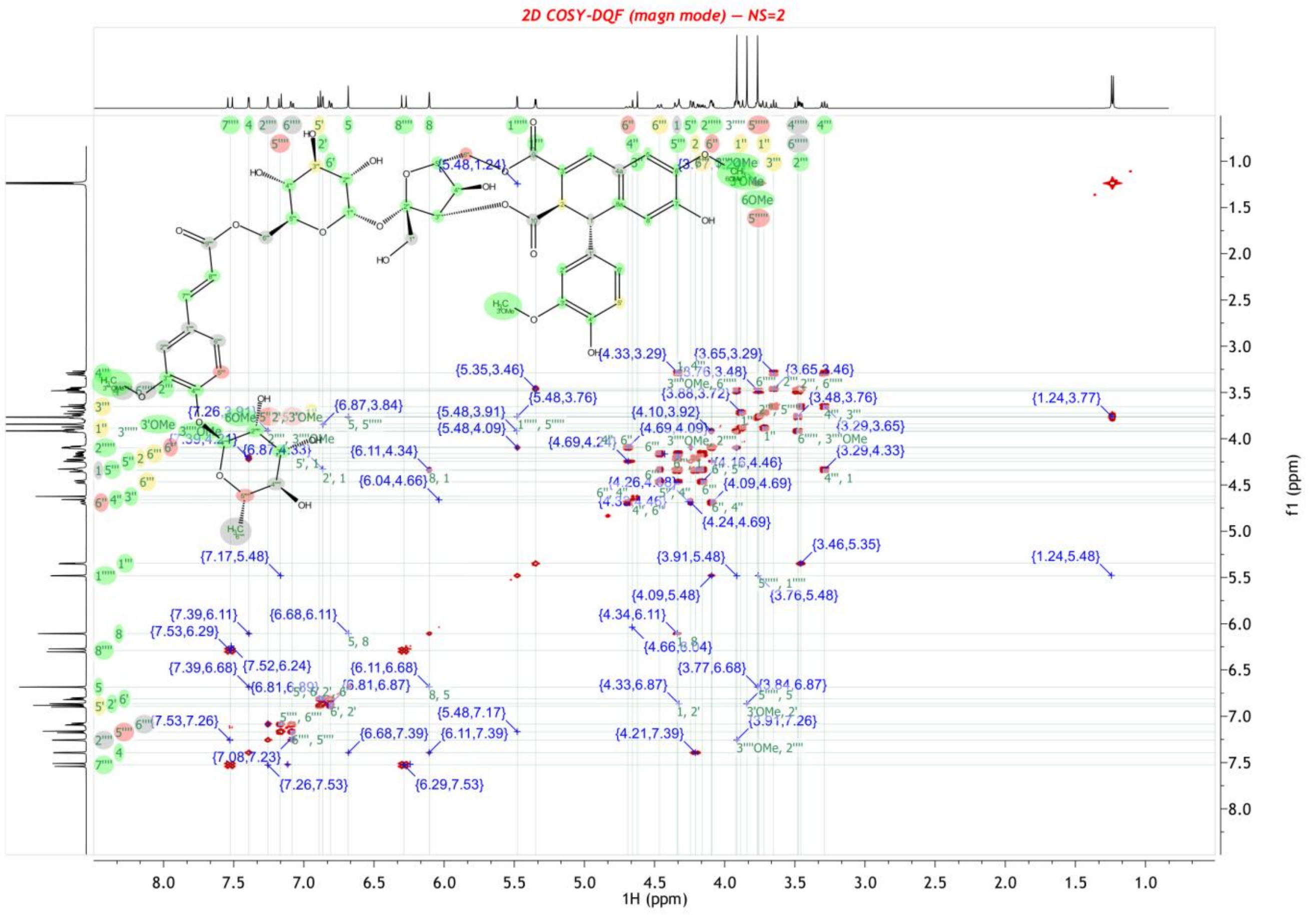

Figure 73S. ^{1}H-^{1}H COSY NMR spectrum of compound 35

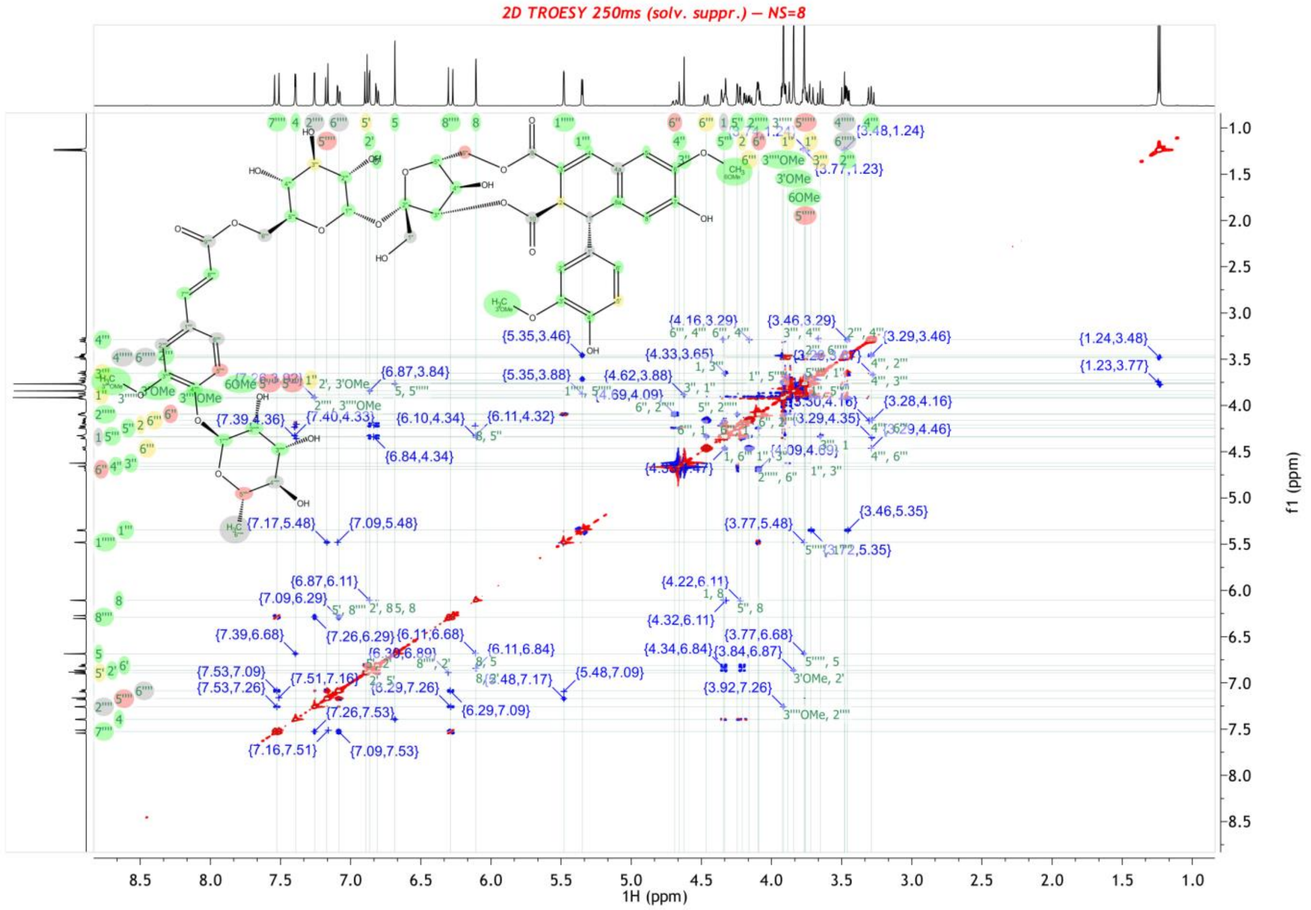

Figure 74S. ^{1}H-^{1}H TROESY (250 ms) NMR spectrum of compound 35

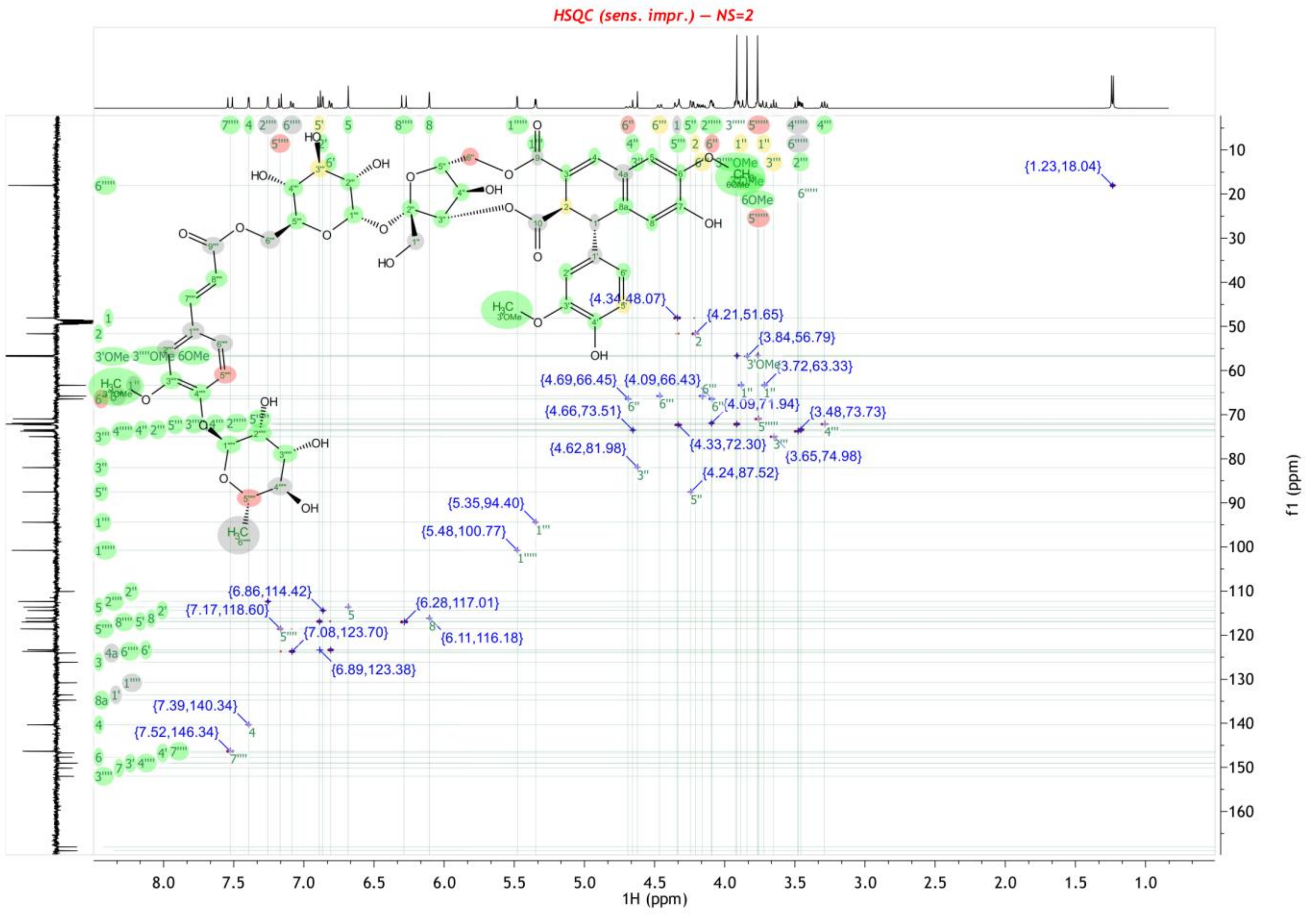

Figure 75S. ^{1}H-^{13}C HSQC NMR spectrum of compound 35

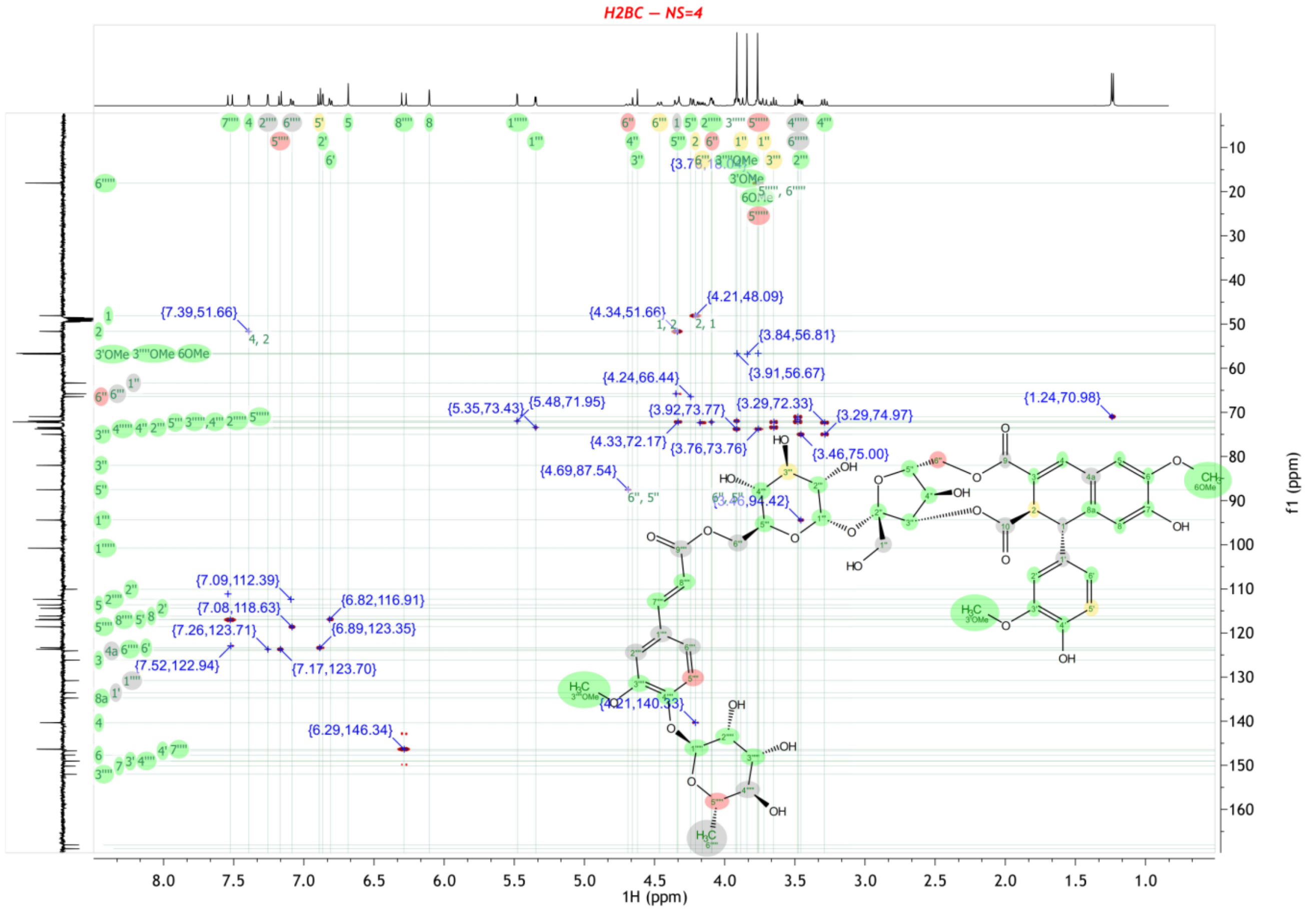

Figure 76S. ^{1}H-^{13}C H2BC NMR spectrum of compound 35

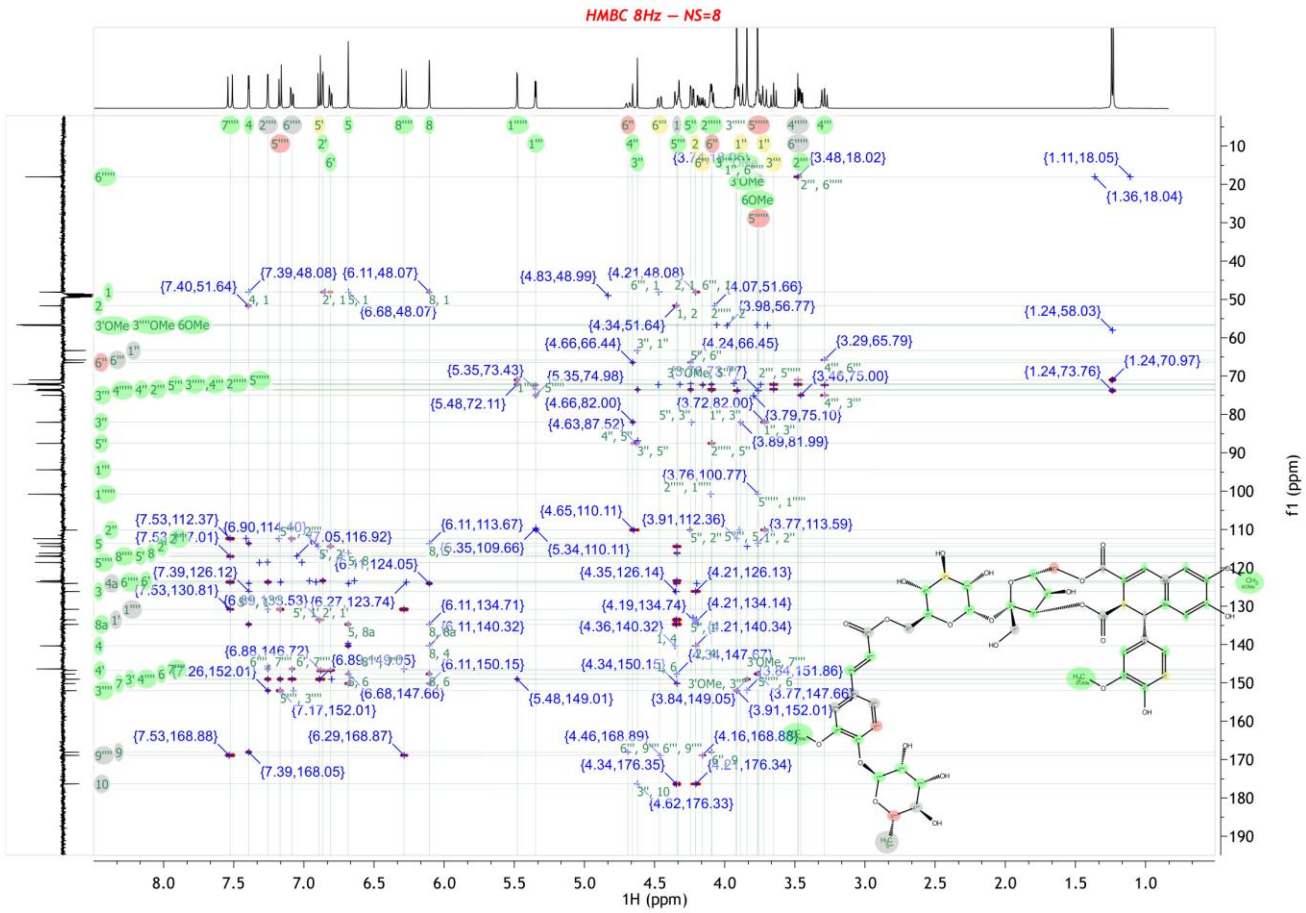

Figure 77S. ^{1}H-^{13}C HMBC (8 Hz) NMR spectrum of compound 35

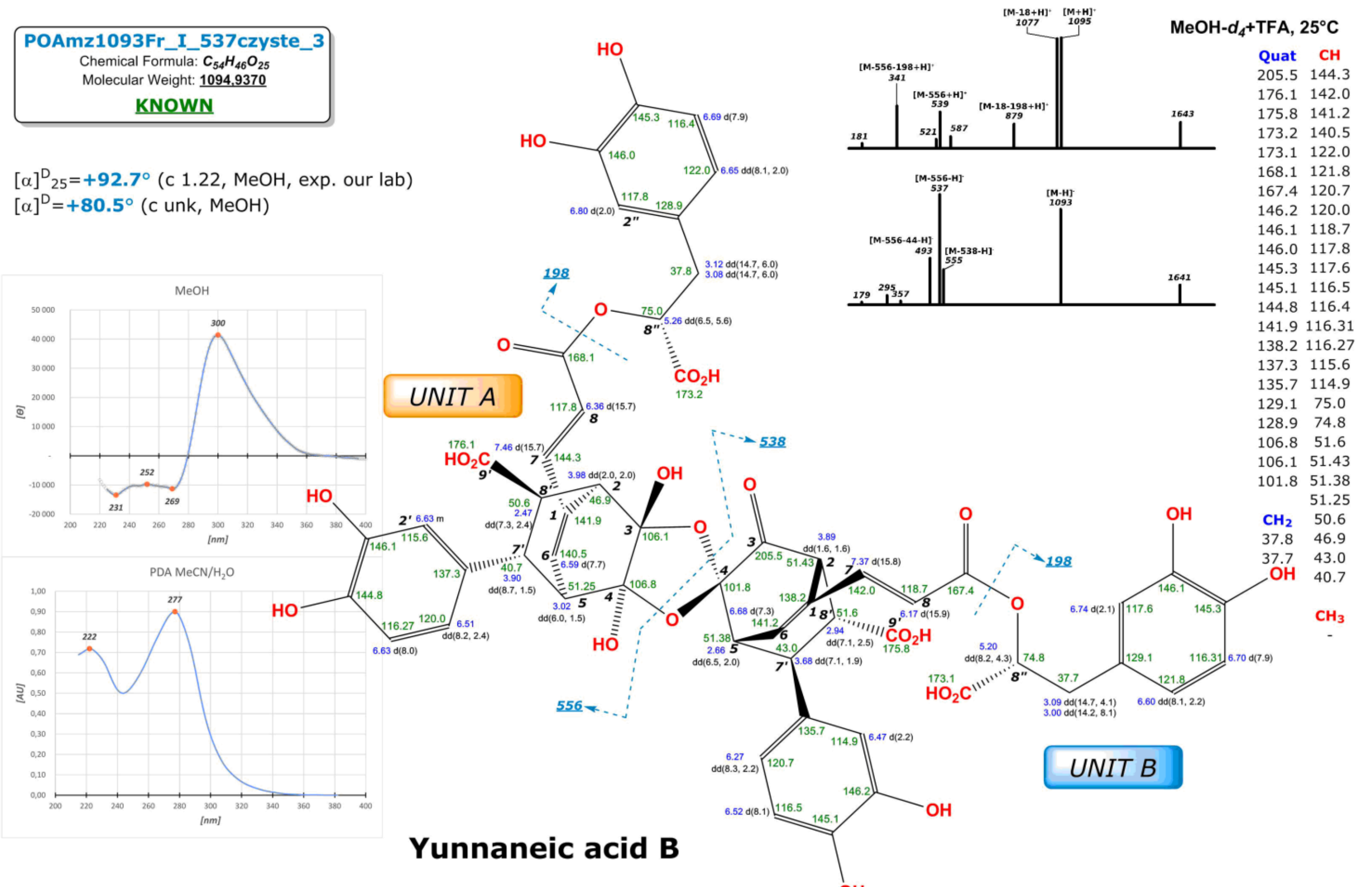

1. Tanaka, T., Nishimura, A., Kouno, I., Nonaka, G.I., Young, T.J., 1996. Isolation and characterization of yunnaneic acids A-D, four novel caffeic acid metabolites from Salvia yunnanensis. J. Nat. Prod. 59, 843–849. doi:10.1021/np960425s
2. Yan, X., 2015. Dan Shen (Salvia miltiorrhiza) in Medicine. Springer Netherlands, Dordrecht. doi:10.1007/978-94-017-9463-3

Figure 78S. ^{1}H (500 MHz) and ^{13}C (125 MHz) NMR data of compound 36 in CD_3OD, 25°C; on-line PDA UV spectrum in $MeCN/H_2O$; ECD spectrum in MeOH

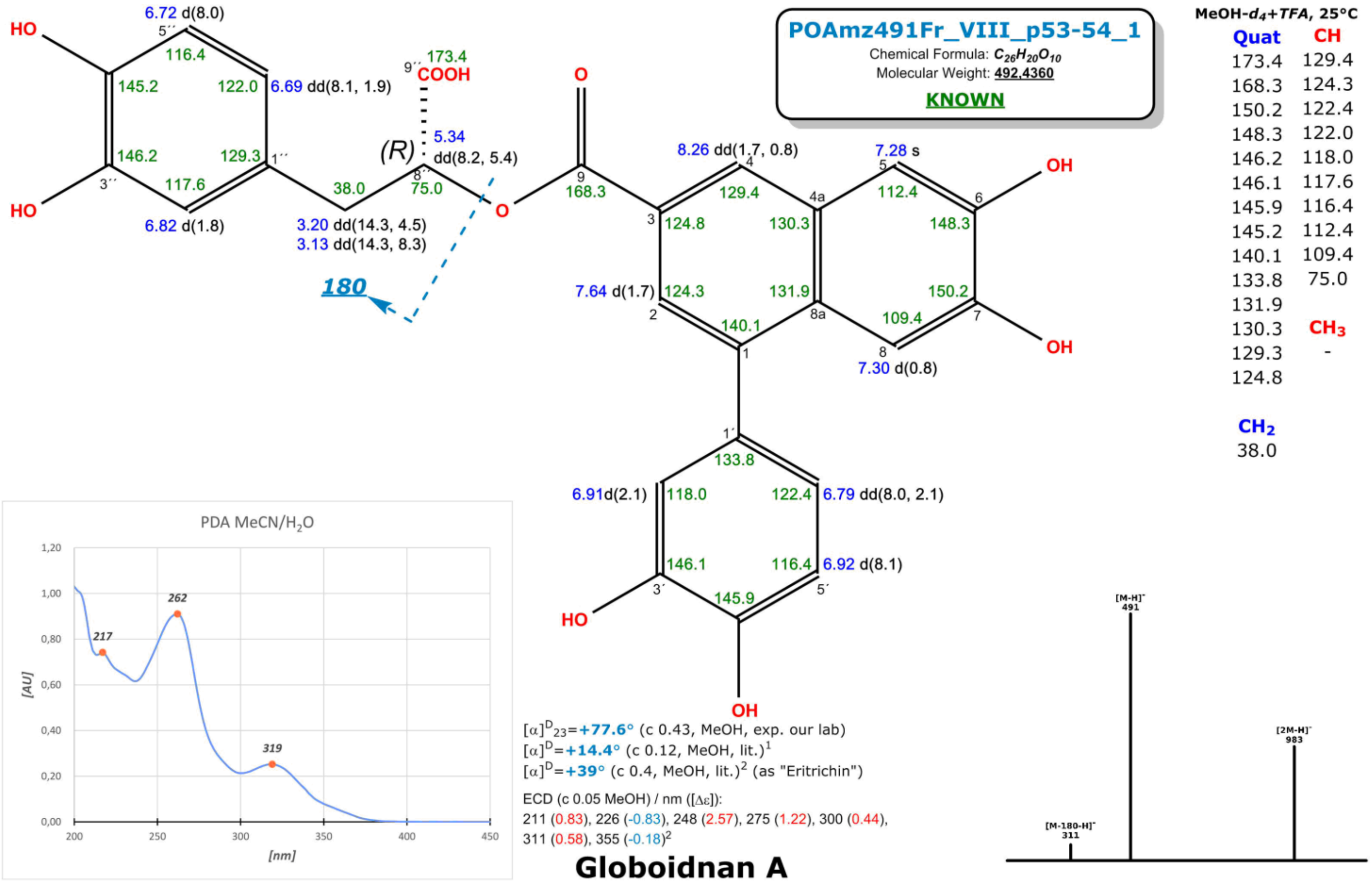

1. OVENDEN, S., YU, J., SANWAN, S., SBERNA, G., MURRAYTAIT, R., RHODES, D., COX, S., COATES, J., WALSH, N., MEURERGRIMES, B., 2004. Globoidnan A: a lignan from Eucalyptus globoidea inhibits HIV integrase. Phytochemistry 65, 3255–3259. doi:10.1016/j.phytochem.2004.10.006

2. Fedoreyev, S.A., Veselova, M. V., Krivoschekova, O.E., Mischenko, N.P., Denisenko, V.A., Dmitrenok, P.S., Glazunov, V.P., Bulgakov, V.P., Tchernoded, G.K., Zhuravlev, Y.N., 2005. Caffeic Acid Metabolites from Eritrichium sericeum Cell Cultures. Planta Med. 71, 446–451. doi:10.1055/s-2005-864141

Figure 79S. ^{1}H (500 MHz) and ^{13}C (125 MHz) NMR data of compound 37 in CD_3OD, 25°C; on-line PDA UV spectrum in MeCN/H_2O

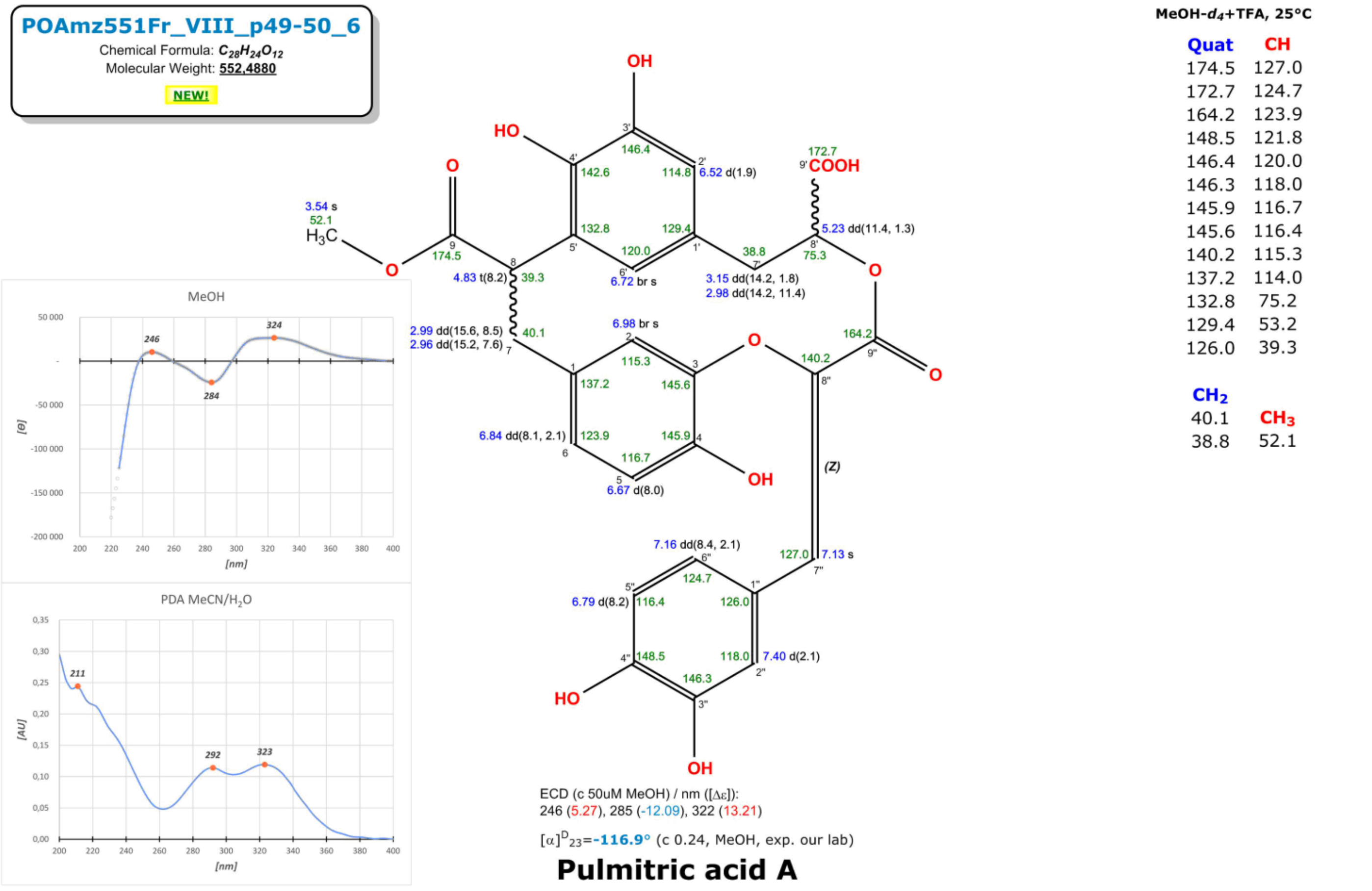

Figure 80S. ^{1}H (500 MHz) and ^{13}C (125 MHz) NMR data of compound 38 in CD_3OD, 25°C; on-line PDA UV spectrum in MeCN/H_2O

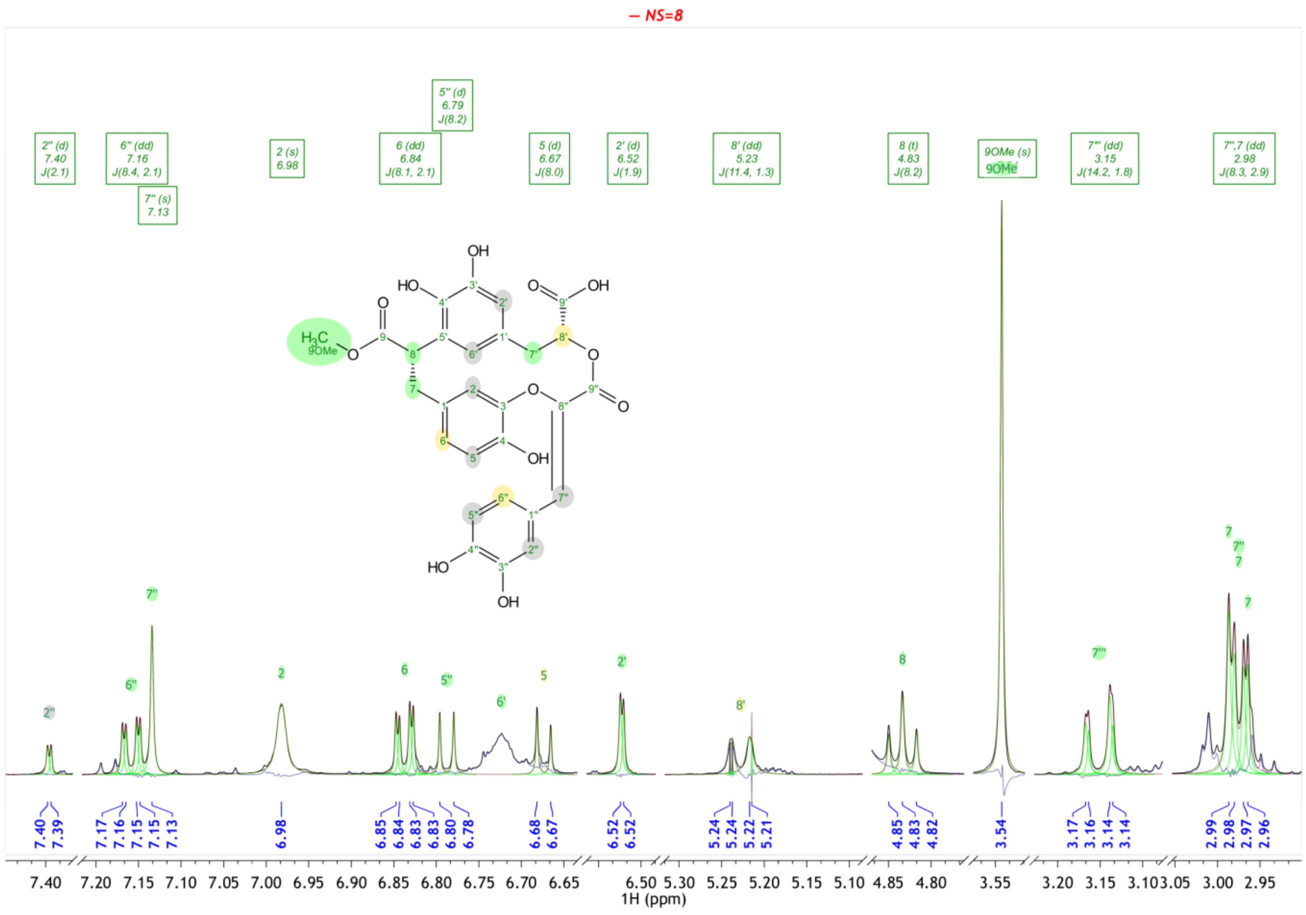

Figure 81S. ^{1}H NMR spectrum of compound 38

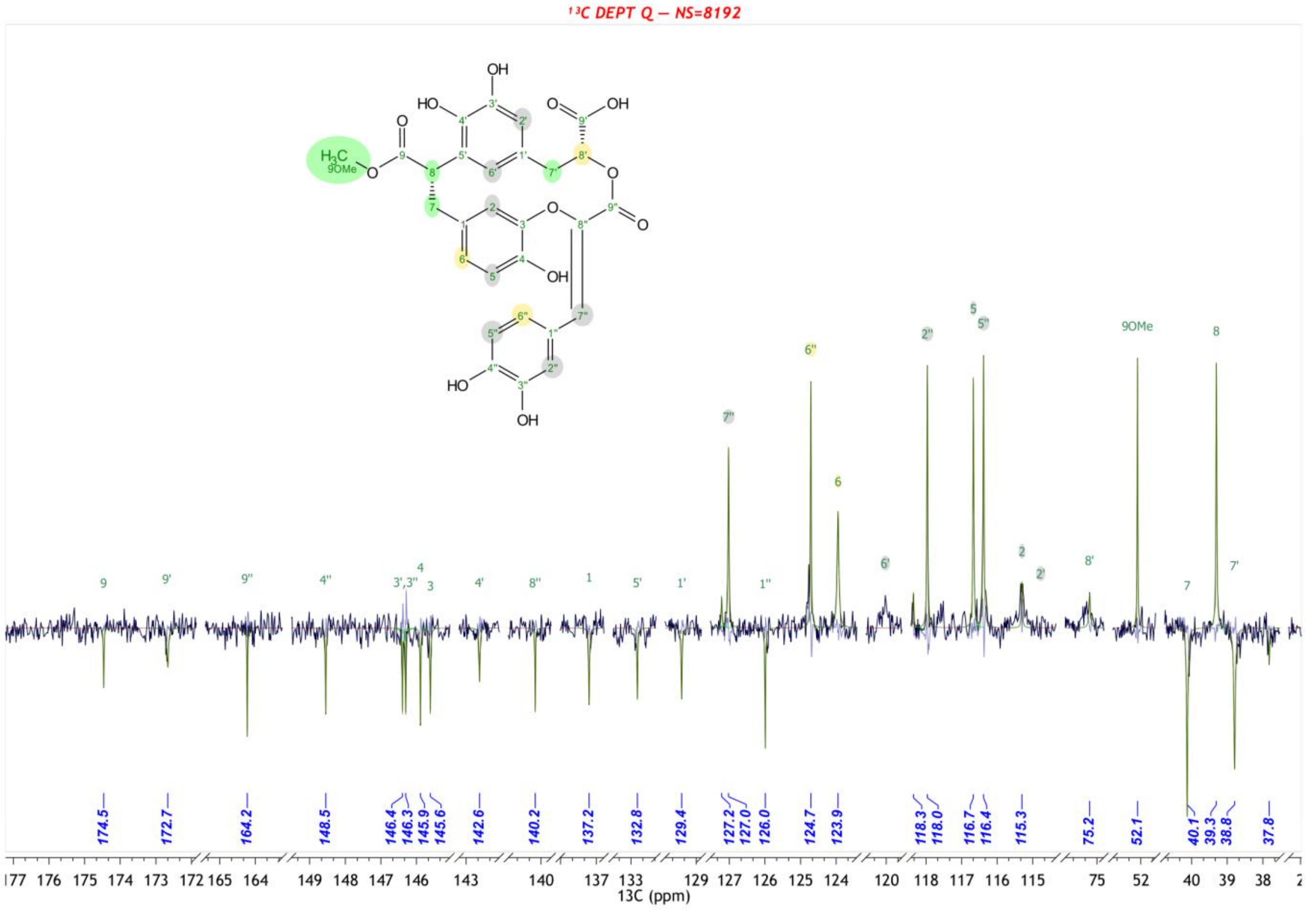

Figure 82S. ^{13}C DEPTQ NMR spectrum of compound 38

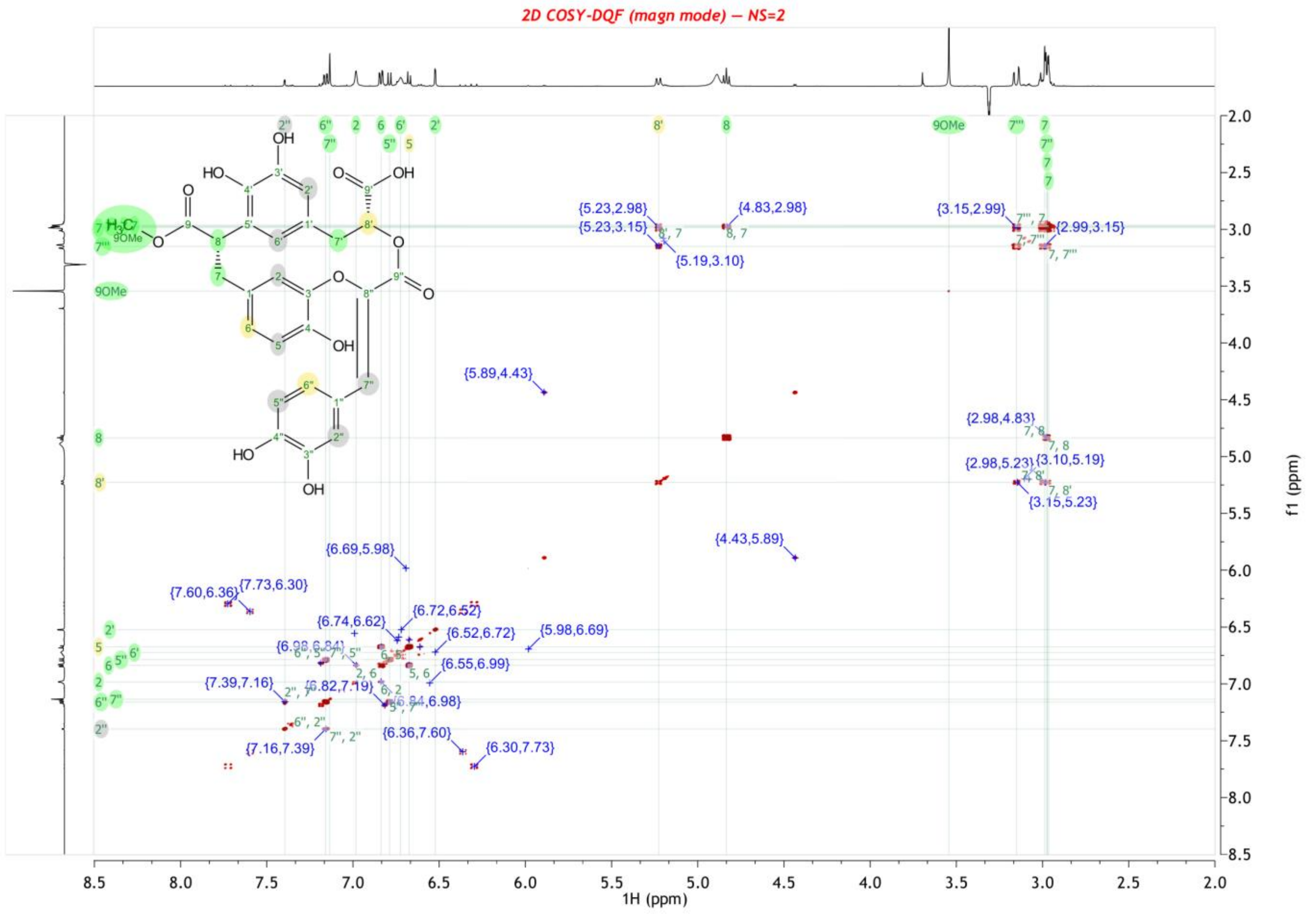

Figure 83S. ^{1}H-^{1}H COSY NMR spectrum of compound 38

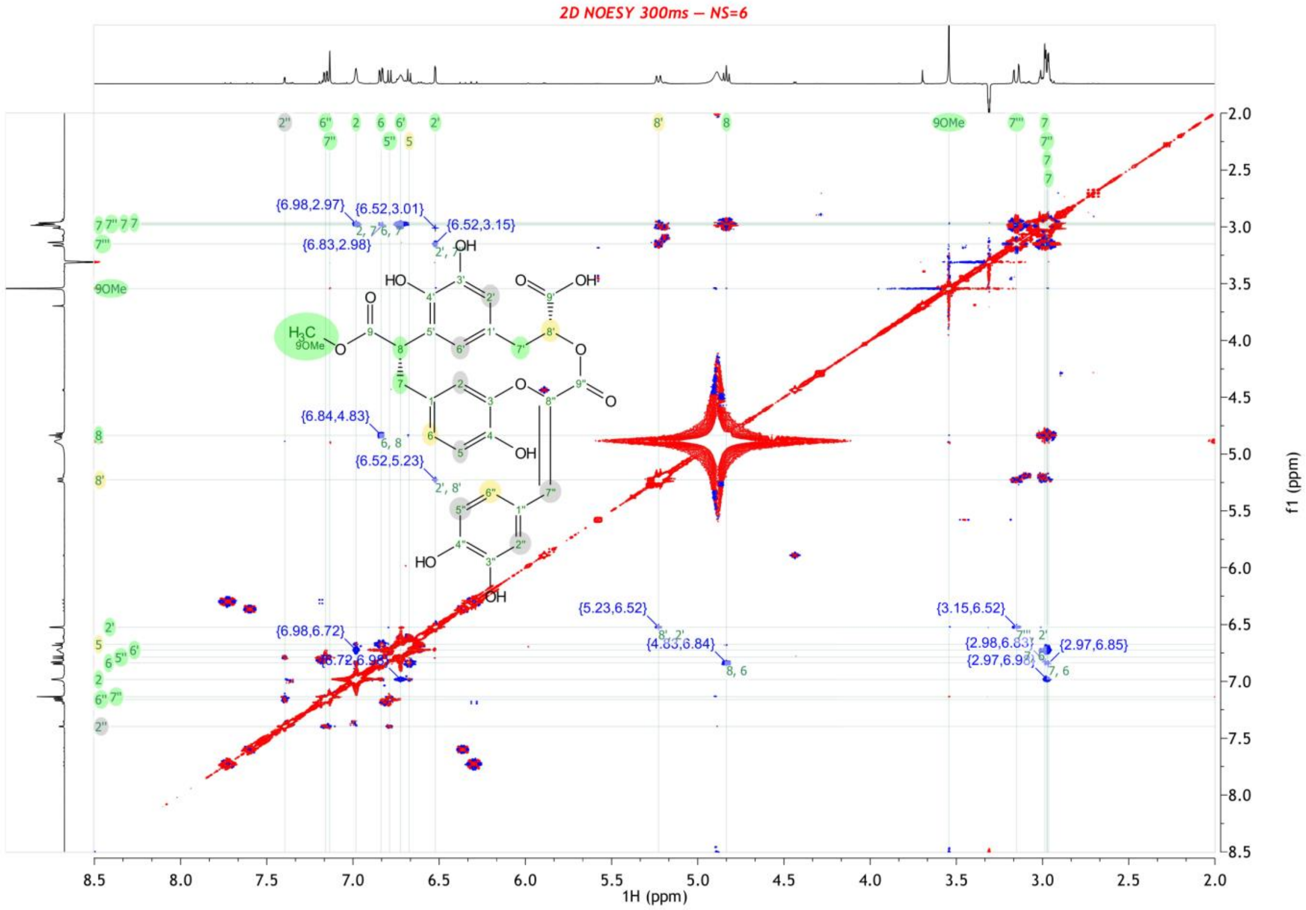

Figure 84S. ^{1}H-^{1}H NOESY (300 ms) NMR spectrum of compound 38

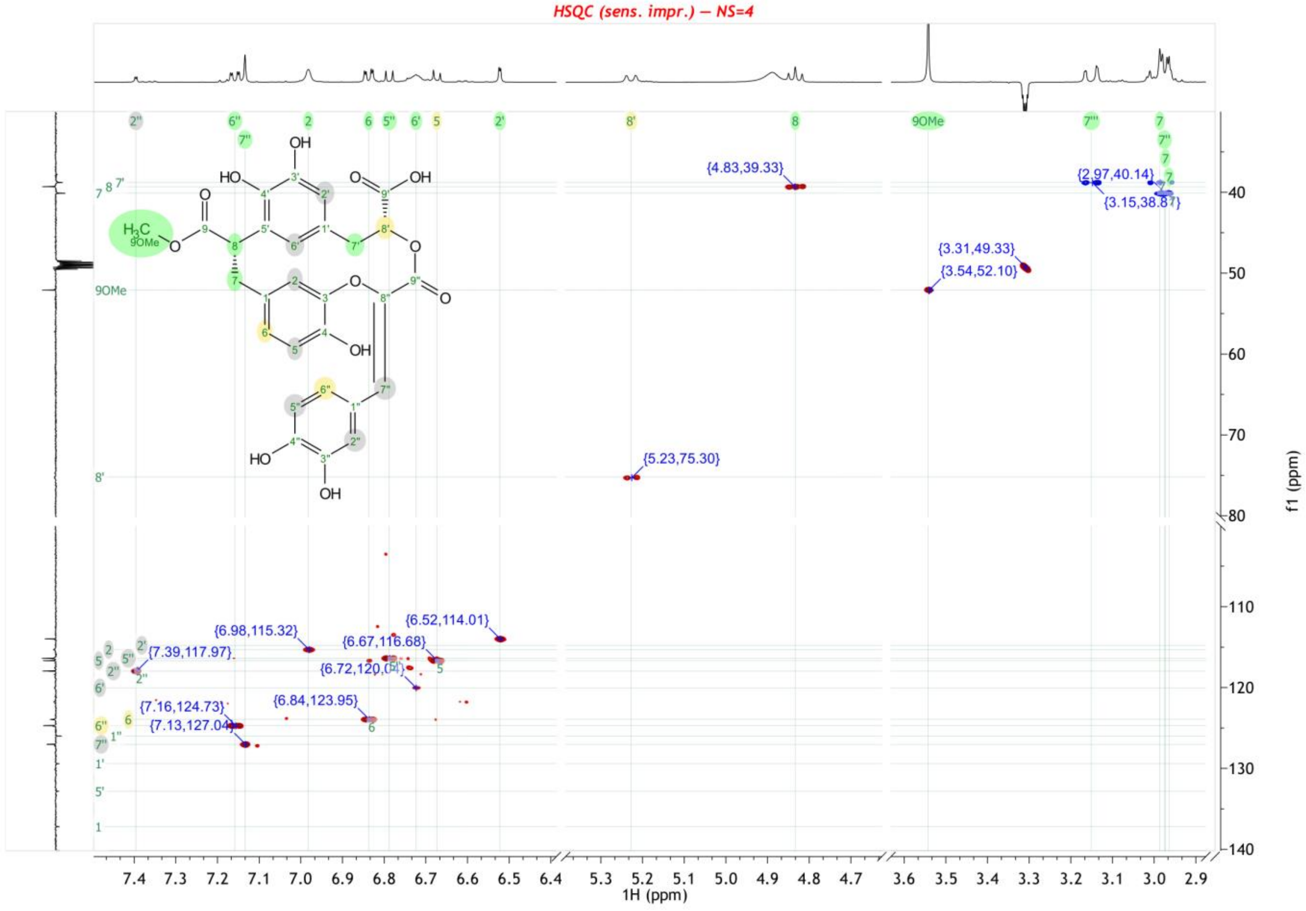

Figure 85S. ^{1}H-^{13}C HSQC NMR spectrum of compound 38

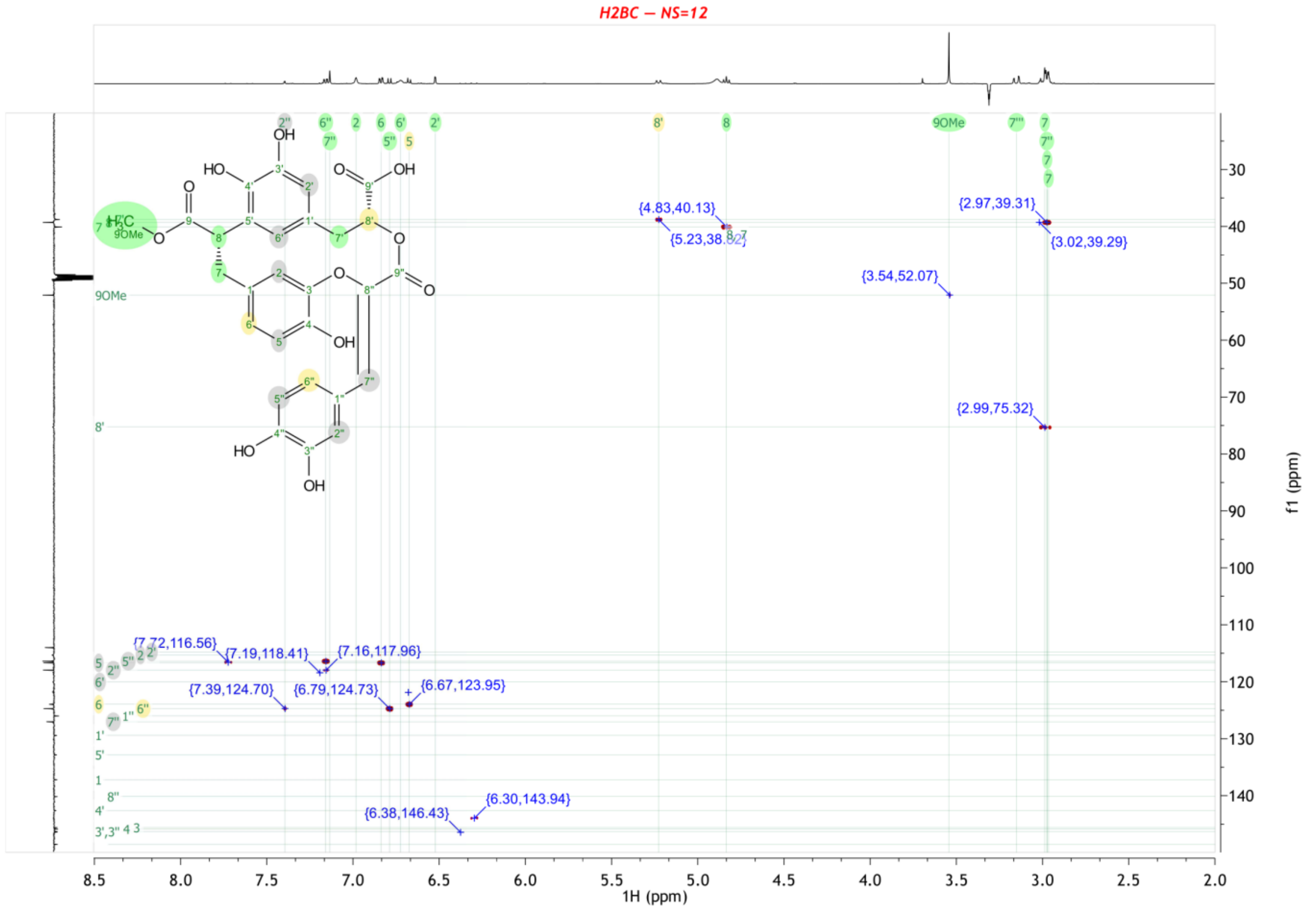

Figure 86S. ^{1}H-^{13}C H2BC NMR spectrum of compound 38

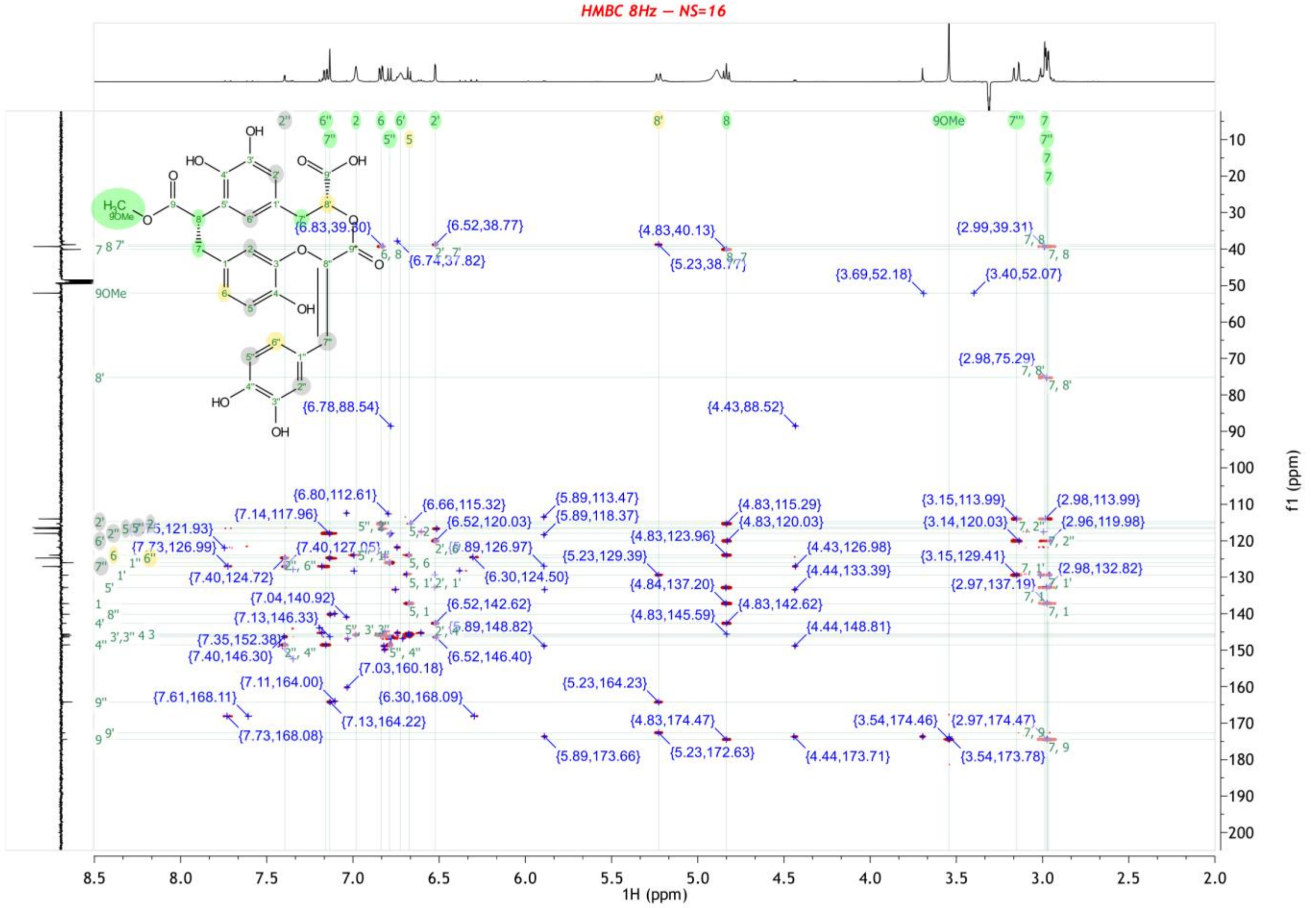

Figure 87S. ^{1}H-^{13}C HMBC (8 Hz) NMR spectrum of compound 38

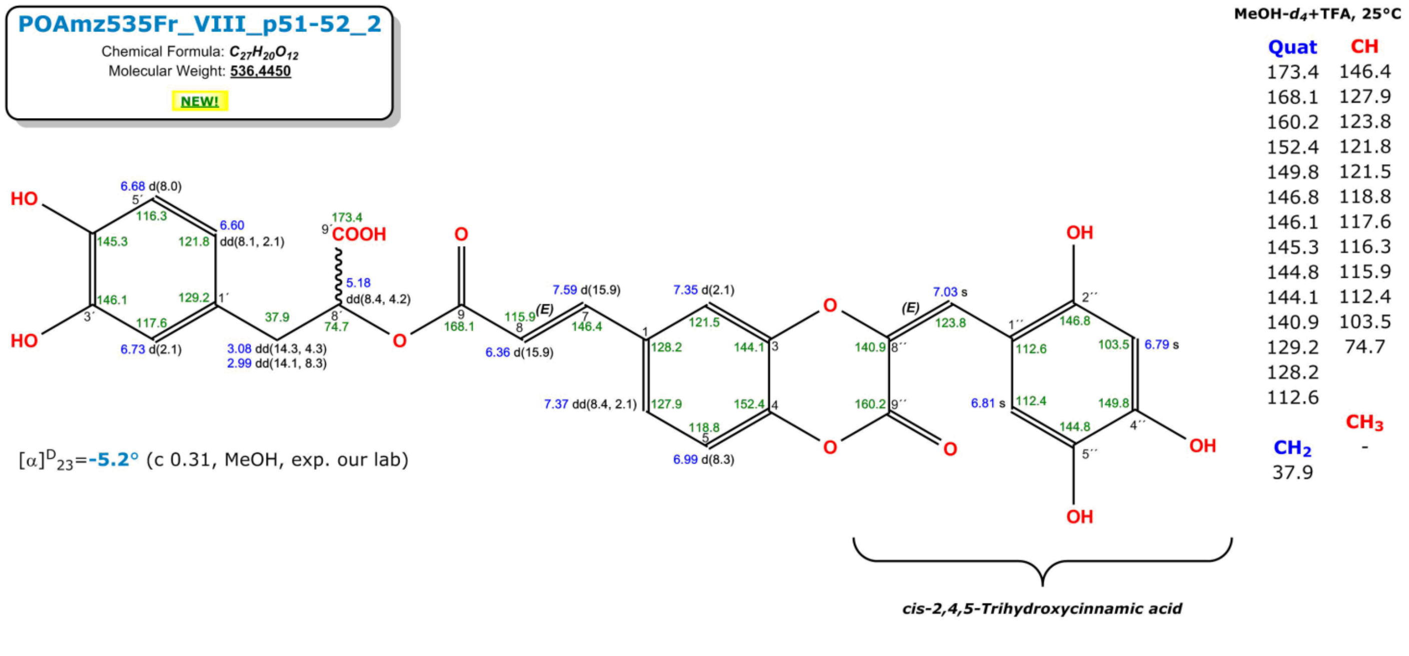

Pulmitric acid B

1. Murata, T., Watahiki, M., Tanaka, Y., Miyase, T., Yoshizaki, F., 2010. Hyaluronidase Inhibitors from Takuran, Lycopus lucidus. Chem. Pharm. Bull. (Tokyo). 58, 394–397. doi:10.1248/cpb.58.394

Figure 88S. ^{1}H (500 MHz) and ^{13}C (125 MHz) NMR data of compound 39 in CD_3OD, 25°C

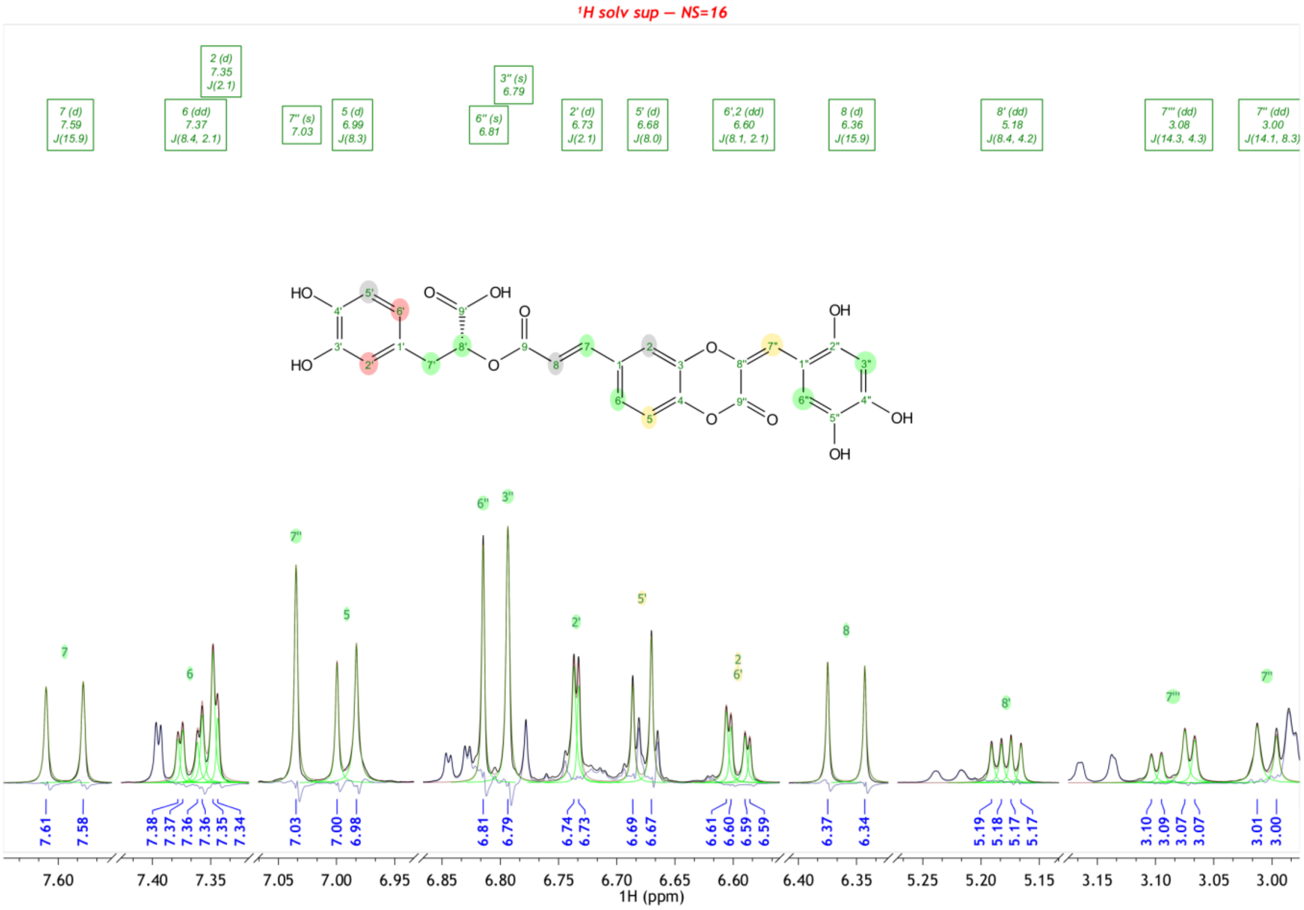

Figure 89S. ^{1}H NMR spectrum of compound 39

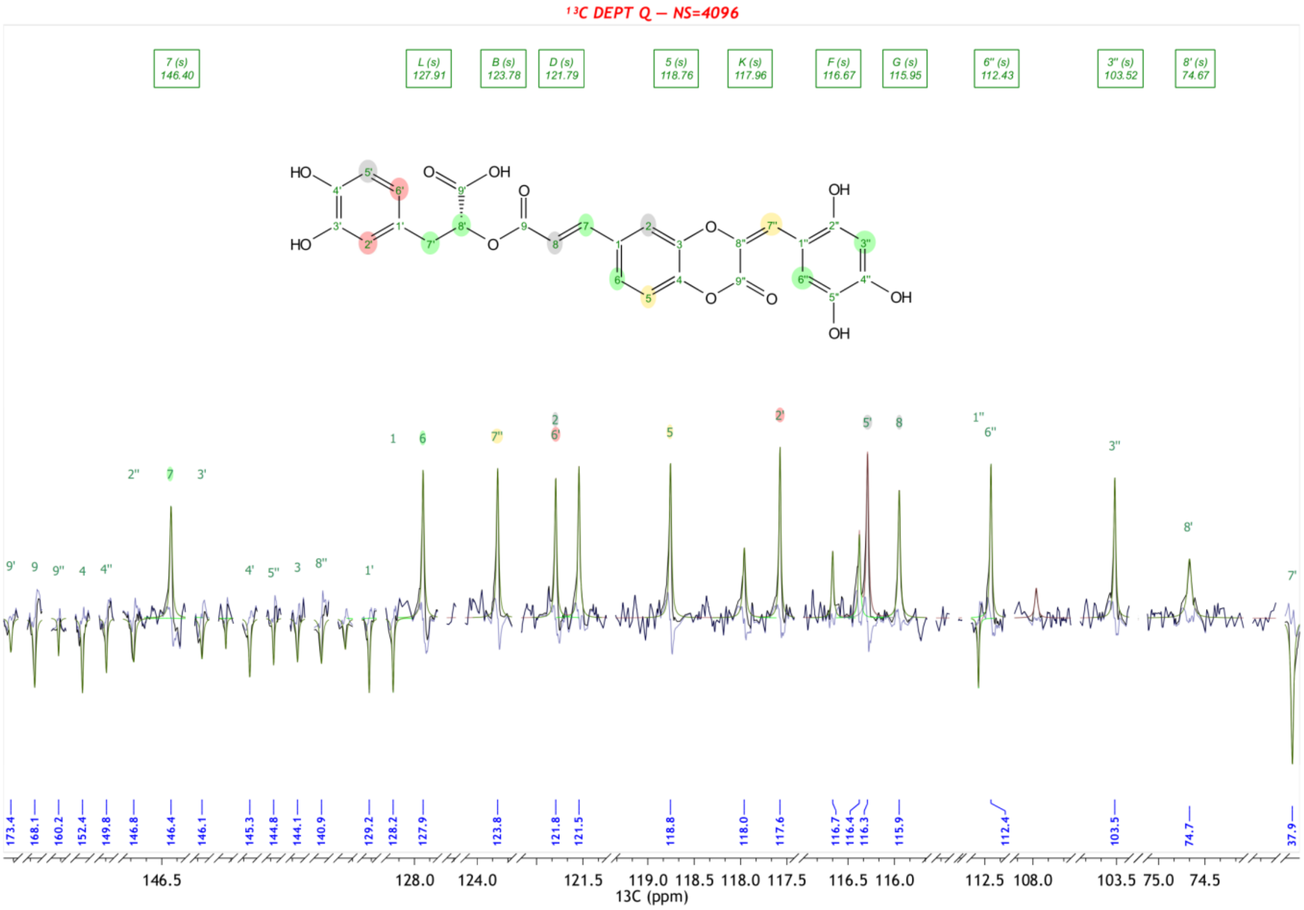

Figure 90S. ^{13}C DEPTQ NMR spectrum of compound 39

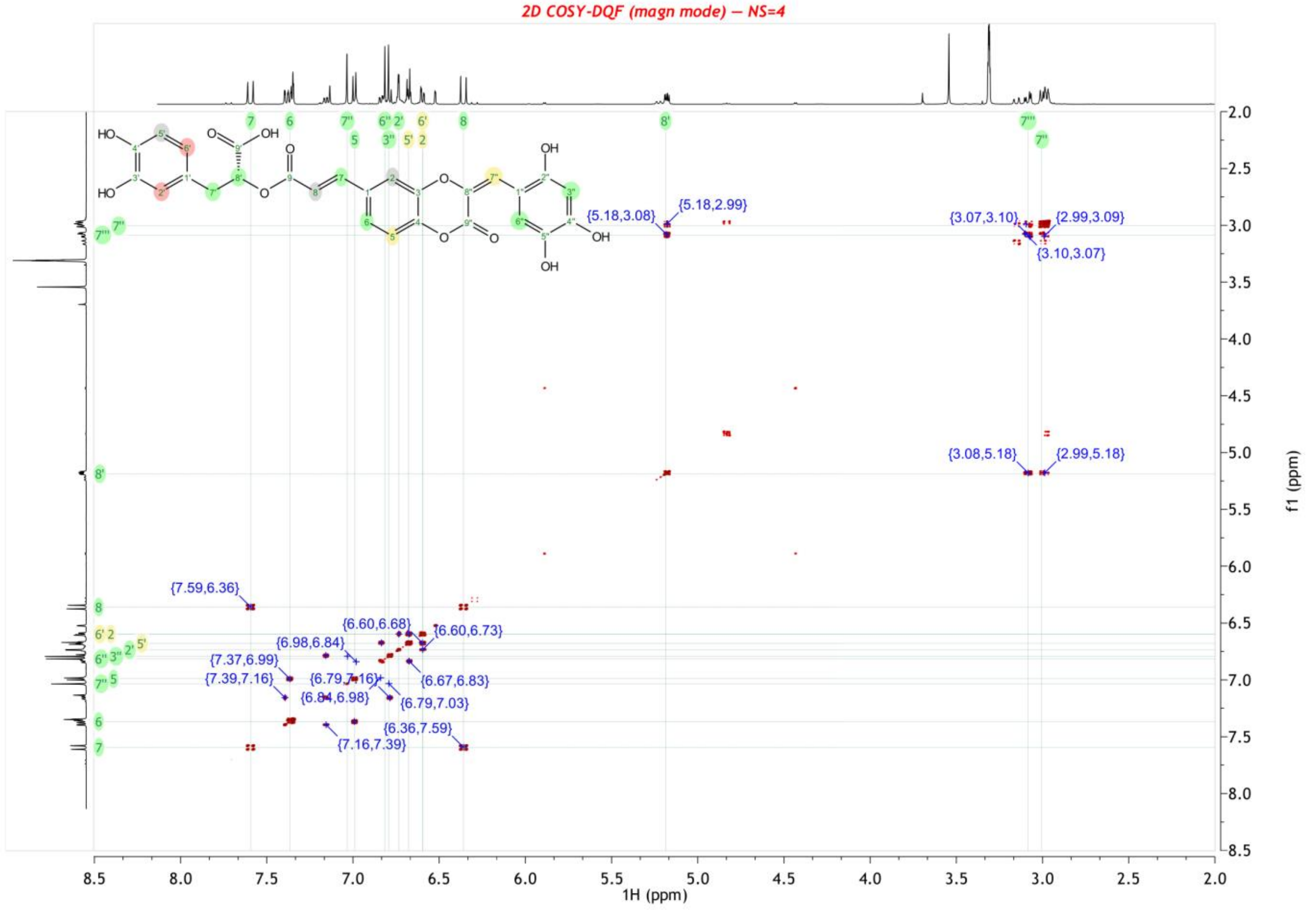

Figure 91S. ^{1}H-^{1}H COSY NMR spectrum of compound 39

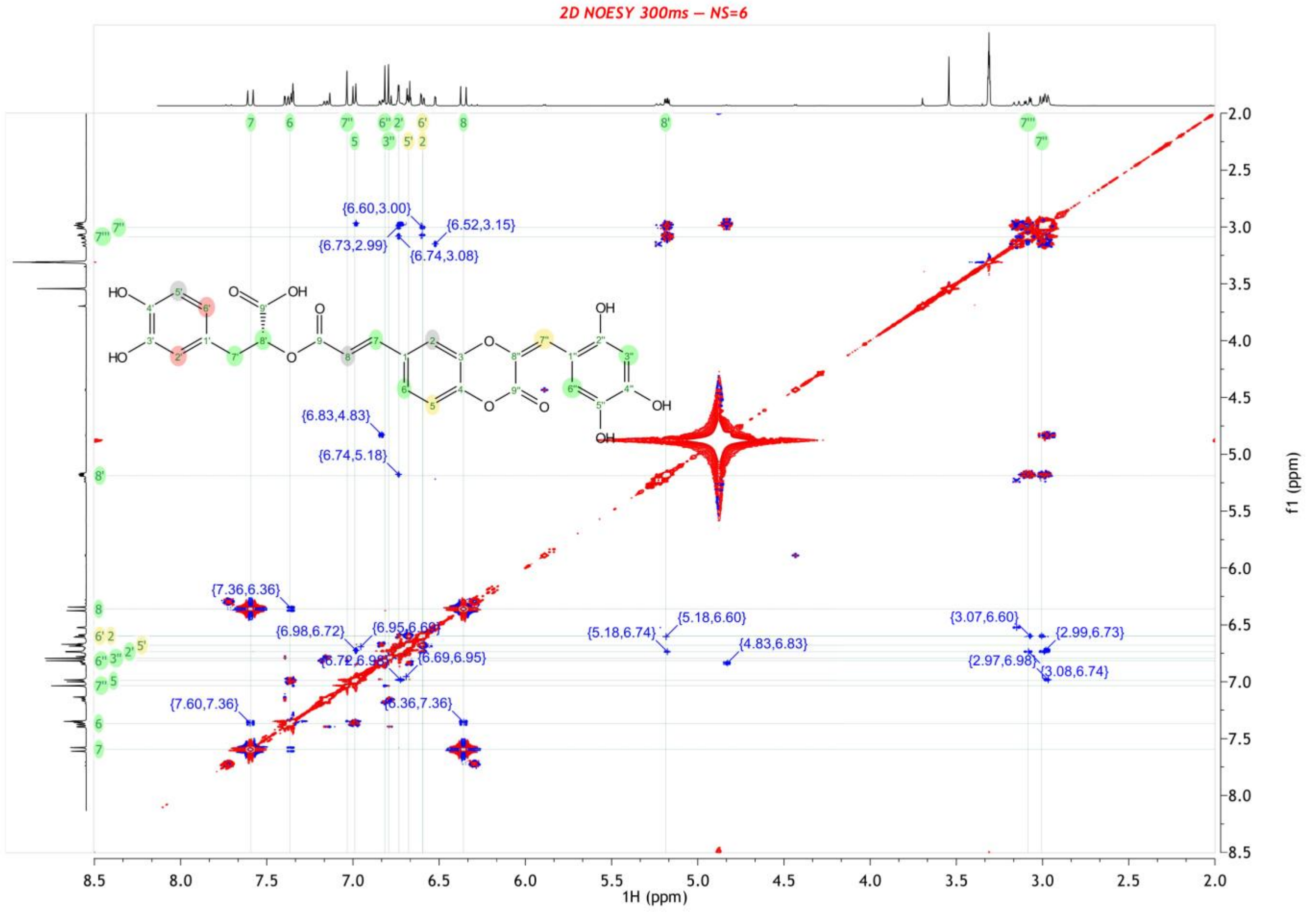

Figure 92S. ^{1}H-^{1}H NOESY (300 ms) NMR spectrum of compound 39

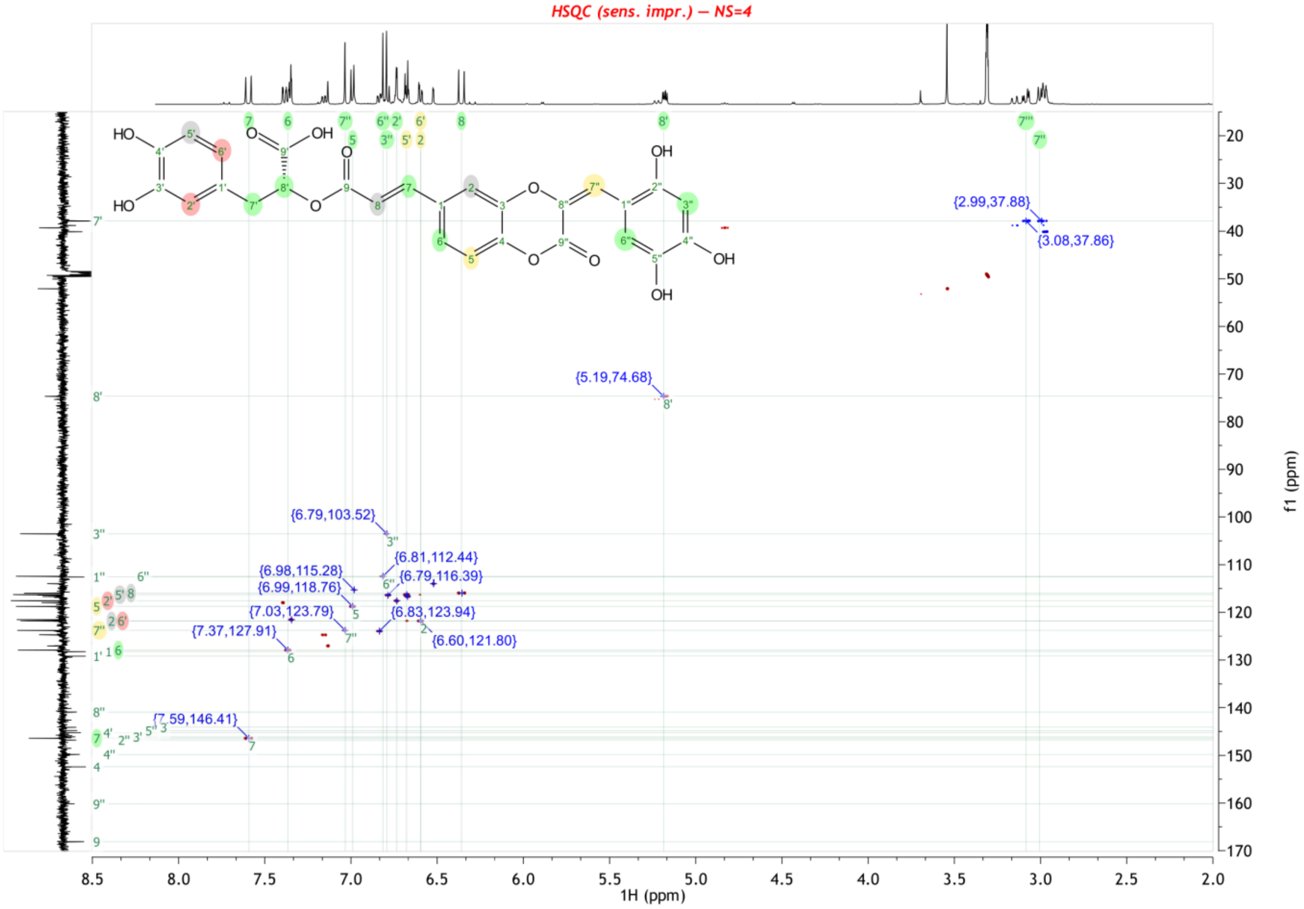

Figure 93S. ^{1}H-^{13}C HSQC NMR spectrum of compound 39

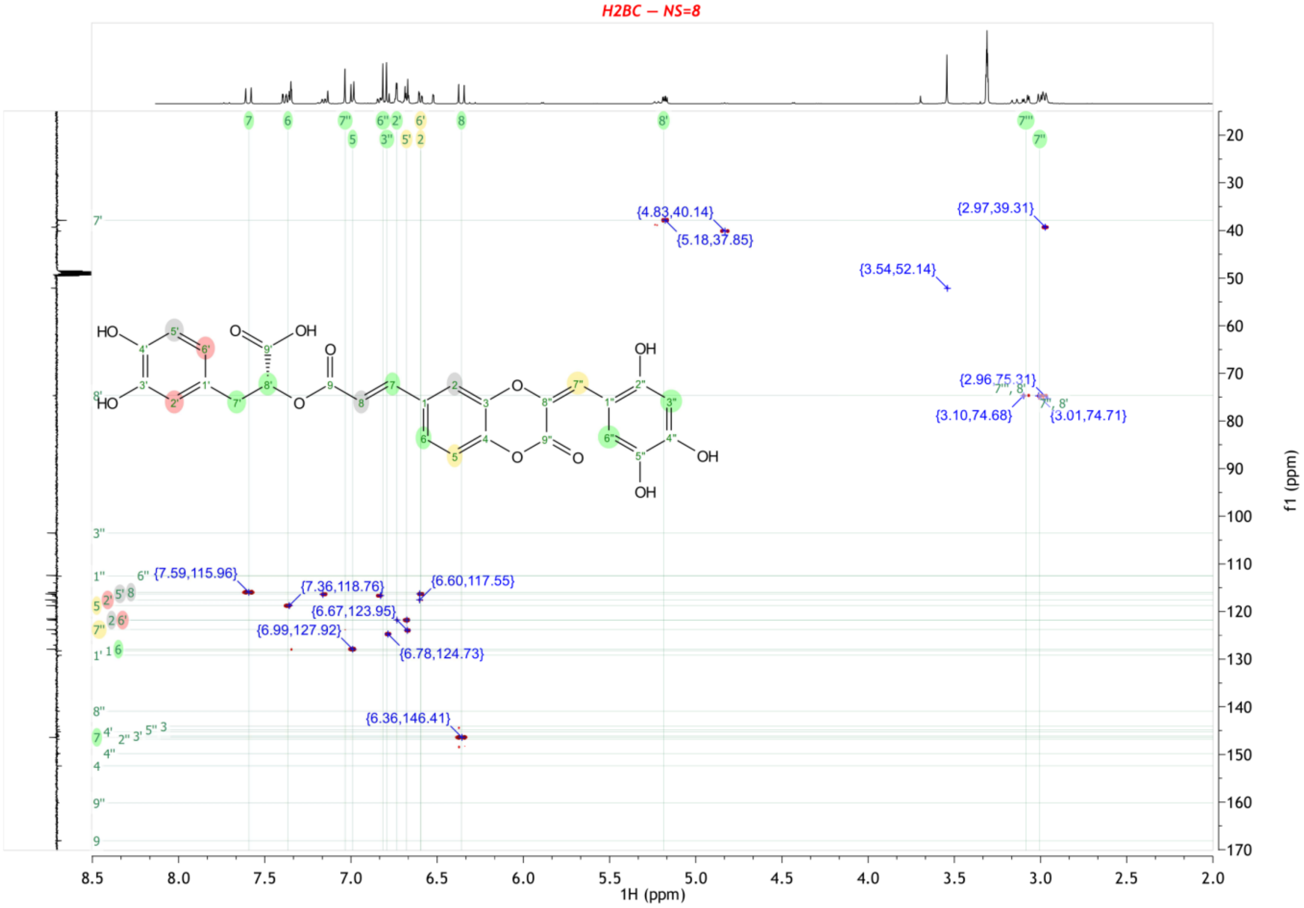

Figure 94S. ^{1}H-^{13}C H2BC NMR spectrum of compound 39

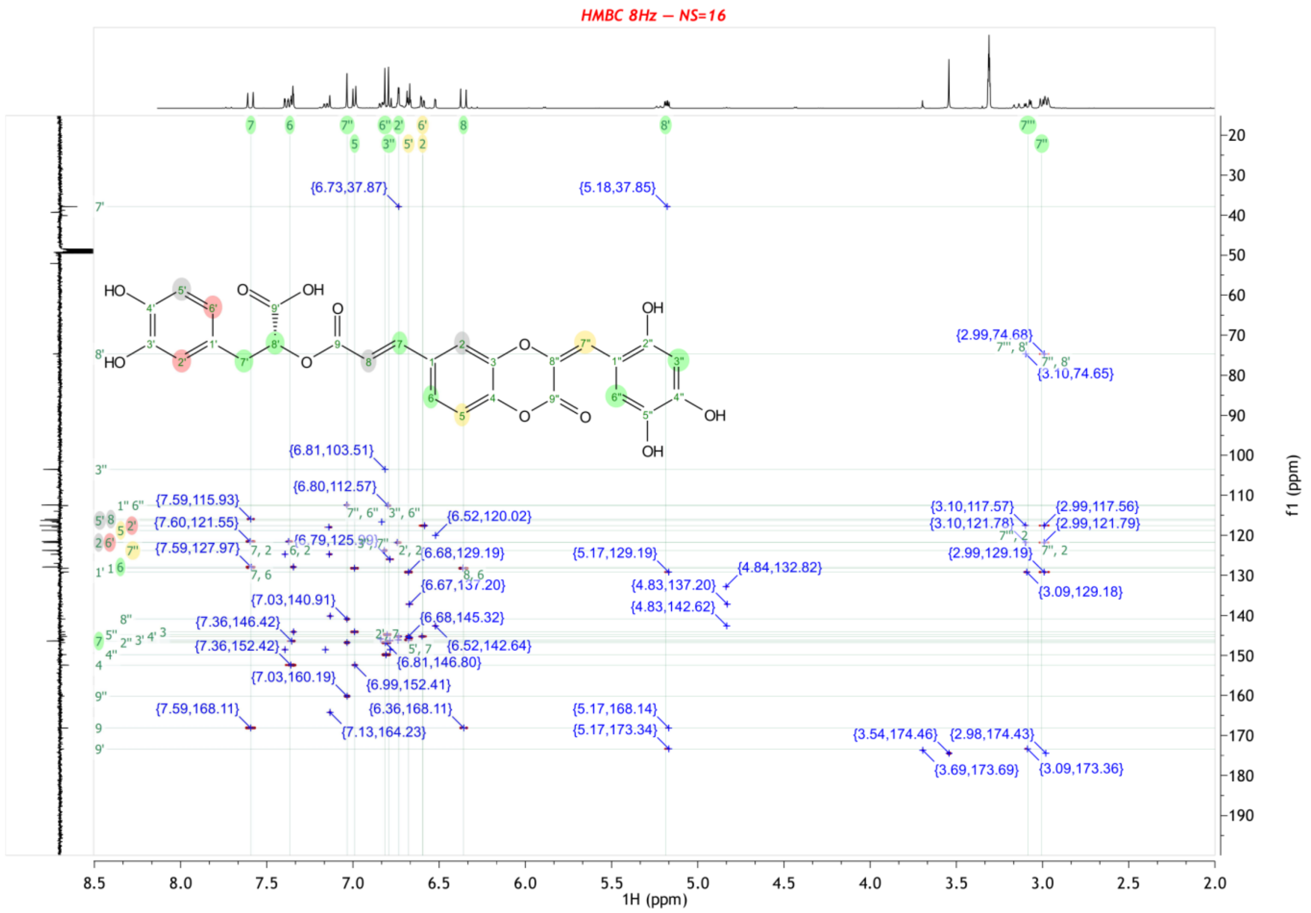

Figure 95S. ^{1}H-^{13}C HMBC (8 Hz) NMR spectrum of compound 39

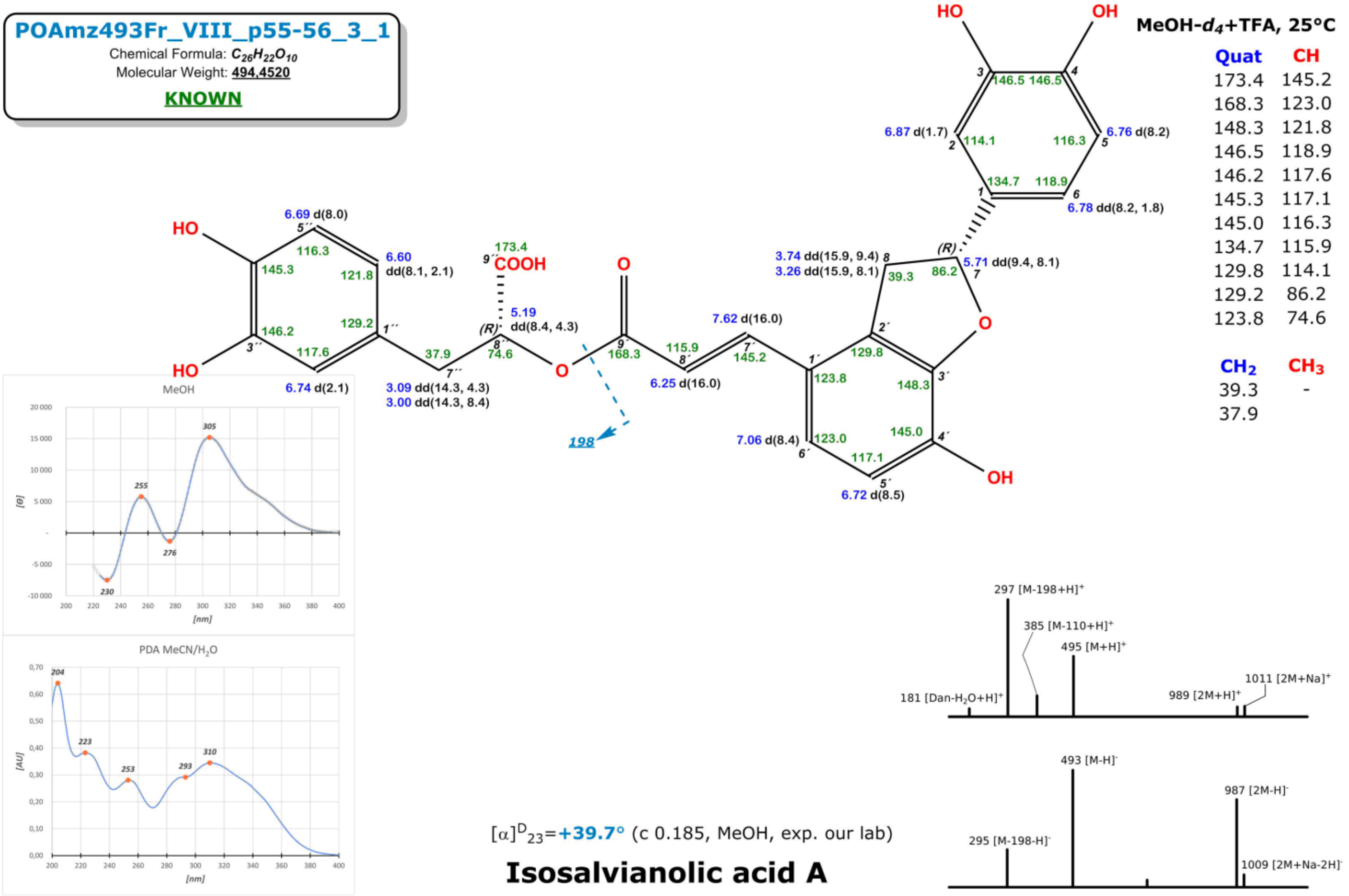

1. Lee, H. J.; Cho, J.-Y.; Moon, J.-H. Chemical conversions of salvianolic acid B by decoction in aqueous solution. Fitoterapia 2012, 83, 1196–1204, doi:10.1016/j.fitote.2012.06.015.

Figure 96S. ^{1}H (500 MHz) and ^{13}C (125 MHz) NMR data of compound 40 in CD_3OD, 25°C; on-line PDA UV spectrum in MeCN/H_2O; ECD spectrum in MeOH

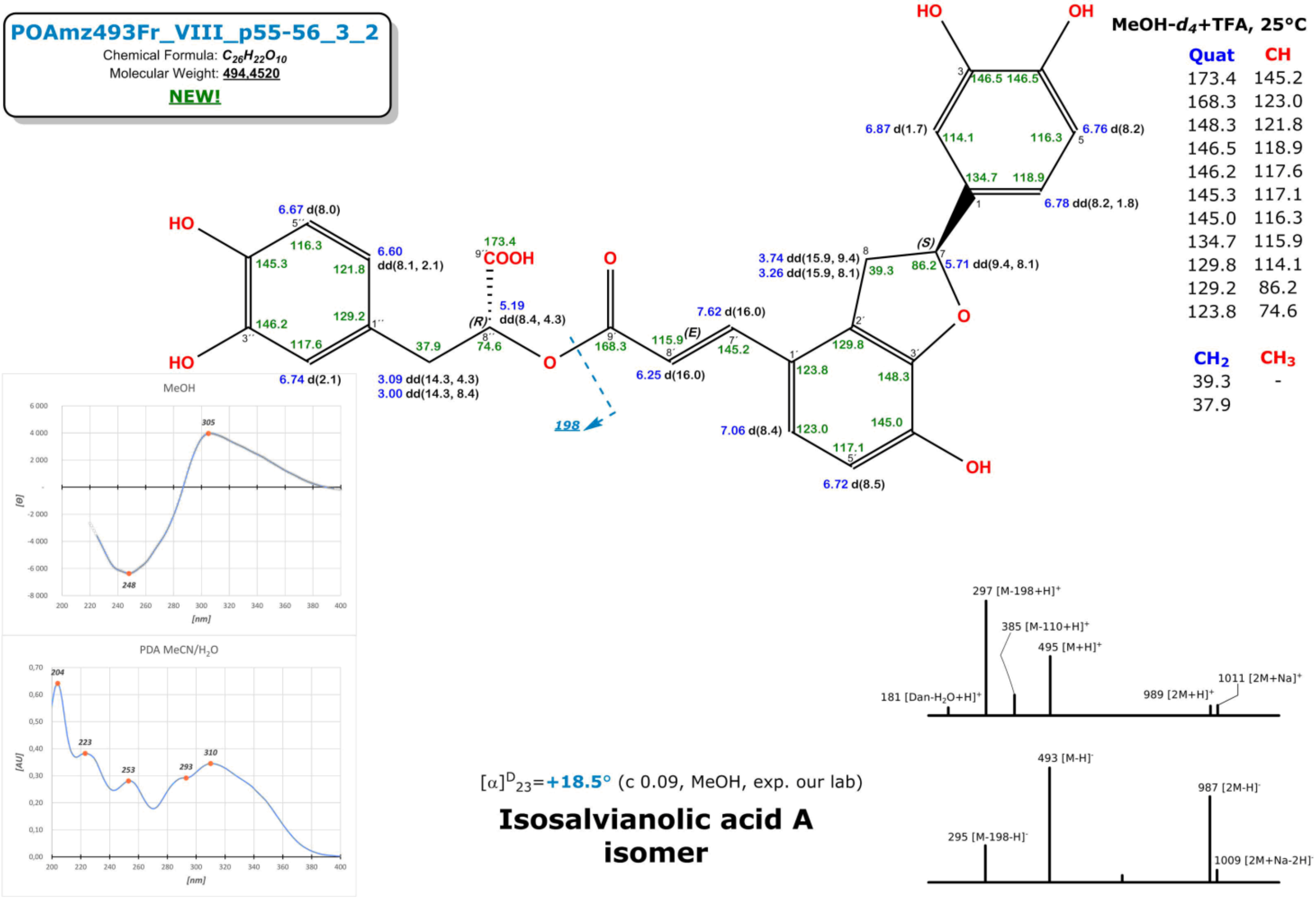

Figure 97S. ^{1}H (500 MHz) and ^{13}C (125 MHz) NMR data of compound 41 in CD_3OD, 25°C; on-line PDA UV spectrum in MeCN/H_2O; ECD spectrum in MeOH

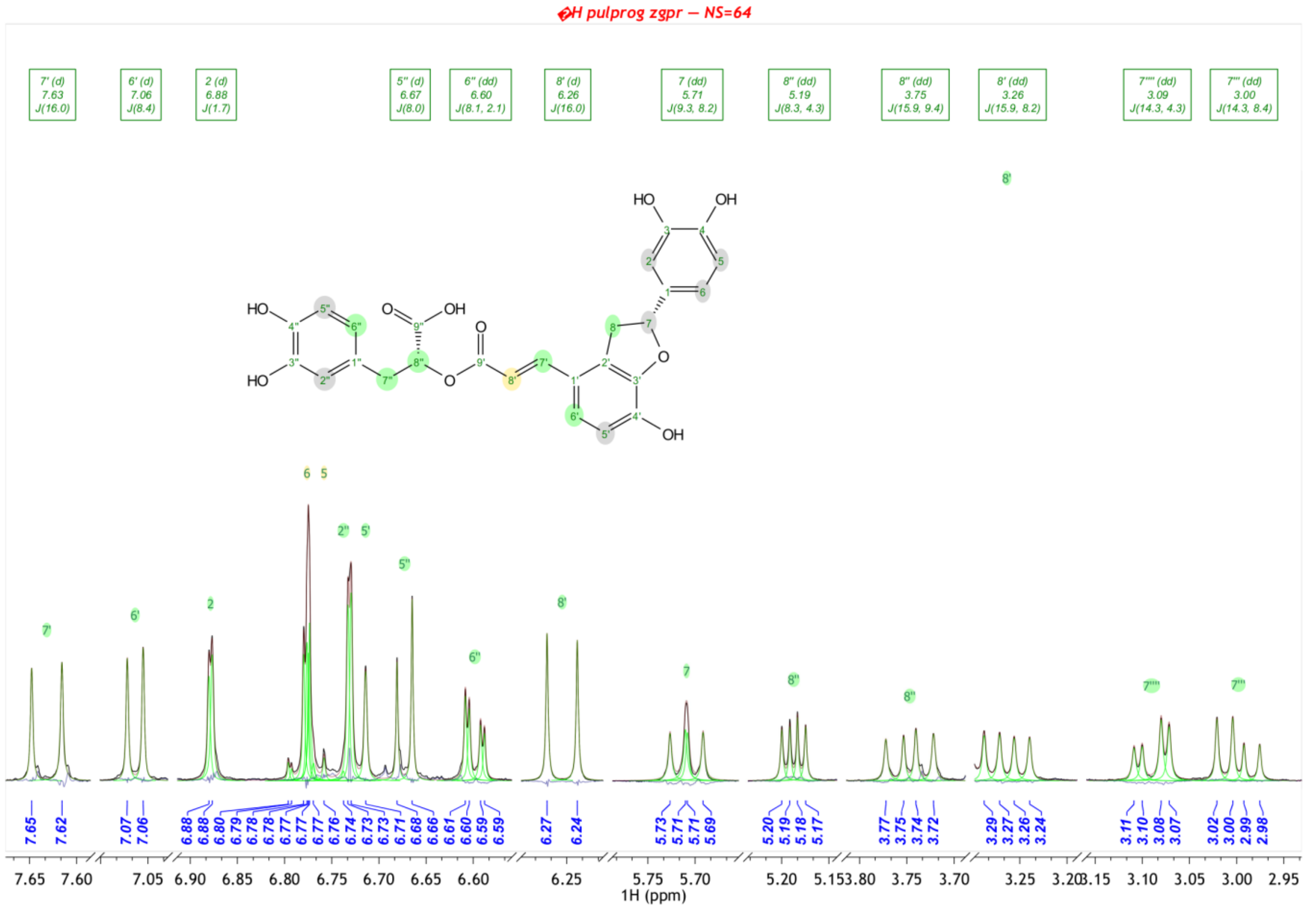

Figure 98S. ^{1}H NMR spectrum of compound 41

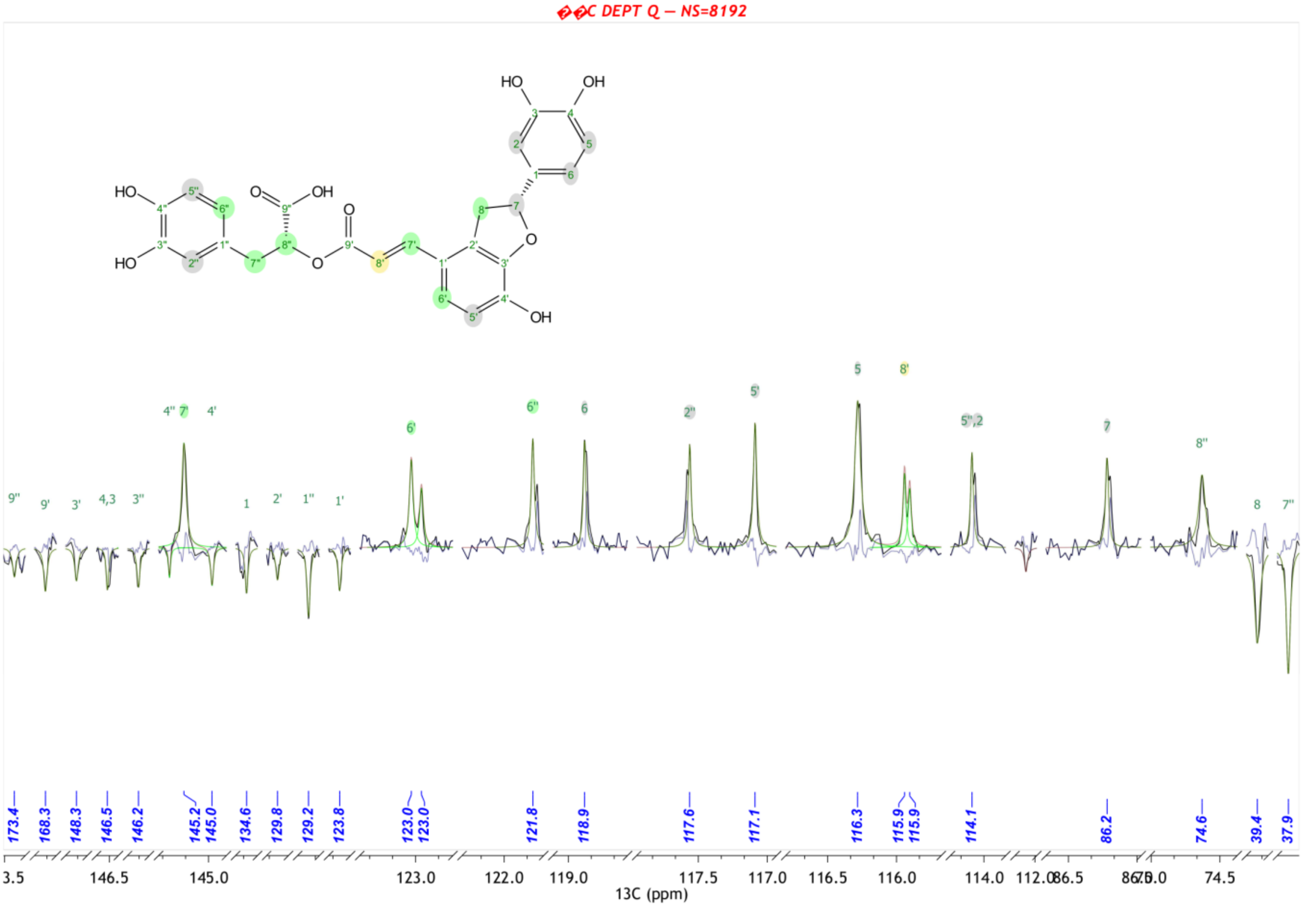

Figure 99S. ^{13}C DEPTQ NMR spectrum of compound 41

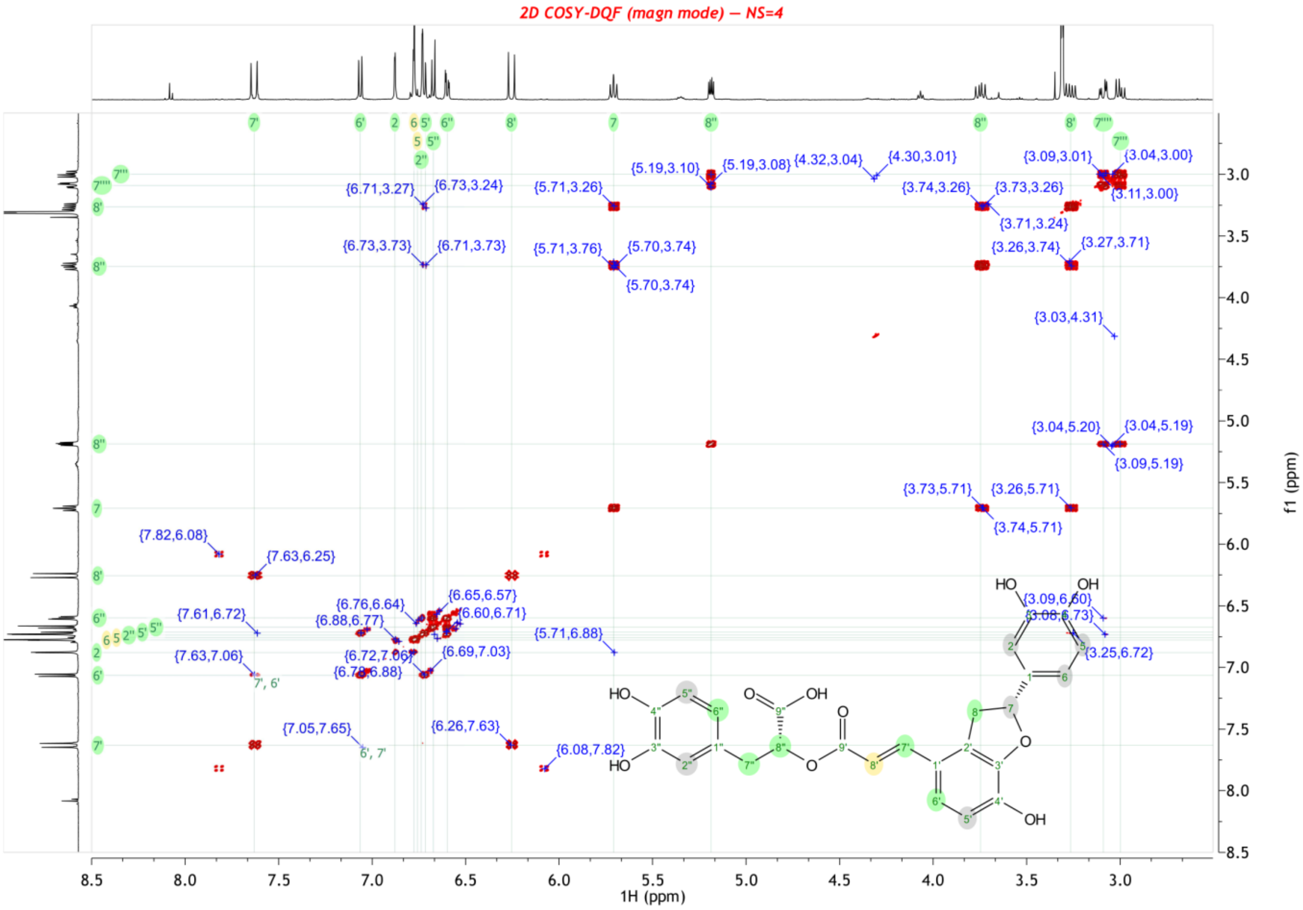

Figure 100S. ^{1}H-^{1}H COSY NMR spectrum of compound 41

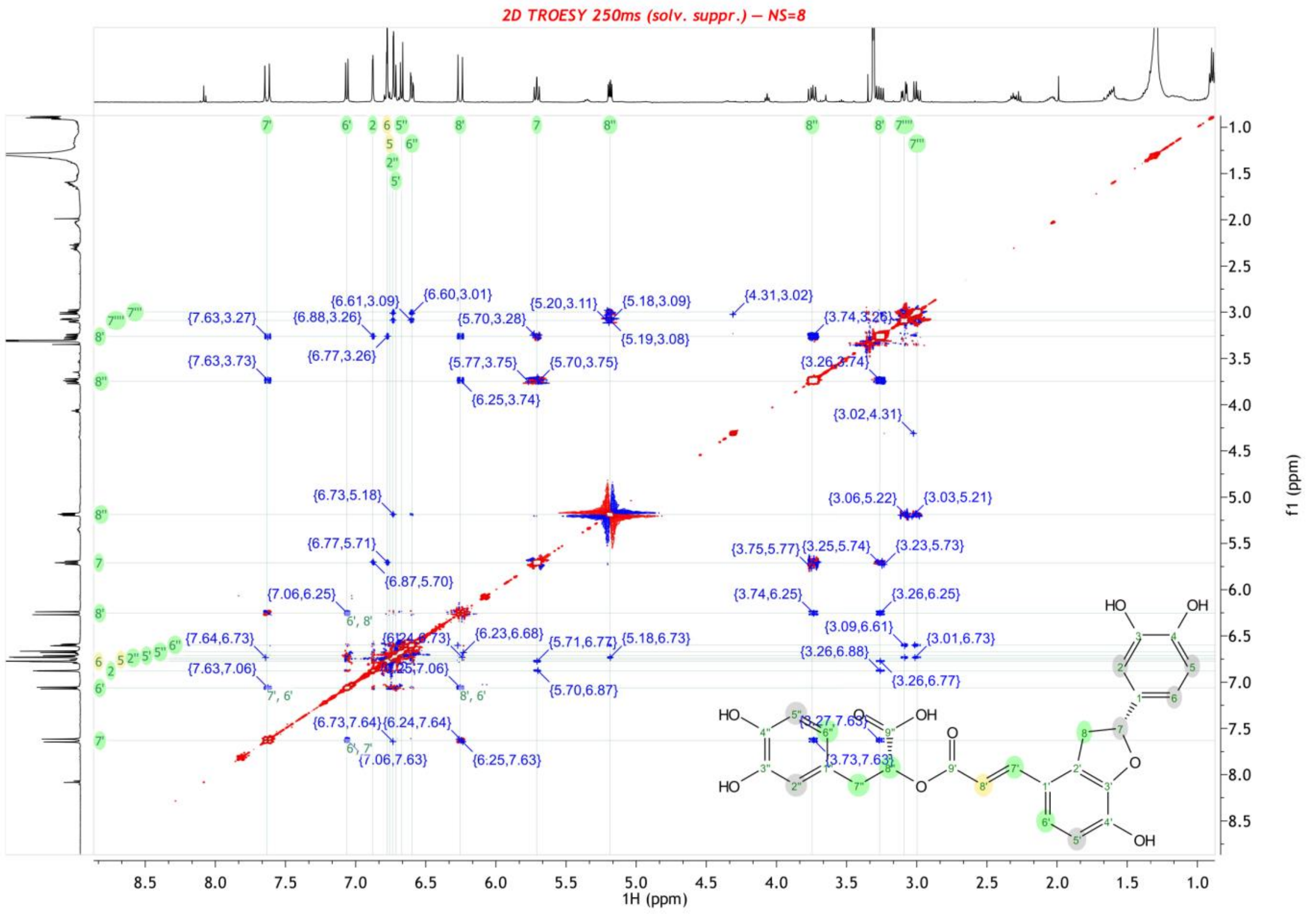

Figure 101S. ^1H-^1H TROESY (250 ms) NMR spectrum of compound 41

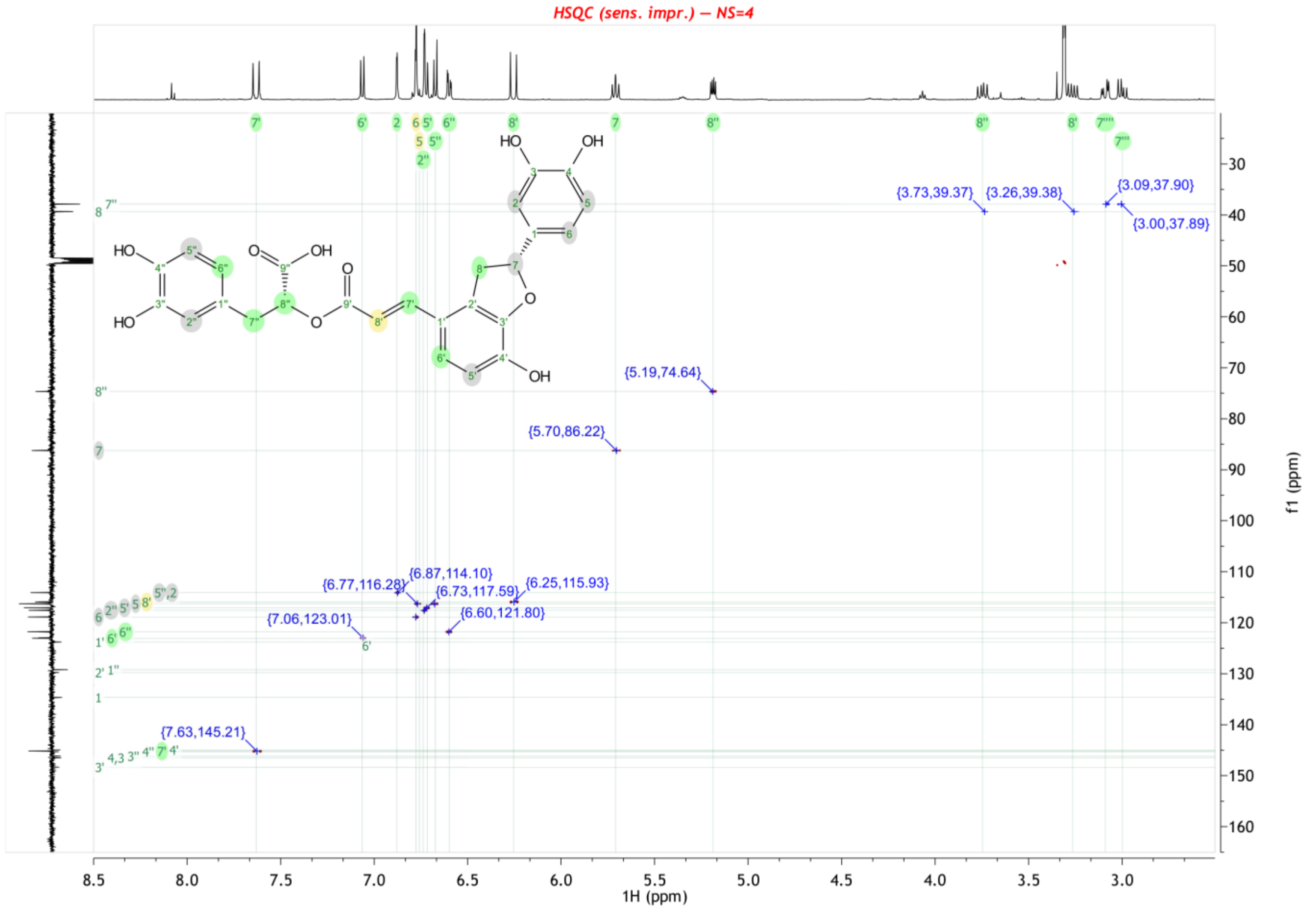

Figure 102S. ^1H-^{13}C HSQC NMR spectrum of compound 41

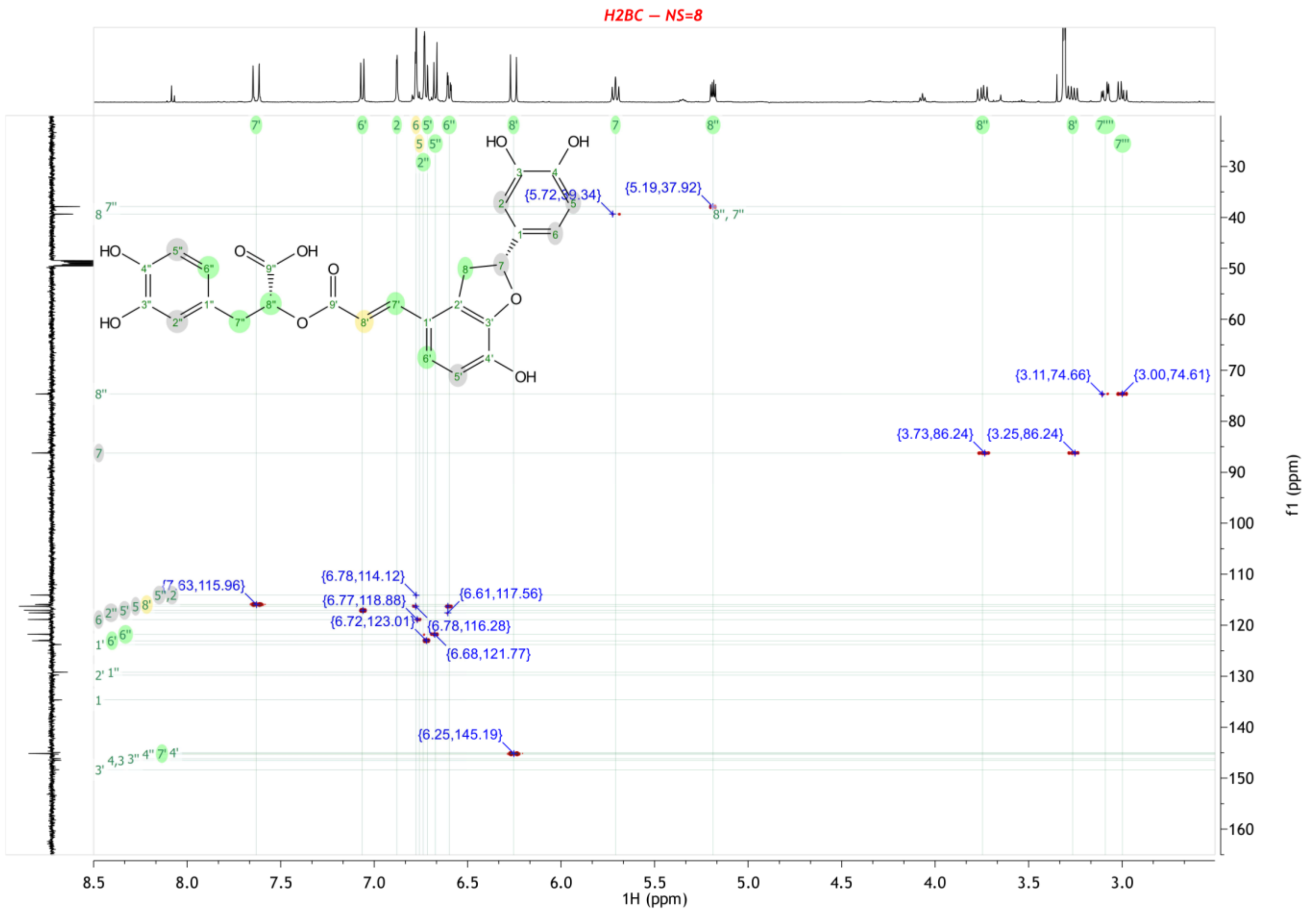

Figure 103S. ^{1}H-^{13}C H2BC NMR spectrum of compound 41

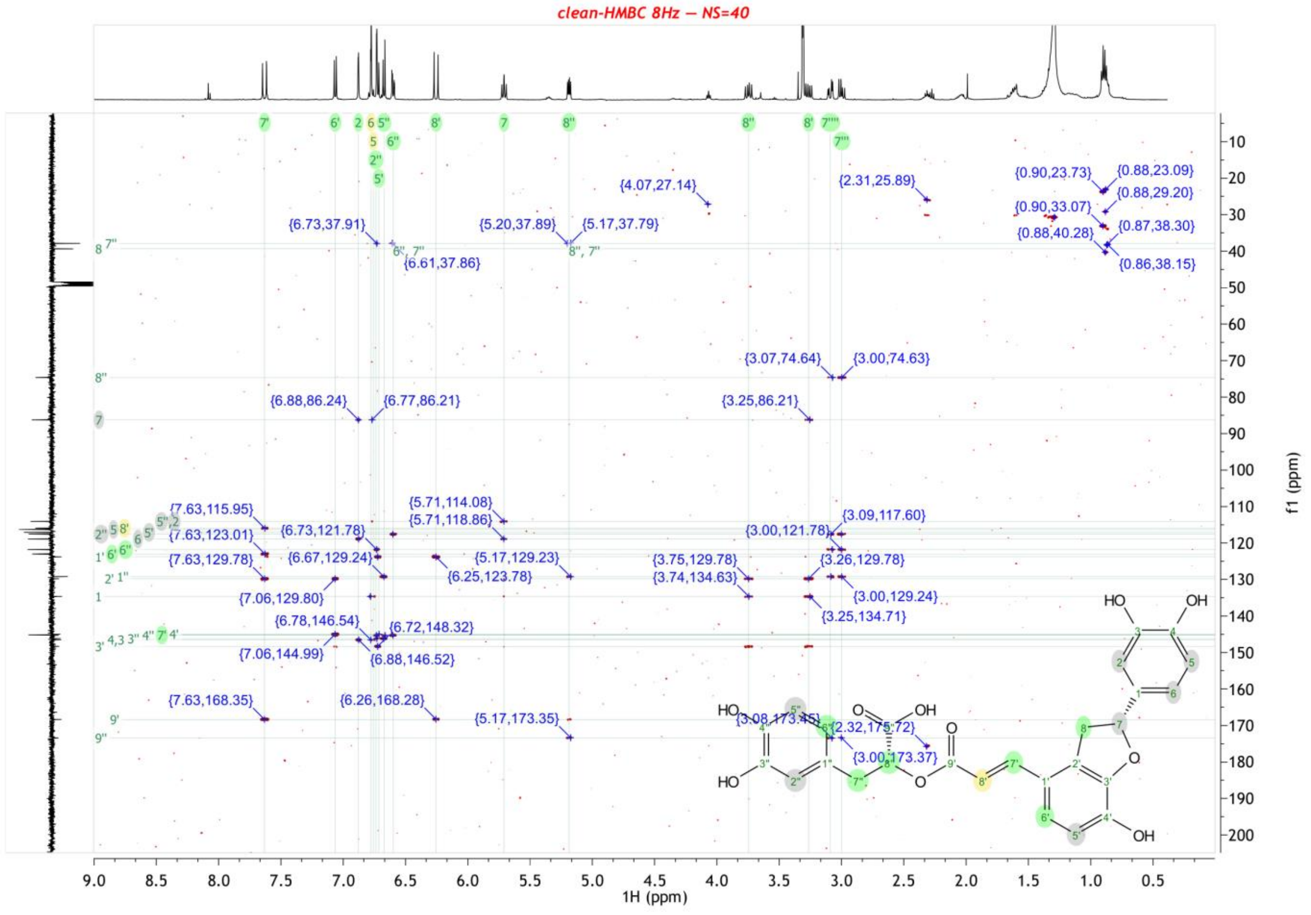

Figure 104S. ^{1}H-^{13}C HMBC (8 Hz) NMR spectrum of compound 41

POAmz373Fr_VIII_p45-48_4-5
Chemical Formula: $C_{19}H_{18}O_8$
Molecular Weight: 374.3450
KNOWN

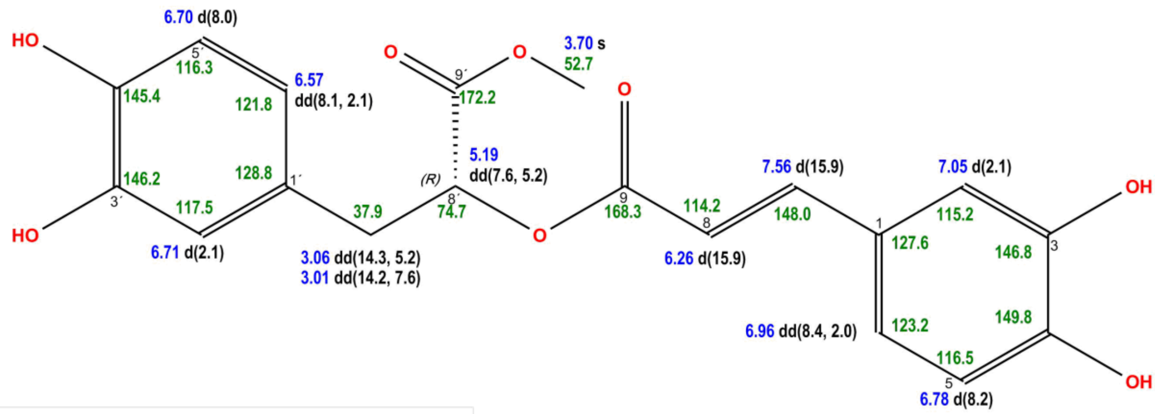

CH
148.0
123.2
121.8
117.5
116.5
116.3
115.2
114.2
74.7
**CH3**
52.7

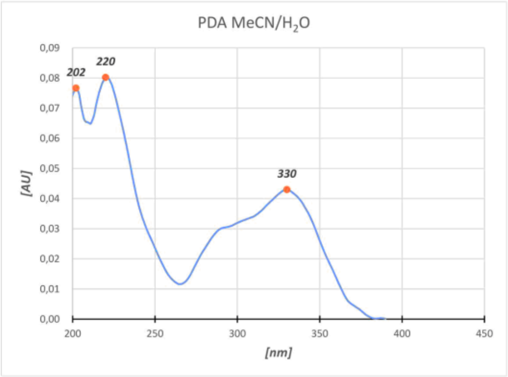

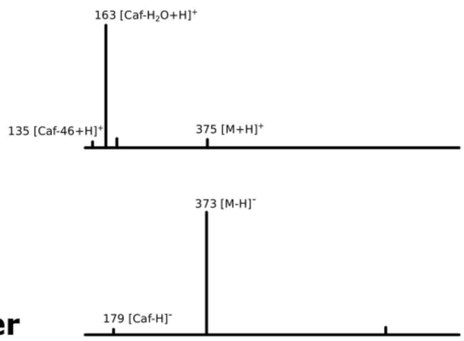

$[\alpha]^{D}_{23}$=**+26.6°** (c 0.26, MeOH, exp. our lab)

Rosmarinic acid methyl ester

Figure 105S. ^{1}H (500 MHz) and ^{13}C (125 MHz) NMR data of compound 43 in CD_3OD, 25°C; on-line PDA UV spectrum in MeCN/H_2O

POBmz551Fr_10
Chemical Formula: $C_{28}H_{24}O_{12}$
Molecular Weight: 552.4880
KNOWN

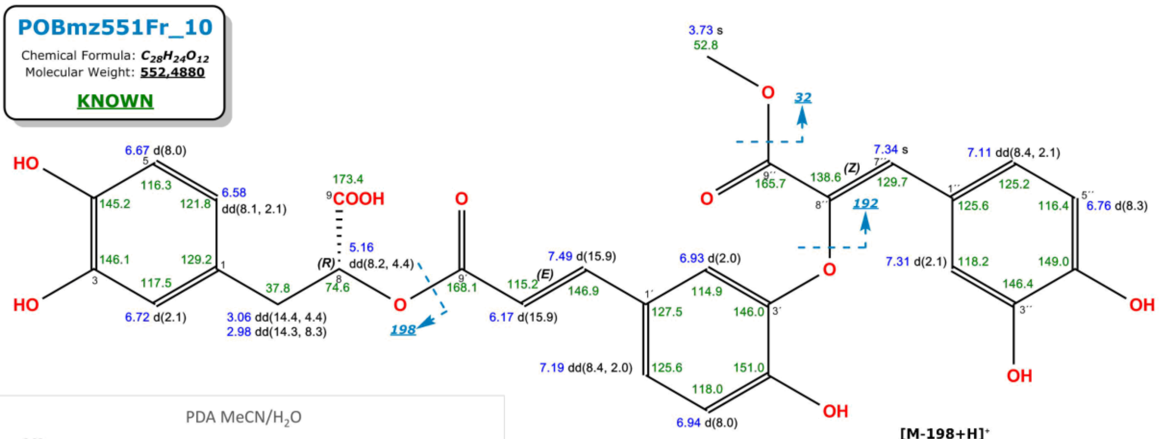

MeOH-d_4, 25°C

Quat	CH
173.4	146.9
168.1	129.7
165.7	125.6
151.0	125.2
149.0	121.8
146.4	118.2
146.1	118.0
146.0	117.5
145.2	116.4
138.6	116.3
129.2	115.2
127.5	114.9
125.6	74.6

CH2	CH3
37.8	52.8

5´

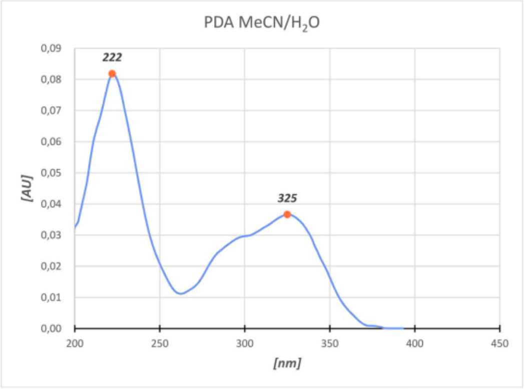

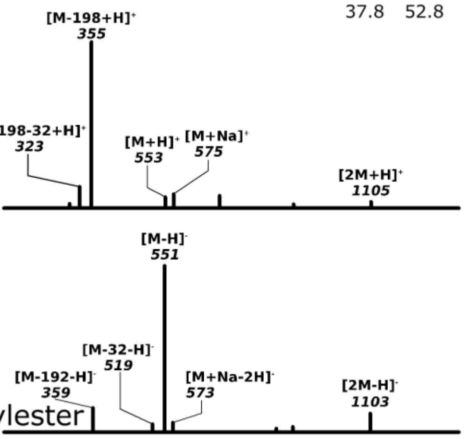

$[\alpha]^{D}_{21}$=**+65.5°** (c 0.55, MeOH)[1]
ECD (c 0.033 MeOH) / nm ([Δε]):
206 (-3.94), 251 (6.58), 280 (2.70), 297 (6.34), 328 (6.67)[1]

Salvianolic acid H-9''-methylester
3'-*O*-(8''-*Z*-caffeoyl)rosmarinic acid-9''-methylester

1.Murata, T., Watahiki, M., Tanaka, Y., Miyase, T., Yoshizaki, F., 2010. Hyaluronidase Inhibitors from Takuran, Lycopus lucidus. Chem. Pharm. Bull. (Tokyo). 58, 394–397. doi:10.1248/cpb.58.394

Figure 106S. ^{1}H (500 MHz) and ^{13}C (125 MHz) NMR data of compound 44 in CD_3OD, 25°C; on-line PDA UV spectrum in MeCN/H_2O

Table S1.

No.	Compound name	Regression equation	R^2	Calibration range [µg/mL]	LOD [µg/mL]	LOQ [µg/mL]
**1**	Danshensu	$y=-0.0002x^2+0.0194x+0.0045$	0.9977	0.2-35	0.1	0.4
**2**	Menisdaurin	$y=-0.0004x^2+0.0374x+0.0067$	0.9987	0.2-35	0.1	0.4
**3**	3-*O*-(*E*)-caffeoyl-L-threonic acid	$y=-0.0024x^2+0.0577x+0.0053$	0.9995	0.2-12	0.1	0.3
**4**	2-*O*-(*E*)-caffeoyl-L-threonic acid	$y=-0.0004x^2+0.0400x+0.0074$	0.9989	0.2-35	0.1	0.4
**5**	Lycoperodine-1	$y=-0.0006x^2+0.0387x+0.0114$	0.9971	0.2-35	0.1	0.4
**6**	Chlorogenic acid	$y=-0.0109x^2+0.2568x+0.0126$	0.9998	0.2-12	0.1	0.3
**7**	Actinidioionoside	$y=-0.0013x^2+0.0762x+0.0342$	0.9937	0.2-12	0.1	0.3
**8**	Caffeic acid	$y=-0.0003x^2+0.0447x+0.0229$	0.9982	0.2-60	0.1	0.3
**9**	Cryptochlorogenic acid		Calibrated using compound **6** curve.			
**10**	3'-*O*-(*E*)-Feruoyl-*α*-sorbopyranosyl-(2'→1)-*α*-glucopyranoside	$y=-0.004x^2+0.0902x+0.0113$	0.9988	0.2-12	0.1	0.4
**11**	2-*O*-(*E*)-caffeoyl-D-glyceric acid	$y=-0.0004x^2+0.0477x+0.021$	0.9979	0.3-60	0.2	0.6
**12**	4-*O*-(*E*)-caffeoyl-L-threonic acid	$y=-0.005x^2+0.1131x+0.0107$	0.9992	0.2-12	0.1	0.3
**13**	Neochlorogenic acid	$y=-0.0109x^2+0.2568x+0.0126$	0.9998	0.2-12	0.1	0.3
**14**	3-*O*-(*E*)-caffeoyl-D-glyceric acid	$y=-0.0006x^2+0.0717x+0.0267$	0.9986	0.2-60	0.1	0.3
**15**	3-*O*-p-coumaroyl-quinic acid	$y=-0.0015x^2+0.0967x+0.0358$	0.9959	0.2-35	0.1	0.4
**16**	4-*O*-p-coumaroyl-quinic acid		Calibrated using compound **15** curve.			
**17**	5-*O*-p-coumaroyl-quinic acid		Calibrated using compound **15** curve.			
**18**	Globoidnan B	$y=-0.0003x^2+0.0489x+0.0157$	0.9989	0.4-60	0.3	0.9
**19**	Rutin	$y=-0.0012x^2+0.0739x+0.0261$	0.9948	0.2-35	0.1	0.3
**20**	Nicotiflorin isomer	Calibrated using compound **24** curve.				
**21**	Quercetin 3-O-β-glucoside	$y=-0.0064x^2+0.1414x+0.0189$	0.9984	0.2-12	0.1	0.3
**22**	Yunnaneic acid E	$y=-0.0001x^2+0.0077x-0.001$	0.9984	0.5-60	0.4	1.2
**23**	Quercetin 3-*O*-(6''-*O*-malonyl)-*β*-glucoside	$y=-0.0004x^2+0.0265x+0.009$	0.9944	0.2-35	0.1	0.3
**24**	Nicotiflorin	$y=-0.0018x^2+0.1125x+0.0327$	0.9966	0.2-35	0.1	0.4
**25**	Astragalin	$y=-0.002x^2+0.1069x+0.0483$	0.9933	0.2-35	0.1	0.3
**26**	Shimobashiric acid C	$y=-0.0001x^2+0.0509x+0.0116$	0.9993	0.3-60	0.2	0.9
**27**	Rosmarinic acid	$y=-0.0001x^2+0.0509x+0.0116$	0.9993	0.2-12	0.1	0.3
**28**	Kaempferol 3-*O*-(6''-*O*-malonyl)-*β*-glucoside	$y=-0.0004x^2+0.0284x+0.0073$	0.9968	0.2-35	0.1	0.3
**29**	Monardic acid A	$y=-0.0001x^2+0.0017x+0.0032$	0.9858	0.5-60	0.5	1.5
**30**	Yunnaneic acid E-1		Not measured			
**31**	Lithospermic acid A	$y=-0.0001x^2+0.0095x+0.0059$	0.9997	0.2-60	0.3	0.9
**32**	Pulmonarioside A	$y=-0.0003x^2+0.0331x+0.0145$	0.9975	0.2-60	0.2	0.6
**33**	Salvianolic acid H	$y=-0.0005x^2+0.0683x+0.0135$	0.9991	0.2-60	0.1	0.3
**34**	Lithospermic acid B		Not measured			
**35**	Pulmonarioside B	$y=-0.0002x^2+0.0291x+0.0105$	0.9982	0.2-60	0.1	0.3
**36**	Yunnaneic acid B	$y=-0.0001x^2+0.0024x-0.0017$	0.9988	0.2-60	0.1	0.3
**37**	Globoidnan A	$y=-0.0072x^2+0.1572x+0.0108$	0.9995	0.2-12	0.1	0.3
**38**	Pulmitric acid A	$y=-0.0128x^2+0.2597x+0.0201$	0.9994	0.2-12	0.1	0.3
**39**	Pulmitric acid B	$y=-0.0026x^2+0.2597x+0.0201$	0.9954	0.2-35	0.1	0.3
**40**	Isosalvianolic acid A	$y=-0.0117x^2+0.2729x+0.0298$	0.9986	0.2-12	0.1	0.3
**41**	Isosalvianolic acid A-1		Calibrated using compound **40** curve.			
**42**	Isosalvianolic acid A isomer		Calibrated using compound **40** curve.			
**43**	Rosmarinic acid methyl ester	$y=-0.0154x^2+0.3333x+0.0683$	0.9973	0.2-12	0.1	0.3
**44**	Salvianolic acid H-9''-methylester	$y=-0.001x^2+0.0691x+0.0125$	0.9984	0.2-35	0.1	0.3
**45**	Lycopic acid C		Not measured			

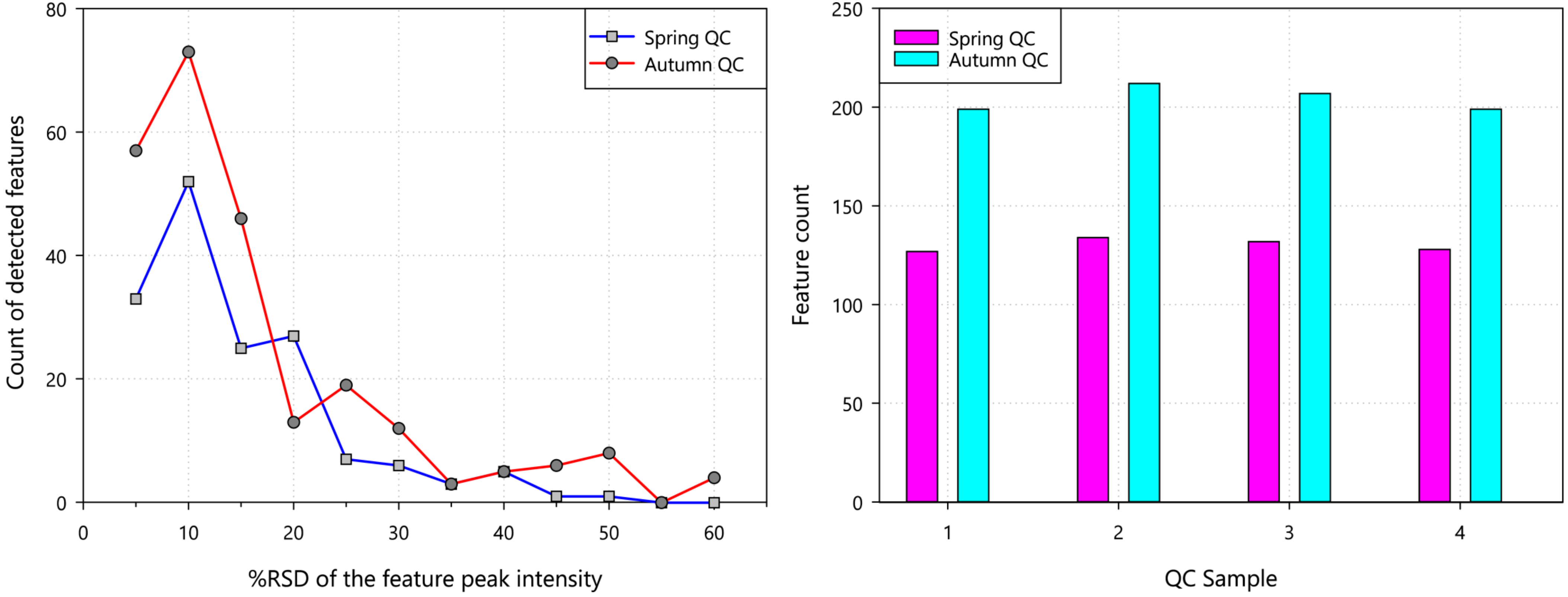

Figure 107S. Frequency distribution of the relative standard deviation for the peak intensities and peak numbers in QC samples
